# Multi-locus inherited neoplasia alleles syndromes in cancer: implications for clinical practice

**DOI:** 10.1038/s41431-025-01785-1

**Published:** 2025-01-23

**Authors:** Jeanette Yuen, Siqin Zhou, Rebecca Caeser, Mallika Venkatramani, Diana Nur Bte Ishak, Shao-Tzu Li, Zewen Zhang, Jianbang Chiang, Sock Hoai Chan, Joanne Ngeow

**Affiliations:** 1 Cancer Genetics Service, Division of Medical Oncology, National Cancer Centre, Singapore, Singapore; 2 Division of Clinical Trial & Epidemiological Sciences, National Cancer Centre, Singapore, Singapore; 3 Oncology Academic Clinical Program, Duke-NUS Medical School, Singapore, Singapore; 4 Lee Kong Chian School of Medicine, Nanyang Technological University, Singapore, Singapore

**Keywords:** Cancer genetics, Cancer genetics

## Abstract

The popularity of multi-gene testing has identified more families with two or more pathogenic variants (PV) in cancer predisposition genes, also known as ‘MINAS’ (multilocus inherited neoplasia alleles syndromes). They are at risk of suboptimal treatment and management as little on this topic is known. We conducted a systematic review of published MINAS cases within cancer predisposition genes to understand their association with more severe presentations. We analysed 413 MINAS carriers, which included 33 novel cases from the Cancer Genetics Service, National Cancer Centre Singapore. Statistical tests were conducted to assess association between carrier characteristics and the number PV identified. Results suggest that MINAS carriers have more malignancies (31.7% vs 21.5% vs 10.3% %; *p* < 0.001), a younger median age of first cancer diagnosis (40.0 vs. 44.0 vs. 49.0 years; *p* < 0.001) and an early onset of cancer (defined as <5% PV-associated cancer risk at age of diagnosis) (24.9% vs 7.7% vs 4.7%; *p* < 0.001) compared to monoallelic and non-carriers. We also studied the association of clinical characteristics by the dominant or recessive nature of PV harboured, where more dominant-dominant (D-D) carriers reported multiple malignancies (34.0%), compared to dominant-recessive (D-R) (23.9%) and recessive-recessive (R-R) carriers (20%;) (*p* = 0.051). Our findings suggest that MINAS carriers are prone to more and younger malignancies and the dominant or recessive nature of PV within double carriers can affect clinical presentation. We suggest a framework to guide management based on the dominant or recessive nature of PV within double PV carriers.

## Introduction

Hereditary cancer syndromes account for 5–10% of cancer, caused by pathogenic variants (PV) in cancer predisposition genes [1]. Carriers of these PV are at increased risk of developing cancers, usually at an earlier age. Identification of PV carriers is crucial to ensure carriers receive timely, specialised and multidisciplinary care for surveillance that will lead to early cancer detection and prevention [2].

In the past, PV was identified through single-gene testing but the advent of next-generation sequencing has enabled widespread multi-gene panel testing, leading to time- and cost-efficiencies [3] and the detection of more double heterozygotes (with ≥2 PV). In 2017, the incidence of double heterozygotes harbouring two cancer predisposition genes was reported at 0.2% [4] but a recent review by McGuigan, Whitworth [5] in 2022 suggested that this is likely an underestimation, considering most multi-PV cases are under-detected and under-reported.

The term ‘MINAS’ (multilocus inherited neoplasia alleles syndromes) was coined by Whitworth et al. referring to individuals who have two or more PV in cancer predisposition genes [6]. Their review of MINAS patients highlighted the possibility of suboptimal treatment, management and inaccurate cancer risk estimations of MINAS patients and families. A database of MINAS patients was created to consolidate these cases (https://databases.lovd.nl/shared/diseases/04296) [5, 6]; and as of Feb 2024, it contains 139 entries but contributions have been lacking to provide further insight on the impact of MINAS and optimal clinical management. The MINAS effect remains poorly characterised; it is unclear whether MINAS cases manifest more severe clinical presentations such as multiple or earlier onset malignancies.

Here, we reviewed 553 published MINAS cases to provide further insight on how to manage MINAS patients and to understand whether characteristics like an early onset cancer or a more malignancies are linked to MINAS. We also highlight 33 unpublished from our local service exhibiting MINAS in cancer predisposition genes.

## Methods

### Systematic review of MINAS cases

We followed the Preferred Reporting Items for Systematic Reviews and Meta-Analyses (PRISMA) guidelines for good reporting [7]. A systematic review of published literature within Medline and PubMed was conducted using a list of inherited cancer genes (*n* = 121) (Supplementary Table 1). This list was constructed based on a review of current cancer genes adapted from COSMIC to represent a comprehensive selection of clinically relevant cancer predisposition genes. Each gene within the list was entered as a search term to produce a list relevant to that gene. The entries are then combined with the “OR” operator to identify other articles that include another gene in the list, to generate 120 lists of articles referring to the given gene in combination with another cancer predisposition gene from the list. Each of these generated lists was subjected to the AND operator with keywords “germline” OR “germ-line” OR “germline mutation” OR “double heterozygosity” OR “double heterozygote” OR “double mutation” OR “double heterozygous mutation” OR “double germline mutation” OR “digenic” OR “MINAS” OR “Multilocus Inherited Neoplasia Alleles Syndrome”. Peer-reviewed articles published in English between 1 January 1995 and 31 Dec 2023 were selected. This timeframe reflects when genetic testing became available and panel testing became more accessible in the past decade [8]. All procedures followed were in accordance to the Declaration of Helsinki.

The resulting articles within the lists were then filtered manually (JY) to assess if it included a case of MINAS. Non-peer reviewed and non-English articles were excluded. The following variables were recorded: sex, ethnicity, clinical history, age of cancer diagnosis, and PV details. Individuals who had three PV were also recorded.

### Local MINAS and case reports

We conducted a retrospective study of individuals who underwent multi-gene panel testing at the National Cancer Centre Singapore, Cancer Genetics Service (CGS) between Jan 2015 and Nov 2023, to identify individuals with two or more PVs within cancer predisposition genes. This study is approved by SingHealth Centralized Institutional Review Board (CIRB 2018/2456). Written informed consent was taken for publication of genetic and clinical data. Multi-gene panel testing was carried out in Clinical Laboratory Improvement Amendments (CLIA)-certified laboratories. All individuals were provided with post-test (result) counselling and conception counselling. All MINAS carriers are managed via a personalised approach involving multi-disciplinary teams.

### Statistical analysis

Statistical analysis was conducted to explore possible predictors of MINAS. The local datasets of patients with one PV (monoallelic) (Supplementary Table 2) and patients with no PV (non-carriers) (Supplementary Table 3) were obtained and used as a basis for comparison against the consolidated database of MINAS cases (Supplementary Table 4). Variables such as age of cancer diagnosis, having an early onset of cancer (defined as <5% PV-associated cancer risk at age of diagnosis), number of cancer diagnoses, and having an atypical phenotype (defined as a phenotype not typically associated with the PV harboured by the carrier) were compared across three datasets (MINAS vs. monoallelic vs. non-carrier) to determine variables that were likely predictors of individuals with MINAS. The presence of atypical phenotypes was determined through the manual review of clinical histories. Categorical variables were summarized as fractions and percentages, whereas continuous variables were summarized using mean with standard deviation (SD), median with inter-quartile range (IQR) and range. Chi-squared test was performed for the following categorical variables (sex, number of cancers, having an early onset of cancer, atypical phenotypes and different cancer types). Fisher’s exact test was used to analyse smaller sample sizes of melanoma and brain cancer types. Kruskal-Wallis rank-sum test was performed to study association of first age of cancer diagnosis with the number of PV identified in a patient. These tests were also used to study associations of clinical characteristics within double PV carriers by gene types, defined by dominant or recessive inheritance: dominant-dominant (D-D), dominant-recessive (D-R), or recessive-recessive (R-R). Genes associated with both dominant and recessive disorders, such as *ATM*, were considered dominant for the analysis. A two-sided *p*-value less than 0.05 was considered statistically significant. All analyses were performed using R software (version 4.2.0).

## Results

### Correlation of MINAS carriers with clinical characteristics

Our systematic review identified 572 MINAS individuals based on our search criteria, where our local service contributed 33 MINAS cases. Of the 572 cases, 159 cases were omitted from statistical analysis for lack of detailed clinical histories (*n* = 99) or the lack of disease or any clinical presentations (*n* = 60) (Supplementary Table 5), resulting in a MINAS cohort of 413 individuals for analysis.

Among the 413 MINAS cases analysed (Tables 1), 83.1% (343/413) were females and 16.9% (70/413) males. Majority had a personal history of cancer (93.2%; 385/413), of which 61.5% (254/413) had one malignancy and 31.7% (131/413) were diagnosed with multiple malignancies at the time of reporting. There were 24.9% (103/413) MINAS cases with an early onset of cancer and 14.5% (60/413) cases presented atypical phenotypes not typically associated with any of the PV identified. We were unable to systematically evaluate ethnicity or family history of cancer as many published cases did not include these details.

**Table 1:** Patient demographics and clinical characteristics of negative, one PV and MINAS carriers.

	Negative (*N* = 1547)	One PV (*N* = 841)	MINAS (*N* = 413)	*p*-value
Sex				0.01
Female	1303 (84.2%)	667 (79.3%)	343 (83.1%)	
Male	244 (15.8%)	174 (20.7%)	70 (16.9%)	
Number of cancers				<0.001
0	95 (6.1%)	68 (8.1%)	28 (6.8%)	
1	1292 (83.5%)	592 (70.4%)	254 (61.5%)	
≥2	160 (10.3%)	181 (21.5%)	131 (31.7%)	
Age of first cancer diagnosis				<0.001^#^
Mean(SD)	49.2 (14.6)	44.8 (14.9)	40.9 (14.2)	
Median (IQR)	49.0 (39.0, 60.0)	44.0 (36.0, 55.0)	40.0 (33.0, 49.0)	
Range	1.0–91.0	0.0–88.0	0.0–87.0	
Early onset of cancer				<0.001
Yes	73 (4.7%)	65 (7.7%)	103 (24.9%)	
No	1474 (95.3%)	776 (92.3%)	310 (75.1%)	
Presence of atypical phenotypes				0.31
Yes		141 (16.8%)	60 (14.5%)	
No		700 (83.2%)	353 (85.5%)	

Cancer types *(those with cancer only)*	Negative (*N* = 1452)	One PV (*N* = 773)	MINAS (*N* = 385)	*p*-value*
Breast	805 (55.4%)	374 (48.4%)	250 (64.9%)	<0.001
Ovarian	266 (18.3%)	169 (21.9%)	56 (14.5%)	0.009
Colorectal	91 (6.3%)	81 (10.5%)	59 (15.3%)	<0.001
Prostate	75 (5.2%)	24 (3.1%)	5 (1.3%)	<0.001
Endometroid	69 (4.8%)	50 (6.5%)	21 (5.5%)	0.231
Thyroid	29 (2.0%)	18 (2.3%)	12 (3.1%)	0.417
Sarcoma	15 (1.0%)	20 (2.6%)	2 (0.5%)	0.003
Melanoma	7 (0.5%)	2 (0.3%)	18 (4.7%)	< 0.001
PGL/PCC	26 (1.8%)	27 (3.5%)	3 (0.8%)	0.004
Brain	3 (0.2%)	1 (0.1%)	7 (1.8%)	<0.001^
Others	155 (10.7%)	129 (16.7%)	44 (11.4%)	<0.001

*P*-value estimated using Chi-squared test unless otherwise stated.

*P-value was calculated within individuals with cancer only.

^^^*P*-value estimated using Fisher’s exact test.

^#^*P*-value estimated using Kruskal-Wallis test.

We compared 413 MINAS cases against 841 with one PV (monoallelic) and 1547 negative (non-carrier) cases from our local service and reported clinical features that are significantly associated with the MINAS dataset (Table 1). Overall, multiple primary cancers were observed more frequently in MINAS patients (31.7%; 131/413) compared to patients with one PV (21.5%; 181/841) and non-carriers (10.3%; 160/1547) (*p* < 0.001). A younger median age of cancer onset (40.0 years) was observed among MINAS patients compared to one PV (44.0 years) and non-carriers (49.0 years) cohort (*p* < 0.001). Additionally, a greater proportion of MINAS cases were associated with an early age of cancer (24.9%; 103/413) compared to the one PV (7.7%; 65/841) and non-carrier (4.7%; 73/1547) cohorts (*p* < 0.001). We compared MINAS and one PV cohort to assess for correlation of atypical phenotypes but observed no statistical difference between MINAS (14.5%;60/413) and one PV (16.8%; 141/841, *p* = 0.31) groups.

Of the 413 MINAS cases, 396 individuals are double PV carriers and 17 individuals are triple PV carriers. Majority are D-D carriers (324/396), whereas 67 are D-R and the remaining 5 are R-R carriers (Table 2). We observed that a greater fraction of D-D carriers reported multiple malignancies (34.0%; 110/324), compared to D-R (23.9%; 16/67) and R-R (20%; 1/5) group (*p* = 0.051). Atypical phenotypes were the least common in D-D (13.3%; 43/324) compared to D-R (17.9%; 12/67) and R-R carriers (60.0%; 3/5) (*p*-value = 0.022). No statistically significant association was observed between an early onset of cancer or the age of first cancer diagnosis and the combination of D-D, D-R and R-R carriers. In R-R carriers, 2/5 presented an early onset of cancer, 3/5 presented with cancers and 1/5 developed three separate malignancies.

**Table 2:** Demographics and clinical characteristics amongst 2PV carriers by variant types.

	Dominant-Dominant (*N* = 324)	Dominant-Recessive (*N* = 67)	Recessive-Recessive (*N* = 5)	*p*-value
Sex				0.227
Female	266 (82.1%)	58 (86.6%)	3 (60.0%)	
Male	58 (17.9%)	9 (13.4%)	2 (40.0%)	
Number of cancers				0.051
0	18 (5.6%)	4 (6.0%)	2 (40.0%)	
1	196 (60.5%)	47 (70.1%)	2 (40.0%)	
≥2	110 (34.0%)	16 (23.9%)	1 (20.0%)	
First age of cancer diagnosis				0.783^#^
Mean(SD)	40.9 (14.5)	41.0 (12.5)	38.0 (8.9)	
Median (IQR)	40.5 (33.0, 50.0)	40.0 (32.5, 48.5)	36.0 (34.0, 40.0)	
Range	0.0–87.0	1.0–70.0	28.0–52.0	
Early onset of cancer				0.367
Yes	78 (24.1%)	20 (29.9%)	2 (40.0%)	
No	246 (75.9%)	47 (70.1%)	3 (60.0%)	
Presence of atypical phenotypes				0.022
Yes	43 (13.3%)	12 (17.9%)	3 (60.0%)	
No	281 (86.7%)	55 (82.1%)	2 (40.0%)	
Number of PV expressed phenotypically				<0.001
0	11 (3.4%)	7 (10.4%)	2 (40.0%)	
1	67 (20.7%)	47 (70.1%)	1 (20.0%)	
2	246 (75.9%)	13 (19.4%)	2 (40.0%)	

17 triple PV carriers were not included in this analysis.

*P*-value estimated using Fisher’s exact test unless otherwise stated.

^#^*P*-value estimated using Kruskal-Wallis test.

### Clinical demographics of MINAS cases identified in the local service

Thirty-three cases of MINAS from 32 families identified in our local cohort of patients (Table 3), majority of whom were female (84.8%; 28/33) and Chinese (69.7%; 23/33). Most had a personal history of cancer (87.9%; 29/33), of which 19/33 (57.6%) had one cancer and 10/33 (30.3%) had more than one cancer. Breast cancer was the most common cancer observed (48.5%; 16/33), followed by ovarian cancer (21.2%; 7/33). Eight individuals (24.2%) had an early onset of cancer. Among these local MINAS patients, most (19/33; 57.6%) presented with clinical features and malignancies consistent with only one PV gene. Moreover, most patients (25/33; 75.6%) reported a family history of cancer, of which 20 patients (60.6%) reported a family history suggestive of only one of the two PV they had. Demographics details of local MINAS cases are summarised in Table 3.

**Table 3:** Patient demographics of local MINAS patients.

Local MINAS patients (*n* = 33)		
Gender		
Female	28	84.8%
Male	4	12.1%
Ethnicity		
Chinese	23	69.7%
Malay	6	18.2%
Indian	1	3.0%
Others	3	9.1%
Tumour/Cancer Status		
Personal history of cancer	29	87.9%
One primary cancer	19	57.6%
>1 primary cancer	10	30.3%
Non-cancer tumour(s)^a^	8	24.2%
Types of cancer		
Breast	16	48.5%
Ovarian	7	21.2%
Endometrial	4	12.1%
Urothelial	2	6.1%
Colorectal	1	3.0%
Pancreatic	2	6.1%
Others	5	15.2%
First age of cancer diagnosis		
0–20	4	12.1%
21–40	13	39.4%
41–60	13	39.4%
61 and above	2	6.1%
Phenotypes		
Early onset age of cancer^b^	8	24.2%
Phenotype associated with both PVs	11	33.3%
Phenotype associated with only 1 PV	19	57.6%
Atypical phenotypes	2	6.1%
Family History		
Cancer family history indicative of 2 PVs	5	15.2%
Cancer family history indicative of 1 PV	20	60.6%
Cancer family history not indicative	8	24.2%
Consanguinity within family	2	6.1%

^a^Tumour type is not associated with any PV patient has.

^b^Early-onset defined <5% cancer risk at the age of diagnosis.

### Correlation of *BRCA1/BRCA2* MINAS carriers with clinical characteristics

Pathogenic variants within *BRCA1* and *BRCA2* genes represented the bulk of deleterious mutations identified in our MINAS review, at 41.1% (162/413) and 43.2% (170/413) respectively. We evaluated 89 cases of *BRCA1/BRCA2* double heterozygotes (21.5%; 89/413), however, none were contributed by our local service. In this cohort, 37.1% (33/89) were diagnosed with >1 primary cancer, among which 15.7% (14/89) were breast-ovarian cancers and 20.2% (18/89) were multiple breast primaries. There were 30.3% (27/89) cases diagnosed with an early age of onset. Six cases of *BRCA1/BRCA2* double heterozygotes were diagnosed with cancers outside of the usual spectrum of breast, ovarian, prostate and pancreatic cancers; three had colorectal cancer, one with gastric cancer, one with endometrial cancer and another with cervical cancer.

### Correlation of *BRCA1/2-*Lynch MINAS carriers with clinical characteristics

Pathogenic variants within Lynch syndrome genes (*EPCAM, MLH1, MSH2, MSH6 and PMS2*) were the next most common within our analysed MINAS cohort at 21.8% (90/413), with *MSH2* PVs being the most common (7.5%; 31/413) and *EPCAM* PVs are the least common (0.8%; 3/413). We evaluated 29 cases with Lynch-*BRCA1/2* variants, of which 34.5% (10/29) presented Lynch-associated malignancies only (colorectal, endometrial, bladder cancers and tubular adenomas), 20.7% (6/29) presented with *BRCA1/2*-related cancers only such as breast cancers and 44.8% (13/29) presented with malignancies associated with both Lynch and *BRCA1/2*. Fourteen cases (48.3%; 14/29) were diagnosed with more than one primary cancer and four cases (13.8%; 4/29) demonstrated an early onset of cancer. Additionally, 24.1% (7/29) reported atypical *BRCA1/2* or Lynch presentation, including renal cell carcinoma, acute myeloid leukaemia, non-Hodgkin’s lymphoma, and gastrointestinal stromal tumour. There was a single case of a triple heterozygote (*BRCA1/MLH1/MSH6*; Supplementary Table 4; case 69) who developed endometrial cancer at age 41 years.

## Discussion

Here, we reviewed a cohort of 413 MINAS cases involving 104 unique inherited cancer genes. A key purpose of this review was to understand the clinical characteristics among MINAS patients, to understand if they present with a more severe clinical expression [6, 9, 10]. Overall, our data indicates that MINAS carriers are significantly associated with having multiple malignancies and an early onset of cancer. These findings are consistent across the entire MINAS dataset and also within *BRCA1/BRCA2* and *BRCA1/2*-Lynch MINAS carriers. Our findings are different from a smaller review of 12 cases by Stradella, del Valle [10] whose data does not support the existence of more severe manifestations in MINAS. However, they describe two cases who have PVs in moderate to low breast cancer risk genes (*ATM/PALB2*; case 119, *ATM/FANCA*; case 122; Supplementary Table 4) with bilateral breast cancer at age 35 and breast cancer at age 35 respectively – highlighting multiple and young onset cancers. Additionally, a recent review by McGuigan, Whitworth [5] found 28% (108/385) of MINAS cases presented multiple primary tumours, which is consistent with our and Whitworth, Skytte [6] findings that MINAS may present with a phenotype that is more severe than when a single PV is present.

Nine (9/89; 10.1%) of the *BRCA1/BRCA2* MINAS cases within this review were reported by Leegte, van der Hout [11] who reported a younger median age of cancer onset in *BRCA1/BRCA2* MINAS at 40.8 years vs. 48 years for single *BRCA1/BRCA2* carriers. A younger median of cancer was similarly reported in Koreans by Bang, Kwon [12] (33.7 years) and Hur, Kim [13] (36 years) when compared to single *BRCA1/BRCA2* carriers. In a German study comparing *BRCA1/BRCA2* MINAS and their single PV female relatives, Heidemann, Fischer [14] reported that *BRCA1/BRCA2* MINAS were substantially younger at onset of first cancer (40.4 years) than their single heterozygous female relatives (51.9 years) and they also manifested more severe disease (1.4 vs. 0.6 manifestations per person). These findings in *BRCA1/BRCA2* MINAS, representing 21.5% of our cohort, seem to reflect overall findings that MINAS are associated with younger ages of diagnosis and were more likely to be diagnosed with more than one primary cancer.

This effect was similarly observed in MINAS patients with two PV in breast cancer genes (*ATM, BRCA1, BRCA2, CHEK2, NBN*) which are known to increase breast cancer risk at a younger age, and in some cases can also be accompanied by multiple malignancies [15–17]. Three studies reported on the mechanisms behind this. Bowen, Yakushiji [18] demonstrated that *ATM/BRCA1* double heterozygote mice experience greater severity of mammary gland cancer, reduced ductal branching, suggesting a synergistic interaction driving carcinogenesis [18]. Further in-vitro studies of *ATM/BRCA1* double heterozygosity led to higher cell transformation rates, delayed recognition of DNA damage, aberrant DNA repair and increased genomic instability which promotes susceptibility to tumour initiation [19, 20]. We have 21 cases of *ATM/BRCA* MINAS in our cohort, 80.9% (17/21) of whom were diagnosed before the age of 50 (range 32 – 76 years) and 28.6% (6/21) were diagnosed with multiple cancer primaries. We report one case of an *ATM/BRCA1/MUTYH* MINAS (case 261) who was diagnosed with synchronous melanoma and ovarian cancer aged 46 and breast cancer aged 49.

There is also ongoing interest in the *TP53* MINAS cases with other cancer predisposition genes. In our cohort, we have 15 *TP53* MINAS cases, 44.4% (8/15) were diagnosed with multiple cancer primaries and 46.7% (7/15) were diagnosed with cancer before age 40. One *BRCA2/TP53* MINAS carrier developed four different primary cancers (case 35; melanoma aged 65, breast and ovarian cancer aged 65, colon cancer aged 74), which happens to only 2% of Li-Fraumeni Syndrome patients [21]. There are also cases of breast cancer diagnosed under age 34, which is the median diagnosis age of LFS-associated breast cancer [22]. Within our cohort, one *BRCA2/TP53* MINAS developed her first breast cancer aged 31 (case 34; Supplementary Table 4), another breast cancer aged 66 and a leiomyosarcoma aged 71. A *BRCA1/TP53* MINAS was diagnosed with two triple-negative breast cancers aged 20 (case 91; Supplementary Table 4). The mechanism for accelerated tumorigenesis may be explained molecularly, where BRCA1 and p53 have been shown to act in concert for tumour suppression. Upon genotoxic stress, p53 plays a role in mediating cell cycle arrest, allowing for DNA repair facilitated by BRCA1 [23, 24], thereby enabling the faithful repair of DNA damage. In patients with germline *TP53* and *BRCA1* PV, it is conceivable that the stacked defects of p53 and BRCA1 at cellular level would result in deficiencies at multiple checkpoints in DNA damage response and repair, leading to greater vulnerability for genomic instability and tumorigenesis. Therefore, these studies are consistent with our findings that *BRCA/TP53* MINAS cases may demonstrate more severe manifestations with multiple malignancies starting at younger ages.

Similarly, understanding the clinical presentations of *BRCA*/Lynch MINAS can guide the clinical care of these carriers. The majority of BRCA/Lynch MINAS developed with both Lynch- and *BRCA1/2*-associated cancers and presented with multiple malignancies over their lifetime. Functional studies have found overlap between the *BRCA1/2*-mediated DNA homologous recombination (HR) pathway and Lynch genes-encoded DNA mismatch repair (MMR) proteins. Within the HR pathway, BRCA1 interacts with MMR proteins to maintain genomic stability by triggering DNA repair [25]. The germline loss of BRCA1/2 or MMR protein function is expected to decrease proficiency of cellular DNA damage response and repair, contributing to accelerated genome instability. This could explain a possible synergistic effect driving carcinogenesis, hence it imperative that BRCA/Lynch carriers are managed and screened for the whole spectrum of cancers associated with *BRCA1/2* and Lynch Syndrome.

There was a recurrent *BRCA1* PV observed in our cohort local; NM_007294.4:c.2726dup (p.Asn909fs) that was found in patients 297 and 298. It was previously reported to be a known founder mutation to the Malays in Singapore and Malaysia [26]. In the SG10k study, where 10,000 unselected individuals received whole genome sequencing, this Malay founder variant was seen more frequently among the unselected Malay population [27] but a further statistically-powered analysis is needed to show that this variant is enriched among our HBOC cases over the general population.

As multi-gene panel testing, exome or whole-genome sequencing increasingly becomes the standard of care, it is crucial to publish more comprehensive guidance on the management of MINAS carriers. Here, we report a prevalence rate of 1.4% (33/2421) of MINAS cases within our local population, which may be an over-estimation considering multi-gene panel testing was only accessible within Singapore from around 10 years ago. Nonetheless, Narayanan, Udyawar [9] cite a higher prevalence rate of 2.4% of MINAS cases in families who underwent whole exome sequencing in India. There is a need to consolidate cases, as suggested by Whitworth, Skytte [6], within a MINAS database to track and elucidate the impact of specific combinations to guide the management of such individuals.

Like all studies, there are inherent limitations to our findings. Firstly, as the local cohort of 33 MINAS was a small sample size, the consolidated MINAS cohort was studied against local cohorts of one PV and non-PV carriers and our findings may come with limitations in view of the heterogeneity amongst these cohorts. We also acknowledge that there may be inherent bias as patients with a more severe presentation may be offered broader gene panel testing which increases the likelihood of more PV detected. The exclusion of MINAS cases without features (i.e. any disease or cancer presentations) may have resulted in an over-estimation of the association of a more severe phenotype in double PV carriers of cancer predisposition genes. Nonetheless, they represent a minority (27.8%; 159/572) and may represent individuals in whom features have yet to show up. The combinations of MINAS in *BRCA1/2* and *BRCA*/Lynch made up the majority of the cases identified and likely reflect ascertainment and testing bias, as these genes are commonly screened for simultaneously in response to the cancers that are prevalent worldwide like breast and colorectal cancers [28–30]. In the local MINAS cohort, there were PVs in *BRCA1* and *BARD1* that were shared by two patients who appear to be from different families through self-reported pedigrees verification, but we were unable retrospectively to verify if they are unrelated genetically as our patients undergo panel-based testing. Lastly, while the review includes a sizeable cohort, but numbers are lacking in specific combinations of MINAS making it hard to draw conclusions relevant to specific MINAS combinations and our findings are preliminary and would benefit from bigger cohort studies to confirm that MINAS is associated with a more severe presentation with multiple malignancies developing at young ages.

### Clinical implications

Our findings highlight the dangers of following Occam’s razor [31], to favour a single diagnosis over multiple diagnoses acting in concert to explain a patient’s clinical presentations; instilling the importance of testing all differentials relating to each presentation exhibited by a patient. Our local cases suggested that family history is not a predictor of MINAS, hence, it is important to include differentials arising from one’s cancer and family history to avoid overlooking MINAS.

**Fig. 1:**
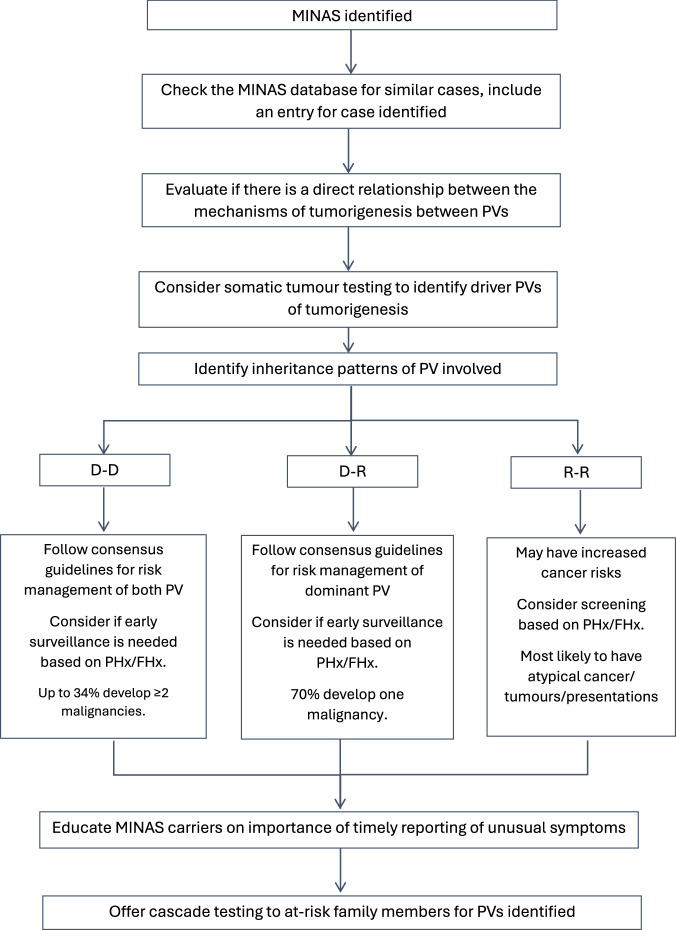
Framework to guide the clinical management of MINAS. D-D Dominant-Dominant; D-R Dominant-Recessive; R-R Recessive-Recessive. PHx personal history of cancer; FHx family history of cancer.

This is the first study to have evaluated the effect of MINAS by the dominant or recessive nature of the PVs harboured by MINAS individuals. Based on our findings we suggest a framework to guide clinical management of MINAS based on the dominant or recessive nature of the PV harboured (Fig. 1). First, check the MINAS database for cases with the same combination of PV, and include an entry for the case identified. Identifying previous reports of the same MINAS can provide insight on presentation of disease and age at which screening should begin. Evaluate if there is a direct relationship between the mechanisms of tumorigenesis of PVs identified through a review of existing literature (including mechanism models and other case reports) and if that exists, then there is plausible concern that a more severe presentation may entail. For example, a direct relationship between the mechanisms of tumorigenesis of two PV (e.g. *APC* and MMR gene mutations) may also point to a more severe clinical presentation [5, 6]. Hence, individuals with such combinations of PV may benefit from a personalised surveillance regime of earlier, more frequent health screenings, guided by available family history data. Where possible, somatic tumour analysis should be offered to decipher loss-of-heterogeneity (LOH) expression of genes mutated, identifying driver mutations of tumorigenesis. Within our review, 14 MINAS cases underwent tumour testing, of which only two of the 15 cancers demonstrated LOH at both genes that had a PV. This finding seems to suggest that the MINAS effect is more likely additive, rather than synergistic. This is further corroborated by Rebbeck, Friebel [32] who reported LOH at only a single locus in four of 14 cancers reported in *BRCA1/BRCA2* MINAS cases, which suggests that tumours develop from a second hit at a single PV and that the MINAS effect in *BRCA1/BRCA2* MINAS is similarly, more likely to be additive. There may also be value in other tumour profiling strategies to explore mechanisms of tumourigenesis which include immunohistochemistry for the gene products of PV, microsatellite instability testing (MSI) and cancer mutational signature analysis [33–35].

For D-D carriers, they can be expected to have multiple malignancies and would benefit from risk management strategies applicable to both dominant PVs detected. For D-R carriers, they should be offered cancer risk management based on the dominant PV and the majority of them are expected to develop one cancer that is usually associated with the dominant PV. Though our findings were not statistically significant but R-R carriers reported the highest proportion of early onset cancer and atypical phenotypes, and should be advised that while cancer/tumour risk is not yet clarified but may be elevated. Nonetheless, as the cancer/tumour risk to MINAS remains poorly elucidated, all MINAS carriers should be encouraged to report any unusual symptoms on a timely basis. Cascade testing of all at-risk adult family members is recommended for dominant PVs detected, and minors should be recommended testing if any dominant PVs detected include childhood cancers risk [36].

Genetic counselling will need to account for different risks of inheritance and options for reproductive choice and prenatal diagnosis for MINAS. The likelihood of inheritance within these families is higher. Two autosomal recessive conditions which segregate independently have a 56% chance of an unaffected child by both disorders [9]. However, genes that have a high chance of segregating together (like *NF1/TP53*) by being located close on the same chromosome will most likely be inherited together – resulting in a 25% chance of an affected child. In the case of patient 302 (Supplementary Table 4), where a single deletion event resulted in the deletion of *EPCAM* and *MSH2*, the chance of an unaffected child would be 50%. In a family with two autosomal dominant conditions, the chance of an unaffected child would be 25%. These rates are also affected by consanguinity within a family. Therefore, identification MINAS would not only require personalised cancer risk management for individuals but also has wider implications on genetic testing and counselling for at-risk family. Clinical management of MINAS patients should be facilitated within a multi-disciplinary team with input from physicians, genetic counsellors and researchers.

## Conclusions

Our findings add insight to the understanding of clinical presentations within MINAS. A greater proportion of MINAS was observed with more severe presentations, with multiple malignancies starting at younger ages and a higher frequency of early onset cancers—suggesting the need for a personalised surveillance regime for MINAS families. Moreover, carriers of D-D PVs had the highest proportion of 2 or more primary cancers. We proposed a framework to guide the management of MINAS based on our findings arising from the dominant/recessive nature of PVs and this should be facilitated within a multi-disciplinary team with input from physicians, genetic counsellors and researchers. As multi-gene, exome and genome sequencing become more common, bigger and deeper studies to understand the impact of varying combinations of PV would be necessary to guide the management of MINAS.

## Supplementary information

Supplementary Table 1: List of cancer genes used for literature search (*N* = 121)

Supplementary Table 2: One PV carriers

Supplementary Table 3: Non-carrier / Negative cases

Supplementary Table 4: MINAS

Supplementary Table 5: Omitted Cases

Supplementary Materials 6: References for the MINAS cohort

## Data Availability

The data that supports the findings of this study are available in the supplementary material of this article.
