# Supplementary material for: Multi-locus inherited neoplasia alleles syndromes in cancer: implications for clinical practice
Source: Eur J Hum Genet. 2025 Jan 23;33(3):289–96. doi: 10.1038/s41431-025-01785-1 (PMC11894078; doi:10.1038/s41431-025-01785-1)
Supplement: Supplementary file 1 — Supplementary Table 1: List of cancer genes used for literature search (N = 121) [file 41431_2025_1785_MOESM1_ESM.pdf]

Supplementary Table 1: List of Cancer Genes used for Literature Search (N = 121)

AIP	EGFR	KDR	RB1	WAS
ALK	EPCAM	KIT	RECQL4	WRN
APC	ERBB4	MAX	RET	WT1
AR	ERCC2	MEN1	RHBDF2	XPA
ATM	ERCC3	MET	RUNX1	XPC
ATR	ERCC4	MLH1	SBDS	
AXIN2	ERCC5	MPL	SDHA	
BAP1	EXT1	MSH2	SDHAF2	
BARD1	EXT2	MSH6	SDHB	
BLM	EZH2	MUTYH	SDHC	
BMPR1A	FANCA	NBN	SDHD	
BRCA1	FANCB	NF1	SETBP1	
BRCA2	FANCC	NF2	SLX4	
BRIP1	FANCD2	NSD1	SMAD4	
BUB1B	FANCE	PALB2	SMARCB1	
CDC73	FANCF	PDGFRA	SMARCE1	
CDH1	FANCG	PHOX2B	SPOP	
CDK4	FANCI	PMS1	STAT3	
CDKN1B	FANCL	PMS2	STK11	
CDKN1C	FANCM	POLD1	SUFU	
CDKN2A	FAT1	POLE	TERT	
CEBPA	FH	POLQ	TGFBR2	
CEP57	FLCN	PRF1	TMEM127	
CHEK2	GATA2	PRKAR1A	TP53	
CXCR4	GPC3	PTCH1	TP63	
CYLD	HNF1A	PTEN	TSC1	
DDB2	HRAS	PTPN13	TSC2	
DICER1	LMO1	RAD51C	TSHR	
DIS3L2	LZTR1	RAD51D	VHL	

A list of inherited cancer genes (n=121) was constructed based a review of current cancer genes adapted from the Cancer Gene Census and commercially available multi-cancer panels to represent a comprehensive selection of clinically relevant cancer predisposition genes.
