# Supplementary material for: Multi-locus inherited neoplasia alleles syndromes in cancer: implications for clinical practice
Source: Eur J Hum Genet. 2025 Jan 23;33(3):289–96. doi: 10.1038/s41431-025-01785-1 (PMC11894078; doi:10.1038/s41431-025-01785-1)
Supplement: Supplementary file 2 — Supplementary Table 2: One PV carriers [file 41431_2025_1785_MOESM2_ESM.pdf]

Supplementary Table 2: One PV carriers

S/N	**CGS ID**	**Ethnicity**	**Sex**	**PV HGVS**	**Clinical Hx with age dx (if known)**
1	CGS0714-01-001.CST	Chinese	F	NM_000038.6(APC):c.646C>T	Colorectal ca 32y
2	CGS1858-01-001.SBA	Malay	F	NM_000179.3(MSH6):c.2307delT	Colorectal ca 48y
3	CGS2929-01-001.TLT	Chinese	F	NM_000059.3(BRCA2):c.631+1G>A	Breast ca 43y
4	CGS1566-01-001.LOK	Chinese	M	NM_000077.5(CDKN2A):c.41_43delinsG	Pancreatic ca 51y
5	CGS1637-01-001.RBA	Malay	M	NM_000249.3(MLH1):c.1658_1667+3del13	Colorectal ca 47y
6	CGS2141-01-001.JCF	Caucasian	F	NM_007294.4(BRCA1): c.(?_?)del (exon 21)	Breast ca 40y
7	CGS1126-01-001.KR	Indian	M	NM_000059.3(BRCA2):c.1964delC	Prostate ca 41y
8	CGS3289-01-001.GA	Indian	F	NM_000249.3(MLH1): c.(?_?)dup (exons 4_6dup)	Endometrial ca 45y
9	CGS3255-01-001.MST	Filipino	F	NM_007294.4(BRCA1):c.4065_4068del	Breast ca 41y<br>Ovarian Cyst,<br>Tuberculosis, Groin Cyst
10	CGS4142-01-001.TN	Indian	F	NM_000059.3(BRCA2):c.5066del	Breast ca 34y
11	CGS4241-01-001.ANB	Burmese	F	NM_007294.4(BRCA1):c.3770_3771del	Breast ca 35y
12	CGS3892-01-001.S	Indonesian	M	NM_002878.3(RAD51D):c.270_271dup	Prostate ca 70y
13	CGS3162-01-001.UR	Indian	F	NM_007294.4(BRCA1):c.2735_2738del	Breast ca 64y, Ovarian ca 64y
14	CGS2086-01-001.KN	Chinese	F	NM_007294.4(BRCA1):c.2354T>A	Ovarian ca 58y
15	CGS4154-01-001.LSP	Chinese	F	NM_000059.3(BRCA2):c.5576_5579del	No cancer 50y; Left breast lump
16	CGS4081-01-001.SBL	Nepali	F	NM_007294.4(BRCA1):c.2214dup	Ovarian ca 53y<br>Uterine fibroid
17	CGS3632-01-001.NTP	Vietnamese	M	NM_000535.4(PMS2):c.746_753del	Colorectal ca 38y<br>Leukopenia
18	CGS3071-01-001.ERC	Filipino	M	NM_001379610.1(SPINK1):c.101A>G	Pancreatic ca 56y
19	CGS2380-01-001.TTP	Chinese	M	NM_000551.3(VHL):c.(?_?)fs (exon 1)	Haemangioblastoma 34y
20	CGS2325-01-001.LMP	Chinese	F	NM_000059.3(BRCA2):c.9143delT	Breast ca 42y
21	CGS2367-01-001.LKF	Chinese	M	NM_000551.3(VHL): g.(?_?)del (5'UTR to exon 2)	Retinoblastoma 2y,<br>Kidney ca 33y,<br>Haemangioblastoma 39y

22	CGS2346-01-001.TKK	Chinese	M	NM_000038.6(APC):c.646C>T	Desmoid Fibromatosis 39y;<br>Duodenal & Gastric Polyps
23	CGS2316-01-001.FBM	Malay	F	NM_007294.4(BRCA1):c.2726dupA	Breast ca 43 & Ovarian ca 46y
24	CGS2347-01-001.TAL	Chinese	F	NM_000143.3(FH):c.1189G>A	Kidney ca 40y
25	CGS0013-01-001.BBT	Vietnamese	F	NM_007294.4(BRCA1):c.4997dupA	Ovarian ca 54y
26	CGS0147-01-001.GBM	Malay	M	NM_130799.2(MEN1):c.467	Carcinoid thymus tumour 60y
27	CGS0024-01-001.KCM	Chinese	M	NM_130799.2(MEN1):c.1579C>T	Thymoma 39y, Neuroendocrine Tumor 39y
28	CGS0025-01-001.SS	Thai	F	NM_000038.6(APC):c.5826_5829delCAGA	Desmoid Fibromatosis 26y
29	CGS0234-01-001.MWK	Chinese	M	NM_000251.2(MSH2):c.1457_1460delAT<br>GA	Colorectal ca 53y
30	CGS0257-01-001.LBR	Malay	F	NM_007294.4(BRCA1):c.2726dupA	Breast ca 59y
31	CGS0263-01-001.BBA	Malay	M	NM_000077.4(CDKN2A): c.(?_?)del<br>(entire coding sequence)	Squamous cell carcinoma 38y,<br>Sarcoma 38y,<br>Nerve sheath tumour 38y
32	CGS0263-01-002.BBA	Malay	M	NM_000077.4(CDKN2A): c.(?_?)del<br>(entire coding sequence)	Left thigh peripheral nerve sheath tumour<br>38y,<br>lung cancer 47y,<br>left cerebellar tumour 47y
33	CGS0268-01-001.WEE	Chinese	F	untraceable	Paraganglioma 24y
34	CGS0269-01-001.RTW	Caucasian	M	NM_004360.3(CDH1):c.1003C>T	Gastric ca 32y
35	CGS0273-01-001.TTH	Chinese	M	NM_000179.3(MSH6): c.(?_?)del (exons<br>1_2del)	Colorectal ca 28y
36	CGS0281-01-001.WYT	Chinese	F	NM_003000.2(SDHB):c.640C>T	Pheochromocytoma 18y
37	CGS0282-01-001.CPE	Chinese	F	NM_000059.3(BRCA2):c.1888_1889insAA	Breast ca 26y
38	CGS0282-02-001.CYS	Chinese	M	NM_000059.3(BRCA2):c.1888_1889insAA	Prostate ca 64y
39	CGS0288-01-001.SZ	Chinese	F	NM_000059.3(BRCA2):c.956dupA	Breast ca 43y
40	CGS0292-03-001.LFM	Chinese	F	NM_000546.6(TP53):c.743G>A	Sarcoma 14y,<br>Breast ca 36y,<br>Atypical spindle cell lipomatous tumour 35y,<br>Juvenile granulosa cell tumour 36y
41	CGS0026-01-001.KY	Chinese	F	NM_001113378.1(FANCI):c.3924+1G>A	Endometrial ca 31y
42	CGS0026-01-002.KJT	Chinese	M	NM_001113378.1(FANCI):c.3924+1G>A	Squamous cell carcinoma 31y

43	CGS0296-01-001.NTM	Vietnamese	F	NM_007294.4(BRCA1):c.1673_1674delAA	Ovarian ca 48y
44	CGS0027-01-001.MML	Chinese	F	NM_000059.3(BRCA2):c.7878G>A	Breast ca 52y
45	CGS0244-01-001.JCL	Chinese	F	NM_003002.3(SDHD):c.325C>T	Paraganglioma 31y
46	CGS0254-01-001.YLK	Chinese	F	NM_003073.5(SMARCB1):c.*82C>T	No cancer 43y; Benign schwannoma
47	CGS0303-01-001.KCK	Chinese	M	NM_003002.3(SDHD):c.10dupC	Pheochromocytoma 11y
48	CGS0024-01-003.FKW	Chinese	M	NM_130799.2(MEN1):c.1579C>T	Pancreatic ca 25y
49	CGS0024-02-001.KQH	Chinese	M	NM_130799.2(MEN1):c.1579C>T	Neuroendocrine tumour 59y
50	CGS0020-01-001.AHT	Chinese	F	NM_003000.2(SDHB):c.136C>T	Paraganglioma 16y
51	CGS0323-01-001.LAZ	Chinese	M	NM_000546.6(TP53):c.329G>C	Sarcoma 0y
52	CGS0326-01-001.MJS	United Arab Emirates	F	NM_000249.3(MLH1):c.2218dupA	Colorectal ca 30y
53	CGS0329-01-001.ELH	Chinese	M	NM_003002.3(SDHD):c.242delC	Paraganglioma 36y, Pheochromocytoma 38y
54	CGS0011-01-001.LKH	Chinese	F	NM_000059.3(BRCA2):c.8915delT	Ovarian ca 47y
55	CGS0337-01-001.TI	Chinese	F	NM_007294.4(BRCA1): c.(?_?)del (exon 2)	Ovarian ca 53y
56	CGS0012-01-001.LML	Chinese	F	NM_000059.3(BRCA2):c.8961_8964delGAGT	Breast ca 45y, Ovarian ca 67y
57	CGS0342-01-001.LL	Chinese	F	NM_000059.3(BRCA2):c.5681dupA	Breast ca 46y, Ovarian ca 52y
58	CGS0346-01-001.JBM	Malay	F	NM_007294.4(BRCA1):c.2726dupA	Ovarian ca 40y
59	CGS0640-01-001.TL	Chinese	F	NM_007294.4(BRCA1):c.3331C>T	Ovarian ca 57y
60	CGS0002-01-002.MBN	Malay	F	NM_007294.4(BRCA1):c.2726dupA	Breast ca 41y
61	CGS0349-01-001.TTH	Burmese	M	NM_000251.2(MSH2):c.1528C>T	Colorectal ca 33y
62	CGS0350-01-001.XFM	Chinese	F	NM_000059.3(BRCA2):c.5645C>A	Ovarian ca 36y
63	CGS0351-01-001.ABT	Malay	F	NM_000059.3(BRCA2):c.9027T>G	Breast ca 39y (R invasive ductal carcinoma TNBC)<br>Breast ca 49y (left ER/PR+ in situ breast carcinoma)<br>Ovarian ca 58y
64	CGS0014-01-001.TSK	Chinese	F	NM_000249.3(MLH1):c.1637_1641dupAGTTA	Colorectal ca 41y; Haematemesis
65	CGS0014-03-001.OYF	Chinese	F	NM_000249.3(MLH1):c.1637_1641dup	Colorectal ca 32y, Endometrial ca 32y
66	CGS0353-01-001.CST	Chinese	F	NM_007294.4(BRCA1):c.3661G>T	Ovarian ca 50y
67	CGS0355-01-001.MAF	Filipino	F	NM_000059.3(BRCA2):c.6566dupA	Breast ca 29y

68	CGS0015-01-001.MBS	Malay	F	NM_000251.2(MSH2):c.1216C>T	Endometrial ca 37y; Sebaceoma
69	CGS0358-01-001.NRB	Malay	F	NM_000059.3(BRCA2):c.5722_5723delCT	Breast ca 41y, Ovarian ca 43y
70	CGS0364-01-001.SGS	Chinese	F	NM_007294.4(BRCA1):c.4201C>T	Breast ca 52y
71	CGS0366-01-001.KSS	Sikh	M	NM_007194.3(CHEK2):c.58C>T	Kidney ca 45y
72	CGS0016-01-001.ABA	Malay	F	NM_000038.6(APC):c.3920_3924delTAAAA	Colorectal ca 33y
73	CGS0016-01-002.BBA	Malay	M	NM_000038.6(APC):c.3920_3924delTAAAA	Colorectal ca 37y
74	CGS0371-01-001.TYM	Chinese	F	NM_001113378.1(FANCI):c.3626_3627delGT	Breast ca 37y
75	CGS0375-01-001.CPH	Chinese	M	NM_002878.3(RAD51D):c.270_271dupTA	Perivascular Epithelioid Cell Tumour (PECOMA) 62y
76	CGS0376-01-001.NYY	Chinese	M	NM_003000.2(SDHB):c.620_621delTG	Paraganglioma 19y
77	CGS0377-01-001.MAA	Portuguese	F	NM_000251.2(MSH2):c.2152C>T	Endometrial ca 29y
78	CGS0382-01-001.LGW	Chinese	F	NM_007294.4(BRCA1):c.3910G>T	Ovarian ca 53y
79	CGS0017-01-001.LSY	Chinese	F	NM_000059.3(BRCA2):c.9294C>G	Breast ca 33y (left invasive ductal carcinoma ER/PR+)<br>Breast ca 45y (right in situ breast carcionma ER/PR/HER2 status unknown)
80	CGS0017-01-002.LSH	Chinese	F	NM_000059.3(BRCA2):c.9294C>G	Breast ca, Thyroid ca 41y
81	CGS0388-01-001.LL	Chinese	F	NM_000059.3(BRCA2):c.6486_6489delACAA	Breast ca 75y, Ovarian Ca 77y, Squamous Cell Carcinoma 77y
82	CGS0390-01-001.TLC	Chinese	F	NM_000051.4(ATM):c.5697C>A	Breast ca 49y
83	CGS0403-01-001.CLP	Chinese	F	NM_000251.2(MSH2):c.1147C>T	Endometrial ca 37y<br>Kidney ca 39y<br>Gastric ca 49y<br>Squamous Cell Carcinoma 51y<br>Bladder ca age unknown<br>Rectosigmoid colon polyp - Tubular adenoma with LGD
84	CGS0407-01-001.LGC	Chinese	F	NM_130799.2(MEN1):c.1243C>T	Pituitary tumour 51y; Left parathyroid adenoma, Pituitary adenoma

85	CGS0409-01-001.PSL	Chinese	F	NM_007294.4(BRCA1):c.442-22_442-13del	Ovarian ca 51y
86	CGS0010-01-001.TCL	Chinese	F	NM_007294.4(BRCA1):c.2635G>T	Ovarian ca 44y
87	CGS0417-01-001.ECL	Chinese	F	NM_000314.4(PTEN):c.71A>G	Breast ca 33y, Desmoid Fibromatosis 42y, Endometrial ca 43y
88	CGS0419-01-001.EYT	Chinese	M	NM_000267.3(NF1):c.5242C>T	No cancer 49y; Neurofibroma
89	CGS0420-01-001.YBI	Malay	F	NM_000546.6(TP53):c.448_460del	Breast ca 25y (R invasive ductal carcinoma TNBC),<br>Breast ca 32y (R invasive ductal carcinoma ER/PR/HER2+)<br>Brease ca 42y (L breast Mixed adenoneuroendocrine carcinoma)<br>Mixed adenoneuroendocrine carcinoma HER2 equiv 42y
90	CGS0423-01-001.NSH	Chinese	F	NM_000249.3(MLH1):c.390C>G	Ovarian ca 37y, Colorectal ca 65y
91	CGS0427-01-001.LSS	Caucasian	M	NM_003000.2(SDHB):c.136C>T	Paraganglioma 56y
92	CGS0430-01-001.TYC	Chinese	M	NM_024675.3(PALB2):c.1059delA	Colorectal ca 46y
93	CGS1136-01-001.TLP	Chinese	F	NM_000546.6(TP53):c.742C>T	Squamous Cell Carcinoma 35y<br>Breast ca 36y<br>Choroid plexus papilloma,<br>Liver angiomyolipoma, Left breast excisions for fibroadenomas
94	CGS0996-01-001.GCP	Chinese	F	NM_007294.4(BRCA1):c.191G>C	Breast ca 38y, Ovarian ca 46y
95	CGS0431-01-001.TLC	Chinese	F	NM_000314.4(PTEN):c.406T>C	Breast ca 31y (L in situ breast carcinoma ER/PR+)<br>Breast ca 31y (R invasive ductal carcinoma ER/PR/HER2+)<br>Breast ca 42y (L IDC ER/PR+)
96	CGS1113-01-001.SAL	Caucasian	F	NM_007294.4(BRCA1):c.(?_?)dup (exon 12dup)	Breast ca 42y
97	CGS1135-01-001.LAL	Chinese	F	NM_000059.3(BRCA2):c.5164_5165delAG	Breast ca 45y, Ovarian ca 52y
98	CGS1141-01-001.HO	Burmese	M	NM_000038.6(APC):c.5524delT	Desmoid Fibromatosis 34y

99	CGS1145-01-001.GD	Indian	F	NM_001128425.1(MUTYH):c.721C>T	Colorectal ca 51y
100	CGS1085-01-001.CKM	Chinese	M	NM_000179.3(MSH6):c.3261dupC	Colorectal ca 63y
101	CGS0547-01-001.ZX	Chinese	F	NM_000059.3(BRCA2):c.9097delA	Breast ca 30y
102	CGS1152-01-001.THL	Chinese	F	NM_000059.3(BRCA2):c.9027T>G	Breast ca 52y, Ovarian ca 52y
103	CGS1174-01-001.MU	Indian	F	NM_007294.4(BRCA1):c.5211_5212delAG	Breast ca 44y
104	CGS1170-01-001.MMT	Arab	F	NM_000059.3(BRCA2):c.7666_7667insSVA	Breast ca 41y
105	CGS0910-01-001.LSK	Chinese	F	NM_002485.4(NBN):c.2083G>T	Ovarian ca 56y
106	CGS1227-01-001.NRN	Filipino	F	NM_000059.3(BRCA2):c.262_263delCT	Breast ca 32y
107	CGS1238-01-001.SWT	Chinese	F	NM_003002.3(SDHD):c.3G>C	Paraganglioma 29y
108	CGS1243-01-001.PTL	Vietnamese	F	NM_007294.4(BRCA1):c.5251C>T	Breast ca 42y
109	CGS1124-01-001.QCH	Chinese	F	NM_000179.3(MSH6):c.3514_3515delAG	Colorectal ca 39y
110	CGS1270-01-001.CSL	Chinese	F	NM_020975.4(RET):c.1901G>A	Thyroid ca, pheochromocytoma 46y
111	CGS1252-01-001.ABH	Malay	F	NM_007294.4(BRCA1):c.2517_2518delCA	Ovarian ca 58y, Endometrial ca 58y
112	CGS1282-01-001.LKT	Chinese	F	NM_000059.3(BRCA2):c.2808_2811delACAA	Breast ca 42y, Ovarian ca 53y, Non-small-cell lung carcinoma 48y
113	CGS1275-01-001.SC	Indian	F	NM_000179.3(MSH6):c.3476dupA	Endometrial ca 70y
114	CGS1280-01-001.LMH	Chinese	F	NM_000059.3(BRCA2):c.8915delT	Breast ca 50y
115	CGS1278-01-001.NBA	Malay	F	NM_020975.4(RET):c.1900T>C	Thyroid ca 24y, pheochromocytoma 25y
116	CGS1200-01-001.LSH	Chinese	M	NM_003000.2(SDHB): c.(?_?)del (exon 1)	Paraganglioma 65y
117	CGS1306-01-001.CHL	Chinese	F	NM_000059.3(BRCA2):c.8945_8946delAA	Ovarian ca 39y
118	CGS1313-01-001.WBS	Malay	F	NM_002878.3(RAD51D):c.343C>T	Ovarian ca 48y
119	CGS1248-01-001.KDR	Indian	F	NM_004360.3(CDH1):c.832+1G>A	Gastric ca 24y
120	CGS1274-01-001.TSH	Chinese	F	NM_007294.4(BRCA1):c.3381T>A	Breast ca 40y, Ovarian ca 64y
121	CGS1285-01-001.CGY	Chinese	F	NM_004168.3(SDHA):c.1A>G	Breast ca 43y
122	CGS1318-01-001.SHL	Chinese	F	NM_007294.4(BRCA1):c.81-2delA	Breast ca 38y
123	CGS0414-01-001.TGS	Chinese	F	NM_020975.4(RET):c.1888T>C	Thyroid ca 40y
124	CGS1425-01-001.KJK	Chinese	F	NM_007294.4(BRCA1):c.442-22_442-13del	Ovarian ca 48y
125	CGS1341-01-001.RA	Bangladeshi	F	NM_007294.4(BRCA1):c.5074+1G>A	Breast ca 41y
126	CGS1452-01-001.NKT	Chinese	M	NM_130799.2(MEN1):c.1193dupA	Pancreatic ca 33y

127	CGS1479-01-001.FA	Caucasian	F	NM_007294.4(BRCA1):c.213-11T>G	Melanoma 35y
128	CGS0920-01-001.GM	Chinese	F	NM_000051.4(ATM):c.3712_3716delTTATT	Breast ca 39y
129	CGS0767-01-001.SUB	Boyanese	F	NM_007294.4(BRCA1):c.2726dupA	Breast ca 38y, Ovarian ca 54y
130	CGS0050-01-001.HJB	Malay	F	NM_007294.4(BRCA1):c.2726dupA	Ovarian ca 52y
131	CGS0050-03-001.NNB	Malay	F	NM_007294.4(BRCA1):c.2726dupA	Breast ca 27y
132	CGS1224-01-001.ACL	Chinese	F	NM_000551.3(VHL):c.(?_?)del (exons 1_3del)	No cancer 34y; Liver hemangiomas & multiple pancreatic cysts,<br>Tiny haemangioblastoma & multiple pancreatic cysts
133	CGS1450-01-001.TCH	Chinese	F	NM_000465.3(BARD1):c.1833dupT	Breast ca 51y (R in situ breast carcinoma, ER+)<br>Breast ca 58y (L in situ breast carcinoma, ER/PR+)
134	CGS0061-01-001.TAK	Chinese	F	NM_000179.3(MSH6):c.3261dupC	Endometrial ca 56y
135	CGS0081-01-001.LD	Indian	F	NM_007194.3(CHEK2):c.58C>T	Breast ca 43y
136	CGS0080-01-001.MS	Indonesian	F	NM_007294.4(BRCA1):c.3257T>G	Breast ca 33y
137	CGS0083-01-001.ZBD	Malay	M	NM_000179.3(MSH6):c.3312delT	Colorectal ca 56y
138	CGS0086-01-001.TRL	Caucasian	F	NM_000465.3(BARD1):c.2099delG	Breast ca 34y (L medullary TNBC)<br>Breast ca 36y (R in situ breast carcinoma ER/PR/HER2 unknown)
139	CGS0091-01-001.FAX	Chinese	F	NM_000546.6(TP53):c.638G>A	Adrenal Cortical Carcinoma 1y
140	CGS1496-01-001.WTL	Chinese	F	NM_000251.2(MSH2): c.(?_?)del (exons 1_6del)	Endometrial ca 57y
141	CGS0096-01-001.CSP	Filipino	F	NM_000059.3(BRCA2):c.262_263delCT	Breast ca 40y
142	CGS0099-01-001.TST	Chinese	M	NM_000314.4(PTEN):c.1027-2A>G	No cancer 32y; Multiple colon polyps
143	CGS1489-01-001.TG	Indian	M	NM_000249.3(MLH1):c.350C>T	Colorectal ca 46y (sigmoid, malignant invasive adenocarcinoma)<br>Breast ca 46y (ascending right malignant invasive adenocarcinoma)
144	CGS0106-01-001.NT	Bangladeshi	F	NM_000546.6(TP53):c.817C>T	Sarcoma 17y
145	CGS0106-02-002.MSU	Bangladeshi	M	NM_000546.6(TP53):c.817C>T	Squamous Cell Carcinoma 50y; Brain lesion, Adrenal nodules

146	CGS0121-01-001.OXY	Chinese	F	NM_020975.4(RET):c.1900T>C	Thyroid ca 24y, pheochromocytoma 10y
147	CGS0134-01-001.NSF	Chinese	F	NM_206937.2(LIG4):c.1144_1145delCT	No cancer 51y; Aplastic anemia
148	CGS1115-01-001.CCH	Chinese	M	NM_003000.2(SDHB):c.136C>T	Paraganglioma 29y;<br>Left parathyroid adenoma<br>Tubular adenoma with low grade dysplasia
149	CGS0148-01-001.DVR	Vietnamese	M	NM_000251.2(MSH2):c.1786_1788delAAT	Colorectal ca 54y
150	CGS0150-01-001.ENS	Chinese	M	NM_000551.3(VHL):c.500G>A	Kidney ca 40y
151	CGS0168-01-001.TLL	Chinese	F	NM_000059.3(BRCA2):c.8915delT	Breast ca 34y
152	CGS0190-01-001.YTJ	Chinese	M	NR_001566.1(TERC):r.311C>T	No cancer 60y; Idiopathic pulmonary fibrosis, MDS
153	CGS0188-01-001.CGJ	Chinese	M	NM_003002.3(SDHD):c.3G>C	Pheochromocytoma 12y
154	CGS0196-02-001.WKS	Chinese	M	NM_000546.6(TP53):c.742C>T	Sarcoma 37y
155	CGS0196-02-002.IWM	Chinese	F	NM_000546.6(TP53):c.742C>T	Thyroid ca 41y
156	CGS0196-01-001.JWW	Chinese	M	NM_000546.6(TP53):c.742C>T	Medulloblastoma 12y; MDS
157	CGS0200-01-001.SS	Indian	F	NM_007294.4(BRCA1):c.3333delA	Ovarian ca 63y
158	CGS0177-01-001.CLM	Chinese	F	NM_007294.4(BRCA1):c.5072C>A	Breast ca 39y (L Invasive atypical medullary carcinoma PR+)<br>Breast ca 45y (R invasive ductal carcinoma TNBC)
159	CGS0285-01-001.CSY	Chinese	F	NM_007294.4(BRCA1):c.191G>C	Breast ca 41y
160	CGS0285-02-001.TM	Chinese	F	NM_007294.4(BRCA1):c.191G>C	Breast ca 50y (R, unknown histo) & 55y (L, unknown histo)
161	CGS0434-01-001.TTT	Vietnamese	F	NM_058216.2(RAD51C):c.955C>T	Breast ca 38y
162	CGS0246-01-001.BV	Caucasian	F	NM_007294.4(BRCA1):c.181T>G	Breast ca 33y
163	CGS0488-01-001.FST	Chinese	F	NM_003000.2(SDHB): c.(?_?)del (exons 1_2del)	Paraganglioma 51y
164	CGS0510-01-001.SG	Sri Lankan	F	NM_000059.3(BRCA2):c.1296_1297delGA	Ovarian ca 41y
165	CGS0506-01-001.SC	Chinese	F	NM_000051.4(ATM):c.9023G>A	Breast ca 44y
166	CGS0511-01-001.SSF	Chinese	F	NM_000059.3(BRCA2):c.3109C>T	Breast ca 43y

167	CGS0527-01-001.SBA	Malay	F	NM_000059.3(BRCA2):c.682-1G>C	Breast ca 30y (L, unknown histo)<br>Breast ca 53y (R invasive ductal breast carcinoma ER/PR/HER2+)
168	CGS0532-01-001.AMB	Indian	M	NM_000251.2(MSH2):c.1042C>T	No cancer 24y; Polyps
169	CGS0517-01-001.GSS	Caucasian	M	NM_000057.4(BLM):c.2207_2212delinsTAGATTC	Leukaemia 59y
170	CGS0535-01-001.ATH	Burmese	M	NM_000249.3(MLH1):c.982C>T	Colorectal ca 40y
171	CGS0503-01-001.LN	Chinese	F	NM_015450.2(POT1): c.(?_?)del (exons 5_6del)	Lacrimal gland carcinoma 22y,<br>Neuroendocrine tumour head of pancreas 27y
172	CGS0252-01-001.PW	Indonesian	F	NM_007294.4(BRCA1):c.5074+3A>T	Breast ca 40y
173	CGS0546-01-001.PN	Chinese	F	NM_000059.3(BRCA2):c.8208_8209insAG	Breast ca 31y
174	CGS0558-01-001.WKX	Chinese	F	NM_015338.6(ASXL1):c.2077C>T	No cancer 11y; Aplastic Anemia, Diabetes mellitus (type 1)
175	CGS0596-01-001.NMC	Chinese	F	NM_198253.3(TERT):c.2147C>T	No cancer 32y; Idiopathic pulmonary fibrosis, Bicytopenia
176	CGS0519-01-001.MEH	Filipino	M	NM_000321.3(RB1):c.399delC	Bilateral retinoblastoma 1y
177	CGS0633-01-001.BCA	Caucasian	M	NM_000551.3(VHL):c.353T>C	Haemangioblastoma 28y
178	CGS0617-01-001.CST	Chinese	F	NM_024675.3(PALB2):c.2167_2168delAT	Breast ca 59y
179	CGS0646-01-001.LHC	Chinese	M	NM_000249.3(MLH1):c.793C>T	Colorectal ca 39y
180	CGS0653-01-001.FCO	Chinese	F	NM_000249.3(MLH1):c.790+1G>A	Endometrial ca 57y
181	CGS0733-01-001.SBB	Malay	F	NM_000267.3(NF1):c.7096_7101del	Ovarian ca 45y Neurofibroma
182	CGS0765-01-001.RBA	Malay	F	NM_001128425.1(MUTYH):c.1435G>T	Gastrointestinal stromal tumour 52y
183	CGS0799-01-001.NHB	Malay	F	NM_002878.3(RAD51D):c.343C>T	Ovarian ca 37y
184	CGS0820-01-001.LST	Chinese	F	NM_007294.4(BRCA1):c.5072C>A	Breast ca 52y
185	CGS0554-01-001.HBM	Malay	F	NM_007294.4(BRCA1):c.981_982delAT	Breast ca 24y
186	CGS0828-01-001.YTP	Chinese	F	NM_007294.4(BRCA1):c.3228_3229delAG	Breast ca 41y
187	CGS0832-01-001.TKN	Chinese	F	NM_000249.3(MLH1):c.390C>G	Colorectal ca 54y, Endometrial ca 52y
188	CGS0832-01-003.TKH	Chinese	M	NM_000249.3(MLH1):c.390C>G	Colorectal ca 45y
189	CGS0842-01-001.TMK	Chinese	F	NM_007294.4(BRCA1):c.442-22_442-13del	Breast ca 37y
190	CGS0870-01-001.TYK	Chinese	M	NM_000249.3(MLH1):c.979C>T	Colorectal ca 32y, Lung cancer 53y

191	CGS0740-01-001.KCE	Chinese	F	NM_007294.4(BRCA1):c.442-22_442-13del	Breast ca 38y (L atypical medullary carcinoma, ER and hER2 unknown, PR-)<br>Breast ca 44y (R invasive ductal breast carcinoma ER/PR/HER2+)
192	CGS0740-02-001.OBK	Chinese	F	NM_007294.4(BRCA1):c.442-22_442-13del	Breast ca 48y & 73y
193	CGS0902-01-001.LKT	Chinese	F	NM_000051.4(ATM): c.(?_?)del (exons 62_63del)	Breast ca 56y
194	CGS0804-01-001.SNB	Malay	F	NM_000546.6(TP53):c.97-1G>T	Adrenal Cortical Carcinoma 2y
195	CGS0192-01-001.NBH	Malay	F	NM_000059.3(BRCA2):c.5583dupA	Breast ca 43y
196	CGS0977-01-001.LCY	Chinese	F	NM_000059.3(BRCA2):c.9154C>T	Ovarian ca 52y
197	CGS0766-01-001.BNK	Chinese	F	NM_002878.3(RAD51D):c.270_271dupTA	Colorectal ca 59y
198	CGS1072-01-001.NLW	Chinese	F	NM_024675.3(PALB2):c.(?_?)dup (exon 13dup, extending into 3' UTR)	Ovarian ca 55y
199	CGS0990-01-001.TSK	Chinese	F	NM_001407096.1(MAX):c.223C>T	Pheochromocytoma 31y
200	CGS1007-01-001.SL	Italian	F	NM_007294.4(BRCA1):c.3228_3229delAG	Breast ca 42y
201	CGS1060-01-001.GOG	Chinese	F	NM_002878.3(RAD51D):c.270_271dupTA	Breast ca 50y
202	CGS0790-01-001.TLE	Chinese	F	NM_000548.4(TSC2):c.3094C>T	No cancer 51y; Tuberous sclerosis complex associated lymphangioleiomyomatosis
203	CGS1071-01-001.KMY	Chinese	F	NM_000455.4(STK11):c.910C>T	No cancer 41y; Multiple small polyps
204	CGS0226-01-002.CLH	Chinese	F	NM_000059.3(BRCA2):c.7791delA	Ovarian ca 62y, Schwannoma 56y
205	CGS1520-01-001.OPC	Chinese	F	NM_007294.4(BRCA1):c.2635G>T	Breast ca 39y, Ovarian ca 48y
206	CGS1530-01-001.DLP	Chinese	F	NM_007294.4(BRCA1):c.442-22_442-13del	Ovarian ca 57y
207	CGS1529-01-001.TTT	Chinese	M	NM_000051.4(ATM):c.1995delT	Pancreatic ca 60y
208	CGS0165-02-001.HKK	Chinese	F	NM_000465.3(BARD1):c.1338C>A	Breast ca 47y (L invasive breast ductal carcinoma, ER/PR+)<br>Breast ca 62y (R invasive breast ductal carcinoma TNBC)
209	CGS0165-01-001.LWL	Chinese	F	NM_000465.3(BARD1):c.1338C>A	Breast ca 37y
210	CGS0945-01-001.CML	Chinese	F	NM_000267.3(NF1):c.964delA	Ovarian ca 41y
211	CGS0456-01-001.WSM	Chinese	F	NM_000059.3(BRCA2):c.5722_5723delCT	Breast ca 37y

212	CGS1535-01-001.WCQ	Chinese	F	NM_000059.3(BRCA2):c.9097delA	Breast ca 33y
213	CGS1536-01-001.TWJ	Chinese	M	NM_003002.3(SDHD):c.314+1G>A	Pheochromocytoma 19y
214	CGS1544-03-001.DMB	Malay	F	NM_000535.4(PMS2): c.(?_?)del (exons 6_7del)	Cervical cancer 31y, Mammary Analogue Secretory Carcinoma 36y
215	CGS1552-01-001.KLX	Chinese	M	NM_032043.2(BRIP1):c.2464dup	Squamous Cell Carcinoma 14y
216	CGS1570-01-001.LBT	Chinese	F	NM_000059.3(BRCA2):c.7976+5G>A	Breast ca 58y, Ovarian ca 66y
217	CGS1571-01-001.TMH	Chinese	F	NM_000249.3(MLH1): c.(?_?)del (entire gene)	Breast ca 40y (R invasive ductal breast carcinoma ER+, PR/HER2-)<br>Breast ca 46y (left, Invasive ductal breast carcinoma, ER+, PR/HER2-)<br>Endometrial ca 53y
218	CGS1572-01-001.WJ	Chinese	F	NM_004168.3(SDHA):c.1A>G (Initiator codon)	Breast ca 46y
219	CGS1572-03-001.TS	Chinese	F	NM_004168.3(SDHA):c.1A>G (Initiator codon)	Germ cell tumour 12y
220	CGS1583-01-001.ELH	Chinese	F	NM_000059.3(BRCA2):c.956dupA	Breast ca 44y
221	CGS1600-01-001.TR	Bangladeshi	F	NM_004168.3(SDHA):c.1258C>T	Breast ca 29y
222	CGS1604-01-001.SAM	Filipino	F	NM_022725.4(FANCF):c.484_485delCT	Breast ca 28y
223	CGS1541-01-001.JGH	Chinese	F	NM_007294.4(BRCA1):c.4782delA	Breast ca 27y
224	CGS1611-01-001.MAI	Italian	F	NM_000267.3(NF1):c.2851-1G>A	Breast ca 47y; Fibroma
225	CGS1644-01-001.WMA	Filipino	F	NM_000059.3(BRCA2):c.4631delA	Breast ca 48y
226	CGS1614-01-001.LCS	Caucasian (British)	F	NM_020975.4(RET):c.1900T>C	Thyroid ca 17y, pheochromocytoma 17y
227	CGS1643-01-001.TSC	Chinese	F	NM_000492.3(CFTR):c.1766+5G>T	Pancreatic ca 41y
228	CGS1669-01-001.FAZ	UAE	F	NM_000251.2(MSH2):c.1662-2A>G	Colorectal ca 36y
229	CGS1673-01-001.WH	Chinese	F	NM_007294.4(BRCA1):c.181T>G	Ovarian ca 50y
230	CGS1605-01-001.LBI	Malay	F	NM_000059.3(BRCA2):c.3680_3681delTG	Breast ca 38y
231	CGS1697-01-001.MS	Indonesian	F	NM_000249.3(MLH1):c.1377dup	Endometrial ca 54y
232	CGS1724-01-001.NKL	Chinese	F	NM_004360.3(CDH1):c.1901C>T	Breast ca 61y
233	CGS1723-01-001.PP	Indonesian	M	NM_000059.3(BRCA2):c.6405_6409del	Astrocytoma 32y
234	CGS1743-01-001.OFF	Chinese	F	NM_001128425.1(MUTYH):c.643G>A	Breast ca 43y
235	CGS1745-01-001.ZBM	Malay	F	NM_000059.3(BRCA2):c.2957dup	Breast ca 35y
236	CGS1752-01-001.TM	Indian	F	NM_007294.4(BRCA1):c.68_69del	Ovarian ca 46y
237	CGS1764-01-001.TPH	Chinese	F	NM_000059.3(BRCA2):c.8585_8586del	Breast ca 44y

238	CGS1774-01-001.CJD	Indian	F	NM_000059.3(BRCA2):c.6155C>A	Ovarian ca 51y
239	CGS1780-01-001.LLS	Chinese	F	NM_000059.3(BRCA2):c.9117G>A	Breast ca 41y (L, unknown histo) & 53y (R, unknown histo)
240	CGS1701-01-001.LSP	Chinese	F	NM_000267.3(NF1):c.3974G>A	Pheochromocytoma 56y; Primary hyperthyroidism ?, Cafe au lait, Scoliosis
241	CGS1701-03-001.WS	Chinese	M	NM_000267.3(NF1):c.3974G>A	No cancer 25y; Neurofibromas
242	CGS1814-01-001.ASH	Chinese	F	NM_004656.3(BAP1):c.852del	Breast ca 61y, Thyroid ca 65y, Kidney ca 66y
243	CGS1829-01-001.SCD	Chinese	M	NM_000267.3(NF1):c.7907+5G>C	No cancer 22y; Neurofibromas
244	CGS1833-01-001.NJY	Chinese	F	NM_024675.3(PALB2):c.2968G>T	Breast ca 38y
245	CGS1847-01-001.LL	Chinese	F	NM_004656.3(BAP1):c.1358_1359del	Breast ca 36y
246	CGS1849-01-001.KTF	Chinese	F	NM_000038.6(APC): c.(?_?)del (exon 12)	No cancer 52y; Multiple colon polyps
247	CGS1861-01-001.WX	Chinese	F	NM_024675.3(PALB2):c.3507_3508del	Breast ca 51y
248	CGS1873-01-001.SS	Sikh	M	NM_004656.3(BAP1):c.588G>A	Mesothelioma 53y
249	CGS1914-01-001.LPR	Chinese	F	NM_001113378.1(FANCI):c.2568_2569del	Breast ca 33y
250	CGS1913-01-001.GLY	Chinese	F	NM_007294.4(BRCA1):c.5470_5477del	Breast ca 43y
251	CGS1916-01-001.LTL	Chinese	M	NM_000492.3(CFTR):c.1210-34TG[12]T[5]	Pancreatic ca 47y
252	CGS1929-01-001.TTH	Chinese	M	NM_000038.6(APC):c.645+1G>T	No cancer 68y; Multiple gastric and colonic polyps
253	CGS1930-01-001.QMK	Chinese	F	NM_000249.3(MLH1):c.1852_1854del	Colorectal ca 46y
254	CGS1970-01-001.VKS	Chinese	F	NM_000553.4(WRN):c.502_503del	Breast ca 40y, Thyroid ca 38y, Left adrenal tumour 43y
255	CGS1979-01-001.YBH	Indian	M	NM_000267.3(NF1):c.3198-2A>G	Prostate ca 41y
256	CGS1988-01-001.FJ	Chinese	M	NM_000251.2(MSH2):c.1102G>T	Colorectal ca 44y, Pancreatic ca 59y
257	CGS1991-01-001.MQK	Pakistani	M	NM_000251.2(MSH2):c.1034G>A	Colorectal ca 29y
258	CGS2002-01-001.ZBM	Malay	F	NM_007294.4(BRCA1):c.140G>A	Breast ca 50y
259	CGS2009-01-001.TCC	Chinese	M	NM_000051.4(ATM):c.2284_2285del	Pancreatic ca 60y
260	CGS2027-01-001.CAL	Chinese	F	NM_000059.3(BRCA2):c.9177dup	Breast ca 35y
261	CGS1966-01-003.TJY	Chinese	M	NM_000321.3(RB1):c.59_71del	Sarcoma 0y
262	CGS2045-01-001.GD	Chinese	M	NM_003002.3(SDHD):c.3G>C	Pheochromocytoma 29y
263	CGS2045-02-001.GE	Chinese	M	NM_003002.3(SDHD):c.3G>C	Paraganglioma 39y, NPC 53y
264	CGS1722-01-001.AT	Chinese	F	NM_207122.2(EXT2):c.668G>C	Kidney ca 4y

265	CGS2065-01-001.QKL	Chinese	F	NM_058216.2(RAD51C):c.905-2A>C	Ovarian ca 60y
266	CGS1942-01-001.MPZ	Chinese	M	NM_000038.6(APC): c.(?_?)del (entire coding sequence)	No cancer 26y; Multiple colon polyps (>100)
267	CGS2075-01-001.MNB	Malay	M	NM_000267.3(NF1): c.(?_?)del (exon 50)	Juvenile myelomonocytic leukemia 0y<br>Supratentorial ependymoma 4y<br>Neurofibromatosis type 1 (clinical diagnosis)
268	CGS2093-01-001.JLT	Chinese	F	NM_000051.4(ATM):c.2999dupA	Breast ca 41y
269	CGS2097-01-001.OYJ	Chinese	F	NM_005732.3(RAD50):c.1111del	Ovarian ca 54y
270	CGS2099-01-001.TMK	Chinese	F	NM_000051.4(ATM):c.1433C>A	Ovarian ca 57y, Endometrial ca 57y
271	CGS2113-01-001.AKC	Chinese	M	NM_000179.3(MSH6):c.3573dup	Colorectal ca 64y
272	CGS2131-01-001.HHM	Burmease	F	NM_000059.3(BRCA2):c.794-2A>G	Breast ca 45y
273	CGS2122-01-001.SBK	Malay	F	NM_007294.4(BRCA1):c.2726dup	Breast ca 33y, Ovarian ca 37y
274	CGS2138-01-001.KML	Chinese	F	NM_058216.2(RAD51C):c.905-2A>C	Breast ca 47y (L invasive breast ductal carcinoma PR+, ER/HER2-)<br>Breast ca 59y (R invasive breast ductal carcinoma, ER/PR- HER2 equi)
275	CGS2178-01-001.TCJ	Chinese	M	NM_000038.6(APC): c.(?_?)del (entire coding sequence)	No cancer 25y; Colon polyps, Colon polyps
276	CGS3616-01-001.TCL	Chinese	F	NM_000038.6(APC): c.(?_?)del (promoters 1B and 1A, exons 2_16del)	Thyroid ca 15y
277	CGS2205-01-001.TPQ	Chinese	F	NM_024675.3(PALB2):c.3350+5G>A	Breast ca 26y
278	CGS2222-01-001.KBA	Malay	F	NM_000059.3(BRCA2):c.2808_2811	Breast ca 41y
279	CGS2227-01-001.MB	Bangladeshi	F	NM_000135.2(FANCA):c.3629dup	Breast ca 58y
280	CGS2229-01-001.JBB	Malay	F	NM_007294.4(BRCA1):c.134+3A>C	Ovarian ca 65y
281	CGS2421-01-001.MA	Indian	F	NM_000051.4(ATM):c.2667T>A	Breast Can 37y
282	CGS2224-01-001.TCJ	Chinese	M	NM_007194.3(CHEK2):c.1459C>T	Leukaemia 29y; MDS
283	CGS2249-01-001.YKC	Chinese	F	NM_000535.4(PMS2):c.1206del	Endometrial ca 66y
284	CGS2185-01-001.LS	Chinese	M	NM_007294.4(BRCA1):c.442-22_442-13del	Colorectal ca 56y<br>Multiple myeloma 64y<br>Non-small cell lung adenocarcinoma 65y<br>HCC 65y
285	CGS2158-01-001.KYT	Chinese	F	NM_000251.2(MSH2):c.1799C>T	Colorectal ca 28y

286	CGS2899-01-003.GSH	Chinese	F	NM_007194.3(CHEK2):c.1459C>T	No cancer 47y; Uterine fibroids, Ovary cyst, Breast cyst
287	CGS2912-01-001.ASN	UAE	F	NM_000038.6(APC):c.3920T>A	Breast ca 53y
288	CGS2914-01-001.NHW	Chinese	F	NM_000059.3(BRCA2):c.9253dup	Breast ca 30y (R in situ breast carcinoma, ER/PR/HER2 unknown)<br>Breast ca 31y (L in situ breast carcinoma ER/PR+)<br>Breast ca 57y (R breast ca, new primary)<br>Neurofibroma/Schwanoma
289	CGS2362-01-001.SSB	Malay	F	NM_007294.4(BRCA1):c.2269del	Breast ca 33y
290	CGS2927-01-001.LPT	Chinese	F	NM_000051.4(ATM): c.(?_?)del (exons 62_63del)	Breast ca 44y, Left pelvic adenocarcinoma 62y
291	CGS2970-01-001.FRS	UAE	F	NM_000059.3(BRCA2):c.5290_5291del	Ovarian ca 58y
292	CGS3004-01-001.LWS	Chinese	M	NM_000267.3(NF1):c.3826C>T	Pheochromocytoma 43y
293	CGS3013-01-001.CSY	Chinese	F	NM_000314.4(PTEN):c.640C>T	Breast ca 37y
294	CGS3124-01-001.YYJ	Chinese	M	NM_001379610.1(SPINK1):c.194+2T>C	Glioma 14y
295	CGS3132-01-001.LGN	Chinese	F	NM_007294.4(BRCA1):c.4583_4589dup	Breast ca 55y, Ovarian ca 55y
296	CGS2197-01-003.WSN	Chinese	F	NM_000179.3(MSH6):c.3886_3917dup	Endometrial ca 53y
297	CGS2197-01-002.WSG	Chinese	F	NM_000179.3(MSH6):c.3886_3917dup	Endometrial ca 54y; Right adrenal adenoma, Melanocytic naevus
298	CGS3152-01-001.AHY	Chinese	F	NM_000546.6(TP53):c.733G>A	Breast ca 30y, Sarcoma age unknown
299	CGS3156-01-001.ABA	Malay	F	NM_000059.3(BRCA2):c.682-1G>C	Breast ca 30y
300	CGS3097-03-001.HV	Indian	F	NM_000546.6(TP53):c.818G>A	Anaplastic Medulloblastoma 4y
301	CGS3097-01-001.VY	Indian	F	NM_000546.6(TP53):c.818G>A	Ovarian ca 32y; Hypothyroidism, PCOS
302	CGS3192-01-001.DCC	Chinese	M	NM_000059.3(BRCA2):c.6635_6637delins34	Pancreatic ca 50y
303	CGS3195-01-001.NQY	Chinese	F	NM_032043.2(BRIP1):c.751C>T	Breast ca 37y
304	CGS1751-01-001.CSH	Chinese	M	NM_000059.3(BRCA2):c.3883C>T	Breast ca 69y
305	CGS1692-01-001.LSM	Chinese	F	NM_000059.3(BRCA2):c.8208_8209insAG	Ovarian ca 48y
306	CGS3209-01-001.MSL	Chinese	F	NM_058216.2(RAD51C):c.905-2A>C	Ovarian ca 53y
307	CGS3163-01-001.SOL	Chinese	F	NM_000492.3(CFTR):c.1210-34TG[12]T[5]	Pancreatic ca 70y; Gallbladder adenomyoma, Chronic pancreatitis

308	CGS3210-01-001.JDS	Malay	F	NM_033084.3(FANCD2):c.4140T>A	Pancreatic ca 44y; Blocked fallopian tubes, fertility issues
309	CGS3149-01-001.SAB	Indonesian	F	NM_000059.3(BRCA2):c.262_263del	Breast ca 33y; Glaucoma
310	CGS3215-01-001.CWH	Chinese	M	NM_144997.5(FLCN):c.1433-2A>C	No cancer 50y<br>Multiple cystic lung disease<br>Right secondary spontaneous pneumothorax<br>Calculous Cholecystitis<br>Multiple skin tags, one lipoma
311	CGS3229-01-001.KKW	Chinese	F	NM_007294.4(BRCA1):c.53T>G	Ovarian ca 36y
312	CGS3232-01-001.HMN	Chinese	F	NM_024675.3(PALB2):c.1059del	Breast ca 39y (L in situ breast carcinoma ER+)<br>Breast ca 41y (R in situ breast carcinoma ER/PR+)<br>Multiple uterine fibroids and ovarian cysts
313	CGS2981-01-001.CPF	Chinese	F	NM_007294.4(BRCA1):c.3858_3861delTGAG	Breast ca 34y (L invassive breast ductal carcinoma ER+, PR-, HER2 equi)<br>Breast ca 37y (R invasive breast ductal carcinoma TNBC)
314	CGS3049-01-001.NYC	Chinese	F	NM_000179.3(MSH6):c.642del	Ovarian ca 50y
315	CGS3148-01-001.MBS	Boyanese	F	NM_007294.4(BRCA1):c.2726dup	Breast ca 46y; Abnormal Uterine Bleeding
316	CGS3035-01-001.CPH	Chinese	M	NM_000251.2(MSH2):c.1076+1G>A	Colorectal ca 50y, High grade urothelial carcinoma 58y
317	CGS3258-01-001.PVM	Chinese	F	NM_000059.3(BRCA2):c.7471C>T	Breast ca 46y; Chronic Kidney Disease<br>Multinodular Goiter with retrosternal extension<br>Subclinical hyperthyroidism<br>Presystolic murmur possibly mitral regurgitation
318	CGS3120-01-001.SCH	Chinese	M	NM_000059.3(BRCA2):c.8400_8402delinsAAAA	Prostate ca 66y
319	CGS3136-01-001.FSW	Chinese	F	NM_007194.3(CHEK2):c.247C>T	Breast ca 47y; Pernicious anemia

320	CGS3136-01-003.FHK	Chinese	M	NM_007194.3(CHEK2):c.247C>T	No cancer 49y<br>Total thyroidectomy for goitre retrosternal extension 2015<br>Benign lump under armpit<br>Infertility
321	CGS3196-01-001.WNB	Malay	F	NM_033084.3(FANCD2):c.4140T>A	Breast ca 53y; Endometrial Hyperplasia
322	CGS3309-01-001.CSF	Chinese	F	NM_007294.4(BRCA1):c.2143_2155delins TCTTT	Ovarian ca 57y
323	CGS2991-01-001.ELH	Chinese	F	NM_001128425.1(MUTYH):c.1435G>T	Breast ca 35y
324	CGS3328-01-001.CTL	Chinese	M	NM_000051.4(ATM):c.3145_3146del	Gastric ca 63y, Prostate ca 62y, Leukaemia 63y
325	CGS1370-01-001.VDG	Indian	F	NM_058216.2(RAD51C):c.394dup	Breast ca 49y, Ovarian ca 49y
326	CGS3341-01-001.WJ	Chinese	F	NM_058216.2(RAD51C):c.905-2A>C	Breast ca 43y; Benign breast lumps, Anemia, Rectosigmoid polyps
327	CGS3344-01-001.CTS	Chinese	F	NM_005732.3(RAD50):c.2786dup	Breast ca 56y
328	CGS3214-01-001.HBA	Malay	F	NM_005591.3(MRE11):c.1500+2del	Ovarian ca 53y, Endometrial ca 52y
329	CGS2075-02-001.NHB	Malay	M	NM_000267.3(NF1): c.(?_?)del (exon 50)	No cancer 45y; Skin neurofibroma
330	CGS3070-01-001.JDK	Indian	F	NM_000059.3(BRCA2):c.5722_5723del	Ovarian ca 63y
331	CGS3350-01-001.SNS	Malay	F	NM_007294.4(BRCA1):c.2726dup	Ovarian ca 45y
332	CGS3383-01-001.LGH	Chinese	F	NM_000059.3(BRCA2):c.4151del	Breast ca 57y (R invasive breast ductal carcinoma ER/PR+ and HER2 equi)<br>Breast ca 60y (L invasive breast ductal carcinoma PR/HER2+ , ER-)
333	CGS3370-01-001.KNH	Chinese	F	NM_000368.5(TSC1):c.1525C>T	Gastric ca 54y; multiple skin lesions around neck & shoulder area
334	CGS3371-01-001.LKY	Chinese	M	NM_015450.2(POT1): c.(?_?)del (entire coding sequence)	Left proximal tibial giant cell tumour (GCT) 23y<br>Lymphoma 69y<br>Hepatocellular carcinoma 72y<br>Leukaemia 73y
335	CGS3368-01-001.BJC	Filipino	F	NM_000059.3(BRCA2):c.6644_6647del	Ovarian ca 54y; small fibroids, Previous perimenopausal DUB
336	CGS3320-01-001.MRB	Malay	M	NM_001379610.1(SPINK1):c.101A>G	Pancreatic ca 44y

337	CGS3381-01-001.PJ	Chinese	F	NM_007194.3(CHEK2):c.433C>T	Breast ca 37y
338	CGS3389-01-001.LLH	Chinese	F	NM_000059.3(BRCA2):c.3716_3717del	Ovarian ca 56y;<br>R Breast nodule: Benign fibroepithelial polyp
339	CGS3395-01-001.KAC	Chinese	F	NM_000249.3(MLH1):c.2042C>T	Ovarian ca 42y
340	CGS3233-01-001.MDS	Indian	F	NM_007294.4(BRCA1):c.5365_5366delins A	Breast ca 33y (L invasive breast ductal carcinoma PR+)<br>Breast ca 36y (R invasive breast ductal carcinoma TNBC)<br>Ovarian ca 46y<br>Acute hepatitis, Latent tuberculosis, Haemorrhoids
341	CGS3431-01-001.RBP	Malay	M	NM_001379610.1(SPINK1):c.101A>G	Pancreatic ca 55y
342	CGS3446-01-001.LWP	Chinese	F	NM_000546.6(TP53):c.844C>T	Breast ca 45y
343	CGS2084-01-001.WSH	Chinese	M	NM_000059.3(BRCA2):c.3847_3848del	Prostate ca 61y
344	CGS3460-01-001.TPY	Chinese	F	NM_032043.2(BRIP1):c.1343G>A	Ovarian ca 65y
345	CGS3356-01-001.NBS	Malay	F	NM_000051.4(ATM):c.9070_*53del	Primary peritoneal cancer 58y
346	CGS3099-01-001.NH	Malay	F	NM_002485.4(NBN):c.2070+1G>A	Breast ca 36y; Left Anterior Uveitis
347	CGS3494-01-001.IRS	Filipino	F	NM_000059.3(BRCA2):c.3362C>G	Breast ca 36y
348	CGS3505-01-001.HG	Unknown	F	NM_000546.6(TP53):c.809T>C	Breast ca 20y, Gastric ca 24y, Lymphoma 27y
349	CGS3513-01-001.TST	Chinese	F	NM_007294.4(BRCA1):c.5074+3A>G	Ovarian ca 45y
350	CGS3524-01-001.TFW	Chinese	F	NM_024675.3(PALB2):c.2968G>T	Breast ca 50y (R invasive breast ductal carcinoma ER/PR+, HER2-)<br>Breast ca 59y (L invasive breast ductal carcinoma ER/PR+)
351	CGS3088-01-003.NLC	Chinese	F	NM_000059.3(BRCA2):c.7414_7415del	Breast ca 34y (laterality unknown, Infiltrating Ductal Carcinoma, ER/PR+)<br>Breast ca 43y (L invasive breast ductal carcinoma TNBC)
352	CGS3549-01-001.WFF	Chinese	M	NM_000059.3(BRCA2):c.4440T>G	Prostate ca 69y
353	CGS3553-01-001.PSF	Chinese	M	NM_000264.3(PTCH1):c.3530del	Multiple BCC 65y;<br>Ischemic heart disease (IHD), Hypothyroidism, Chronic inflammatory

354	CGS3556-01-001.CCP	Chinese	F	NM_001204.7(BMPR2): c.(?_?)del (exons 2_5del)	No cancer 46y; Pulmonary arterial hypertension, Heart failure
355	CGS3052-01-001.BDK	Indian	F	NM_007294.4(BRCA1):c.68_69del	Breast ca 46y
356	CGS3495-01-001.STS	Indian	F	NM_000546.6(TP53):c.659A>G	Sarcoma 29y<br>Colorectal ca 46y<br>Uterine fibroids w/ endometrial thickening<br>left parathyroid adenoma
357	CGS3572-01-001.LBL	Chinese	M	NM_000179.3(MSH6):c.3746_3749dup	Colorectal ca 68y; Hearing issues from age 60
358	CGS3529-01-001.LGT	Chinese	F	NM_000059.3(BRCA2):c.9097del	Breast ca 47y, Ovarian ca 53y, Lung ca 57y; Hypothyroid
359	CGS3544-01-001.WSY	Chinese	F	NM_000059.3(BRCA2):c.2808_2811del	Ovarian ca 60y
360	CGS3644-01-001.KSF	Vietnamese	F	NM_000249.3(MLH1):c.790+1G>A	Colorectal ca 37y Ovarian cyst
361	CGS3651-01-001.WX	Chinese	F	NM_001379610.1(SPINK1):c.194+2T>C	Pancreatic ca 40y
362	CGS3558-01-001.WAC	Chinese	M	NM_000251.2(MSH2): c.(?_?)del (exons 1_3del)	Colorectal ca 79y, Prostate ca 81y, Adenocarcinoma of the duodenum age unknown
363	CGS2383-01-001.LYQ	Chinese	F	NM_000314.4(PTEN):c.493G>A	Breast ca 27y
364	CGS0875-02-001.BTN	Indian	M	NM_000059.3(BRCA2):c.9380G>A	Prostate ca 68y
365	CGS3681-01-001.SSJ	Indian	F	NM_007294.4(BRCA1):c.2719_2722del	Ovarian ca 69y
366	CGS3683-01-001.CSP	Chinese	F	NM_007294.4(BRCA1):c.3661G>T	Ovarian ca 67y
367	CGS3693-01-001.ELS	Chinese	F	NM_001128425.1(MUTYH):c.55C>T	Breast ca 29y,<br>Recurrent malignant phyllodes tumour in the abdomen 30y;<br>GERD
368	CGS3696-01-001.CCT	Chinese	F	NM_005591.3(MRE11):c.1888C>T	Breast ca 30y
369	CGS3698-01-001.AT	Japanese	F	NM_000251.2(MSH2):c.1216C>T	Colorectal ca 39y
370	CGS3552-03-002.TCW	Chinese	F	NM_007294.4(BRCA1):c.3331C>T	No cancer 29y; R breast fibroadenoma, L breast benign lumps
371	CGS3707-01-001.SKH	Chinese	F	NM_000465.3(BARD1):c.2208T>A	Colorectal ca 44y
372	CGS3731-01-001.MJ	Chinese	M	NM_000551.3(VHL):c.242C>A	pTa low grade papillary urothelial cancer 51y<br>Prostate ca 52y<br>Gastric leiomyoma
373	CGS2005-01-001.SEF	Chinese	F	NM_005236.2(ERCC4):c.2169C>A	Breast ca 35y

374	CGS3759-01-001.TTK	Chinese	F	NM_032043.2(BRIP1):c.1343G>A	Ovarian ca 75y
375	CGS3753-02-001.ARP	Indian	F	NM_007294.4(BRCA1):c.68_69del	Breast ca 36y
376	CGS3767-01-001.OPL	Chinese	F	NM_000059.3(BRCA2):c.7895dup	Breast ca 46y, Metachronous high grade urothelial cancer age unknown
377	CGS3767-01-002.OPK	Chinese	F	NM_000059.3(BRCA2):c.7895dup	Breast ca 61y
378	CGS2943-01-001.TQQ	Chinese	F	NM_144997.5(FLCN):c.1432+1G>C	No cancer 21y; Pulmonary cysts -
379	CGS3803-01-001.KLS	Chinese	F	NM_000059.3(BRCA2):c.6486_6489del	Breast ca 47y, Thyroid ca 48y
380	CGS3802-01-001.NLF	Chinese	F	NM_000321.3(RB1):c.1494T>G	Bilateral Retinoblastoma 3y; Sarcoma 43y; Appendectomy, Left hemi thyroidectomy for hypothyroidism
381	CGS3840-01-001.YQL	Chinese	F	NM_000267.3(NF1):c.6791dup	Breast ca 55y
382	CGS3843-01-001.LKG	Chinese	F	NM_007294.4(BRCA1): c.(?_?)del (exons 1_2del)	Breast ca 42y; Graves's disease, Hyperthyroidism
383	CGS3819-01-001.OG	Chinese	F	NM_007294.4(BRCA1): c.(?_?)del (exons 16_19del)	Breast ca 26y
384	CGS3858-01-001.FBM	Malay	F	NM_000038.6(APC):c.3183_3187del	Colorectal ca 30y, Desmoid tumour (Mesenteric) 35y
385	CGS3862-01-001.KBT	Chinese	F	NM_000251.2(MSH2):c.1165del	Colorectal ca 36y (ascending right)<br>Colon ca 36y<br>Endometrial ca 44y
386	CGS3862-01-004.XJ	Chinese	M	NM_000251.2(MSH2):c.1165del	No cancer 34y; Polyps
387	CGS3862-02-001.KCH	Chinese	M	NM_000251.2(MSH2):c.1165del	Colorectal ca 62y
388	CGS3815-01-001.MFB	Indian	M	NM_000249.3(MLH1):c.306G>A	Colorectal ca 37y
389	CGS3861-01-001.WY	Chinese	F	NM_024675.3(PALB2):c.1408del	Breast ca 41y
390	CGS3737-01-001.TBH	Chinese	F	NM_000179.3(MSH6):c.3873_3888dup	Colorectal ca 60y
391	CGS3789-01-001.CKC	Chinese	M	NM_000179.3(MSH6):c.3261del	Colorectal ca 39y
392	CGS3902-01-001.NJG	Chinese	F	NM_007294.4(BRCA1):c.66dup	Breast ca 38y, Pancreatic ca 44y, Lymphoma 62y
393	CGS3901-01-001.FSH	Chinese	F	NM_000059.3(BRCA2):c.6486_6489del	Breast ca 36y
394	CGS3908-01-001.HZ	Chinese	F	NM_002878.3(RAD51D):c.270_271dup	Ovarian ca 59y
395	CGS3914-01-001.LKL	Chinese	F	NM_000038.6(APC):c.847C>T	No cancer 43y; pan colonic and rectal polyps-hundreds
396	CGS3916-01-001.RSE	Filipino	F	NM_007294.4(BRCA1):c.5335del	Breast ca 33y

397	CGS3917-01-001.PR	Chinese	F	NM_004260.4(RECQL4):c.1258+1G>A	Breast ca 36y; Colon polyp - Tubular adenoma with low grade dysplasia
398	CGS3612-01-002.LFH	Chinese	M	NM_000059.3(BRCA2):c.2808_2811del	No cancer 69y; Prostate enlargement, Lump on his left armpit
399	CGS3641-01-002.YHM	Chinese	M	NM_024675.3(PALB2):c.3426_3429del	No cancer 64y; Prostate enlargement
400	CGS3641-03-001.YX	Chinese	F	NM_024675.3(PALB2):c.3426_3429del	No cancer 33y; Breast cyst
401	CGS3950-01-001.SDV	Indian	F	NM_000127.3(EXT1):c.1659C>G	Sarcoma 32y TB
402	CGS3951-01-001.NSC	Chinese	F	NM_144997.5(FLCN):c.1301_1302del	No cancer 47y; Congenital cystic disease
403	CGS3955-01-001.YSL	Chinese	F	NM_000546.6(TP53):c.473G>A	Breast ca 51y,<br>Multifocal urothelial cancer 53y<br>Lung adenocarcinoma 53y<br>Bladder cancer 53y
404	CGS3968-01-001.TLC	Chinese	F	NM_000059.3(BRCA2):c.2808_2811del	Breast ca 46y
405	CGS3899-01-001.ZBM	Malay	F	NM_000051.4(ATM):c.8110dup	Ovarian ca 44y; Fibroids
406	CGS3362-01-002.NKS	Chinese	F	NM_000059.3(BRCA2):c.5164_5165del	Breast ca 61y; Hyperthydoisim (total thyroidectomy)
407	CGS3989-01-001.NS	Chinese	F	NM_000059.3(BRCA2):c.3847_3848del	No cancer 33y; Breast fibroadenoma
408	CGS4007-01-001.NGZ	Chinese	M	NM_000321.3(RB1):c.1666C>T	Left eye retinoblastoma 3y
409	CGS4010-01-001.KLC	Chinese	F	NM_007294.4(BRCA1): c.(?_?)del (exons 1_2del)	Breast ca 42y (left invasive ductal breast carcinoma, ER/PR/HER2 status unknown)<br>Breast ca 49y (R invasive ductal breast carcinoma PR/HER2+)<br>Endometrial ca 65y
410	CGS4025-01-001.JLG	Chinese	F	NM_000059.3(BRCA2):c.6129del	Breast ca 52y
411	CGS4057-01-001.ASK	Chinese	F	NM_001943.5(DSG2):c.3039C>A	No cancer 53y; Aplastic anaemia (Non severe), Graves disease, Breast cyst
412	CGS4082-01-001.TCT	Chinese	F	NM_007294.4(BRCA1): c.(?_?)dup (exon 12dup)	Ovarian ca 54y
413	CGS4026-01-001.PFG	Chinese	M	NM_000546.6(TP53):c.740A>T	Sarcoma 28y
414	CGS4111-01-001.YWH	Chinese	M	NM_000251.2(MSH2):c.3226C>T	Kidney ca 27y; Kidney stones
415	CGS4133-01-001.OLL	Chinese	F	NM_000546.6(TP53):c.422G>A	Adrenal Cortical Carcinoma 14y

416	CGS4140-01-001.AL	Chinese	F	NM_000267.3(NF1):c.2540T>C	No cancer 22y;<br>Left CN X (vagus nerve) neurogenic tumour,<br>Right thigh plexiform neurofibroma,<br>Longstanding migraine
417	CGS4019-01-001.SSK	Chinese	M	NM_007294.4(BRCA1):c.3607C>T	Kidney ca 68y; Allergic rhinitis, Diabetes mellitus, Rheumatoid arthritis
418	CGS3844-01-001.FBM	Chinese	F	NM_007294.4(BRCA1):c.5075-2A>G	Breast ca 37y, Ovarian ca 60y
419	CGS4161-01-001.TYM	Chinese	F	NM_000059.3(BRCA2):c.7617+1G>A	Breast ca 39y
420	CGS4168-01-001.LMM	Chinese	F	NM_000051.4(ATM):c.875C>T	Endometrial ca 50y
421	CGS4181-01-001.MSM	Chinese	F	NM_024675.3(PALB2):c.7G>T	Breast ca 37y
422	CGS4053-01-001.LCF	Chinese	F	NM_000179.3(MSH6):c.3108_3109del	Breast ca 43y
423	CGS4187-01-001.AM	Indian	M	NM_000553.4(WRN):c.2886del	Colorectal ca 55y; LUL lung acinar adenocarcinoma 49y LUNG, VARIOUS LESIONS
424	CGS4174-01-001.ERA	Filipino	F	NM_000535.4(PMS2):c.2500_2501delins G	Breast ca 42y; Hypothyroidism, Gestational DM, Laparoscopic cholecystectomy
425	CGS4208-01-001.PPE	Chinese	F	NM_000249.3(MLH1):c.121G>A	Colorectal ca 62y; Hyperthyroid, Nodular goiter
426	CGS4217-01-001.YSH	Chinese	M	NM_000051.4(ATM):c.1402_1403del	Hyams Grade 1 Olfactory neuroblastoma y
427	CGS4223-01-001.SWK	Chinese	F	NM_000059.3(BRCA2):c.8585_8586del	Breast ca 49y
428	CGS4220-01-001.LLN	Chinese	F	NM_007294.4(BRCA1):c.4372C>T	Ovarian ca 56y
429	CGS4253-01-001.KLH	Chinese	F	NM_000059.3(BRCA2):c.5164_5165del	Breast ca 44y, Thyroid ca 58y; Osteoma, nos 1 - Right Benign neoplasm bones skull & face
430	CGS4264-01-001.AG	Indian	M	NM_000251.2(MSH2):c.1786_1788del	Colorectal ca 44y
431	CGS2264-01-001.LAH	Chinese	F	NM_007294.4(BRCA1):c.3858_3861del	Breast ca 40y (left iIDC, ER/PR-, HER2 unknown)<br>Breast ca 51y (R IDC ER/PR/HER2 unknown)
432	CGS4268-01-001.RMD	Indian	F	NM_000492.3(CFTR):c.1210-34TG[13]T[5]	Leukaemia 58y, Sarcoma 58y
433	CGS4267-01-001.YKT	Chinese	F	NM_002878.3(RAD51D):c.480+1G>A	Breast ca 43y
434	CGS4271-01-001.AHH	Chinese	F	NM_007194.3(CHEK2):c.247del	Sarcoma 51y
435	CGS4173-01-001.FTW	Chinese	F	NM_007294.4(BRCA1):c.5353C>T	Ovarian ca 62y

436	CGS4274-01-001.AHM	Chinese	F	NM_004655.3(AXIN2):c.1614_1642del	No cancer 39y; Minor coronary artery disease, Dyslipidemia
437	CGS4283-01-001.KB	Indian	F	NM_000059.3(BRCA2):c.4003G>T	Breast ca 51y
438	CGS4290-01-001.LJE	Filipino	M	NM_000249.3(MLH1):c.794G>C	Colorectal ca 46y; Neck lipoma
439	CGS4325-01-001.NBA	Malay	F	NM_000179.3(MSH6):c.3341_3342insC	Endometrial ca 50y; Osteoarthritis
440	CGS4334-01-001.OPL	Chinese	M	NM_006767.3(LZTR1):c.1018C>T	Prostate ca 66y; Recurrent balanitis
441	CGS4345-01-001.KSM	Chinese	F	NM_007294.4(BRCA1): c.(?_?)del (exons 1_2del)	Breast ca 43y (R, histology unknown, ER/PR/HER2 unknown)<br>Breast ca 57y (L invasive breast ductal carcinoma ER/PR/HER2 negative)
442	CGS4100-01-001.ABA	Malay	M	NM_000059.3(BRCA2):c.3109C>T	Prostate ca 62y
443	CGS4395-01-001.YJN	Chinese	F	NM_000546.6(TP53):c.438G>A	Breast ca 25y, Sarcoma 12y
444	CGS0077-01-001.CLS	Chinese	F	NM_000059.3(BRCA2):c.3002C>G	Ovarian ca 64y
445	CGS0702-01-001.OLE	Chinese	F	NM_007294.4(BRCA1): c.(?_?)del (exons 16_19del)	Breast ca 42y (R invasive breast ductal carcinoma PR+)<br>Breast ca 47y (L in situ breast ductal carcinoma ER/PR-, HER2 unknown)
446	CGS0006-01-001.WKK	Chinese	F	NM_007294.4(BRCA1):c.(?_?)dup (exons 4_6dup)	Ovarian ca 61y
447	CGS4078-01-001.LGH	Chinese	M	NM_000051.4(ATM):c.1402_1403del	Prostate ca 62y
448	CGS3113-01-001.GTA	Chinese	F	NM_000059.3(BRCA2):c.3167_3170del	Primary Peritoneal Cancer 63y
449	CGS4034-01-001.WYO	Chinese	F	NM_058216.2(RAD51C):c.394dup	Ovarian ca 60y
450	CGS0003-01-001.PLW	Chinese	F	NM_007294.4(BRCA1):c.3331C>T	Ovarian ca 47y
451	CGS0209-02-001.CM	Chinese	F	NM_000059.3(BRCA2):c.9097dupA	Breast ca 62y
452	CGS3331-01-001.RBH	Malay	F	NM_000251.2(MSH2):c.2131C>T	Endometrial ca 52y; Left LL DVT + B/L PE, Left LL sciatica
453	CGS2335-01-001.RS	Indian	F	untraceable	Breast ca 21y (left invasive breast ductal carcinoma ER+)<br>Breast ca 39y (R invasive breast ductal carcinoma TNBC)
454	CGS0002-01-001.MBN	Malay	F	NM_007294.4(BRCA1):c.2726dupA	Breast ca 39y
455	CGS2972-01-001.LFK	Chinese	F	NM_003001.3(SDHC):c.214C>T	Breast ca 42y
456	CGS0730-01-001.ABA	Malay	F	NM_007294.4(BRCA1):c.5152+1G>A	Breast ca 30y

457	CGS3086-01-001.CT	Chinese	F	NM_000251.2(MSH2):c.1394dup	Pancreatic ca 41y
458	CGS3687-01-001.TSP	Chinese	F	NM_000051.4(ATM):c.7515del	Breast ca 31y
459	CGS0008-01-001.GLW	Chinese	F	NM_007294.4(BRCA1):c.4709dupT	Breast ca 42y, Ovarian ca 56y
460	CGS4355-01-001.SS	Indian	F	NM_000059.3(BRCA2):c.3823dup	Ovarian ca 70y; Mild AS EF 60-65%, nil RWMA
461	CGS2095-01-001.OAN	Chinese	F	NM_000059.3(BRCA2):c.7379_7382del	Ovarian ca 70y
462	CGS0018-01-002.HCL	Chinese	F	NM_000059.3(BRCA2):c.631+2T>A	Breast ca 54y
463	CGS3761-01-001.CHH	Chinese	M	NM_001184.4(ATR):c.6836dup	Breast ca 59y
464	CGS4260-01-001.GHG	Chinese	F	NM_000059.3(BRCA2):c.4381del	Breast ca 41y; Osteoporosis, Left wrist ganglion
465	CGS2947-01-001.RTC	Chinese	F	NM_000251.2(MSH2):c.1662-2A>G	Endometrial ca 42y; Congenital rubella
466	CGS1338-01-001.NBB	Malay	F	NM_000059.3(BRCA2):c.8191delC	Breast ca 36y
467	CGS0612-01-001.YZ	Chinese	F	NM_000059.3(BRCA2):c.9772_9775delGAGA	Breast ca 31y
468	CGS2886-01-001.TSJ	Chinese	F	NM_007294.4(BRCA1):c.5072C>A	Breast ca 29y
469	CGS4109-01-001.ASB	Indian	M	NM_000251.2(MSH2):c.1042C>T	Colorectal ca 29y
470	CGS2391-01-001.LJX	Chinese	F	NM_000551.3(VHL):c.499C>T	Pheochromocytoma 17y; Retinal hemangioma
471	CGS3913-02-001.TSN	Chinese	F	NM_007294.4(BRCA1):c.442-22_442-13del	Breast ca 60y, Ovarian ca 78y
472	CGS2451-01-001.CTY	Chinese	F	NM_005732.3(RAD50):c.2786dup	Ovarian ca 71y
473	CGS4340-01-001.AS	Chinese	F	NM_032043.2(BRIP1): c.(?_?)del (exon 8)	Ovarian ca 88y stage 3A<br>CKD, H pylori + gastritis, rectal tubulovillous adenoma with LGD
474	CGS0652-01-001.WYF	Chinese	F	NM_007294.4(BRCA1):c.3G>T	Breast ca 42y, Ovarian ca 53y
475	CGS1969-01-001.CTL	Chinese	F	NM_024675.3(PALB2):c.2607del	Breast ca 51y (L invasive breast ductal carcinoma ER/PR-, HER2 equi)<br>Breast ca 54y (Right in situ breast carcinoma ER/PR+)
476	CGS3157-01-001.MBA	Malay	F	NM_007194.3(CHEK2):c.433C>T	Breast ca 53y

477	CGS2303-01-001.ETC	Chinese	F	untraceable	Breast ca 38y (L invasive breast ductal carcinoma, ER/PR-, HER2 equi)<br>Breast ca 45y (R invasive breast ductal carcinoma TNBC)
478	CGS4278-01-001.COL	Chinese	F	NM_007294.4(BRCA1): c.(?_?)del (exons 1_2del)	No cancer 54y; Mild CKD, Gout, Leukocytosis
479	CGS3951-01-002.NSJ	Chinese	F	NM_144997.5(FLCN):c.1301_1302del	No cancer 52y; Quick heart beat
480	CGS0007-01-001.KYN	Chinese	F	NM_007294.4(BRCA1):c.2707delT	Breast ca 36y (left invasive breast ductal carcinoma ER+, PR-, HER2 equi)<br>Breast ca 37y (R in situ breast carcinoma ER/PR/HER2 status unknown)
481	CGS3144-01-001.SSB	Indian	F	NM_000314.4(PTEN):c.388C>T	Endometrial ca 37y
482	CGS2977-01-001.LLX	Chinese	F	NM_024675.3(PALB2):c.1240C>T	Breast ca 37y
483	CGS4070-01-001.RFM	Indian	F	NM_007294.4(BRCA1):c.68_69del	Breast ca 37y
484	CGS1933-01-001.MBM	Malay	F	NM_007294.4(BRCA1):c.2726dup	Breast ca 30y
485	CGS1917-01-001.ESW	Chinese	M	NM_000267.3(NF1):c.801G>A	NF1 (clinically diagnosed) 23y
486	CGS2447-01-001.LPH	Chinese	F	NM_000492.3(CFTR):c.1210-34TG[12]T[5]	Breast ca 66y
487	CGS3962-01-001.MNB	Indian	M	NM_000546.6(TP53): c.(?_?)del (promoter)	Prostate ca 71y, Squamous Cell Carcinoma 59y, Sarcoma 70y
488	CGS0698-01-001.LSC	Chinese	F	NM_007294.4(BRCA1):c.5072C>A	Breast ca 54y (left invasive breast ductal carcinoma TNBC)<br>Breast ca 59y (R invasive breast ductal carcinoma TNBC)
489	CGS2090-01-001.SAC	Chinese	F	NM_002878.3(RAD51D):c.270_271dup	Breast ca 36y
490	CGS3076-01-001.LGE	Chinese	F	NM_005732.3(RAD50):c.2165dup	Colorectal ca 41y, Endometrial ca 44y
491	CGS0718-01-001.LHB	Chinese	F	NM_000551.3(VHL):c.191G>C	Pheochromocytoma 35y; Right capillary haemangioma
492	CGS4351-01-001.WH	Chinese	F	NM_003977.3(AIP): c.(?_?)del (exons 1_2del)	Breast ca 36y
493	CGS4039-01-001.LSW	Chinese	F	NM_000553.4(WRN):c.502_503del	Breast ca 35y
494	CGS1716-01-001.FWF	Chinese	F	NM_000059.3(BRCA2):c.5809_5812del	Ovarian ca 65y
495	CGS2156-01-001.SRB	Malay	F	NM_000059.3(BRCA2):c.9027T>G	Breast ca 67y, Ovarian ca 67y

496	CGS3552-01-001.LSE	Chinese	F	NM_007294.4(BRCA1):c.3331C>T	Ovarian ca 72y
497	CGS1729-01-001.TCS	Chinese	F	NM_007294.4(BRCA1):c.3770_3771del	Breast ca 60y
498	CGS2957-01-001.LCH	Chinese	F	NM_032043.2(BRIP1):c.1510dup	Ovarian ca 54y
499	CGS2237-01-001.KDG	Indian	F	NM_024675.3(PALB2):c.2716del	Breast ca 61y
500	CGS1592-01-001.TBC	Chinese	F	NM_032043.2(BRIP1): c.(?_?)del (exons 5_6del)	Ovarian ca 51y
501	CGS2284-01-001.TLS	Chinese	F	NM_000059.3(BRCA2):c.5799_5802delCCAA	Ovarian ca 51y
502	CGS4352-01-001.YLF	Chinese	F	NM_007294.4(BRCA1):c.3627dup	Breast ca 42y (right mucinous carcinoma, ER/PR+)<br>Breast ca 45y (R mucinous carcinoma ER/PR/HER2+)<br>Ovarian ca 60y
503	CGS3998-01-001.KLN	Chinese	F	NM_000057.4(BLM):c.2160_2163dup	Breast ca 36y
504	CGS0004-01-001.JFS	Chinese	F	NM_000059.3(BRCA2):c.3847_3848delGT	Breast ca 49y
505	CGS1790-01-001.TSG	Chinese	F	NM_000051.4(ATM):c.7886_7890del	Breast ca 43y (left invasive breast ductal carcinoma ER/PR+)<br>Breast ca 51y (R invasive breast ductal carcinoma ER/PR+ HER2 equi)
506	CGS0413-01-001.LH	Chinese	F	NM_007294.4(BRCA1):c.141C>A	Breast ca 50y
507	CGS0018-01-001.HCH	Chinese	F	NM_000059.3(BRCA2):c.631+2T>A	Breast ca 45y
508	CGS3811-01-001.PIJ	Filipino	F	NM_000059.3(BRCA2):c.1103C>A	No cancer 48y; Benign breast cyst
509	CGS2356-01-001.RSC	Filipino	F	NM_000059.3(BRCA2):c.6491delA	Breast ca 35y
510	CGS2449-01-001.LNC	Chinese	F	NM_024675.3(PALB2):c.2257C>T	Breast ca 42y
511	CGS3538-01-001.OSY	Chinese	F	NM_007194.3(CHEK2):c.1116dup	Breast ca 36y
512	CGS3852-01-001.NLW	Chinese	F	NM_007194.3(CHEK2):c.1459C>T	Breast ca 25y; Scoliosis, Hyperthyroidism, Tinnitus
513	CGS4246-01-001.LGC	Chinese	F	NM_000179.3(MSH6):c.3827_3830dup	Endometrial ca 69y; Colonic polyp, haemorroids
514	CGS2433-01-001.STK	Chinese	F	NM_058216.2(RAD51C):c.675del	Breast ca 56y, Ovarian ca 45y
515	CGS0957-01-001.LLK	Chinese	F	NM_007294.4(BRCA1):c.5332+1G>A	Breast ca 57y, Ovarian ca 63y
516	CGS0778-01-001.MDR	Indian	F	NM_000059.3(BRCA2):c.6468_6469dupTC	Ovarian ca 60y

517	CGS0771-01-001.CSK	Chinese	F	NM_000059.3(BRCA2):c.8188G>C	Breast ca 69y
518	CGS4033-01-001.AKS	Chinese	M	NM_000059.3(BRCA2):c.5073dup	Pancreatic ca 66y
519	CGS4270-01-001.LML	Chinese	F	NM_024675.3(PALB2):c.1314dup	Breast ca 55y
520	CGS3088-01-001.NLH	Chinese	F	NM_000059.3(BRCA2):c.7414_7415del	Breast ca 51y
521	CGS1279-01-001.SHE	Chinese	F	NM_007294.4(BRCA1):c.213-12A>G	Breast ca 43y
522	CGS3656-01-001.LSB	Chinese	F	NM_058216.2(RAD51C):c.394dup	Ovarian ca 48y
523	CGS2974-01-001.AIA	Malay	M	NM_000546.6(TP53):c.542G>A	Gastric ca 41y; Polycystic kidney disease
524	CGS0797-01-001.NSY	Chinese	F	NM_007294.4(BRCA1):c.5332+1G>A	Ovarian ca 34y
525	CGS2918-01-001.YSY	Chinese	F	NM_007294.4(BRCA1):c.981_982del	Breast ca 35y
526	CGS3008-01-001.NM	Burmese	F	NM_007294.4(BRCA1):c.4717del	Breast ca 39y
527	CGS0800-01-001.AS	Chinese	F	NM_000551.3(VHL):c.(?_?)del (exons 1_3del)	Kidney ca 34y;<br>L optic disc haemgioma,<br>Brain/spinal tumours (from MRI), Breast fibroadenoma L
528	CGS2967-01-001.NBN	Malay	F	NM_007294.4(BRCA1):c.2726dup	Breast ca 34y
529	CGS0802-01-001.DXL	Chinese	M	NM_000038.6(APC):c.2097G>A	Colorectal ca 30y
530	CGS3479-01-001.CMK	Chinese	F	NM_007294.4(BRCA1):c.5335del	Breast ca 34y
531	CGS3552-03-001.ZP	Chinese	F	NM_007294.4(BRCA1):c.3331C>T	No cancer 29y; PCOS
532	CGS0247-01-001.SBI	Malay	M	NM_000551.3(VHL):c.481C>T	No cancer; Left retinal angioma
533	CGS3435-01-001.SNB	Malay	F	NM_007294.4(BRCA1):c.2726dup	No cancer 22y; Breast lumps
534	CGS2056-01-001.LLT	Chinese	F	NM_007294.4(BRCA1):c.5332+1G>A	Breast ca 52y, Ovarian ca 64y
535	CGS3649-01-001.CKE	Chinese	F	NM_007294.4(BRCA1):c.981_982del	Ovarian ca 66y
536	CGS3362-01-001.NKY	Chinese	F	NM_000059.3(BRCA2):c.5164_5165del	Breast ca 57y; endometrial fibroid
537	CGS3864-01-001.OLB	Chinese	F	NM_000059.3(BRCA2):c.8915del	Ovarian ca 61y
538	CGS1346-01-001.PSC	Chinese	F	NM_000051.4(ATM):c.8435_8436delCT	Breast ca 56y
539	CGS3641-01-001.YHH	Chinese	M	NM_024675.3(PALB2):c.3426_3429del	Breast ca 57y; Castleman disease
540	CGS4234-01-001.SY	Chinese	M	NM_001379610.1(SPINK1):c.194+2T>C	Colorectal ca 57y, low grade papillary urothelial carcinoma 57y; Pancreatitis
541	CGS3161-01-001.TTL	Chinese	F	NM_002878.3(RAD51D):c.270_271dup	Breast ca 50y
542	CGS2061-01-001.CML	Chinese	F	NM_007294.4(BRCA1):c.4120_4121del	Breast ca 38y (R invasive breast ductal carcinoma TNBC)<br>Breast ca 49y (L invasive breast ductal carcinoma TNBC)

543	CGS4080-01-001.H	Indonesian	F	NM_007294.4(BRCA1): c.(?_?)del (exons 1_5del)	Breast ca 50y
544	CGS0138-01-001.LHH	Chinese	F	NM_000038.6(APC):c.1638del	Thyroid ca 19y, Desmoid tumour 36y
545	CGS1612-01-001.RBA	Indian	F	NM_000059.3(BRCA2):c.6468_6469dupTC	Breast ca 38y (R invasive breast ductal carcinoma ER/PR+ HER2 equi)<br>Breast ca 45y (L invasive lobular breast carcinoma ER/PR+, HER2-)
546	CGS3878-01-001.MMB	Indian	F	NM_000059.3(BRCA2):c.6468_6469dup	Breast ca 36y; Papilloma
547	CGS2226-01-001.LWW	Chinese	F	NM_007294.4(BRCA1):c.5072C>A	Breast ca 39y
548	CGS2382-01-001.TPY	Chinese	F	NM_000314.4(PTEN):c.641del	Breast ca 26y; Mild intellectual impairment, Macrocephaly (60cms), Haemangiomata, Thyroid nodules and prominent cervical lymph nodes
549	CGS0810-01-001.CCL	Chinese	F	NM_000059.3(BRCA2):c.8331+1G>A	Breast ca 59y, Ovarian ca 60y
550	CGS3895-01-001.KAC	Chinese	F	NM_000059.3(BRCA2):c.7133C>G	Breast ca 43y (R, histo unknown) & 65y (R invasive ductal breast carcinoma TNBC)
551	CGS3990-01-001.TCT	Chinese	M	NM_000059.3(BRCA2):c.5645C>A	Prostate ca 61y
552	CGS0226-01-001.CLT	Chinese	F	NM_000059.3(BRCA2):c.7791delA	Ovarian ca 55y
553	CGS3913-01-001.JKP	Chinese	F	NM_007294.4(BRCA1):c.442-22_442-13del	Breast ca 57y;<br>Benign ovarian cysts, Right tongue nodule, Multinodular goitre with thyroid nodule
554	CGS2468-01-001.HST	Chinese	F	NM_000059.3(BRCA2):c.5722_5723del	Pancreatic ca 53y, Basaloid neoplasm 52y
555	CGS3520-01-001.CFL	Chinese	F	NM_000059.3(BRCA2):c.2442del	Breast ca 52y
556	CGS3603-01-001.TGP	Chinese	F	NM_007294.4(BRCA1):c.4372C>T	Breast ca 50y
557	CGS0759-01-001.LLH	Chinese	F	NM_007294.4(BRCA1):c.2635G>T	Ovarian ca 44y
558	CGS4090-01-002.JC	Chinese	F	NM_000038.6(APC):c.3096del	Colorectal ca 42y, Desmoid tumour 46y
559	CGS0875-01-001.BMT	Indian	F	NM_000059.3(BRCA2):c.9380G>A	Breast ca 43y
560	CGS4051-01-001.YSL	Chinese	F	NM_000267.3(NF1):c.5426G>C	Breast ca 42y; endometriotic cysts
561	CGS2357-01-001.TSP	Chinese	F	untraceable	Breast ca 37y
562	CGS1191-01-001.WHC	Chinese	M	NM_000179.3(MSH6):c.2731C>T	Prostate ca 68y
563	CGS3592-01-001.KPP	Chinese	F	NM_000059.3(BRCA2):c.6486_6489del	Breast ca 32y (Left, histo unknown) & 61y (R invasive ductal breast carcinoma ER/PR+)
564	CGS1300-01-001.CCN	Chinese	F	NM_007294.4(BRCA1):c.3286C>T	Ovarian ca 68y

565	CGS4259-01-001.TSK	Chinese	F	NM_058216.2(RAD51C):c.394dupA	Ovarian ca 61y
566	CGS1226-01-001.LSE	Chinese	F	NM_007294.4(BRCA1):c.3214delC	Breast ca 42y, Ovarian ca 52y
567	CGS1680-01-001.TSC	Chinese	F	NM_000051.4(ATM):c.2413C>T	Ovarian ca 55y
568	CGS3552-01-003.LCH	Chinese	F	NM_007294.4(BRCA1):c.3331C>T	Breast ca 46y
569	CGS3301-01-001.TYM	Chinese	F	NM_000492.3(CFTR):c.1210-34TG[13]T[5]	Ovarian ca 53y; Breast Fibroadenomatoid hyperplasia
570	CGS3666-01-001.AJL	Chinese	F	NM_032043.2(BRIP1):c.1066C>T	Ovarian ca 70y
571	CGS2937-01-001.CL	Chinese	F	NM_000251.2(MSH2):c.528_529del	Endometrial ca 49y
572	CGS1416-01-001.NBI	Malay	F	NM_024675.3(PALB2):c.2257C>T	Breast ca 40y
573	CGS3555-01-001.KS	Malay	F	NM_007294.4(BRCA1):c.2713C>T	Breast ca 38y
574	CGS3418-01-001.PYC	Chinese	M	NM_000267.3(NF1):c.7843C>T	Neurofibromatosis type 1, Retinal detachment 21y
575	CGS3715-01-001.LPY	Chinese	F	NM_000465.3(BARD1):c.69_70delins25	Ovarian ca 25y, Breast ca 64y<br>R Horner's Syndrome (Neurilemoma, benign neoplasm skin scalp & neck)
576	CGS2252-01-001.TYN	Chinese	F	untraceable	Ovarian ca 52y, Breast ca 53y
577	CGS4002-01-001.YMH	Chinese	F	NM_007194.3(CHEK2):c.1376-1G>A	Ovarian ca 78y
578	CGS4336-01-001.YLC	Chinese	F	NM_000059.3(BRCA2):c.3847_3848del	Breast ca 69y; Uterine fibroids, Right Benign mucinous cystadenofibroma
579	CGS3767-01-004.OPL	Chinese	M	NM_000059.3(BRCA2):c.7895dup	Pancreatic ca 60y
580	CGS3122-01-001.RBH	Indian	F	NM_000179.3(MSH6):c.3827_3830dup	Colorectal ca 58y; hemifacial spasm, Chronic Periodontitis
581	CGS1958-01-001.WGL	Chinese	F	NM_007294.4(BRCA1):c.5335del	Ovarian ca 55y
582	CGS4185-01-001.KLE	Chinese	F	NM_000492.3(CFTR):c.1210-34TG[13]T[5]	Pancreatic ca 55y
583	CGS1585-01-001.TLM	Chinese	F	NM_002878.3(RAD51D):c.270_271dupTA	Breast ca 50y
584	CGS2428-01-001.TCH	Chinese	F	NM_007294.4(BRCA1):c.5072C>A	Breast ca 42y (R invasive breast ductal carcinoma, ER/PR- HER2 unknown)<br>Breast ca 62y (left invasive ductal breast carcinoma ER+, PR/HER2-)
585	CGS1695-01-001.TSH	Chinese	F	NM_007294.4(BRCA1):c.800C>G	Breast ca 44y, Ovarian ca 50y

586	CGS3978-01-001.TGY	Chinese	F	NM_000267.3(NF1):c.7996_7997del	Multiple neurofibromas over all extremities and trunk since age 20s<br>Breast ca 45y
587	CGS4207-01-001.LCK	Chinese	F	NM_005732.3(RAD50):c.2165dup	Breast ca 48y; Osteopenia, Frozen shoulder, Alopecia areata
588	CGS0952-01-001.SEL	Filipino	F	NM_000059.3(BRCA2):c.3599_3600delGT	Colorectal ca 39y
589	CGS1169-01-001.HLC	Chinese	F	NM_024675.3(PALB2): c.(?_?)del (entire coding sequence)	Breast ca 39y
590	CGS4350-01-001.KBM	Malay	F	NM_000059.3(BRCA2):c.7868A>T	Breast ca 40y
591	CGS3009-01-001.NBR	Malay	F	NM_000051.4(ATM):c.8110dup	Breast ca 37y
592	CGS4141-01-001.OCW	Chinese	M	NM_000267.3(NF1):c.396del	Headache, Multiple NF on the trunk and limbs, Freckling and cafe au lait 36y
593	CGS3078-01-001.CYM	Chinese	F	NM_000135.2(FANCA):c.2015-2A>C	Metastatic adenocarcinoma of unknown origin 34y
594	CGS2897-01-001.NWM	Chinese	M	NM_000059.3(BRCA2):c.7007+1G>C	Pancreatic ca 32y
595	CGS1542-01-001.KBK	Indian	F	NM_007294.4(BRCA1):c.1204G>T	Ovarian ca 65y, Gastric ca 65y
596	CGS1471-01-001.SCH	Chinese	F	NM_032043.2(BRIP1):c.1343G>A	Breast ca 38y
597	CGS4036-01-001.TBH	Chinese	M	NM_000051.4(ATM):c.8224_8225del	Pancreatic ca 53y
598	CGS4016-01-001.YAG	Chinese	F	NM_007294.4(BRCA1): c.(?_?)dup (exon 7dup)	Breast ca 50y, Ovarian ca 60y
599	CGS1508-01-001.HHW	Chinese	F	NM_024675.3(PALB2): c.(?_?)del (entire coding sequence)	Ovarian ca 65y
600	CGS2345-01-001.NSC	Chinese	F	untraceable	Breast ca 37y (R invasive ductal breast carcinoma TNBC)<br>Breast ca 44y (L invasive ductal breast carcinoma PR+)
601	CGS1689-01-001.GCY	Chinese	F	NM_002485.4(NBN):c.38-2A>G	Breast ca 42y; Multinodular thyroid goitre
602	CGS3234-01-001.MN	Indian	F	NM_007294.4(BRCA1): c.(?_?)del (exons 1_5del)	Breast ca 30y
603	CGS3752-01-001.SLX	Chinese	F	NM_007294.4(BRCA1):c.191G>C	Breast ca 29y
604	CGS3200-01-001.MSG	Indian	F	NM_130799.2(MEN1):c.1546dup	No cancer; Parathyroid Adenoma 23y
605	CGS0714-03-001.CNX	Chinese	F	NM_000038.6(APC):c.646C>T	No cancer 10y; Polyps

606	CGS3491-01-001.ON	Chinese	F	NM_000321.3(RB1):c.2013dup	Bilateral retinoblastoma 0y; Posterior cleft palate
607	CGS1543-01-001.NTM	Vietnamese	F	NM_000251.2(MSH2): c.(?_?)del (exon 1)	Colorectal ca 43y, Endometrial ca 50y
608	CGS2116-01-001.CMS	Filipino	F	NM_000249.3(MLH1):c.793C>T	Colorectal ca 48y, Pancreatic ca 56y
609	CGS2980-01-001.SIL	Bangladeshi	F	NM_000059.3(BRCA2):c.2612C>A	Breast ca 38y
610	CGS2985-01-001.AHS	UAE	M	NM_000251.2(MSH2):c.2634G>A	Colorectal ca 31y
611	CGS2979-01-001.RMS	UAE	F	NM_000059.3(BRCA2):c.2588dup	Ovarian ca 73y
612	CGS2402-01-001.HHH	Burmese	F	NM_007294.4(BRCA1):c.745delA	Breast ca 40y
613	CGS2201-01-001.NBK	Chinese	F	NM_007294.4(BRCA1):c.5332+1G>A	Breast ca 50y, Ovarian ca 62y
614	CGS1767-01-001.KBV	Indian	M	NM_000249.3(MLH1):c.381-2A>T	Colorectal ca 57y
615	CGS1697-01-003.KS	Indonesian	M	NM_000249.3(MLH1):c.1377dup	Colorectal ca 50y
616	CGS1697-01-002.MS	Indonesian	F	NM_000249.3(MLH1):c.1377dup	Endometrial ca 48y
617	CGS1069-01-001.JAA	Filipino	F	NM_000249.3(MLH1):c.380+1G>A	Colorectal ca 32y
618	CGS1070-01-001.DMD	Malay	M	NM_000059.3(BRCA2):c.1763_1766delATAA	Breast ca 53y (L invasive ductal breast carcinoma ER/PR/HER2+)<br>Breast ca 55y (R invasive ductal breast carcinoma ER/PR+)
619	CGS0119-01-001.YSF	Chinese	F	NM_007294.4(BRCA1):c.5213_5215del	Breast IDC ER/PR+ Her2- 48y
620	CGS0336-01-001.SCL	Chinese	F	NM_058216.2(RAD51C):c.571+5G>A	Ovarian Stage 4 papillary serous 52y
621	CGS0383-01-001.TBC	Chinese	F	NM_006231.3(POLE):c.720+1G>A	TNBC Breast CA 29y
622	CGS0532-03-002.ASB	Chinese	M	NM_000251.2(MSH2):c.1042C>T	CRC adenoCA caecum MSI high<br>Sigmoid diverticulitis 27y
623	CGS0778-01-003.MDR	Unknown	F	NM_000059.3(BRCA2):c.6468_6469dup	IC2 HGSC Ovarian CA 61y
624	CGS0867-01-002.CMK	Chinese	F	NM_024675.3(PALB2):c.2968G>T	Breast G3 TNBC 54y
625	CGS1254-01-001.PYX	Chinese	F	NM_024529.4(CDC73):c.271C>T	Uterine adenosarcoma 25y
626	CGS1555-01-001.SSH	Chinese	F	NM_006231.3(POLE):c.2T>C	L Breast IDC ER/PR+ Her2- 42y<br>BCC 57y<br>R Breast ILC ER+PR- 60y
627	CGS1555-01-002.SSH	Chinese	F	NM_006231.3(POLE):c.2T>C	3A MMMT Endometrial CA 55y
628	CGS1948-01-001.TCK	Chinese	F	NM_000051.4(ATM):c.4909+4C>T	R Breast IDCgr3, ER/PR+HER2- 54y<br>L Breast DCIS gr3
629	CGS2031-01-001.NKY	Chinese	F	NM_058216.2(RAD51C):c.571+5G>A	HGS PPC Ovarian CA 79y
630	CGS2158-02-001.KFC	Chinese	M	NM_000251.2(MSH2):c.1799C>T	CRC Loss of MSH2 and MSH6 54y

631	CGS2279-01-001.KLH	Unknown	F	NM_002878.3(RAD51D):c.270_271dup	3C multiple recurrent serous ovarian CA 56y
632	CGS2286-01-001.OBE	Chinese	F	NM_000059.3(BRCA2):c.7697dup	T1N0 (stage II) Breast CA
633	CGS2369-02-001.VNJ	Chinese	F	NM_018062.3(FANCL):c.933T>A	Stage 2 (sigmoid) CRC 69y
634	CGS2419-01-001.TSC	British-Chinese	F	NM_000251.2(MSH2):c.1801C>T	FIGO1 Endometrial endometrioid carcinoma, MSH2/MSH6 deficient 48y
635	CGS3146-01-001.LFY	Malay	F	NM_000051.4(ATM):c.3078G>T	L Breast CA ER/PR-Her2+ 38y
636	CGS3230-01-001.LKH	Chinese	F	NM_006231.3(POLE):c.1191C>G	FIGO stage IVB USC, pMMR MSS, HER2-<br>Stage IIIC HGS ovarian CA 56y
637	CGS3375-01-001.ABM	Malay	F	NM_000251.2(MSH2):c.1661+5G>A	HGSC Ovarian CA 54y
638	CGS3385-01-001.RBH	Malay	F	NM_021930.6(RINT1):c.310C>T	G3 IDC ER/PR+, cerbB2- L Breast CA 39y
639	CGS3455-01-001.TSL	Unknown	F	NM_021930.6(RINT1):c.1966C>T	Grade 2 IDC ER/PR+ HER2- Breast CA 49y
640	CGS3503-01-001.KJM	Chinese	F	NM_000051.4(ATM):c.7989_7991del	G3 IDC ER 3/8 PR 2/8 HER 2+ L Breast CA 33y
641	CGS3516-01-001.PSR	Indian	F	NM_000051.4(ATM):c.6154G>A	IDC G3, ER 90% PR 1% HER2- L Breast CA 37y
642	CGS3704-01-001.SPB	Malay	F	NM_007294.4(BRCA1):c.2726dup	FIGO stage IVB HGSC R Ovarian CA 53y
643	CGS3747-01-001.SSC	Chinese	M	NM_000249.3(MLH1):c.1937A>G	Pancreatic adenocarcinoma, MLH1/PMS2 deficient 48y
644	CGS3814-01-001.ND	Unknown	F	NM_000051.4(ATM):c.3078G>T	IDC G2 ER/PR+ HER2 equivocal FISH- L Breast CA 37y
645	CGS3949-01-001.DTG	Chinese	M	NM_000249.3(MLH1):c.918T>A	Adenocarcinoma CRC 37y
646	CGS4010-01-002.KSM	Unknown	F	NM_007294.4(BRCA1): c.(?_?)del (exons 1_2del)	R Breast CA 44y<br>IDC TNBC L Breast CA 58y
647	CGS4092-01-001.LLH	Chinese	F	NM_000251.2(MSH2):c.942+2T>A	IB2 G3 Adenoca with clear cell features Endometrial CA 39y
648	CGS4109-01-001.IDR	Indian	F	NM_000465.3(BARD1):c.1678-3C>G	IDC provi G3 ER/PR+ HER2 equivocal FISH -ve L Breast CA 28y<br>IDC ER/PR+ CERB2 equivocal R Breast Ca 50y
649	CGS4117-01-001.SAH	Chinese	F	NM_058216.2(RAD51C):c.571+5G>A	Stage IIIC High grade serous Ovarian CA 70y<br>L Breast CA IDC ER/PR+ HER2- 73y
650	CGS4121-01-001.JNC	Chinese	F	NM_021930.6(RINT1):c.1966C>T	IDC+ILC G2 ER/PR+ HER2- R Breast CA 53y
651	CGS4220-01-002.LLH	Chinese	F	NM_007294.4(BRCA1):c.4372C>T	R Breast CA G2 IDC ER+PR+HER2- 50y<br>IDC L Breast CA G3 ER/PR+ HER2 equivocal FISH- 66y

652	CGS4291-04-001.MNB	Malay	M	NM_000546.6(TP53): c.(?_?)del (promoter)	HSPC Prostate CA 71y<br>SCC<br>Sarcoma 71y
653	CGS4307-01-001.THG	Chinese	F	NM_021930.6(RINT1):c.1966C>T	IDC G2 with high grade DCIS, ER/PR- HER2 equivocal R Breast CA 49y
654	CGS4322-01-001.FSH	Chinese	F	NM_021930.6(RINT1):c.1966C>T	IDC ER/PR Her2- R Breast CA 68y
655	CGS4361-01-001.BWJ	Chinese	M	NM_003002.3(SDHD):c.3G>C	PGL/PCC 23y
656	CGS4376-01-001.VAG	Unknown	F	NM_000059.3(BRCA2):c.4631del	Stage IVB HGS Ovarian CA 44y
657	CGS4390-01-001.BJ	Unknown	F	NM_007294.4(BRCA1):c.3770_3771del	ER+PR-HER2 2+ (FISH –ve)L Breast CA 45y
658	CGS4404-01-001.LNH	Chinese	F	NM_000251.2(MSH2):c.970C>T	1st CRC 57y<br>2nd Sigmoid mod ddx adenoca CRC 71y<br>MSH/MSH6 deficient<br>Stage 1A endometrial ca
659	CGS4407-01-001.TMW	Chinese	F	NM_006231.3(POLE):c.1191C>G	Stage IIA mucinous carcinoma G2 ER/PR+ HER2- R Breast CA 42y
660	CGS4408-01-001.SBR	Malay	M	NM_000059.3(BRCA2):c.9027T>G	Prostatic adenocarcinoma, Gleason 5+4, score 9 Prostate CA 67y
661	CGS4410-01-001.LSH	Malay	F	NM_058216.2(RAD51C):c.905-2A>C	Stage 4B HGSC Ovarian CA 60y
662	CGS4410-01-003.LSL	Chinese	F	NM_058216.2(RAD51C):c.905-2A>C	L Breast CA 45y
663	CGS4425-01-001.LAN	Chinese	F	NM_005236.2(ERCC4):c.2169C>A	Clear cell adenocarcinoma Ovarian CA 41y<br>Endometrial endometroid carcinoma, FIGO g1, Endometrial CA 41y
664	CGS4426-01-001.NBA	Malay	F	NM_000059.3(BRCA2):c.3106G>T	IDC G2 DCIS ER/PR+ HER2- Breast CA 60y<br>Stage IIIC PPC High grade serous carcinoma Ovarian CA 61y
665	CGS4441-01-001.TSW	Chinese	F	NM_020975.4(RET):c.1858T>C	Multifocal MTC Thyroid CA 26y
666	CGS4442-01-001.FPF	Chinese	F	NM_020937.4(FANCM):c.2605_2606del	TNBC Breast CA 61y
667	CGS4446-01-001.KDG	Indian	F	NM_000136.2(FANCC): c.(?_?)del (exon 4)	IDC ER/PR+ HER2- Breast CA 52y
668	CGS4460-01-001.NSH	Chinese	F	NM_000553.4(WRN):c.2194C>T	BRAF v600E mut cutaneous Melanoma 48y
669	CGS4472-01-001.JN	Chinese	M	NM_144997.5(FLCN):c.1432+1G>C	G1 Renal cell carcinoma 53y
670	CGS4477-01-001.SML	Chinese	F	NM_000535.4(PMS2):c.2174+1G>A	Caecal adenoca, PMS2 loss CRC 65y
671	CGS4482-01-001.TAH	Unknown	F	NM_007294.4(BRCA1):c.81-2del	Stage 2B HGSC Ovarian CA 43y

672	CGS4484-01-001.NSW	Chinese	M	NM_000059.3(BRCA2):c.1763_1766del	Metastatic Pancreatic Adenocarcinoma, Pancreatic CA 54y
673	CGS4508-01-001.TSE	Chinese	F	NM_000059.3(BRCA2):c.9097dup	IDC ER+PR-HER2- Breast CA 52y<br>Synchronous L RCC Renal CA 52y<br>Stage IIIC R fallopian tube HGSC Ovarian CA 63y
674	CGS4518-01-001.OYQ	Chinese	F	NM_000546.6(TP53):c.535C>G	Bilateral synchronous breast cancer Breast CA 26y<br>R Breast G3 ER/PR+ HER2+ IDC with ductal features<br>L Breast G2 ER/PR+ HER2+ IDC with ductal features
675	CGS4519-01-001.AG	Chinese	F	NM_001283009.2(RTEL1):c.3715_3716del	MDS in transformation to AML 37y
676	CGS4525-01-001.JCH	Chinese	F	NM_000179.3(MSH6):c.2230dup	FIGO stage IIB OCCC Ovarian CA 51y
677	CGS4526-01-001.LLH	Chinese	F	NM_007294.4(BRCA1):c.5089T>C	Infiltrative ductal carcinoma, ER+PR- HER2- Breast CA 47y
678	CGS4529-01-001.SPK	Chinese	F	NM_024675.3(PALB2):c.7G>T	DCIS ER+ L Breast CA 47y
679	CGS4548-01-001.SBH	Chinese	F	NM_005236.2(ERCC4):c.1447_1450del	L Breast mucinous ca, G1 ER/PR+HER2- Breast CA 37y
680	CGS4560-01-001.AGE	Chinese	F	NM_058216.2(RAD51C):c.571+5G>A	Invasive ca w/ apocrine features, R TNBC 49y
681	CGS4565-01-001.MRK	Indian	F	NM_020937.4(FANCM):c.4153G>T	R TNBC Breast CA 59y
682	CGS4566-01-001.YPC	Chinese	F	NM_000051.4(ATM):c.2413C>T	Recurrent metastatic Breast CA 51y<br>Invasive carcinoma ER/PR+ HER2-
683	CGS4567-01-001.FMS	Chinese	M	NM_000059.3(BRCA2):c.4799del	Primary refractory metastatic cervical esophageal SCC 66y
684	CGS4573-01-001.ADS	Indian	F	NM_007294.4(BRCA1):c.68_69del	FIGO stage IVB HGSC R fallopian tube, Ovarian CA 45y
685	CGS4574-01-001.ABM	Malay	F	NM_000249.3(MLH1):c.1865T>C	Stage 3A, caecal adenocarcinoma, pMMR CRC 40y

686	CGS4600-01-001.HL-	Chinese	F	NM_000179.3(MSH6):c.3458_3459insTA	Synchronous Stage 2B CA ovary endometrioid adenoca G2 46y<br>Stage 1A CA endometrium endometrioid adenoca, MSH6 loss G1
687	CGS4604-01-001.AGS	Chinese	F	NM_000059.3(BRCA2):c.2612C>G	Recurrent bilateral Breast CA<br>T1cN1M0, stage 2 R Breast 34y<br>DCIS ER/PR+ L Breast 44y & 56y
688	CGS4610-01-001.WSC	Chinese	F	NM_000051.4(ATM): c.(?_?)del (exons 62_63del)	Metastatic Liver-Limited mid-rectal adenocarcinoma, pMMR CRC 39y
689	CGS4627-01-001.TPS	Chinese	F	NM_000059.3(BRCA2):c.774_775del	Synchronous L Breast IDC G2 ER/PR+ HER2+ Breast CA 58y<br>Stage III PPC, Ovarian CA 58y
690	CGS4628-01-001.AW	Chinese	M	NM_003000.2(SDHB):c.136C>T	Noradrenergic PPGL Syndrome ,PGL/PCC 13y
691	CGS4646-01-001.CWT	Unknown	F	NM_058216.2(RAD51C):c.1003_1004insT TCC	Metachronous stage I G2 ascending colon adenoCa CRC 75y<br>Recurrent FIGO IVA HGSC R fallopian tube, Ovarian CA 80y
692	CGS4647-01-001.TCI	Chinese	F	NM_002878.3(RAD51D):c.270_271dup	3C HGSC Ovarian CA 48y
693	CGS4657-01-001.LC	Chinese	M	NM_000546.6(TP53):c.375G>A	Metastatic advanced CS prostate adenocarcinoma 80y
694	CGS4686-01-001.OZH	Chinese	M	NM_000249.3(MLH1):c.790+1G>A	Mucinous adenoca, PMS2/MLH1 absent CRC 32y
695	CGS4688-01-001.MYB	Indian	M	NM_000251.2(MSH2):c.1786_1788del	Stage 2 ascending colon invasive adenocarcinoma, MSH2/MSH6 loss, CRC 59y
696	CGS4700-01-001.KMS	Chinese	F	NM_002878.3(RAD51D):c.270_271dup	TNBC R Breast CA 46y
697	CGS4710-01-001.LYY	Chinese	F	NM_024675.3(PALB2):c.3256del	Metastatic pancreatic body adenocarcinoma, pMMR 48y
698	CGS4719-01-001.LMJ	Chinese	F	NM_000059.3(BRCA2):c.1103C>A	Invasive ca Gr2 ER/PR+ HER2 equivocal L Breast CA 34y
699	CGS4729-01-001.SCT	Chinese	M	NM_000059.3(BRCA2):c.3109C>T	GS 5+3 (ISUP G4)Prostate CA 73y
700	CGS4747-01-001.LCL	Chinese	F	NM_020975.4(RET):c.1888T>C	TNBC Breast CA 62y
701	CGS4750-01-001.CTT	Chinese	F	NM_007294.4(BRCA1):c.3661G>T	Recurrent HGSC Ovarian CA 44y
702	CGS4761-01-001.TSN	Chinese	F	NM_000051.4(ATM):c.8435_8436del	IDC ER/PR+ Her2- L Breast CA 44y

703	CGS4765-01-001.CHL	Chinese	F	NM_000492.3(CFTR):c.1210-34TG[12]T[5]	IDC ER/PR+ cerbB2 equivocal R Breast CA 41y
704	CGS4770-01-001.YSM	Chinese	F	NM_000492.3(CFTR):c.1210-34TG[12]T[5]	Stage IV pancreatic adenoca, pMMR 50y
705	CGS4773-01-001.CHH	Chinese	M	NM_000546.6(TP53):c.1101-1G>A	Oligomets L femur MFH (soft tissue sarcoma) 28y<br>Descending colon signet ring cell carcinoma, pMMR CRC 34y
706	CGS4776-01-001.PT	Chinese	F	NM_000553.4(WRN):c.673C>T	G3 ER+PR+HER2- IDC R Breast CA 38y
707	CGS4792-01-001.PGL	Chinese	F	NM_000179.3(MSH6):c.3838C>T	Stage 1A endometrial endometrioid carcinoma, FIGO G2 61y
708	CGS4793-01-001.LYK	Chinese	M	NM_000249.3(MLH1):c.1937A>G	Denovo Metastatic Hormone Sensitive (G 5+4) Prostate CA 75y
709	CGS4796-01-001.TSH	Chinese	F	NM_000059.3(BRCA2):c.3109C>T	Fallopian tube HGSC Stage3C Ovarian CA 68y
710	CGS4813-01-001.AKS	Unknown	F	NM_000059.3(BRCA2):c.2818C>T	ILC ER/PR+ HER2- Breast CA 40y
711	CGS4813-02-001.GSM	Unknown	F	NM_000059.3(BRCA2):c.2818C>T	L Breast CA 58y
712	CGS4817-01-001.NJJ	Chinese	F	NM_007294.4(BRCA1):c.4372C>T	TNBC Breast CA 23y
713	CGS4822-01-001.CLH	Chinese	F	NM_000059.3(BRCA2):c.2808_2811del	FIGO stage IIIC tubal HGSC, HRD+ Ovarian CA 69y
714	CGS4825-01-001.ESB	Indian	F	NM_177438.3(DICER1):c.5387_5388del	Sertoli-Leydig cell tumor, Ovarian CA 13y
715	CGS4833-01-001.CHH	Chinese	F	NM_000135.2(FANCA):c.709+5G>A	FIGO IC3 HGSC L ovary, ER histoscore 180 Ovarian CA 33y
716	CGS4852-01-001.DSA	Indian	F	NM_000038.6(APC):c.3920T>A	IDC ER/PR+ HER2- Breast CA 37y
717	CGS4860-01-001.NYH	Chinese	F	NM_000059.3(BRCA2):c.7878G>A	IDC G1 ER+PR+HER2-Breast CA 38y
718	CGS4866-01-001.LKF	Chinese	F	NM_000059.3(BRCA2):c.771_775del	R DCIS Breast CA 44y<br>L Breast IDC ER/PR+ c-ERB-B2 equivocal 66y<br>Uterine leiomyosarcoma 66y
719	CGS4884-01-001.ZH	Chinese	F	NM_006231.3(POLE): c.(?_?)del (exons 19_23del)	IDC ER-PR+cerB2+ L Breast CA 38y
720	CGS4914-01-001.EBA	Malay	F	NM_000059.3(BRCA2):c.9097dup	PPC HGSC Ovarian CA 44y
721	CGS4924-01-001.LKK	Chinese	F	NM_000051.4(ATM):c.1402_1403del	R IDC Breast CA 43y

722	CGS4930-01-001.OPH	Chinese	F	NM_000057.4(BLM):c.3299del	Stage II ccRenal CA 57y<br>IDC ER/PR+ c-ERB-B2- R Breast CA 58y<br>IDC ER/PR+ c-erb-B2- L Breast CA 58y
723	CGS4946-01-001.CSL	Chinese	F	NM_000051.4(ATM):c.1369C>T	mixed ILC/IDC ER+PR+HER2- 42y<br>ILC ER+PR-HER2- 50y<br>Distal cholangiocarcinoma 58y<br>Metastatic adenocarcinoma ER+PR-HER2- 59y
724	CGS4947-01-001.TEL	Chinese	F	NM_000143.3(FH):c.1189G>A	Multiple fibroids (leiomyomata) 32y<br>L breast intraductal papilloma 37y<br>Papillary Renal cell CA 37y<br>Hepatic adenomas 37y
725	CGS4950-01-001.TSC	Chinese	F	NM_000059.3(BRCA2):c.1591A>T	IDC Triple+ R Breast CA 40y
726	CGS4956-01-001.CTK	Chinese	F	NM_001184.4(ATR):c.4912C>T	IDC G1 ER/PR+Her2- R Breast Ca 63y<br>High Grade DCIS L Breast CA 63y
727	CGS4957-01-001.AGK	Chinese	F	NM_007194.3(CHEK2):c.433C>T	Metachronous R breast ER/PR+Her2 36y<br>IDC G3 R Breast 48y<br>L Breast DCIS 48y
728	CGS4958-01-001.CW	Chinese	F	NM_000135.2(FANCA):c.1748del	IDC G1 ER/PR+Her2- L Breast CA 35y
729	CGS4972-01-001.LWK	Chinese	M	NM_007194.3(CHEK2):c.319+1G>A	Metastatic pancreatic adenocarcinoma – Stage IV Pancreatic CA 74y
730	CGS4984-01-001.KS	Chinese	F	NM_000059.3(BRCA2):c.3109C>T	R Breast Ca 34y<br>G2 DCIS/LCIS ER+PR-HER2- L Breast 43y<br>Metastatic IDC ER/PR+HER2 equivocal Breast CA 46y
731	CGS4988-01-001.BPL	Chinese	F	NM_024675.3(PALB2):c.2996+1G>T	TNBC Breast CA 37y
732	CGS4998-01-001.CSF	Chinese	F	NM_007294.4(BRCA1):c.4185+1G>A	FIGO stage IVB HGSC Ovarian CA 68y
733	CGS5024-01-001.TSC	Chinese	F	NM_006502.3(POLH):c.272+1G>C	Xeroderma Pigmentosum<br>SCC 55y
734	CGS5033-01-001.CST	Chinese	F	NM_003001.3(SDHC): c.(?_?)del (exons 2_3del)	ER/PR-Her2+ Invasive carcinoma w/ apocrine differentiation, Breast CA 75y
735	CGS5055-01-001.NJT	Unknown	F	NM_000059.3(BRCA2):c.6275_6276del	Metastatic ER/PR+Her2- Breast CA 46y

736	CGS5061-01-001.MLG	Chinese	F	NM_000051.4(ATM):c.8224_8225del	ER+PR+HER2+ L Breast CA 42y<br>Pancreatic tail ductal adenocarcinoma, Pancreatic CA 60y<br>LLL lung adenocarcinoma, Lung CA 60y
737	CGS5080-01-001.CPC	Chinese	M	NM_000135.2(FANCA):c.1777-1G>C	Metastatic CRPC to bones (Gleason 4+4)<br>Prostate CA 57y
738	CGS5082-01-001.WBW	Indian	F	NM_002485.4(NBN):c.1882_1885del	L breast DCIS + R breast ALH ER/PR+ Breast CA 64y
739	CGS5083-01-001.NYK	Chinese	M	NM_002834.3(PTPN11):c.782T>A	Adenocarcinoma IIA (G3+4= 7) Prostate CA 70y<br>Chronic myelomonocytic leukaemia 70y<br>IIIA Urothelial Bladder CA 75y
740	CGS5110-01-001.LSM	Chinese	F	NM_000059.3(BRCA2):c.5467A>T	DCIS Breast CA 45y
741	CGS5112-01-001.LYY	Chinese	F	NM_000368.5(TSC1):c.2625+367A>G	High-grade DCIS ER/PR- R Breast CA 75y<br>Stage III G3 TNBC ER/PR-HER2 equivocal L Breast CA 75y
742	CGS5115-01-001.SK	Indian	M	NM_000059.3(BRCA2):c.9276T>G	Metastatic high grade papillary urothelial carcinoma 65y<br>Metachronous localised stage 2b Invasive carcinoma ER/PR+Her2equivocal Breast CA 67y
743	CGS5119-01-001.SAM	Unknown	F	NM_000051.4(ATM):c.6100C>T	IDC+ILC G2 ER/PR+HER2- Breast CA 55y
744	CGS5128-01-001.SHB	Malay	F	NM_033084.3(FANCD2):c.3103C>T	DCIS ER/PR+ R Breast CA 40y
745	CGS5138-01-001.QLK	Chinese	F	NM_007294.4(BRCA1):c.4327C>T	TNBC L Breast CA 72y
746	CGS5161-01-001.ARV	Unknown	F	NM_002439.3(MSH3):c.2871_2874del	Synchronous G1 IDC R Breast CA 48y<br>G2 IDC ER+PR+HER2- L Breast CA 48y
747	CGS5165-01-001.NBA	Malay	F	NM_004168.3(SDHA):c.923C>T	R breast IDC Gr2 ER/PR+ HER2 equivocal 60y<br>L breast IDC Gr2 ER/PR+ HER2 equivocal 60y
748	CGS5176-01-001.TMC	Chinese	F	NM_000179.3(MSH6):c.3401dup	Stage 1A Endometrioid Carcinoma dx age 38<br>IHC: MSH6 loss, MSI high
749	CGS5204-01-001.TBC	Chinese	F	NM_002878.3(RAD51D):c.270_271dup	FIGO IIB HGSC of FT, Ovarian CA 67y<br>FIGO I endometrioid endometrial carcinoma (pMMR) 67y

750	CGS5220-01-001.RJ	Indian	F	NM_007294.4(BRCA1):c.66dup	Stage 2 HGSC, Ovarian CA 36y
751	CGS5226-01-001.FSC	Unknown	F	NM_000059.3(BRCA2):c.9097dup	LABC ER/PR+HER2- R Breast CA 39y
752	CGS5233-01-001.JDR	Indian	F	NM_005732.3(RAD50):c.1966C>T	Stage 3C HGSC Ovarian CA 47y
753	CGS5237-01-001.EET	Unknown	F	NM_000059.3(BRCA2):c.1572del	R breast: G2 ILC ER/PR+ HER2- 46y<br>L breast: G1 ER/PR+ Her2 equivocal 46y
754	CGS5248-01-001.ABA	Malay	F	NM_000179.3(MSH6):c.3477C>G	Stage 3B, MSH6 loss Endometrial CA 68y
755	CGS5249-01-001.LKL	Chinese	F	NM_000059.3(BRCA2):c.7480C>T	Stage 3B HGSC Ovarian CA 40y
756	CGS5253-01-001.ASM	Unknown	F	NM_007294.4(BRCA1):c.5035del	Grade 3 IDC ER/PR+ HER2- R Breast CA 37y<br>DCIS ER/PR- R Breast CA 50y
757	CGS5258-01-001.NBO	Malay	F	NM_000059.3(BRCA2):c.3106G>T	L ovarian tumour cx by peritoneal metastases & moderate ascites 69y
758	CGS5271-01-001.KAE	Chinese	F	NM_007294.4(BRCA1): c.(?_?)del (exons 1_15del)	Stage 4 HGSC Ovarian CA 78y
759	CGS5274-01-001.GSP	Chinese	F	NM_007294.4(BRCA1):c.191G>C	Stage 2B HGSC Ovarian CA 52y
760	CGS5300-01-001.SSH	Chinese	F	NM_000135.2(FANCA):c.709+5G>A	ILC ER/PR+ HER2- G2 R Breast CA 49y
761	CGS5315-01-001.JDK	Unknown	F	NM_003000.2(SDHB): c.(?_?)del (exon 1)	IDC G2 ER/PR+ HER2- R Breast CA 57y
762	CGS5323-01-001.TLS	Chinese	M	NM_000249.3(MLH1):c.790+1G>A	Rectal adenocarcinoma 50y<br>Small bowel mucinous adenocarcinoma 51y
763	CGS5335-01-001.CTK	Chinese	M	NM_000051.4(ATM):c.7989_7991del	Locally advanced pancreatic adenocarcinom, pMMR Pancreatic CA 67y
764	CGS5344-01-001.YL	Chinese	F	NM_000535.4(PMS2):c.1577del	IDC G2 ER/PR+ HER2- R Breast CA 33y
765	CGS5354-01-001.WLL	Chinese	F	NM_001128425.1(MUTYH):c.643G>A	Metastatic IDC G3 ER/PR+ c-erb-B2(HER2)- R Breast CA 44y
766	CGS5368-01-001.VH	Indian	F	NM_000535.4(PMS2):c.1376C>A	Stage IIA IDC ER/PR+ HER2+ L Breast CA 48y
767	CGS5368-02-001.KRM	Indian	F	NM_000535.4(PMS2):c.1376C>A	Endometrial CA 65y
768	CGS5376-01-001.JLK	Chinese	F	NM_000267.3(NF1):c.2953C>T	FIGO stage IIB L tubal HGSC, ER- Ovarian CA 60y
769	CGS5395-01-001.WSL	Chinese	F	NM_024675.3(PALB2):c.693dup	TNBC IDC L Breast CA 67y<br>IDC ER/PR+ Her2- R Breast CA 67y
770	CGS5397-01-001.DKD	Unknown	F	NM_058216.2(RAD51C):c.701C>G	Stage 4B carcinosarcoma Ovarian CA 56y
771	CGS5407-01-001.JBS	Chinese	F	NM_000179.3(MSH6):c.2569_2572del	Stage 3C2 Endometrial endometroid carcinoma,<br>MSI-H, MLH1/PMS2/MSH6 loss 66y

772	CGS5417-01-001.LNY	Chinese	F	NM_000249.3(MLH1):c.1162del	FIGO IIIB G3 endometrioid Ca endometrium, MSI-H, MLH1/PMS2 loss 60y
773	CGS5424-01-001.FGS	Chinese	F	NM_000535.4(PMS2):c.1831dup	ILC ER/PR+ HER2 equivocal R Breast CA 68y
774	CGS5426-01-001.CVY	Chinese	M	NM_000267.3(NF1):c.7858_7861dup	Neurofibromatosis Type1 9y<br>Right thigh Malignant Peripheral Nerve Sheath Tumor 29y
775	CGS5430-01-001.MBH	Malay	F	NM_024675.3(PALB2):c.338del	IDC G3 TNBC R Breast CA 41y<br>Stage II IDC G3 ER/PR-, HER2+ L Breast CA 64y
776	CGS5443-01-001.CGN	Chinese	F	NM_007294.4(BRCA1):c.5470_5477del	G2-3 IDC TNBC R Breast CA 50y
777	CGS5485-01-001.GLC	Chinese	F	NM_007294.4(BRCA1): c.(?_?)del (exons 16_18del)	Stage 3C HGS ovarian adenoCA 60y
778	CGS5486-01-001.WSF	Unknown	F	NM_000136.2(FANCC):c.640dup	Metastatic IDC breast ER/PR+ c-ERB-B2- Breast CA 60y
779	CGS5489-01-001.HPS	Chinese	F	NM_000059.3(BRCA2):c.7480C>T	DCIS ER/PR+ L Breast CA 41y<br>IDC G3 ER/PR+ HER2- 43y
780	CGS5503-01-001.EC	Unknown	M	NM_020975.4(RET):c.2410G>A	Medullary Thyroid cancer stage IVA, Thyroid CA 53y
781	CGS5517-01-001.EH	Indian	F	NM_000135.2(FANCA):c.862G>T	DCIS L Breast CA 36y<br>IDC ER/PR+ HER2- L Breast CA 39y
782	CGS5519-01-001.RS	Unknown	F	NM_007294.4(BRCA1):c.5357T>C	Stage IIA G3 TNBC R Breast CA 43y<br>FIGO stage IIIC L tubal carcinosarcoma Ovarian CA 55y
783	CGS5528-01-001.MY	Chinese	F	NM_000546.6(TP53):c.818G>A	TNBC R Breast CA 41y
784	CGS5537-01-001.AN	Indian	F	NM_000546.6(TP53):c.817C>T	WHO G3 Left fronto-parietal choroid plexus carcinoma, CPC 3y
785	CGS5551-01-001.NBM	Malay	F	NM_000059.3(BRCA2):c.7964_7965delAA	ILC ER/PR+ c-erbB2- R Breast CA 47y
786	CGS5559-01-001.PNL	Chinese	F	NM_000051.4(ATM):c.3078G>T	Invasive mucinous carcinoma ER/PR+ HER2- R Breast CA 67y
787	CGS5588-01-001.JTN	Chinese	F	NM_002878.3(RAD51D):c.270_271dup	Stage 4 with peritoneal metastasis Ovarian CA 52y

788	CGS5608-01-001.BDP	Indian	F	NM_001128425.1(MUTYH):c.1438G>T	Stage 1- ER/PR+ Her2 equivocal R Breast CA 36y
789	CGS5619-01-001.NHL	Chinese	F	NM_002528.2(NTHL1):c.699_700del	DCIS ER/PR+ R Breast CA 44y
790	CGS5621-01-001.BKA	Unknown	F	NM_000038.6(APC):c.3920T>A	Stage 2B ER/PR+ HER2- R Breast CA 48y
791	CGS5625-01-001.DDT	Indian	F	NM_001128425.1(MUTYH):c.859del	Stage 4 carcinosarcoma Ovarian CA 74y
792	CGS5639-01-001.TLT	Chinese	F	NM_000492.3(CFTR):c.1766+5G>T	Metastatic IDC stage IV ER/PR+ Her2- Breast CA 49y
793	CGS5653-01-001.NAP	Chinese	F	NM_000059.3(BRCA2):c.643G>T	Stage 4B HGSC Ovarian CA 70y
794	CGS5658-01-001.AAB	Unknown	M	NM_000038.6(APC):c.457A>T	Rectosigmoid adenoca CRC 62y<br>Thyroid PTC 62y
795	CGS5669-01-001.TS	Unknown	F	NM_001128425.1(MUTYH):c.1518+2del	ER/PR+ HER2- mixed invasive mucinous & IDC Breast CA 41y
796	CGS5672-01-001.PYL	Chinese	F	NM_000059.3(BRCA2):c.6582del	Stage IIA ER+PR- R Breast CA 42y<br>Stage IIIA IDC G2 ER/PR+ HER2 equivocal L Breast CA 66y
797	CGS5676-01-001.PY	Unknown	F	NM_007294.4(BRCA1):c.3214del	L Breast CA 29y<br>R Breast CA 39y<br>Urothelial papillary cancer 58y
798	CGS5686-01-001.TWS	Chinese	M	NM_000249.3(MLH1):c.114C>A	Stage 1 adenocarcinoma, dMMR PMS2, CRC 64y
799	CGS5698-01-001.II	Malay	M	NM_000321.3(RB1):c.157_158del	Bilateral Retinoblastoma 0y
800	CGS5739-01-001.KA	Unknown	F	NM_000251.2(MSH2): c.(?_?)del (exons 1_3del)	Endometrioid adenocarcinoma G2 ER/PR+ MSH2/MSH6 loss 41y
801	CGS5740-01-001.NBK	Chinese	F	NM_000249.3(MLH1):c.793C>T	Stage 3C HGS bilateral ovarian adenoCA 54y
802	CGS5758-01-001.GHP	Chinese	F	NM_002878.3(RAD51D):c.270_271dup	Uterine cancer IA G1 43y<br>ER/PR+ HER2- L Breast CA 49y
803	CGS5762-01-001.SBA	Malay	F	NM_020975.4(RET):c.2410G>A	Stage III Medullary Thyroid Carcinoma 72y
804	CGS5768-01-001.KBT	Chinese	F	NM_000251.2(MSH2):c.1165del	G2 MSH2/MSH6 loss Endometrioid endometrial CA 44y
805	CGS5770-01-001.WGH	Chinese	F	NM_000535.4(PMS2): c.(?_?)dup (exon 2dup)	Stage 1A G2 Endometrioid CA endometrium MSH6/PMS2 loss (MSI-H) 74y

806	CGS5772-01-001.MNB	Malay	M	NM_000038.6(APC):c.3183_3187del	Rectosigmoid colon adenocarcinoma, pMMR CRC 40y<br>Familial adenomatosis polyposis - multiple polyps >200 40y
807	CGS5785-01-001.TCK	Chinese	M	NM_000251.2(MSH2):c.2360_2361dup	G2 mucinous Sigmoid cancer, MSH-2: equivocal MSH-6:(-)ve, MSI-H CRC 59y
808	CGS5787-01-001.KMJ	Unknown	F	NM_000249.3(MLH1):c.116+1G>A	Stage II adenocarcinoma, MLH1/PMS2 loss, CRC 43y
809	CGS5795-01-001.OSL	Chinese	F	NM_032043.2(BRIP1): c.(?_?)del (exons 5_6del)	Synchronous Bilateral Breast Cancers HER2+ 69y<br>L Breast: invasive carcinoma G2 ER/PR-HER2+<br>R Breast: invasive carcinoma G2 ER/PR-HER2+
810	CGS5819-01-001.LLS	Chinese	F	NM_007294.4(BRCA1):c.4327C>T	3C R ovarian HGSC Ovarian CA 65y
811	CGS5862-01-001.THM	Chinese	F	NM_007294.4(BRCA1):c.3424delG	TNBC L Breast CA 28y<br>TNBC R Breast CA 32y
812	CGS5889-01-001.LHL	Chinese	F	NM_058216.2(RAD51C):c.394dup	FIGO IIIC2 Ca endometrium, pMMR 58y<br>FIGO IA HGSC Ovarian CA 58y
813	CGS5926-01-001.SSL	Chinese	F	NM_000059.3(BRCA2):c.8400_8402delins AAAA	Stage 3 Type 1/2 GEJ tumor extending to gastric cardia, Her2 2+ 65y<br>Invasive carcinoma G3 ER/PR+ c-erb-B2 equivocal R Breast CA 69y
814	CGS5991-01-001.LKH	Chinese	M	NM_000492.3(CFTR):c.1210-34TG[13]T[5]	Prostate CA 82y<br>PDAC of pancreatic tail, metastatic adenocarcinoma 84y
815	CGS5995-01-001.LYS	Chinese	M	NM_000051.4(ATM):c.7989_7991del	Stage 3 duodenum adenocarcinoma, pMMR 78y
816	CGS6007-01-001.SIB	Unknown	F	NM_000051.4(ATM):c.8373C>A	IDC ER/PR+ HER2 FISH- L Breast CA 41y
817	CGS6028-01-001.PS	Unknown	M	NM_007294.4(BRCA1):c.1621C>T	L thyroid Medullary Ca 43y
818	CGS6031-01-001.HKH	Chinese	F	NM_032043.2(BRIP1):c.1066C>T	Stage 4 HGSC Ovarian CA 87y
819	CGS6084-01-001.HLL	Chinese	F	NM_002439.3(MSH3):c.724C>T	Metastatic G2 IDC ER/PR-HER2 Equivocal R Breast CA 52y

820	CGS6085-01-001.RBY	Malay	F	NM_001379610.1(SPINK1):c.101A>G	Metastatic distal body and tail pancreatic adenocarcinoma, pMMR 61y
821	CGS5136-01-001.SJ	Chinese	F	NM_000267.3(NF1):c.910C>T	Left chest wall neurofibroma<br>Uterine fibroids
822	CGS6107-01-001.LBH	Chinese	M	NM_003002.3(SDHD):c.3G>C	Bilateral carotid body tumour
823	CGS4543-01-001.CTK	Chinese	F	NM_000267.3(NF1):c.3299dup	NF1 with multiple UBOs<br>Moya-moya disease
824	CGS5214-01-001.NBH	Malay	F	NM_032043.2(BRIP1):c.2247del	hepatic flexure polyp, 3mm TA histology
825	CGS5708-01-001.PKY	Chinese	F	NM_000143.3(FH):c.701C>A	Fibroid - fumarate hydratase deficiency<br>Uterine septum<br>RO endometriotic cyst
826	CGS5439-01-001.YS	Chinese	F	NM_000267.3(NF1):c.5515del	Neurofibromatosis Type 1
827	CGS4613-01-001.LLH	Chinese	F	NM_000038.6(APC):c.896_897del	Ovarian cyst
828	CGS5947-01-001.PTK	Chinese	M	NM_144997.5(FLCN):c.1579_1580insA	cystic lung disease<br>pneumothorax<br>Fistula-in-ano<br>lung cysts
829	CGS5997-01-001.QR	Chinese	F	NM_024675.3(PALB2):c.2167_2168delAT	Breast lumps
830	CGS4567-03-003.FTW	Chinese	M	NM_000059.3(BRCA2):c.4799del	Polyps
831	CGS4815-01-001.PLZ	Chinese	M	NM_000267.3(NF1):c.2155dup	Neurofibromas<br>Lump on right gluteal area - l
832	CGS5157-01-001.CSW	Chinese	F	NM_000077.4(CDKN2A): c.(?_?)del (entire coding sequence)	Large retroperitoneal mass and Right Ovarian dermoid cyst
833	CGS5655-01-001.KSC	Chinese	M	NM_007294.4(BRCA1):c.5470_5477del	Atypical meningioma (WHO grade 2)<br>Scar epilepsy<br>Hyperthyroidism
834	CGS5297-01-001.THC	Chinese	M	NM_000251.2(MSH2):c.2038C>T	Multiple flat advanced colorectal tubular adenomas
835	CGS5554-01-001.HLM	Chinese	F	NM_000267.3(NF1):c.5749+332A>G	Neurofibromatosis Type 1<br>Liver cysts<br>Breast subcutaneous nodule, benign breast cysts

836	CGS5211-01-001.MDH	Malay	M	NM_000551.3(VHL):c.481C>T	duplex renal arteries showing multiple cysts - like PCKD
837	CGS4728-01-001.YCS	Chinese	F	NM_004656.4(BAP1):c.1203dup	combined nevus (banal and Spitzoid nevus/melanocytoma)
838	CGS6151-01-001.AVO	Filipino	F	NM_001354712.2(THRB):c.1357C>A	Resistance to thyroid hormone<br>Memory loss
839	CGS4867-01-001.NYL	Chinese	F	NM_144997.5(FLCN):c.1579_1580insA	Lung cysts with bilateral PTX
840	CGS4594-01-001.LTT	Chinese	F	NM_000535.4(PMS2):c.825A>G	Left breast lump removed -benign<br>Bilateral hypoechoic nodules (benign appearance)<br>Right shoulder rotator cuff tear s/p right shoulder arthroscopic rotator cuff repair
841	CGS5006-01-001.HKD	Sikh	F	NM_000038.6(APC):c.1744-2A>G	Desmoid tumour<br>Multiple polyps
