# Supplementary material for: Multi-locus inherited neoplasia alleles syndromes in cancer: implications for clinical practice
Source: Eur J Hum Genet. 2025 Jan 23;33(3):289–96. doi: 10.1038/s41431-025-01785-1 (PMC11894078; doi:10.1038/s41431-025-01785-1)
Supplement: Supplementary file 3 — Supplementary Table 3: Non-carrier / Negative cases [file 41431_2025_1785_MOESM3_ESM.pdf]

Supplementary Table 3: Non-carrier / Negative cases

CGS ID	Case	Ethnicity	Sex	Clinical Hx with age dx (if known)
CGS0711-01-001.JBS	1	Malay	F	Breast ca 33y
CGS1537-01-001.HBJ	2	Malay	F	Breast ca 52y
CGS2930-01-001.WPS	3	Chinese	F	Breast ca 42y
CGS1726-01-001.YCF	4	Chinese	M	Paraganglioma 79y
CGS4314-01-001.RBD	5	Malay	F	Breast ca 51y
CGS3181-01-001.PEL	6	Caucasian	F	Breast ca 56y
CGS0033-01-001.ATK	7	Chinese	F	Breast ca 39y
CGS0059-01-001.OLH	8	Chinese	F	Ovarian ca 52y
CGS1746-01-001.JKL	9	Filipino	F	Breast ca 30y
CGS1222-01-001.RSD	10	Caucasian	M	Thyroid ca 51y
CGS0493-01-001.GQ	11	Chinese	F	Breast ca 29y
CGS0859-01-001.MML	12	Filipino	F	Breast ca 53y
CGS3190-01-001.GR	13	Indian	F	Breast ca 35y; Endometriosis, Infertility
CGS2453-01-001.NP	14	Indian	F	Breast ca 45y
CGS4335-01-001.ZS	15	Chinese	F	Pancreatic ca 73y; Pulmonary embolism, Right ureteric stenosis
CGS3537-01-001.PLH	16	Chinese	F	Pancreatic ca 61y
CGS0021-01-001.MJT	17	Caucasian	F	Ovarian ca 53y
CGS2473-01-001.WL	18	Chinese	F	Pancreatic ca 54y
CGS3528-01-001.EMC	19	Filipino	F	Breast ca 51y, other unknown ca age unknown; undifferentiated MPN ?CNL
CGS0048-01-001.DSW	20	ıcasian (Brit	F	Breast ca 31y
CGS3014-01-001.HH	21	Burmese	F	Gastric ca 35y
CGS3889-01-001.SA	22	Indian	F	No cancer 43y; Bilateral breast nodules, Uterine fibroids, endometriosis and adenomyosis, Endometriosis
CGS1056-01-001.LB	23	Caucasian	F	Breast ca 36y
CGS2298-01-001.LMK	24	Chinese	F	Breast ca 35y, Fourth ventricular tumour - haemangioblastoma 52y; Retinal angiomas
CGS2244-01-001.LSC	25	Chinese	F	Kidney ca 56y; Polycystic liver disease, Several cysts in both kidneys
CGS2342-01-001.KBI	26	Malay	F	Breast ca 37y
CGS0229-01-001.CFL	27	Chinese	F	Breast ca 32y
CGS0240-01-001.KWH	28	Chinese	F	Breast ca 35y
CGS1296-01-001.NSL	29	Chinese	F	Intraabdominal right paravertebral tumour 11y; ROHHADNET syndrome
CGS0258-01-001.BGL	30	Chinese	F	Paraganglioma 15y
CGS0260-01-001.KLK	31	Chinese	M	Kidney ca 34y
CGS0261-01-001.MHO	32	UAE	F	Breast ca 38y
CGS0262-01-001.FT	33	Chinese	F	Breast ca 28y
CGS0264-01-001.CSI	34	Malay	F	Kidney ca 34y
CGS0265-01-001.HX	35	Chinese	F	Breast ca 32y
CGS0270-01-001.LI	36	Indonesian	F	Breast ca 33y
CGS0271-01-001.LSY	37	Chinese	M	Pancreatic ca 63y
CGS0272-01-001.CWL	38	Chinese	F	Breast ca 34y
CGS0274-01-001.NLC	39	Chinese	F	Breast ca 34y
CGS0280-01-001.LEC	40	Chinese	M	Colorectal ca 50y
CGS0283-01-001.KMY	41	Chinese	F	Breast ca 29y

CGS0284-01-001.HBA	42	Indian	F	Ganglioneuroblastoma 1y; Left Horner syndrome
CGS0289-01-001.JBB	43	Malay	F	Breast ca 43y, Ovarian ca 51y
CGS0292-01-001.TNK	44	Chinese	M	Nasopharyngeal carcinoma 52y
CGS0293-01-001.YPS	45	Chinese	F	Breast ca 31y
CGS0636-01-001.LS	46	Chinese	F	Breast ca 26y
CGS0236-01-001.SBS	47	Malay	F	Breast ca 36y
CGS0027-01-002.MMP	48	Chinese	F	Ovarian ca 39y
CGS0237-01-001.MDH	49	Indian	F	Breast ca 42y, Liver core biopsies: consistent with metastatic breast carcinoma 42y
CGS0239-01-001.HB	50	Indian	F	Ovarian ca 64y
CGS0241-01-001.MVV	51	Indian	F	Breast ca 49y
CGS0242-01-001.LLI	52	Chinese	F	Ovarian ca 44y
CGS0245-01-001.CLQ	53	Chinese	F	Breast ca 30y
CGS0248-01-001.TSP	54	Chinese	F	Breast ca 54y
CGS0249-01-001.MFB	55	Indian	M	Paraganglioma 36y
CGS0250-01-001.CD	56	Chinese	F	Thyroid ca 50y
CGS0306-01-001.TBC	57	Chinese	F	Pheochromocytoma 53y
CGS0309-01-001.TAL	58	Chinese	F	Breast ca 58y
CGS0312-01-001.KBO	59	Malay	F	Thyroid ca 24y
CGS0313-01-001.LCY	60	Chinese	F	Breast ca 41y
CGS0316-01-001.TGL	61	Chinese	F	Breast ca 39y
CGS0317-01-001.TLW	62	Chinese	F	Breast ca 35y
CGS0320-01-001.KBT	63	Chinese	F	Ovarian ca 60y
CGS0322-01-001.QSC	64	Chinese	F	Colorectal ca 39y
CGS0325-01-001.NHP	65	Chinese	F	Breast ca 39y
CGS0330-01-001.NSA	66	Indian	F	Breast ca 55y, Ovarian ca 59y
CGS0331-01-001.NBM	67	Malay	F	Pheochromocytoma 24y
CGS0677-01-001.TSL	68	Chinese	F	Breast ca 30y
CGS0333-01-001.GSK	69	Chinese	F	Breast ca 56y
CGS0334-01-001.CYE	70	Chinese	F	Colorectal ca 43y
CGS0335-01-001.TSH	71	Chinese	F	Ovarian ca 53y
CGS0338-01-001.NAB	72	Malay	F	Ovarian ca 23y
CGS0340-01-001.LLH	73	Chinese	F	Ovarian ca 72y
CGS0341-01-001.NYE	74	Chinese	F	Ovarian ca 64y
CGS0343-01-001.ZBM	75	Malay	F	Breast ca 33y, ovarian ca, 59y, gastrointestinal stromal tumour 59y
CGS0345-01-001.COB	76	Chinese	F	Ovarian ca 54y
CGS0348-01-001.ABA	77	Malay	F	Colorectal ca 29y
CGS0559-01-001.TMF	78	Chinese	F	Ovarian ca 60y
CGS0359-01-001.YN	79	Chinese	F	Ovarian ca 43y
CGS0360-01-001.OLH	80	Chinese	F	Breast ca 36y
CGS0557-01-001.ACN	81	Chinese	F	Ovarian ca 62y
CGS0365-01-001.PHL	82	Chinese	F	Ovarian ca 49y
CGS0372-01-001.FPW	83	Chinese	F	Breast ca 54y
CGS0381-01-001.GSC	84	Chinese	M	Colorectal ca 76y
CGS0384-01-001.GGL	85	Chinese	F	Colorectal ca 60y
CGS0385-01-001.KYF	86	Chinese	F	Breast ca 42y
CGS0387-01-001.LSH	87	Chinese	F	Breast ca 31y
CGS0394-01-001.ASH	88	rabian/India	F	Breast ca 33y

CGS0396-01-001.WMY	89	Chinese	F	Breast ca 54y
CGS0398-01-001.DT	90	Indonesian	F	Breast ca 37y
CGS0400-01-001.NHP	91	Chinese	F	Pheochromocytoma 28y
CGS0404-01-001.MT	92	Indonesian	F	Breast ca 61y
CGS0801-01-001.NL	93	Chinese	F	Breast ca 29y
CGS0628-01-001.LCS	94	Chinese	F	Ovarian ca 38y
CGS0408-01-001.LSZ	95	Chinese	F	Breast ca 32y
CGS0686-01-001.OHL	96	Chinese	F	Ovarian ca 52y
CGS0410-01-001.MEM	97	hinese/India	F	Breast ca 41y
CGS0411-01-001.LLL	98	Chinese	F	Breast ca 44y
CGS0415-01-001.LKF	99	Chinese	F	Breast ca 35y
CGS0416-01-001.LSH	100	Chinese	F	Breast ca 28y
CGS0418-01-001.OSN	101	Chinese	F	Ovarian ca 49y
CGS0421-01-001.STH	102	Chinese	M	Colorectal ca 60y, prostate ca 61y
CGS0422-01-001.TLC	103	Chinese	F	Breast ca 33y
CGS0424-01-001.CX	104	Chinese	F	Melanoma 30y
CGS2443-01-001.WLM	105	Chinese	F	Pancreatic ca 65y, Pilocytic astrocytoma 51y
CGS1111-01-001.ZR	106	Chinese	F	Breast ca 38y
CGS1106-01-001.TWY	107	Chinese	F	Breast ca 25y
CGS1116-01-001.LHM	108	Chinese	F	Leukaemia 7y
CGS1138-01-001.CML	109	FILIPINO	F	Breast ca 39y
CGS1130-01-001.AH	110	Chinese	F	Breast ca 31y
CGS1129-01-001.TAA	111	Chinese	F	Ovarian ca 85y
CGS0706-01-001.TLM	112	Chinese	F	Breast ca 48y
CGS1005-01-001.SSL	113	Chinese	F	Thyroid ca 38y, gastrointestinal stromal tumour 28y
CGS0913-01-001.SA	114	rmese/Jew	F	Ovarian ca 40y
CGS1221-01-001.HBM	115	Malay	M	Colorectal ca 62y
CGS0806-01-001.CNW	116	Chinese	F	Breast ca 21y
CGS1121-01-001.LKY	117	Chinese	F	Breast ca 38y
CGS1147-01-001.TLN	118	Chinese	F	Breast ca 29y
CGS1163-01-001.GSN	119	Indonesian	F	Ovarian ca 47y
CGS1168-01-001.D	120	Indonesian	F	Breast ca 36y
CGS1172-01-001.HCH	121	Chinese	F	Ovarian ca 50y, Endometrial ca 50y
CGS1122-01-001.NSH	122	Chinese	F	Endometrial ca 51y
CGS1182-01-001.SMM	123	Chinese	F	Breast ca 34y
CGS1203-01-001.CRM	124	Filipino	F	Breast ca 36y
CGS1190-01-001.HER	125	Caucasian	M	Pheochromocytoma 48y
CGS0912-01-001.CPF	126	Chinese	F	Breast ca 38y
CGS1241-01-001.PGG	127	CAUCASIAN	F	Breast ca 37y
CGS1246-01-001.TS	128	Chinese	F	Breast ca 48y
CGS1242-01-001.CCC	129	Chinese	M	Colorectal ca 48y
CGS1259-01-001.TVM	130	Indian	F	Breast ca 51y
CGS1244-01-001.ABM	131	Malay	F	Breast ca 44y
CGS1267-01-002.YYS	132	Chinese	F	Breast ca 47y
CGS0232-01-001.MTB	133	Malay	M	Colorectal ca 59y, kidney ca 59y
CGS1298-01-001.ADH	134	Indonesian	F	Breast ca 38y
CGS1293-01-001.EYJ	135	Chinese	F	Breast ca 23y
CGS1305-01-001.CYP	136	Chinese	F	Ovarian ca 43y
CGS1321-01-001.KMP	137	Chinese	F	Breast ca 35y

CGS1284-01-001.CYY	138	Chinese	F	Breast ca 38y
CGS1288-01-001.LDG	139	Indian	F	Breast ca 36y
CGS1255-01-001.LSH	140	Chinese	F	Ovarian ca 54y
CGS1233-01-001.AHY	141	Chinese	F	Ovarian ca 62y
CGS1343-01-001.KHL	142	Chinese	F	Breast ca 45y
CGS1344-01-001.JLM	143	Chinese	F	Breast ca 50y
CGS1340-01-001.NHK	144	Chinese	F	Breast ca 39y
CGS1260-01-001.YK	145	Cambodian	M	Colorectal ca 40y
CGS1327-01-001.KSF	146	Chinese	F	Pituitary tumour 62y; MEN1
CGS1335-01-001.RBA	147	Malay	F	Ovarian ca 45y, Endometrial ca 45y
CGS1360-01-001.CSL	148	Chinese	F	Ovarian ca 47y
CGS1422-01-001.ZW	149	Chinese	F	Breast ca 34y
CGS1424-01-001.WKM	150	Caucasian	F	Breast ca 42y
CGS1429-01-001.SD	151	Cambodian	F	Breast ca 27y
CGS1139-01-001.OSH	152	Chinese	F	Breast ca 21y
CGS1456-01-001.DTX	153	Vietnamese	F	Breast ca 33y
CGS1458-01-001.MTA	154	UAE	F	Endometrial ca 39y
CGS1439-01-001.HCW	155	Chinese	F	Breast ca 53y
CGS1462-01-001.SPC	156	Chinese	M	Breast ca 60y, Lung cancer 56y
CGS1465-01-001.LKL	157	Chinese	M	Colorectal ca 39y
CGS1473-01-001.HA	158	Indian	M	Rhabdoid tumor 1y
CGS1469-01-001.KFN	159	Bangladesh	F	Breast ca 43y
CGS0760-01-001.SLH	160	Chinese	F	Colorectal ca 59y, Endometrial ca 59y
CGS1492-01-001.HSK	161	Chinese	F	Breast ca 61y
CGS1493-01-001.TKH	162	Chinese	F	Breast ca 58y
CGS1460-01-001.OLF	163	Chinese	F	Breast ca 48y
CGS0035-01-001.CHL	164	Chinese	F	Medulloblastoma 13y
CGS0577-01-001.WS	165	Chinese	F	Breast ca 35y
CGS0054-01-001.MYC	166	Chinese	F	Breast ca 50y
CGS0058-01-001.LHT	167	Chinese	F	Ovarian ca 55y
CGS1476-01-001.RH	168	Indonesian	F	Breast ca 43y
CGS1258-01-001.TGH	169	Chinese	F	Endometrial ca 56y
CGS0095-01-001.NCT	170	Chinese	F	Breast ca 50y
CGS0107-01-001.SG	171	Nepalese	F	Breast ca 42y
CGS0110-01-001.YEL	172	Chinese	F	Breast ca 36y
CGS2404-01-001.LTM	173	Vietnamese	F	Esophagus cancer 66y
CGS0082-01-001.DW	174	Chinese	F	Breast ca 29y
CGS0117-01-001.SBS	175	Malay	F	Breast ca 42y
CGS0120-01-001.LSH	176	Chinese	F	Breast ca 53y
CGS0100-01-001.MLY	177	Chinese	F	Breast ca 36y
CGS0062-01-001.FBA	178	Malay	F	Breast ca 60y, Ovarian ca 64y
CGS0135-01-001.SBS	179	Malay	F	Breast ca 32y
CGS1470-01-001.LGE	180	Chinese	F	Breast ca 64y, Ovarian ca 56y
CGS0155-01-001.SLW	181	Chinese	M	Desmoid fibromatosis 53y, Mesenteric fibromatosis 55y
CGS0154-01-001.WZM	182	Chinese	F	Malignant proliferating trichilemmal tumour 31y
CGS0166-01-001.NAL	183	Chinese	F	Ovarian ca 44y
CGS0205-01-001.TPS	184	Chinese	F	Endometrial ca 43y
CGS0143-01-001.OAC	185	Chinese	F	Ovarian ca 59y

CGS0173-01-001.LMJ	186	Filipino	F	Breast ca 37y
CGS0180-01-001.PNJ	187	Chinese	F	Breast ca 35y
CGS0182-01-001.LY	188	Chinese	F	Breast ca 48y
CGS0187-01-001.SDG	189	Indian	F	Breast ca 39y
CGS2242-01-001.FLP	190	Chinese	F	Oligocystic-macrocystic serous cystadenoma with neuroendocrine microadenoma 63y; Hyperparathyroidism, Hyperthyroidism
CGS0194-01-001.CHM	191	Chinese	F	Breast ca 29y
CGS0079-01-001.OPK	192	Chinese	F	Ovarian ca 63y
CGS0201-01-001.YSK	193	Chinese	F	Breast ca 59y, Nasopharyngeal carcinoma 49y
CGS0207-01-001.OMC	194	Chinese	F	Breast ca 54y
CGS0211-01-001.KSH	195	Chinese	F	Breast ca 39y
CGS0212-01-001.LJL	196	Chinese	F	Breast ca 29y
CGS0221-01-001.GMR	197	Filipino	F	Ovarian ca 49y
CGS0208-01-001.OHL	198	Chinese	F	Breast ca 41y
CGS0347-01-001.OSP	199	Chinese	F	Breast ca 34y; Right breast benign fibrocystic change and sclerosing adnosis, Right breast benign intraductal papilloma with adjacent small focus of low grade LCIS in surrounding breast
CGS0406-01-001.PPH	200	Chinese	F	Breast ca 43y
CGS0447-01-001.TR	201	Chinese	M	Left retinal capillary hemangioma 65y
CGS0455-01-001.CKL	202	Chinese	M	Prostate ca 71y
CGS0453-01-001.HBK	203	Malay	M	Colorectal ca 68y
CGS0460-01-001.LH	204	Indonesian	F	High grade serous primary peritoneal cancer 73y
CGS0468-01-001.CM	205	Chinese	F	Breast ca 31y
CGS0151-01-001.KOL	206	Chinese	F	Ovarian ca 54y
CGS0469-01-001.SNT	207	Chinese	F	No cancer 21y; Pancytopenia, hypocellular marrow with dysplasia in the erythroid series (though insufficient to be called MDS)
CGS0490-01-001.ACJ	208	Indian	F	Breast ca 41y
CGS0197-01-001.SYK	209	Chinese	M	Colorectal ca 62y
CGS0501-02-001.RBA	210	Malay	F	No cancer 63y; right eye myasthenia gravis (follow up with SNEC), ?IPMN
CGS0487-01-001.ANA	211	Malay	F	Breast ca 40y
CGS0437-01-001.OSK	212	Chinese	F	Breast ca 44y, Colorectal ca 40y
CGS0512-01-001.PMK	213	Chinese	F	Breast ca 35y
CGS0515-01-001.OW	214	Chinese	F	Paraganglioma 74y, thymoma 72
CGS0518-01-001.MEP	215	Chinese	F	Breast ca 44y
CGS0565-01-001.TWL	216	Chinese	F	Ovarian ca 31y
CGS0606-01-001.KAB	217	Malay	M	Lung adenocarcinoma 19y
CGS0522-01-001.HYY	218	Chinese	F	Thyroid ca 35y, Liposarcoma 35y, Glioblastoma multiforme 21y
CGS0741-01-001.IBA	219	Malay	M	Thymoma 39y, adrenal cortical carcinoma 48y
CGS0792-01-001.KLM	220	Chinese	F	Endometrial ca 61y
CGS0528-01-001.KSH	221	Chinese	F	Endometrial ca 63y
CGS0176-01-001.YY	222	Chinese	F	Breast ca 22y
CGS0804-02-001.FJ	223	Malay	M	No cancer 52y; Eccrine poroma
CGS0893-01-001.KCK	224	Chinese	F	Colorectal ca 70y, Endometrial ca 56y
CGS0483-01-001.PMF	225	Filipino	F	Breast ca 37y

CGS0078-03-001.GCG	226	Chinese	F	Breast ca 60y, Ovarian ca 40y
CGS0643-01-001.CX	227	Chinese	F	Breast ca 39y
CGS0989-01-001.CSK	228	Chinese	F	Ovarian ca 50y
CGS1038-01-001.CKF	229	Chinese	F	Breast ca 46y
CGS1059-01-001.LGP	230	Chinese	F	Colorectal ca 44y
CGS1079-01-001.LYL	231	Chinese	F	Breast ca 44y
CGS1080-01-001.NML	232	Indonesian	F	Breast ca 33y
CGS1504-01-001.TY	233	Chinese	F	Breast ca 35y
CGS1511-01-001.LKM	234	Chinese	F	Endometrial ca 55y
CGS1075-01-001.CKJ	235	Chinese	F	Ovarian ca 63y
CGS1527-01-001.YKL	236	Chinese	M	No cancer 60y; Multiple juvenile polyps, Multiple harmatomatous polyp (50-100)
CGS0132-01-001.LSY	237	Chinese	F	Breast ca 28y
CGS1551-01-001.PNX	238	Vietnamese	F	Malignant peripheral nerve sheath tumor (MPNST) 15y, Cerebello-pontine angle tumour (ATRT) 16y
CGS1491-01-001.TOL	239	Chinese	F	Breast ca 43y, melanoma 44y
CGS1534-01-001.MAN	240	Malay	M	Sarcoma 6y
CGS1576-01-001.TST	241	Chinese	F	Breast ca 48y, Adenocarcinoma-in-situ of the cervix 36y
CGS0525-01-001.IT	242	Chinese	F	Ovarian ca 41y, Endometrial ca 41y
CGS1587-01-001.HLH	243	Chinese	F	Breast ca 37y, Nasopharyngeal carcinoma 24y
CGS0572-01-001.CSY	244	Chinese	F	Ovarian ca 48y, Endometrial ca 48y
CGS1599-01-001.TOK	245	Chinese	F	Leukaemia 52y
CGS1602-01-001.TLH	246	Chinese	F	Breast ca 56y
CGS1618-01-001.NCS	247	Chinese	F	Breast ca 48y
CGS1594-01-001.HLW	248	Chinese	F	Breast ca 36y
CGS1625-01-001.BG	249	Mongolian	F	No cancer 21y; Aplastic anemia
CGS1629-01-001.CCW	250	Chinese	F	Breast ca 30y
CGS1505-01-001.NHS	251	Malay	F	Leukaemia 11y, sarcoma 11y
CGS1635-01-001.SYL	252	Chinese	F	Ovarian ca 54y
CGS1598-01-001.ZF	253	Chinese	F	Breast ca 48y
CGS0213-01-001.LAL	254	Chinese	M	Thyroid ca 44y
CGS1649-01-001.STS	255	Chinese	F	Breast ca 41y
CGS1655-01-001.LZS	256	Chinese	F	Endometrial ca 35y
CGS1659-01-001.SH	257	Cambodian	M	Desmoid fibromatosis 18y
CGS1663-01-001.LHC	258	Chinese	M	Pheochromocytoma 60y
CGS1666-01-001.TAC	259	Chinese	F	Breast ca 39y
CGS1677-01-001.TFS	260	Chinese	F	Breast ca 42y
CGS1676-01-001.LLG	261	Chinese	F	Pituitary tumour 27y
CGS1719-01-001.CYH	262	Chinese	F	Ovarian ca 67y
CGS1725-01-001.EW	263	Indonesian	F	Pancreatic ca 46y
CGS1688-02-002.RPS	264	Malay	F	Breast ca 62y
CGS1693-01-001.CBH	265	Chinese	F	Breast ca 45y
CGS1699-01-001.LYZ	266	Chinese	F	Thyroid ca 16y
CGS1736-01-001.JTW	267	Chinese	F	Breast ca 36y
CGS1737-01-001.LSP	268	Chinese	F	Ovarian ca 80y
CGS1760-01-001.PS	269	Indonesian	F	Breast ca 38y
CGS1761-01-001.CLJ	270	Chinese	F	Breast ca 45y

CGS1761-01-002.LJY	271	Chinese	F	Breast ca 39y; Sjogren syndrome, Alpha Thal Trait, Appendicectomy
CGS1763-01-001.AGK	272	Chinese	F	Breast ca 41y
CGS1762-01-001.TSC	273	Chinese	F	Breast ca 49y
CGS1770-01-001.KM	274	Chinese	F	Breast ca 36y
CGS1773-01-001.SLK	275	Chinese	F	Colorectal ca 67y
CGS1647-01-001.LJE	276	Chinese	F	Breast ca 49y
CGS1784-01-001.CSF	277	Chinese	F	Breast ca 38y, Cervical intraepithelial neoplasm 32y
CGS1706-01-001.TAL	278	Chinese	M	Kidney ca 52y
CGS1791-01-001.LGC	279	Chinese	F	Ovarian ca 55y
CGS1791-01-002.LGL	280	Chinese	F	Breast ca 57y
CGS1798-01-001.WSL	281	Chinese	F	Breast ca 35y
CGS1801-01-001.LSC	282	Chinese	F	Ovarian ca 59y
CGS1802-01-001.LMY	283	Chinese	F	Breast ca 35y
CGS1803-01-001.LSC	284	Chinese	F	Ovarian ca 41y
CGS1808-01-001.FBS	285	Malay	F	Breast ca 45y
CGS1811-01-001.THN	286	Chinese	F	Breast ca 48y
CGS1810-01-001.SKH	287	Chinese	F	Ovarian ca 19y
CGS1823-01-001.YWC	288	Chinese	F	Ovarian ca 30y
CGS1837-01-001.PCS	289	Chinese	M	Pancreatic ca 65y
CGS1844-01-001.CLS	290	ıcasian (Brit	F	Breast ca 52y
CGS0217-01-001.ZBA	291	Malay	F	Breast ca 52y
CGS1852-01-001.CBG	292	Chinese	F	Breast ca 43y
CGS1854-01-001.DCC	293	Filipino	M	Prostate ca 44y
CGS1851-01-001.CSH	294	Chinese	F	Breast ca 42y
CGS1443-01-001.THH	295	Chinese	F	Breast ca 50y, Pheochromocytoma 48y
CGS1868-01-001.TYT	296	Chinese	F	Breast ca 30y
CGS1884-01-001.LLK	297	Chinese	F	Breast ca 41y
CGS1862-01-001.SX	298	Chinese	F	Breast ca 50y, thyroid ca 50y
CGS1892-01-001.JK	299	Australian	F	Breast ca 39y
CGS1895-01-001.LCN	300	Chinese	F	Ovarian ca 53y
CGS1769-01-001.WLS	301	Chinese	F	Breast ca 49y
CGS1897-01-001.LXY	302	Chinese	F	Ovarian ca 51y, Endometrial ca 51y
CGS1915-01-001.HHB	303	Chinese	F	Breast ca 70y
CGS1902-01-001.NCP	304	Chinese	F	Breast ca 39y
CGS1918-01-001.QWC	305	Chinese	F	Breast ca 35y
CGS1927-01-001.KSW	306	Chinese	F	Breast ca 61y
CGS1923-01-001.WYL	307	Chinese	F	Ovarian ca 31y
CGS1928-01-001.CSL	308	Chinese	F	Breast ca 31y
CGS1937-01-001.LCI	309	Chinese	F	Ovarian ca 64y; Bicytopenia
CGS1912-01-001.TLL	310	Chinese	F	Breast ca 35y
CGS1938-01-001.TSK	311	Chinese	M	Thymic neuroendocrine tumor 57y
CGS1940-01-001.HL	312	Chinese	F	Breast ca 41y
CGS1825-01-001.LBL	313	Chinese	F	Endometrial ca 36y
CGS1843-01-001.LTF	314	Chinese	F	Breast ca 53y
CGS1962-01-001.LSC	315	Chinese	F	Ovarian ca 48y
CGS1964-01-001.TSL	316	Chinese	F	Ovarian ca 57y, Endometrial ca 57y
CGS2049-01-001.GYP	317	Chinese	F	Breast ca 32y
CGS1982-01-001.QMH	318	Chinese	F	Breast ca 35y

CGS1999-01-001.ETS	319	Chinese	F	Breast ca 56y, Lung cancer 52y
CGS2018-01-001.TLL	320	Chinese	F	Ovarian ca 46y
CGS2028-01-001.RBB	321	Malay	F	Ovarian ca 64y
CGS1708-01-001.CJA	322	Filipino	F	Breast ca 40y
CGS2036-01-001.TAK	323	Chinese	F	Breast ca 62y, sarcoma 62y
CGS2047-01-001.LSH	324	Chinese	F	Leukaemia 30y
CGS2040-01-001.SAY	325	Chinese	M	Pancreatic ca 64y
CGS2052-01-001.JC	326	Chinese	F	Breast ca 39y; Right phyllodes breast tumor
CGS1958-01-003.WKC	327	Chinese	M	Kidney ca 50y
CGS1958-01-002.NGN	328	Chinese	F	Breast ca 58y
CGS2067-01-001.QBL	329	Chinese	M	Pancreatic ca 62y
CGS1942-02-001.PMW	330	Chinese	M	No cancer 62y; Colon polyps (<10)
CGS2135-01-001.LWH	331	Chinese	F	Breast ca 41y
CGS2078-01-001.NBI	332	Chinese	F	Breast ca 64y
CGS2087-01-001.VLS	333	Indian	F	Ovarian ca 50y
CGS2092-01-001.OCT	334	Chinese	F	Endometrial ca 55y
CGS2098-01-001.HKW	335	Chinese	M	Prostate ca 63y
CGS2104-01-001.KHK	336	Chinese	M	Pancreatic ca 53y
CGS2110-01-001.YLC	337	Chinese	F	Breast ca 46y
CGS2119-01-001.TKJ	338	Chinese	F	Ovarian ca 57y
CGS2126-01-001.SSK	339	Chinese	F	Ovarian ca 58y
CGS2125-01-001.NR	340	Indian	F	Ovarian ca 38y
CGS2130-01-001.NLL	341	Chinese	F	Breast ca 60y, Ovarian ca 61y
CGS2142-01-001.HAL	342	Chinese	F	Ovarian ca 57y
CGS2140-01-001.HNH	343	Chinese	F	Breast ca 60y
CGS2166-01-001.TGH	344	Chinese	F	Pancreatic ca 65y
CGS2175-01-002.LBH	345	Chinese	F	Breast ca 49y
CGS2134-01-001.KHE	346	Chinese	F	Breast ca 40y
CGS1993-01-001.DPJ	347	Chinese	F	Breast ca 58y, Endometrial ca 52y
CGS2202-01-001.GSL	348	Chinese	F	Breast ca 37y
CGS2204-01-001.OCW	349	Chinese	F	Endometrial ca 53y
CGS2090-02-001.HKC	350	Chinese	F	Breast ca 63y
CGS2213-01-001.SNV	351	Indian	F	Posterior fossa ependymoma 1y, Thoracic neuroblastoma 1y, Diffuse midline glioma 14y, Spine tumor 13y
CGS2415-01-001.SWY	352	Chinese	F	Breast ca 38y
CGS2429-01-001.LSE	353	Chinese	F	Breast ca 69y
CGS2219-01-001.YMC	354	Chinese	F	Ovarian ca 56y
CGS2438-01-001.NNN	355	Burmese	F	Breast ca 46y
CGS2446-01-001.LCE	356	Chinese	F	Breast ca 44y
CGS2452-01-001.MMK	357	Burmese	F	Breast ca 40y
CGS2457-01-001.KGT	358	Chinese	F	Breast ca 64y
CGS2462-01-001.VP	359	Indian	F	Breast ca 36y, sarcoma 14y
CGS2463-01-001.ECT	360	German	F	Breast ca 44y
CGS4330-01-001.KYR	361	Chinese	F	No cancer 32y; Haemorrhoids
CGS2474-01-001.LLY	362	Chinese	F	Breast ca 48y
CGS2477-01-001.CGL	363	Chinese	F	Ovarian ca 73y
CGS2476-01-001.WJ	364	Chinese	F	Breast ca 50y, Colorectal ca 50y
CGS2481-01-001.M	365	Chinese	F	Breast ca 66y
CGS2872-01-001.CHM	366	Chinese	F	Sarcoma 15y

CGS2880-01-001.NSC	367	Chinese	F	Ovarian ca 57y
CGS2270-01-001.VLS	368	Chinese	F	Breast ca 25y
CGS2911-01-001.NBM	369	Malay	F	Breast ca 48y, desmoid fibromatosis 40y
CGS2917-01-001.TTK	370	Chinese	F	Ovarian ca 70y
CGS2955-01-001.NRM	371	French	M	Kidney ca 40y
CGS2975-01-001.TLT	372	Chinese	F	Breast ca 48y
CGS2969-01-001.SBS	373	Malay	F	Breast ca 33y
CGS2897-02-002.OBK	374	Chinese	F	No cancer 58y; Duplex kidney, Recurrent UTIST VAB of right breast central outer microcalcifications
CGS3110-01-001.LYM	375	Chinese	F	Breast ca 43y; Ovarian cysts
CGS3176-01-001.CSK	376	Chinese	F	Ovarian ca 59y
CGS3096-01-001.CLC	377	Chinese	F	Ovarian ca 42y, Endometrial ca 38y; Left breast friboadenoma, Multinodular goiter, Thyroid nodule
CGS3201-01-001.KHC	378	Chinese	F	Breast ca 56y
CGS2006-01-001.YMM	379	Chinese	F	Ovarian ca 57y
CGS3001-01-001.QPC	380	Chinese	F	Ovarian ca 57y
CGS3218-01-001.CKW	381	Chinese	F	Sarcoma 32y; Diffuse goiter, Desmoid tumor
CGS3240-01-001.SA	382	Chinese	M	Sarcoma 9y
CGS3251-01-001.STS	383	Chinese	M	Gastric ca 65y; Plantar Fibromatosis
CGS3103-01-001.KYC	384	Chinese	F	Breast ca 50y
CGS3106-01-001.CSC	385	Chinese	F	Breast ca 68y, Colorectal ca 72y, Endometrial ca 52y; Anemia, Acute kidney injury
CGS3267-01-001.OBY	386	Chinese	F	Ovarian ca 35y; Benign breast microcalcifications
CGS3043-01-001.BS	387	Sikh	M	Gastric ca 54y
CGS3224-01-001.LC	388	Burmese	F	Breast ca 35y; Hole in heart
CGS3263-01-001.YWS	389	Chinese	F	Ovarian ca 48y; Deep vein thrombosis, Hypothyroidism, Fibroadenoma (left breast)
CGS3280-01-001.CYK	390	Chinese	F	Breast ca 43y
CGS3136-01-002.FST	391	Chinese	F	No cancer 51y; Hyperthyroidism, Iron deficient anemia, Uterine fibroids
CGS3294-01-001.TS	392	Chinese	F	No cancer 59y; Breast lump
CGS3312-01-001.PCH	393	Chinese	M	Prostate ca 71y
CGS3070-03-001.RBD	394	Indian	F	No cancer 44y; Connective tissue disorder (inflammatory arthritis), Arthralgia
CGS3070-03-002.SBD	395	Indian	F	No cancer 47y; Ovarian cysts, Fibroids
CGS3353-01-001.QSC	396	Chinese	F	Breast ca 58y
CGS3357-01-001.AFS	397	Caucasian	F	Wilms tumor 8y
CGS3361-01-001.LSK	398	Chinese	F	Breast ca 58y, Ovarian ca 58y; Endometrial Polyp
CGS3369-01-001.LCY	399	Chinese	F	Ovarian ca 35y
CGS2170-01-001.YLK	400	Chinese	F	No cancer 46y; Primary hyperparathyroidism with hyperfunctioning parathyroid superior and posterior to left upper pole of parathyroid
CGS3379-01-001.AER	401	Filipino	F	Breast ca 35y
CGS3363-01-001.GCK	402	Chinese	F	Pancreatic ca 65y
CGS3388-01-001.TCL	403	Chinese	M	Prostate ca 76, Giant cell tumour of right femur 66y

CGS3409-01-001.NS	404	Thai	F	Lung adenocarcinoma 50y
CGS3393-01-001.SBS	405	Malay	F	Breast ca 29y
CGS3394-01-001.SSE	406	Chinese	F	Ovarian ca 71y; Ascending colon polyp - tubular adenoma with low-grade dysplasia-
CGS3396-01-001.LHC	407	Chinese	F	Pancreatic ca 59y
CGS3398-01-001.HLC	408	Chinese	F	Primary Peritoneal Cancer 72y
CGS3401-01-001.OHM	409	Chinese	F	Ovarian ca 48y
CGS3345-01-001.CCX	410	Chinese	F	Sarcoma 12y, Secondary malignant neoplasm (Bones) 14y
CGS3351-01-001.LHM	411	Chinese	F	Breast ca 26y
CGS3410-01-001.HJD	412	Indian	F	No cancer 62y; Left neck mass, Asymmetric tonsils, Right Thyroid nodule; Consistent with benign follicular nodule
CGS2999-01-001.LCH	413	Chinese	M	Prostate ca 68y
CGS3433-01-001.OBS	414	Chinese	F	Breast ca 36y
CGS3443-01-001.TPK	415	Chinese	F	Breast ca 63y
CGS3466-01-001.YHM	416	Chinese	F	Numerous small lung cysts suspected for lymphangioleiomyomatosis 36y
CGS3463-01-001.LCY	417	Chinese	F	Breast ca 52y
CGS3469-01-001.WCL	418	Chinese	F	Breast ca 59y, Ovarian ca 70y
CGS3522-01-001.OHM	419	Chinese	M	Pancreatic ca 32y
CGS3487-01-001.TSD	420	Chinese	F	Breast ca 52y, Left chest wall lesion (Oligometastatic disease) 51y
CGS3489-01-001.TCL	421	Chinese	M	Pituitary tumour 64y; Hyperparathyroidism-hypercalcemia, Thyroid nodules
CGS3501-01-001.TKK	422	Chinese	M	Prostate ca 69y; Ischemic heart disease
CGS3182-01-001.YEN	423	x	F	Breast ca 30y; Benign thyroid nodules
CGS3343-01-002.LCL	424	Chinese	F	Breast ca 43y
CGS3511-01-001.OHE	425	Chinese	F	Endometrial ca 55y
CGS3513-01-005.THP	426	Chinese	F	No cancer 56y; L paraovarian cyst
CGS3513-01-006.THN	427	Chinese	M	No cancer 67y; Heart problems (underwent arterial constriction)
CGS3287-01-001.MDB	428	Malay	M	No cancer 33y; Pulmonary alveolar proteinosis
CGS3530-01-001.LPC	429	Chinese	M	Prostate ca 66y
CGS0045-01-001.SS	430	Indian	F	Breast ca 39y
CGS3527-01-001.TSL	431	Chinese	F	Breast ca 47y
CGS3088-01-002.NLE	432	Chinese	F	No cancer 56y
CGS3547-01-001.LPK	433	Caucasian	F	No cancer 38y; Multiple polyps (90-100 adenoma and hyperplastic) polyps
CGS2008-02-001.KHK	434	Chinese	F	Colorectal ca 46y
CGS3574-01-001.JLW	435	Indian	M	Pancreatic ca 56y; Minor CAD, Hyperlipidaemia, Right branch retinal artery occlusion
CGS3438-01-001.TCL	436	Chinese	M	Colorectal ca 45y
CGS3569-01-001.KGM	437	Chinese	F	Breast ca 44y
CGS3580-01-001.LCH	438	Chinese	M	No cancer 64y; Multiple polyps
CGS3585-01-001.CPG	439	Chinese	F	No cancer 41y; Uterine Leimyomas, Dearmal lumps (Forearm)
CGS3586-01-001.PFC	440	Chinese	F	Breast ca 60y
CGS3599-01-001.TCK	441	Chinese	F	Breast ca 66y; Bilateral cataracts
CGS3596-01-001.OKY	442	Chinese	F	Colorectal ca 40y; Chiari malformation

CGS3613-01-001.CVC	443	Chinese	F	Breast ca 39y
CGS3623-01-001.AAB	444	Malay	M	Rectalsigmoid cancer 44y
CGS3659-01-001.SNA	445	Malay	F	Breast ca 30y
CGS3677-01-001.THC	446	Chinese	M	Pancreatic ca 60y
CGS3678-01-001.WSC	447	Chinese	F	Breast ca 80y, Ovarian ca 80y
CGS3686-01-001.SHP	448	Chinese	M	Adrenal cortical carcinoma 50y; Diabetes
CGS3690-01-001.LHP	449	Chinese	F	Breast ca 57y; Osteopenia
CGS3672-01-001.HJX	450	Chinese	F	Breast ca 35y
CGS3733-01-001.PSE	451	Chinese	F	Breast ca 43y; Ovarian cysts, ?Cognitive impairment, ?Subclinical hypothyroidism
CGS3727-01-001.PJW	452	Chinese	M	Spinal plexiform neurofibroma 33y; LV hypertrophy, Cafe au lait spots
CGS3730-01-001.QNH	453	Chinese	F	Breast ca 62y, Endometrial ca 66y; Hep B carrier
CGS3738-01-001.KBE	454	Chinese	F	Breast ca 46y
CGS3734-01-001.OLH	455	Chinese	F	Colorectal ca 51y
CGS3739-01-001.LSY	456	Chinese	F	Breast ca 38y
CGS3748-01-001.JGP	457	Chinese	F	Ovarian ca 66y
CGS3751-01-001.CSN	458	Chinese	F	Breast ca 45y
CGS3735-01-001.WCK	459	Chinese	F	Pheochromocytoma 31y
CGS3756-01-001.LJH	460	Chinese	F	Kidney ca 24y; Lung nodules (suggestive of mets disease)
CGS3758-01-001.WPB	461	Malay	F	Breast ca 49y
CGS3788-01-001.AB	462	Chinese	F	Breast ca 57y
CGS3839-01-001.KBK	463	Malay	F	Ovarian ca 54y; High frequency SHNL (Sensoneural hearing loss), Ovarian cysts (Benign), Fibroadenoma
CGS3835-01-001.TLK	464	Chinese	F	Ovarian ca 52y
CGS3837-01-001.NBH	465	Chinese	F	Ovarian ca 66y; total thyroidectomy for MNG
CGS3807-01-001.CTP	466	Chinese	F	Endometrial ca 59y; Grave's hyperthyroidism, ? Had vaginal lump s/p I&D
CGS3810-01-001.SKH	467	Chinese	F	No cancer 55y; Left breast nodules - benign, Graves` disease (overproduction of thyroid hormones), Hypothyroidism
CGS3679-01-001.SIW	468	Chinese	F	Breast ca 48y; See comments (multiple adenoma/polyp)
CGS3817-01-001.HYM	469	Chinese	M	Prostate ca 65y; Chronic kidney disease, Presumptive IHD
CGS3752-01-004.CSL	470	Chinese	F	No cancer 28y; Polycystic ovaries, Fibroids
CGS3752-02-001.CKG	471	Chinese	M	No cancer 65y; Arthropathy
CGS3858-03-003.NKB	472	Malay	F	No cancer 9y; Bilateral severe to moderate hearing loss
CGS3873-01-001.SYC	473	Chinese	M	Pancreatic ca 38y
CGS3869-01-001.CM	474	Chinese	F	Ovarian ca 50y
CGS3886-01-001.ZBA	475	Malay	F	Breast ca 48y, Langerhans cell histiocytosis age unknown; Endometriosis, Chronic cholecystitis, R breast fibrocystic change and fibroadenoma
CGS3732-01-001.GF	476	Caucasian	M	Left intraventricular high-grade glioma 11y; High-functioning autistic spectrum disorder
CGS3829-01-001.LKK	477	Chinese	M	Pheochromocytoma 48y ; Severe debilitating anxiety/depression disorder

CGS3305-01-001.LYC	478	Chinese	M	Colorectal ca 53y
CGS3904-01-001.AMS	479	Chinese	F	Breast ca 40y
CGS3915-01-001.SKS	480	Chinese	M	Pancreatic ca 45y
CGS3931-01-001.JLL	481	Chinese	F	Ovarian ca 48y; Fibroid uterus
CGS3928-01-001.TLK	482	Chinese	F	Breast ca 68y; Follicular adenoma in the background of multinodular goitre
CGS3936-01-001.FKG	483	Eurasian	F	Breast ca 52y
CGS3939-01-001.CSH	484	Chinese	F	Breast ca 53y
CGS3956-01-001.ZY	485	Chinese	M	Colorectal ca 62y, prostate ca 66y
CGS3965-01-001.CYA	486	Chinese	F	Pancreatic ca 61y
CGS3976-01-001.KKH	487	Chinese	M	Renal cell carcinoma 41y
CGS3752-01-005.LJ	488	Chinese	F	No cancer 36y; Meningioma
CGS3991-01-001.NPE	489	Chinese	F	Breast ca 34y
CGS4000-01-001.WKW	490	Chinese	M	Metastatic cholangiocarcinoma 57y
CGS3997-01-001.ASP	491	Chinese	F	Breast ca 53y
CGS4018-01-001.SH	492	Burmese	F	Breast ca 30y
CGS4017-01-001.SBS	493	Indonesian	F	Breast ca 53y
CGS4021-01-001.SCS	494	Chinese	M	Breast ca 51y
CGS4027-01-001.ACA	495	Filipino	F	Breast ca 31y; Suspicious left breast nodule
CGS4046-01-001.KBH	496	Chinese	F	Breast ca 37y
CGS4055-01-001.FJ	497	Chinese	M	Thymic NET 38y
CGS4071-01-001.SKG	498	Chinese	F	Breast ca 58y; Uterine fibroids
CGS4075-01-001.LFM	499	Chinese	F	Colorectal ca 69y
CGS4083-01-001.TXY	500	Chinese	F	No cancer 26y; Right groin cyst, Thalassemia minor
CGS4026-02-001.YSC	501	Chinese	F	No cancer 69y; Uterine fibroids
CGS4097-01-001.LCF	502	Chinese	F	Breast ca 54y
CGS4095-01-001.NKT	503	Chinese	F	Gastric ca 70y, gastrointestinal stromal tumour 70y, Multiple hyperplastic polyps, some with dysplasia 70y
CGS4107-01-001.KKD	504	Indian	F	Breast ca 32y; Hypothyroidism, Turner's syndrome
CGS4064-01-001.JBM	505	Malay	M	Prostate ca 67y
CGS4146-01-001.AGK	506	Chinese	F	Breast ca 51y
CGS4149-01-001.CLC	507	Chinese	F	Ovarian ca 51y
CGS4150-01-001.RSV	508	Indian	M	No cancer 53y; Interstitial lung disease (autoimmune)
CGS4108-01-001.SSN	509	Chinese	F	No cancer 65y; TIA, AF, Colonic polyps
CGS4197-01-001.NVB	510	Indian	M	Thyroid ca 36y
CGS4199-01-001.LKM	511	Chinese	F	Adrenal cortical carcinoma 77y, Olfractory Neuroblastoma 77y, Right lung adenocarcinoma 77y, Right Renal Cell Carcinoma 77y
CGS4198-01-001.MUA	512	Filipino	F	Breast ca 48y; Endometrial polyp
CGS4226-01-001.LMC	513	Chinese	F	Endometrial ca 50y
CGS4219-01-001.SBS	514	Malay	F	Breast ca 43y
CGS4229-01-001.RTS	515	Chinese	F	Breast ca 39y
CGS4228-01-001.DBD	516	Indian	F	Breast ca 38y
CGS4240-01-001.SI	517	Indian	F	Endometrial ca 63y, pancreatic ca 64y; DM - HbA1c 8.7%, Right CN 6 palsy, Photosensitive dermatitis

CGS4250-01-001.GSK	518	Chinese	F	Breast ca 52y
CGS4252-01-001.LYB	519	Chinese	M	Paraganglioma 61y ; PNET
CGS4248-01-001.OLB	520	Chinese	F	Breast ca 59y, Colorectal ca 59y
CGS4280-01-001.YJJ	521	Chinese	M	Bilateral vestibular schwanomma (acoustic neuromas) 28y; Right lacrimal gland mass, Skin lesions
CGS4286-01-001.LHH	522	Chinese	F	No cancer 48y; R breast lump - likely benign
CGS4289-01-001.HMA	523	Chinese	F	Breast ca 38y
CGS4309-01-001.IRH	524	Caucasian	M	Colorectal ca 71y, prostate ca 75y, melanoma 85y; Paget's disease, Pseudophakia, Fuchs corneal dystrophy
CGS4326-01-001.SCC	525	Chinese	F	Pancreatic ca 53y; R common peroneal neuropathy likely compressive, COPD
CGS4331-01-001.SSL	526	Chinese	F	Endometrial ca 44y; Hyperthyroidism
CGS3113-03-005.GZM	527	Chinese	F	No cancer 34y; Polycystic ovary syndrome
CGS4341-01-001.NLJ	528	Chinese	F	Breast ca 58y
CGS4337-01-001.DPJ	529	Caucasian	M	Prostate ca 68y
CGS4368-01-001.OKL	530	Chinese	F	Breast ca 56y, synchronous metastatic NSCLC adenocarcinoma 56y; Hypothyroidism
CGS4379-01-001.MTB	531	Malay	M	No cancer 34y; Non-erosive gastritis, nodule mucosa terminal ileum with small ulcer
CGS4389-01-001.LQH	532	Chinese	F	No cancer 36y; Weight mgmt program (sleep apnea), chronic urticaria/ symptomatic dermographism
CGS4399-01-001.SYF	533	Chinese	F	Breast ca 59y; Left total knee replacement, Ovarian cystectomy and myomectomy
CGS4396-01-001.CS	534	Chinese	F	Breast ca 33y
CGS4392-01-001.CSF	535	Chinese	F	Breast ca 68y
CGS2895-01-001.LCK	536	Chinese	F	Breast ca 66y
CGS2044-01-001.LCW	537	Chinese	F	Colorectal ca 70y
CGS1188-01-001.CKY	538	Chinese	F	Breast ca 63y
CGS3658-01-001.CSC	539	Chinese	F	Pancreatic ca 70y
CGS0097-01-001.YM	540	Chinese	F	Ovarian ca 54y
CGS1237-01-001.SPB	541	Chinese	M	Colorectal ca 74y
CGS2418-01-001.ARB	542	Malay	M	Prostate ca 64y
CGS3885-01-001.LYH	543	Chinese	F	Ovarian ca 72y; Severe L4/5 spinal canal stenosis with lumbar spondylolisthesis
CGS0449-01-001.KCK	544	Chinese	M	Prostate ca 65y
CGS3317-01-001.GLL	545	Chinese	F	Breast ca 55y; Endocervical polyp, Non-specific right lung subcm lung nodules
CGS3781-01-001.JOA	546	Chinese	F	Ovarian ca 61y
CGS0564-01-001.ABA	547	Malay	F	Ovarian ca 53y
CGS3020-01-001.LYH	548	Chinese	F	Breast ca 49y
CGS2019-01-001.HKM	549	Chinese	F	Ovarian ca 55y
CGS3380-01-002.GML	550	Chinese	F	Breast ca 55y
CGS3612-01-001.LYK	551	Chinese	F	Breast ca 54y; Rheumatoid arthritis
CGS0109-01-001.KSN	552	Chinese	F	Breast ca 52y
CGS2139-01-001.TTH	553	Chinese	F	Ovarian ca 51y
CGS1816-01-001.CI	554	Chinese	F	Endometrial ca 51y
CGS2993-01-001.MVA	555	Eurasian	F	Ovarian ca 57y

CGS3462-01-001.CTS	556	Chinese	M	Prostate ca 61y
CGS1670-01-001.SMM	557	Indian	F	Ovarian ca 81y
CGS0504-01-001.TLH	558	Chinese	F	Breast ca 55y
CGS1269-01-001.SLK	559	Burmease	F	Ovarian ca 47y
CGS3872-01-001.KTE	560	Chinese	F	Thyroid ca 51y
CGS0574-01-001.Sz	561	Indian	F	Breast ca 56y
CGS1286-01-001.GCW	562	Chinese	F	Breast ca 45y
CGS0578-01-001.KKK	563	Chinese	F	Breast ca 42y
CGS0579-01-001.APL	564	Chinese	F	Breast ca 39y
CGS0580-01-001.LYL	565	Chinese	F	Breast ca 31y
CGS1525-01-001.SM	566	Thai	F	Breast ca 42y
CGS3060-01-001.ESL	567	Chinese	F	Breast ca 42y
CGS3975-01-001.KSL	568	Chinese	F	Breast ca 43y
CGS1287-01-001.ASM	569	Chinese	F	Breast ca 37y
CGS0584-01-001.GGH	570	Chinese	F	Breast ca 36y
CGS3740-01-001.RLL	571	Chinese	F	No cancer 38y; Breast lumps and fibroids
CGS3039-01-001.LYH	572	Chinese	F	Breast ca 36y
CGS1428-01-001.AJP	573	Indian	F	Breast ca 33y
CGS4086-01-001.WTH	574	Chinese	F	Breast ca 29y
CGS0553-01-001.SJH	575	Chinese	M	No cancer 19y; Parathyroid adenoma
CGS2996-01-001.LKL	576	Chinese	F	Prostate ca 62y
CGS1772-01-001.APB	577	Indian	F	Breast ca 65y, Colorectal ca 68y
CGS3600-01-001.WPS	578	Chinese	F	Breast ca 67y
CGS4402-01-001.WJ	579	Chinese	F	Breast ca 69y, Ovarian ca 68y
CGS0594-01-001.CSE	580	Chinese	F	Breast ca 66y, Ovarian ca 72y
CGS1630-01-001.CPG	581	Chinese	F	Breast ca 53y
CGS3884-01-001.LK	582	Chinese	M	Prostate ca 68y
CGS2414-01-001.SPY	583	Chinese	M	Pancreatic ca 63y
CGS2269-01-001.LSK	584	Chinese	F	Pancreatic ca 63y
CGS2271-01-001.SSC	585	Chinese	F	No cancer 57y; Retinal capilary hemangiom - R eye
CGS3919-01-001.NCM	586	Chinese	F	Pheochromocytoma 63y ; Hep B carrier
CGS1971-01-001.TCL	587	Chinese	F	Breast ca 60y
CGS3821-01-001.CPK	588	Chinese	F	Thyroid ca 60y, pancreatic ca 61y
CGS0600-01-001.PPS	589	Chinese	F	Endometrial ca 54y
CGS3452-01-001.HAS	590	Chinese	M	Prostate ca 62y
CGS0605-01-001.GJE	591	Chinese	F	Urothelial carcinoma 53
CGS3238-01-001.CPG	592	Chinese	F	Breast ca 56y; Endometriotic Cyst
CGS0126-01-001.STS	593	Chinese	F	Colorectal ca 39y, Endometrial ca 54y
CGS0607-01-001.KTT	594	Chinese	M	Colorectal ca 49y
CGS0608-01-001.LLS	595	Chinese	M	Haemangloblastoma 51y
CGS0614-01-001.TWC	596	Chinese	F	Ovarian ca 60y
CGS4239-01-001.KKF	597	Chinese	F	Breast ca 63y; Hep B carrier
CGS2167-01-001.SJB	598	Indian	F	Breast ca 61y
CGS4190-01-001.WP	599	Chinese	M	Gastric ca 57y
CGS3112-01-001.MSB	600	Malay	M	Leukaemia 51y ; Anemia, NSTEMI
CGS1175-01-001.WTC	601	Chinese	F	Ovarian ca 45y
CGS4296-01-001.TPC	602	Chinese	F	Endometrial ca 52y
CGS2106-01-001.RAN	603	Filipino	F	Ovarian ca 49y
CGS3633-01-001.FSP	604	Chinese	F	Breast ca 49y

CGS1158-01-001.JJ	605	Chinese	F	Breast ca 38y
CGS2983-01-001.LSI	606	Chinese	F	Breast ca 47y
CGS3308-01-001.SR	607	Chinese	F	Breast ca 48y; Benign breast tissue with sclerosis adenosis (left)
CGS2881-01-001.HMK	608	Chinese	F	Ovarian ca 47y
CGS1148-01-001.NKS	609	Chinese	M	Melanoma 34y
CGS0624-01-001.CMR	610	Chinese	F	No cancer 41y; Benign right vestibular schwannoma
CGS2349-01-001.IV	611	Sri Lankan	F	Breast ca 38y
CGS0627-01-001.WQ	612	Chinese	F	Gastric ca 41y
CGS2471-01-001.KBL	613	Chinese	F	Ovarian ca 40y
CGS2422-01-001.SSX	614	Chinese	F	Ovarian ca 32y
CGS3536-01-001.KSH	615	Chinese	F	Ovarian ca 40y, Low grade appendiceal mucinous neoplasm 40y
CGS0631-01-001.RKJ	616	Caucasian	F	Melanoma 28y
CGS0175-01-001.HSY	617	Chinese	F	Breast ca 36y
CGS2379-01-001.KCL	618	Chinese	F	Breast ca 28y
CGS3063-01-001.CJ	619	Chinese	F	Breast ca 34y
CGS4294-01-001.ERL	620	Chinese	F	No cancer 35y; Severe endometriosis, Tubo ovarian abscess IR drainage, Mild chronic gastritis
CGS3674-01-001.LKT	621	Chinese	M	Prostate ca 68y; Hemangioma, Liver and biliary passages - benign neoplasm, Oligodendroglioma, Left cavernous meningioma
CGS4377-01-001.LBL	622	Chinese	M	Pancreatic ca 65y; NASH liver cirrhosis
CGS3850-01-001.KSG	623	Chinese	M	Prostate ca 75y; Hep B carrier
CGS4106-01-001.OSM	624	Chinese	M	Prostate ca 66y; Bilateral lumbosacral radiculopathy both lower limbs, Osteoporosis, DM
CGS2102-01-001.PJM	625	Chinese	F	No cancer 68y; Right eye retinal angioma
CGS3062-01-001.WLJ	626	Chinese	F	Breast ca 68y
CGS3171-01-001.LMK	627	Chinese	F	Breast ca 67y, pancreatic ca 76y
CGS4257-01-001.NKL	628	Chinese	F	Ovarian ca 65y
CGS3334-01-001.NMK	629	Chinese	F	Breast ca 62y; Chronic lacunar infarcts, Right myringoplasty, Obstructive Sleep Apnea
CGS1501-01-001.NSG	630	Chinese	F	Breast ca 59y
CGS3935-01-001.KLK	631	Chinese	F	Breast ca 61y
CGS0466-01-001.NST	632	Chinese	F	Endometrial ca 56y
CGS0454-01-001.WCH	633	Chinese	F	Breast ca 51y
CGS0650-01-001.DCL	634	Chinese	F	Ovarian ca 51y
CGS0651-01-001.OSC	635	Chinese	F	Breast ca 49y, Ovarian ca 52y
CGS1132-01-001.LAL	636	Chinese	F	Breast ca 53y
CGS3428-01-001.TCM	637	Chinese	M	Pancreatic ca 55y
CGS3058-01-001.LKK	638	Chinese	F	Breast ca 45y; Neurofibroma
CGS0655-01-001.YYW	639	Chinese	F	Breast ca 67y
CGS0657-01-001.BPJ	640	Chinese	F	Ovarian ca 45y
CGS0658-01-001.LGK	641	Chinese	F	Breast ca 38y
CGS1483-01-001.GLH	642	Chinese	F	Ovarian ca 47y
CGS2217-01-001.CY	643	Chinese	M	Prostate ca 78y
CGS3871-01-001.TSK	644	Chinese	F	Breast ca 45y

CGS0659-01-001.PGN	645	Chinese	F	Ovarian ca 74y
CGS1299-01-001.SAH	646	Chinese	F	Ovarian ca 57y
CGS3809-01-001.KDM	647	Chinese	F	Ovarian ca 63y; Trigger finger (left middle), right wrist metacarpal bossing, cervical spondylosis, Haemorrhoids, Osteopenia
CGS3797-01-001.CLF	648	Chinese	F	Ovarian ca 55y
CGS0373-01-001.WL	649	Chinese	F	Ovarian ca 53y
CGS0664-01-001.AMW	650	Caucasian	F	Breast ca 48y
CGS0813-01-001.ORS	651	Chinese	F	Ovarian ca 60y
CGS1744-01-001.GV	652	Indian	F	Breast ca 50y
CGS0669-01-001.PCY	653	Chinese	F	Breast ca 37y; endometrial polyp ~2cm arising from right lateral wall, RIGHT HEMI-THYROID: Adenomatous nodule
CGS1402-01-001.TLL	654	Chinese	F	No cancer 40y; Pituitary microadenoma
CGS1181-01-001.BLM	655	Filipino	F	Breast ca 38y
CGS0671-01-001.TSH	656	Chinese	F	Breast ca 40y
CGS2152-01-001.CAC	657	Chinese	F	Breast ca 40y
CGS3114-01-001.NBT	658	Chinese	M	Kidney ca 40y
CGS3244-01-001.SCF	659	Chinese	F	Breast ca 40y; Nephrotic syndrome, Thyroid cyst and Multinodular goiter
CGS0676-01-001.SBS	660	Malay	F	Breast ca 36y
CGS0499-01-001.CYL	661	Chinese	F	Breast ca 38y
CGS4014-01-001.SSJ	662	Chinese	F	Breast ca 39y
CGS4304-01-001.TCH	663	Chinese	M	No cancer 38y; Subfertility and azoospermia
CGS1444-01-001.LKS	664	Chinese	F	Breast ca 34y
CGS0681-01-001.MTT	665	Chinese	F	Ovarian ca 29y, Endometrial ca 29y
CGS3150-01-001.LYC	666	Chinese	F	No cancer 34y; Breast cysts
CGS0682-01-001.LTJ	667	Chinese	M	Gastric ca 26y
CGS0683-01-001.LGA	668	Chinese	M	Gastric ca 23y
CGS0684-01-001.VTS	669	Chinese	F	Pancreatic ca 19y, pituitary tumour, age unknown
CGS1228-01-001.OSK	670	Chinese	F	Endometrial ca 65y
CGS3764-01-001.LMY	671	Chinese	F	Ovarian ca 70y; Osteoarthritis, Renal cyst
CGS3514-01-001.WPN	672	Chinese	F	Breast ca 71y, Ovarian ca 64y
CGS4084-01-001.OBT	673	Chinese	M	Colorectal ca 66y, prostate ca 67y
CGS3780-01-001.BSM	674	Chinese	F	Ovarian ca 68y
CGS3203-01-001.LAH	675	Chinese	F	Endometrial ca 67y; Rectal bleeding
CGS3366-01-001.YYL	676	Chinese	F	Breast ca 46y, Lung adenocarcinoma 68y
CGS1095-01-001.KBD	677	PAKISTANI	F	Breast ca 69y
CGS4118-01-001.TKH	678	Chinese	M	Prostate ca 79y; Stage 3 CKD, Gout, BGIT 2' duodenal ulcer
CGS1441-01-001.CLY	679	Chinese	F	Breast ca 63y, Ovarian ca 55y
CGS1351-01-001.TSM	680	Chinese	F	Ovarian ca 59y
CGS3765-01-001.LAL	681	Chinese	F	Ovarian ca 71y
CGS4277-01-001.LCP	682	Chinese	F	Ovarian ca 70y
CGS4052-01-001.AI	683	Indian	F	Breast ca 65y, Endometrial ca 65y, gastrointestinal stromal tumour 65y
CGS3048-01-001.LMH	684	Chinese	F	Pancreatic ca 63y
CGS1006-01-001.RBH	685	Malay	F	Ovarian ca 57y
CGS3521-01-001.OSK	686	Chinese	F	Breast ca 45y

CGS2221-01-001.GJW	687	Chinese	F	Ovarian ca 57y
CGS2293-01-001.LCF	688	Chinese	M	Kidney ca 54y
CGS3408-01-001.SGH	689	Chinese	F	Breast ca 55y
CGS1219-01-001.THE	690	Chinese	F	Breast ca 49y
CGS0712-01-001.NAB	691	Malay	F	Breast ca 38y
CGS3223-01-001.SSM	692	Chinese	F	Ovarian ca 47y
CGS0716-01-001.HBA	693	Malay	F	Breast ca 41y
CGS0122-01-001.NMW	694	Chinese	F	Breast ca 41y
CGS3626-01-001.RBY	695	Malay	F	Breast ca 44y
CGS0530-01-001.ZL	696	Chinese	F	Sebaceous carcinoma 36y
CGS1789-01-001.SE	697	Chinese	F	Breast ca 42y
CGS1683-01-001.TMK	698	Chinese	F	Breast ca 41y
CGS1019-01-001.L	699	Chinese	F	Breast ca 39y
CGS0722-01-001.KJH	700	Chinese	F	Breast ca 32y
CGS0925-01-001.LMY	701	Chinese	F	Breast ca 38y
CGS3691-01-001.NBY	702	Malay	F	Breast ca 31y
CGS2954-01-001.YCK	703	Chinese	M	Prostate ca 74y
CGS0295-01-001.BAC	704	Eurasian	F	Ovarian ca 76y; Tubular Adenoma with Low Grade Dysplasia
CGS2094-01-001.HML	705	Chinese	F	Colorectal ca 75y
CGS0731-01-001.YAK	706	Chinese	M	Colorectal ca 78y
CGS0448-01-001.MSF	707	Chinese	F	Ovarian ca 61y
CGS1142-01-001.LBM	708	Malay	F	Breast ca 60y
CGS3768-01-001.TBL	709	Chinese	F	Colorectal ca 63y
CGS3233-01-001.DOS	710	Indian	F	No cancer 59y; Multiple miscarriages (3)
CGS3546-01-001.SLH	711	Chinese	F	Ovarian ca 60y; Endometrium and Cervical Polyp (benign)
CGS3068-01-001.TSK	712	Chinese	F	Breast ca 42y
CGS4152-01-001.PPS	713	Chinese	F	Breast ca 54y; Multi-factorial SOB, Hypokalaemia likely 2' poor oral intake, Symptomatic hypercalcaemia
CGS3221-01-001.TGM	714	Chinese	F	Breast ca 55y, Colorectal ca 55y; Atypical adenomatous proliferation in lungs, Ovarian cysts, Cholecystitis s/p laparoscopic cholecystectomy
CGS1146-01-001.LMY	715	Chinese	F	Breast ca 60y
CGS3906-01-001.MMJ	716	Indian	F	Ovarian ca 68y
CGS4302-01-001.NBD	717	Chinese	F	Ovarian ca 57y, Right cerebellar tumour (probs mets from primary ovarian cancer) 57y; Ischaemia (small bowel obstruction)
CGS4255-01-001.PST	718	Chinese	F	Breast ca 52y; Indeterminate adrenal mass
CGS3104-01-001.GGA	719	Eurasian	F	Breast ca 48y
CGS1281-01-001.KCL	720	Chinese	F	Breast ca 43y
CGS3397-01-001.GYM	721	Chinese	F	Breast ca 45y; benign phyllodes tumor, Acid reflux/ Dyspepsia
CGS3316-01-001.CMK	722	Chinese	F	Breast ca 42y; Back cyst, Eye lid cyst
CGS0742-01-001.LSP	723	Chinese	F	Breast ca 40y
CGS2053-01-001.SJL	724	Chinese	F	Breast ca 44y
CGS0743-01-001.HSB	725	Malay	M	Kidney ca 39y
CGS2072-01-001.OGH	726	Chinese	F	Breast ca 36y

CGS0438-01-001.AYL	727	Chinese	F	Breast ca 41y
CGS1180-01-001.LA	728	Indian	F	Breast ca 38y
CGS2884-01-001.P	729	Malay	F	Breast ca 40y
CGS1832-01-001.NCY	730	Chinese	F	Breast ca 38y
CGS0753-01-001.YSM	731	Chinese	F	Breast ca 33y
CGS3766-01-001.NSY	732	Chinese	F	Breast ca 37y
CGS4213-01-001.LN	733	Chinese	F	Kidney ca 32y; Depression, Cervical dysplasia
CGS0357-01-001.LLT	734	Chinese	F	Breast ca 25y
CGS3045-01-001.NHC	735	Chinese	F	Pancreatic ca 67y
CGS4188-01-001.LKN	736	Chinese	F	Breast ca 65y, Pheochromocytoma 54y; parathyroid adenoma, bilateral minimal carpal tunnel syndrome, Parkinson disease
CGS1735-01-001.LCK	737	Chinese	F	Colorectal ca 66y
CGS0210-01-001.CYS	738	Chinese	F	Kidney ca 77y, paraganglioma 65y, Cervical tumour 60y
CGS3373-01-001.LPL	739	Chinese	M	Prostate ca 84y
CGS3322-01-001.LEC	740	Chinese	F	Breast ca 58y; Noduler goiter, Endometrial polyp
CGS3265-01-001.LPW	741	Chinese	F	Breast ca 61y
CGS1143-01-001.HKL	742	Chinese	M	Colorectal ca 58y, Gastric ca 57y; Colon polyps
CGS0399-01-001.GKD	743	Sikh	F	Breast ca 50y
CGS0028-01-001.AYY	744	Chinese	F	Ovarian ca 53y
CGS1372-01-001.PKH	745	Chinese	M	Colorectal ca 35y
CGS3314-01-001.TSN	746	Chinese	F	Breast ca 55y
CGS3380-01-001.GMC	747	Chinese	F	Breast ca 54y
CGS0769-01-001.YSH	748	Chinese	F	Breast ca 50y
CGS0862-01-001.LLH	749	Chinese	F	Ovarian ca 53y, Endometrial ca 53y
CGS0772-01-001.TWK	750	Chinese	F	Breast ca 48y
CGS1223-01-001.RTS	751	Chinese	F	Breast ca 48y
CGS2420-01-001.LLM	752	Chinese	F	Ovarian ca 63y
CGS4032-01-001.ASH	753	Chinese	F	Pancreatic ca 65y
CGS2234-01-001.WG	754	Chinese	F	Breast ca 50y
CGS4049-01-001.TBC	755	Chinese	F	No cancer 52y; Bilateral ovarian cysts
CGS2077-01-001.CLY	756	Chinese	F	Breast ca 44y, sarcoma 46y
CGS2332-01-001.OCM	757	Chinese	F	Breast ca 39y
CGS3405-01-001.LHY	758	Chinese	F	Breast ca 43y
CGS4327-01-001.TYC	759	Chinese	M	Colorectal ca 44y
CGS2359-01-001.MSH	760	Chinese	F	Breast ca 40y
CGS3044-01-001.CHP	761	Chinese	F	Breast ca 41y
CGS0796-01-001.TYL	762	Chinese	F	Breast ca 34y
CGS3705-01-001.ZL	763	Chinese	F	Breast ca 40y
CGS0798-01-001.A	764	Indian	F	Breast ca 33y
CGS2378-01-001.LWL	765	Chinese	F	Breast ca 30y, Small cell carcinoma 31y
CGS3508-01-001.CJ	766	Chinese	F	Breast ca 38y
CGS3272-01-001.FDN	767	Indian	F	Breast ca 32y; Left benign lump
CGS1368-01-001.MFB	768	Pakistani	M	No cancer 23y; Right retinal angioma
CGS2470-01-001.TCK	769	Chinese	F	Pancreatic ca 73y
CGS1209-01-001.CLK	770	Chinese	F	Ovarian ca 68y
CGS0737-01-001.KCE	771	Chinese	F	Breast ca 46y, Gastric ca 69y, lymphoma 64y, Gall bladder cancer 69y

CGS2427-01-001.ABM	772	Malay	M	Prostate ca 72y
CGS1855-01-001.TGK	773	Chinese	F	Breast ca 72y, Endometrial ca 81y
CGS3795-01-001.LHT	774	Chinese	M	Prostate ca 62y
CGS3261-01-001.SGN	775	Chinese	F	Pancreatic ca 75y
CGS0705-01-001.LKH	776	Chinese	F	Ovarian ca 66y
CGS0450-01-001.CYK	777	Chinese	F	Breast ca 59y
CGS1922-01-001.CSK	778	Chinese	F	Breast ca 64y
CGS2444-01-001.LTP	779	Chinese	M	Colorectal ca 76y, prostate ca 75y, squamous cell carcinoma 75y
CGS2021-01-001.FMC	780	Chinese	F	Ovarian ca 63y
CGS2431-01-001.LLK	781	Chinese	F	Pancreatic ca 61y
CGS0188-02-001.TBG	782	Chinese	F	Breast ca 40y
CGS1606-01-001.HNH	783	Chinese	F	Breast ca 54y, Colorectal ca 57y
CGS3235-01-001.HYL	784	Chinese	F	Breast ca 35y; Breast Lump (left)
CGS0223-01-001.TPI	785	Chinese	F	Breast ca 38y, pancreatic ca 55y; Tubular adenoma with low grade dysplasia
CGS1339-01-001.PPC	786	Chinese	F	Breast ca 54y, Endometrial ca 54y
CGS0548-01-001.LKY	787	Chinese	F	Endometrial ca 52y
CGS3493-01-001.ECK	788	Chinese	M	Thyroid ca 54y; Caecal polyp: Tubular adenoma with low grade dysplasia.
CGS3918-01-001.CLL	789	Chinese	F	Ovarian ca 57y
CGS2313-01-001.SSM	790	Chinese	F	Breast ca 42y
CGS3037-01-001.FNP	791	Chinese	M	Paraganglioma 60y
CGS4101-01-001.MM	792	Pakistani	M	Prostate ca 56y
CGS0108-01-001.TSL	793	Chinese	F	Breast ca 31y
CGS0715-01-001.LAT	794	Chinese	F	Breast ca 45y, thyroid ca 47y
CGS0822-01-001.CEA	795	Chinese	F	Breast ca 45y
CGS2189-01-001.TSS	796	Chinese	F	Breast ca 44y
CGS3231-01-001.KS	797	Japanese	F	Breast ca 28y
CGS0824-01-001.OB	798	Caucasian	F	Breast ca 43y
CGS0827-01-001.TLC	799	Chinese	F	Breast ca 40y
CGS1679-01-001.NHS	800	Chinese	F	Breast ca 36y
CGS0830-01-001.TPS	801	Chinese	F	Breast ca 40y
CGS4387-01-001.LSH	802	Chinese	F	Breast ca 47y
CGS1853-01-001.CKS	803	Indian	F	Breast ca 44y
CGS2351-01-001.LME	804	Chinese	F	Breast ca 37y
CGS3034-01-001.LLY	805	Chinese	F	Breast ca 44y; Churg Strauss syndrome
CGS2464-01-001.ZY	806	Chinese	F	Breast ca 42y
CGS0835-01-001.RHB	807	Malay	F	Breast ca 36y
CGS2364-01-001.THK	808	Chinese	M	Colorectal ca 34y
CGS3583-01-001.LMY	809	Chinese	F	Breast ca 41y
CGS0840-01-001.MH	810	Indian	F	Breast ca 37y
CGS3987-01-001.KP	811	Chinese	F	Breast ca 38y
CGS2390-01-001.FYP	812	Chinese	F	No cancer 18y; Capillary hemangioma
CGS1332-01-001.TBE	813	Chinese	F	Squamous cell carcinoma 63y
CGS2199-01-001.NJP	814	Chinese	M	Pancreatic ca 70y
CGS0846-01-001.LYS	815	Chinese	M	Thyroid ca 64y
CGS3629-01-001.LPH	816	Chinese	F	Pancreatic ca 68y
CGS2256-01-001.WAM	817	Chinese	M	Breast ca 64y
CGS3199-01-001.LMK	818	Chinese	M	Pancreatic ca 71y; Ganglion Cyst

CGS2898-01-001.CYH	819	Chinese	M	Pancreatic ca 62y
CGS4116-01-001.RBS	820	Indian	F	Endometrial ca 63y
CGS3718-01-001.LHK	821	Chinese	F	Ovarian ca 62y
CGS0496-02-001.LLN	822	Chinese	F	Breast ca 46y
CGS2901-01-001.ZBS	823	Chinese	F	Breast ca 59y
CGS0855-01-001.OEL	824	Chinese	F	Ovarian ca 51y
CGS0857-01-001.ATS	825	Chinese	M	Prostate ca 54y
CGS2971-01-001.LPM	826	Chinese	F	Ovarian ca 57y, Endometrial ca 57y
CGS1319-01-001.SBH	827	Indian	F	Endometrial ca 51y, kidney ca 51y
CGS2295-01-001.CLL	828	Chinese	F	Pancreatic ca 53y
CGS0112-01-001.RBS	829	Malay	F	Breast ca 40y
CGS0277-01-001.LLG	830	Chinese	F	Breast ca 51y
CGS1347-01-001.WSH	831	Chinese	F	No cancer 50y; Left multiple retinal angiomas
CGS1423-01-001.LYL	832	Chinese	F	Ovarian ca 73y
CGS1513-01-001.NKM	833	Chinese	F	Ovarian ca 62y
CGS3440-01-001.TKL	834	Chinese	F	Breast ca 50y, Cervical cancer Stage IIB (mucinous adenoca) 58y
CGS1257-01-001.MJ	835	Chinese	F	Breast ca 29y
CGS1232-01-001.LBH	836	Chinese	F	Ovarian ca 57y
CGS3007-01-001.DE	837	Chinese	F	Ovarian ca 52y
CGS0867-01-001.CMY	838	Chinese	F	Breast ca 48y, thymoma 41y
CGS3300-01-001.SPT	839	Chinese	F	Breast ca 48y; Uterine fibroidGastric polyps
CGS0872-01-001.LKE	840	Chinese	F	Breast ca 43y
CGS0874-01-001.PEC	841	ıcasian (Brit	F	Breast ca 44y
CGS0060-01-001.NSF	842	Chinese	F	Colorectal ca 40y
CGS1183-01-001.MDB	843	Chinese	F	Breast ca 39y
CGS1739-01-001.CT	844	Chinese	F	Breast ca 37y; Benign phyllodes tumour, right breast
CGS4163-01-001.CPL	845	Chinese	F	Breast ca 33y
CGS4191-01-001.OSP	846	Chinese	F	Breast ca 40y
CGS4073-01-001.TPY	847	Chinese	F	Breast ca 40y; Hyperthyroid
CGS4178-01-001.CHF	848	Chinese	F	Breast ca 41y
CGS3105-01-001.SNB	849	Malay	F	Breast ca 35y
CGS1165-01-001.VT	850	Indonesian	F	Breast ca 30y
CGS2963-01-001.TCP	851	Chinese	F	Breast ca 31y
CGS0886-01-001.HIM	852	Chinese	F	Thyroid ca 62y
CGS4292-01-001.LLH	853	Chinese	F	Ovarian ca 69y, Colorectal ca 52y
CGS1514-01-001.KSY	854	Chinese	F	No cancer 64y; Bilateral multiple peripheral capillary haemagiomas
CGS0887-01-001.HHJ	855	Chinese	M	Prostate ca 57y
CGS3256-01-001.LMF	856	Chinese	M	Breast ca 69y; Haemorrhoids, Gout
CGS3728-01-001.LTL	857	Chinese	M	Prostate ca 59y; Hepatitis B
CGS3135-01-001.CAM	858	Chinese	F	Paraganglioma 72y
CGS4203-01-001.TAL	859	Chinese	F	Ovarian ca 67y
CGS0794-01-001.TKH	860	Chinese	F	Ovarian ca 59y
CGS3779-01-001.TTK	861	Chinese	M	Prostate ca 74y; Chronic hepb
CGS4031-01-001.TSE	862	Chinese	F	No cancer 66y; Benign fibrocystic left breast tissue
CGS2177-01-001.GLI	863	Chinese	F	Pancreatic ca 63y
CGS3631-01-001.HLC	864	Chinese	F	Ovarian ca 63y; colon polyp

CGS2989-01-001.RBA	865	Malay	F	Ovarian ca 58y
CGS0899-01-001.TGI	866	Chinese	F	Ovarian ca 53y
CGS0901-01-001.OBW	867	Chinese	F	Endometrial ca 53y
CGS1394-01-001.JKS	868	Chinese	F	Breast ca 47y, Colorectal ca 49y
CGS3977-01-001.NBZ	869	Malay	F	Endometrial ca 63y
CGS1872-01-001.KDN	870	Indian	F	Ovarian ca 51y
CGS3127-01-001.KSM	871	Indian	F	Breast ca 53y
CGS3291-01-001.KCH	872	Chinese	M	Breast ca 58y, Bone cancer (mets from breast cancer) 58y
CGS0908-01-001.GKG	873	Chinese	F	Breast ca 32y
CGS3411-01-001.CFC	874	Chinese	F	Breast ca 46y
CGS0796-01-002.TMK	875	Chinese	F	Breast ca 41y
CGS2348-01-001.HHY	876	Chinese	F	Breast ca 39y
CGS1090-01-001.FSN	877	Chinese	F	Breast ca 40y
CGS2133-01-001.TKY	878	Chinese	F	Endometrial ca 39y
CGS0458-01-001.CSC	879	Chinese	F	Breast ca 39y
CGS2195-01-001.LPS	880	Chinese	F	Breast ca 39y
CGS4114-01-001.NKL	881	Chinese	F	Breast ca 35y
CGS0184-01-001.MAB	882	Malay	M	No cancer 27y; Parathyroid Adenoma
CGS3167-01-001.WFK	883	Chinese	M	Pancreatic ca 71y; Colon polyps
CGS0921-01-001.TPN	884	Chinese	F	Ovarian ca 59y
CGS1788-01-001.TSL	885	Chinese	F	Breast ca 63y
CGS1754-01-001.GHL	886	Chinese	F	Ovarian ca 67y
CGS4062-01-001.LYL	887	Chinese	F	Breast ca 62y
CGS2250-01-001.LHW	888	Chinese	M	Pancreatic ca 71y
CGS4305-01-001.GAT	889	Chinese	F	Breast ca 72y, gastrointestinal stromal tumour 72y; Subclinical Hyperthyroidism, Gastric Ulcer (Gastritis)
CGS3504-01-001.TYG	890	Chinese	F	Breast ca 76y
CGS3560-01-001.YJN	891	Chinese	F	Pancreatic ca 72y
CGS0098-01-001.YKL	892	Chinese	F	Pheochromocytoma 71y
CGS1624-01-001.FKL	893	Chinese	F	Ovarian ca 75y
CGS2162-01-001.LAM	894	Chinese	F	Ovarian ca 69y
CGS0928-01-001.CAH	895	Chinese	M	Colorectal ca 64y
CGS2894-01-001.FAM	896	Chinese	F	Colorectal ca 76y
CGS0930-01-001.LFF	897	Chinese	F	Breast ca 45y
CGS3480-01-001.TCK	898	Chinese	F	Colorectal ca 65y
CGS3057-01-001.EGK	899	Chinese	F	Breast ca 64y
CGS0709-01-001.AHC	900	Chinese	F	Endometrial ca 53y
CGS0935-01-001.KCH	901	Chinese	F	Ovarian ca 54y
CGS4393-01-001.APY	902	Chinese	F	No cancer 64y; Polyp - Peutz Jegher cancer
CGS3284-01-001.OCY	903	Chinese	M	Pancreatic ca 62y
CGS1048-01-001.RBM	904	Malay	F	Ovarian ca 59y
CGS1314-01-001.LHC	905	Chinese	M	Prostate ca 56y
CGS1990-01-001.CKE	906	Chinese	F	Breast ca 59y
CGS1785-01-001.TBH	907	Chinese	F	Ovarian ca 42y
CGS3200-02-001.MAS	908	Indian	F	No cancer 59y; Pterygium (left)
CGS0402-01-001.TSM	909	Chinese	F	Breast ca 55y
CGS4288-01-001.KTT	910	Chinese	F	Breast ca 32y
CGS3568-01-001.LGY	911	Chinese	F	Breast ca 48y

CGS3080-01-001.HFL	912	Chinese	F	Ovarian ca 73y
CGS3100-01-001.LSH	913	Chinese	F	Breast ca 60y, Ovarian ca 59y; Brugada's syndrome
CGS3775-01-001.WKF	914	Chinese	F	Ovarian ca 57y
CGS3836-01-001.SKL	915	Chinese	F	Breast ca 56y
CGS3841-01-001.NYL	916	Chinese	F	Breast ca 58y, Colorectal ca 42y
CGS3019-01-001.NCG	917	Chinese	F	Breast ca 51y
CGS0948-01-001.TTK	918	Chinese	F	Breast ca 46y
CGS3575-01-001.LLS	919	Chinese	F	Breast ca 50y
CGS1665-01-001.OBN	920	Chinese	F	Breast ca 48y, thyroid ca 41y
CGS1334-01-001.WLY	921	Chinese	F	Breast ca 45y
CGS2965-01-001.CCY	922	Chinese	F	Breast ca 48y
CGS0956-01-001.FHY	923	Chinese	F	Ovarian ca 39y
CGS2016-01-001.NHB	924	Malay	F	Breast ca 32y
CGS3653-01-001.USK	925	Chinese	F	Breast ca 32y
CGS0966-01-001.LAB	926	Malay	F	Basal cell carcinoma 21y
CGS0967-01-001.ALZ	927	Chinese	F	No cancer 18y; Right eye angioma
CGS1952-01-001.CSS	928	Chinese	M	Ampullary cancer 70y
CGS0072-01-001.SPK	929	Chinese	F	Breast ca 62y
CGS0968-01-001.CL	930	Chinese	F	Breast ca 57y
CGS2424-01-001.KSS	931	Chinese	M	Prostate ca 65y
CGS4058-01-001.CCH	932	Chinese	F	Prostate ca 69y; BP
CGS3486-01-001.TCS	933	Chinese	M	Prostate ca 66y
CGS0971-01-001.ACT	934	Chinese	F	Ovarian ca 70y, other unknown ca
CGS1518-01-001.GSN	935	Chinese	F	Colorectal ca 72y
CGS1526-01-001.LSK	936	Chinese	F	Breast ca 61y
CGS3792-01-001.LOA	937	Chinese	F	Ovarian ca 76y
CGS0974-01-001.TSH	938	Chinese	F	Breast ca 47y
CGS1231-01-001.SKD	939	Sikh	F	Ovarian ca 51y
CGS2946-01-001.AWG	940	Indian	F	Breast ca 59y
CGS3671-01-001.WLY	941	Chinese	F	Gastric ca 58y
CGS2128-01-001.CYS	942	Chinese	M	Colorectal ca 56y; Desmoid-type fibromatosis
CGS3129-01-001.NBS	943	Javanese	F	Ovarian ca 54y
CGS0113-01-001.LSH	944	Chinese	F	Endometrial ca 50y
CGS0123-01-001.LCK	945	Chinese	F	Breast ca 52y
CGS4281-01-001.TSC	946	Chinese	F	Pancreatic ca 61y
CGS0622-01-001.MM	947	Indian	F	Ovarian ca 51y
CGS3985-01-001.GSK	948	Chinese	F	No cancer 64y; Retina angioma
CGS3053-01-001.MCH	949	Chinese	F	Pancreatic ca 49y
CGS2441-01-001.CKY	950	Chinese	F	Breast ca 49y
CGS1349-01-001.WMC	951	Chinese	F	Thyroid ca 46y
CGS1213-01-001.WSW	952	Chinese	F	Breast ca 46y
CGS0214-01-001.WMF	953	Chinese	F	Breast ca 41y
CGS1002-01-001.ALW	954	Chinese	F	Breast ca 40y
CGS1137-01-001.KLL	955	Chinese	F	Breast ca 41y
CGS3168-01-001.YYX	956	Chinese	F	Breast ca 43y
CGS3023-01-001.THN	957	Chinese	F	Breast ca 40y
CGS2374-01-001.SH	958	Indonesian	F	Breast ca 32y
CGS1012-01-001.ABZ	959	Malay	F	Breast ca 31y
CGS0195-01-001.SBS	960	Malay	F	Breast ca 33y

CGS3180-01-001.CKM	961	Chinese	M	No cancer 21y; multiple cervical lymphadenopathy, Lymphadenopathy, Dermatitis
CGS2416-01-001.NAA	962	Indian	F	Thyroid ca 19y; Severe aplastic anemia
CGS3667-01-001.KYZ	963	Chinese	F	Rhabdomyosarcoma: Orbital embryonal RMS 10
CGS2051-01-001.YZY	964	Chinese	F	Breast ca 19y
CGS1107-01-001.TZX	965	Chinese	M	Mixed mediastinal germ cell tumour 15y
CGS3403-01-001.VNR	966	Chinese	F	Sarcoma 16y
CGS1017-01-001.LCR	967	Chinese	M	No cancer 13y; Right capillary hemangioma
CGS3719-01-001.RWM	968	Indian	M	No cancer 18y; Megakaryocytic and erythroid hyperplasia
CGS2920-01-001.BMB	969	Filipino	M	Retinoblastoma 1y
CGS2878-01-001.SLJ	970	Chinese	M	Desmoid fibromatosis 17y
CGS1031-01-001.OSM	971	Arabic	M	Colorectal ca 91y
CGS1487-01-001.KHS	972	ed Arab Emi	F	Ovarian ca 67y
CGS2120-01-001.PD	973	Cambodian	F	Ovarian ca 46y
CGS1033-01-001.RM	974	Filipino	F	Breast ca 41y
CGS2393-01-001.SSS	975	ed Arab Emi	F	Breast ca 25y
CGS2395-01-001.NTK	976	Vietnamese	F	Breast ca 41y
CGS1035-01-001.AKL	977	Chinese	F	Kidney ca 58y, pituitary tumour age unknown
CGS0087-01-001.NTB	978	Vietnamese	F	Breast ca 43y
CGS2875-01-001.GSA	979	Chinese	F	Breast ca 48y
CGS0218-01-001.VCG	980	Filipino	F	Sarcoma 46y
CGS2137-01-001.TTY	981	Vietnamese	F	Breast ca 38y
CGS1749-01-001.HAS	982	Indonesian	F	Paraganglioma 37y
CGS1117-01-001.YYK	983	Burmese	F	Breast ca 37y
CGS1088-01-001.DTT	984	Vietnamese	F	Breast ca 41y
CGS1039-01-001.KAH	985	arabian	F	Thyroid ca 36y
CGS1040-01-001.JK	986	Chinese	F	Breast ca 49y
CGS2165-01-001.RS	987	Thai	F	Breast ca 32y
CGS1044-01-001.SR	988	Arabian	F	Breast ca 38y
CGS1903-01-001.ZAA	989	UAE	F	Breast ca 29y
CGS2956-01-001.HSA	990	x	F	Breast ca 45y
CGS1047-01-001.HMA	991	Bangladesh	F	Ovarian ca 53y
CGS1049-01-001.SS	992	Malay	F	Breast ca 48y
CGS1052-01-001.NK	993	pakistani	F	Ovarian ca 62y
CGS2032-01-001.PTH	994	Chinese	F	Breast ca 44y
CGS1336-01-001.MME	995	iddle Easte	F	Breast ca 30y
CGS1342-01-001.HL	996	Indonesian	F	Ovarian ca 61y
CGS0074-01-001.SAM	997	ed Arab Emi	F	Breast ca 30y
CGS1061-01-001.SAE	998	chean (cam	F	Breast ca 40y
CGS1828-01-001.MMY	999	Burmese	F	Breast ca 43y
CGS1062-01-001.CW	1000	Indonesian	F	Breast ca 37y
CGS1064-01-001.YCH	1001	Chinese	F	Thyroid ca 59y
CGS1870-01-001.KL	1002	ampuchea	F	Breast ca 35y
CGS1550-01-001.SMP	1003	Bangladesh	F	Ovarian ca 64y
CGS0500-01-001.JAB	1004	Indonesian	F	Breast ca 40y
CGS1078-01-001.PKH	1005	Vietnamese	M	Paraganglioma 60y
CGS2198-01-001.TLH	1006	Chinese	F	Breast CA 36y<br>Breast CA 50y

CGS4899-01-001.MBA	1007	Others	F	Desmoid Fibromatosis 34y
CGS4922-01-001.YK	1008	Malay	F	Breast CA 66y
CGS5043-01-001.SSN	1009	Indian	F	Breast CA 46y
CGS5084-01-001.LES	1010	Others	F	Breast CA 37y
CGS4840-01-001.KMT	1011	Chinese	F	Ovarian CA 39y
CGS4973-01-001.MTA	1012	Others	F	Breast CA 40y<br>Thyroid CA 40y
CGS5535-01-001.LPK	1013	Chinese	M	Salivary Gland CA 34y
CGS5208-01-001.ZJ	1014	Chinese	F	Breast CA 41y
CGS5994-01-001.ATM	1015	Others	F	Breast CA 43y
CGS4886-01-001.LXYN	1016	Chinese	F	Ovarian CA 12y
CGS6171-01-001.BP	1017	Others	F	Breast CA 44y
CGS0298-01-001.PCY	1018	Chinese	F	Ovarian CA 43y<br>ACC 66y<br>Lung CA 66y
CGS0405-01-001.FHW	1019	Chinese	F	Breast CA 48y
CGS0983-01-001.TAH	1020	Chinese	F	Ovarian CA 54y
CGS1199-01-001.LK	1021	Others	F	Ovarian CA 58y
CGS1463-01-001.YLM	1022	Chinese	F	Sarcoma 40y
CGS0085-01-001.TSY	1023	Chinese	F	Breast CA 42y<br>Breast CA 50y
CGS0189-01-001.HBS	1024	Malay	F	Breast CA 47y<br>Endometrial CA 56y
CGS0118-01-001.HST	1025	Chinese	F	Breast CA 55y
CGS0145-01-002.SMB	1026	Malay	F	Breast CA 43y<br>Breast CA 50y
CGS0446-01-001.CM	1027	Chinese	F	Breast CA 31
CGS0457-01-001.QBL	1028	Chinese	F	Breast CA 44y
CGS1589-01-001.LY	1029	Others	F	Breast CA 43y
CGS1522-01-002.LBK	1030	Chinese	F	Breast CA 49y
CGS0955-01-001.LSL	1031	Chinese	F	Breast CA 40y
CGS1748-01-001.TMT	1032	Chinese	F	Breast CA 39y
CGS1871-01-001.TSH	1033	Chinese	F	Colorectal CA 58y
CGS0984-01-001.SDS	1034	Indian	F	Breast CA 50y<br>Breast CA 51y
CGS1641-01-001.LJF	1035	Chinese	M	Parathyroid Carcinoma 51y
CGS1955-01-001.SCH	1036	Chinese	M	Colorectal CA 61y
CGS1957-01-001.CLK	1037	Chinese	M	Prostate CA 56y
CGS1985-01-001.HLS	1038	Chinese	F	Breast CA 44y
CGS3138-01-001.CYM	1039	Chinese	F	Breast CA 68y
CGS3326-01-001.TLN	1040	Chinese	F	Endometrial CA 61y
CGS3216-01-001.HWN	1041	Chinese	M	Sarcoma 68y
CGS3342-01-001.GLH	1042	Chinese	F	Breast CA 59y
CGS3439-01-001.PTP	1043	Chinese	F	Ovarian CA 26y
CGS3458-01-001.SEM	1044	Chinese	F	Breast CA 47y
CGS2290-01-001.TYY	1045	Chinese	F	Breast CA 37y
CGS3502-01-001.HY	1046	Chinese	F	Breast CA 45y
CGS3825-01-001.CYK	1047	Chinese	M	Prostate CA 71y
CGS3890-01-001.TTL	1048	Chinese	M	Prostate CA 66y
CGS3808-01-001.YJM	1049	Chinese	F	Breast CA 33y

CGS4042-01-001.NJB	1050	Malay	F	Ovarian CA 37y
CGS4009-01-001.STB	1051	Malay	F	Ovarian CA 45y
CGS4128-01-001.TAC	1052	Chinese	F	Ovarian CA 77y
CGS4139-01-001.CGH	1053	Chinese	F	Breast CA 46y
CGS4189-01-001.NAJ	1054	Chinese	F	Breast CA 42y
CGS4193-01-001.LPT	1055	Chinese	F	Colorectal CA 30y
CGS4216-01-001.ZY	1056	Chinese	F	Breast CA 46y
CGS4360-01-001.YLL	1057	Chinese	F	Breast CA 64y
CGS4406-01-001.SBD	1058	Malay	F	Ovarian CA 37y<br>Kidney CA 45y
CGS4391-01-001.NKC	1059	Chinese	F	Breast CA 50y
CGS4411-01-001.TKK	1060	Chinese	F	Breast CA 73y
CGS4411-03-001.ALS	1061	Chinese	F	Breast CA 38y
CGS4414-01-001.TCL	1062	Chinese	F	Breast CA 44y
CGS4115-01-001.M	1063	Indian	F	Endometrial CA 66y
CGS4420-01-001.LCC	1064	Chinese	M	Colorectal CA 60y
CGS4434-01-001.KKK	1065	Chinese	F	Breast CA 49y
CGS4380-01-001.TKB	1066	Chinese	F	Breast CA 51y
CGS4452-01-001.HCO	1067	Chinese	F	Breast CA 47y
CGS4458-01-001.NBA	1068	Malay	F	Breast CA 35y
CGS4459-01-001.RDS	1069	Others	M	Melanoma 34y
CGS4464-01-001.MLK	1070	Chinese	F	Breast CA 79y
CGS4470-01-001.CGE	1071	Chinese	F	Breast CA 77y
CGS4483-01-001.KGN	1072	Chinese	F	PGL/PCC 39y
CGS4481-01-001.JBJ	1073	Malay	F	Ovarian CA 46y
CGS4297-01-001.DNP	1074	Chinese	F	Breast CA 59y
CGS4488-01-001.CGJ	1075	Chinese	M	Kidney CA 29y
CGS4520-01-001.TKL	1076	Chinese	F	Breast CA 32y
CGS4582-01-001.SL	1077	Chinese	F	Breast CA 57y
CGS4523-01-001.AKJ	1078	Indian	F	Breast CA 50y
CGS4555-01-001.MS	1079	Chinese	F	Ovarian CA 44y
CGS4503-01-001.LYS	1080	Chinese	F	Breast CA 30y
CGS4522-01-001.BYH	1081	Others	F	Breast CA 44y
CGS4485-01-001.SPR	1082	Indian	M	Kidney CA 38y
CGS4546-01-001.SYL	1083	Chinese	F	Breast CA 43y
CGS4576-01-001.LSP	1084	Chinese	F	Breast CA 40y
CGS4579-01-001.TTC	1085	Chinese	M	Colorectal CA 74y
CGS4592-01-001.SS	1086	Others	F	Breast CA 35y
CGS4595-01-001.YLY	1087	Chinese	F	Breast CA 49y<br>Breast CA 52y
CGS4605-01-001.YPS	1088	Chinese	F	Breast CA 39y
CGS4608-01-001.OKE	1089	Chinese	F	Ovarian CA 68y
CGS4330-01-001.NSC	1090	Chinese	F	Breast CA 45y
CGS4625-01-001.DMD	1091	Indian	F	Breast CA 49y
CGS4626-01-001.TJC	1092	Chinese	M	Colorectal CA 32y
CGS4612-01-001.CGL	1093	Chinese	F	Breast CA 64y
CGS4614-01-001.TMM	1094	Chinese	F	Breast CA 53y
CGS4633-01-001.ATH	1095	Chinese	F	Breast CA 44y
CGS4637-01-001.YAG	1096	Chinese	F	Breast CA 38y<br>Endometrial CA 45y

CGS4692-01-001.KSL	1097	Chinese	M	Pancreatic CA 57y
CGS4632-01-001.CI	1098	Chinese	F	Breast CA 39y
CGS4684-01-001.NKC	1099	Chinese	M	Colorectal CA 55y
CGS4683-01-001.LSH	1100	Chinese	F	Breast CA 60y<br>Ovarian CA 68y & 70y
CGS4660-01-001.FJM	1101	Others	F	Breast CA 42y
CGS4669-01-001.GL	1102	Indian	F	Breast CA 63y
CGS4666-01-001.SAL	1103	Chinese	F	Breast CA 48y<br>Ovarian CA 55y
CGS4667-01-001.BTM	1104	Others	F	Melanoma 42y
CGS4681-01-001.LYY	1105	Chinese	F	Breast CA 43y
CGS4679-01-001.CSC	1106	Chinese	F	Breast CA 46y
CGS4678-01-001.GTL	1107	Chinese	F	Breast CA 38y
CGS4405-01-001.JLD	1108	Chinese	M	Retinoblastoma 2y
CGS4675-01-001.TCC	1109	Chinese	F	Breast CA 39y
CGS4630-01-001.LWP	1110	Chinese	F	Breast CA 44y<br>Breast CA 58y
CGS4705-01-001.HPT	1111	Chinese	F	Ovarian CA 58y
CGS4706-01-001.AM	1112	Malay	F	Breast CA 42y
CGS4740-01-001.STM	1113	Chinese	F	Ovarian CA 60y
CGS4741-01-001.MSN	1114	Chinese	F	Pancreatic CA 68y
CGS4751-01-001.TIC	1115	Chinese	F	Breast CA 40y
CGS4743-01-001.SNL	1116	Chinese	F	Breast CA 40y
CGS4782-01-001.SSH	1117	Chinese	F	Breast CA 35y
CGS4787-01-001.KYS	1118	Chinese	F	Breast CA 44y
CGS4768-01-001.MTV	1119	Others	F	Breast CA 32y
CGS4767-01-001.ZBI	1120	Malay	F	Ovarian CA 66y
CGS4802-01-001.NAB	1121	Malay	F	Ovarian CA 19y
CGS4807-01-001.ATC	1122	Chinese	M	Pancreatic CA 69y
CGS4799-01-001.LWL	1123	Chinese	F	Breast CA 47y
CGS4838-01-001.MDM	1124	Indian	F	Ovarian CA 42y<br>Colorectal CA 50y
CGS4757-01-001.MCM	1125	Others	F	Breast CA 41y
CGS4881-01-001.KSL	1126	Chinese	F	PGL/PCC 61y
CGS4853-01-001.WKW	1127	Chinese	M	Pancreatic CA 72y
CGS4517-01-001.CQK	1128	Chinese	M	Anaplastic ependymoma (WHO grade III) 1y
CGS4865-01-001.EJ	1129	Chinese	F	Breast CA 52y
CGS4918-01-001.TSH	1130	Chinese	M	Prostate CA 71y
CGS4871-01-001.LKS	1131	Chinese	M	Pancreatic CA 54y
CGS4969-01-001.KTL	1132	Chinese	M	Prostate CA 62y
CGS5011-01-001.TTK	1133	Chinese	F	Breast CA 64y
CGS4920-01-001.LEE	1134	Chinese	F	Breast CA 40y
CGS5017-01-001.NWL	1135	Chinese	F	Breast CA 53y
CGS5038-01-001.TLK	1136	Chinese	F	Breast CA 61y
CGS2324-01-001.PWS	1137	Chinese	F	Breast CA 42y
CGS5053-01-001.SZL	1138	Malay	F	Breast CA 39y
CGS5064-01-001.NHH	1139	Chinese	F	Breast CA 43y
CGS5058-01-001.PSH	1140	Chinese	F	Breast CA 24y
CGS5070-01-001.TEP	1141	Chinese	F	Ovarian CA 59y<br>Endometrial CA 59y

CGS5098-01-001.CHG	1142	Chinese	F	Breast CA 32y
CGS5100-01-001.SDP	1143	Indian	F	Pancreatic CA 60y
CGS5106-01-001.YSY	1144	Chinese	F	Breast CA 46y
CGS5139-01-001.CGH	1145	Chinese	F	Breast CA 61y
CGS5145-01-001.SMH	1146	Chinese	M	Colorectal CA 65y<br>Prostate CA 65y
CGS4929-01-001.MM	1147	Others	F	Ovarian CA 53y
CGS5124-01-001.SSU	1148	Chinese	F	Breast CA 43y
CGS5109-01-001.CK	1149	Chinese	M	Pancreatic CA 73y<br>Prostate CA 73y
CGS5118-01-001.CYH	1150	Chinese	F	Breast CA 43y
CGS5130-01-001.GDP	1151	Indian	F	Endometrial CA 56y
CGS5129-01-001.KTT	1152	Chinese	F	Breast CA 45y
CGS5168-01-001.OYS	1153	Chinese	F	Ovarian CA 73y
CGS5164-01-001.NHP	1154	Chinese	F	Breast CA 42y
CGS5163-01-001.KTH	1155	Chinese	F	Breast CA 65y<br>Breast CA 69y
CGS4786-01-001.CPY	1156	Chinese	F	Breast CA 33y
CGS5016-01-001.YJC	1157	Chinese	F	Papillary Thyroid Cancer 28y
CGS5201-01-001.AN	1158	Chinese	F	Ovarian CA 63y
CGS5185-01-001.LQ	1159	Chinese	F	Breast CA 45y
CGS5183-01-001.CLY	1160	Chinese	F	Breast CA 39y
CGS5149-01-001.LLL	1161	Chinese	F	Breast CA 48y
CGS5219-01-001.NFT	1162	Chinese	M	Pancreatic CA 66y
CGS5113-01-001.CS	1163	Indian	F	Breast CA 41y
CGS5257-01-001.TXT	1164	Chinese	F	Breast CA 36y
CGS5262-01-001.CHM	1165	Chinese	F	Breast CA 42y
CGS5266-01-001.JCA	1166	Others	F	Breast CA 47y
CGS5134-01-001.CMK	1167	Chinese	M	Colorectal CA 68y
CGS5217-01-001.NAM	1168	Chinese	F	Ovarian CA 70y
CGS5319-01-001.TGL	1169	Chinese	F	Breast CA 56y
CGS5329-01-001.LMW	1170	Chinese	F	Breast CA 52y
CGS5332-01-001.YTL	1171	Chinese	M	Gastric CA 59y
CGS5346-01-001.SLM	1172	Others	F	Colorectal CA 29y
CGS5340-01-001.CSW	1173	Chinese	F	Kidney CA 35y
CGS5052-01-001.LBD	1174	Others	F	Breast CA 31y & 35y
CGS5352-01-001.TBS	1175	Chinese	M	Prostate CA 53y
CGS5364-01-001.CM	1176	Chinese	F	Endometrial CA 40y
CGS5385-01-001.LRB	1177	Malay	F	Breast CA 42y
CGS5394-01-001.YKM	1178	Chinese	F	Breast CA 45y<br>Colorectal CA 49y
CGS5427-01-001.LJ	1179	Chinese	F	Colorectal CA 44y
CGS5402-01-001.KSH	1180	Chinese	F	Breast CA 56y<br>Breast CA 68y
CGS5231-01-001.TMK	1181	Chinese	F	Breast CA 57y<br>Breast CA 62y
CGS5097-01-001.SNB	1182	Malay	F	Breast CA 23y
CGS5462-01-001.GWL	1183	Chinese	F	Breast CA 45y<br>Breast CA 50y
CGS4806-01-001.CCH	1184	Chinese	F	Breast CA 46y

CGS5309-01-001.YWL	1185	Chinese	F	Breast CA 44y
CGS5488-01-001.CN	1186	Chinese	F	Ovarian CA 58y
CGS5494-01-001.HSS	1187	Chinese	M	Prostate CA 65y
CGS5495-01-001.NDK	1188	Indian	F	Breast CA 73y
CGS5501-01-001.IAP	1189	Chinese	F	Breast CA 57y
CGS5234-01-001.CKH	1190	Chinese	F	Breast CA 37y
CGS5314-01-001.SRB	1191	Malay	F	Breast CA 41y
CGS5337-01-001.KTP	1192	Chinese	F	Ovarian CA 44y<br>Endometrial CA 44y
CGS5555-01-001.TSH	1193	Chinese	F	PGL/PCC 58y
CGS5600-01-001.NBE	1194	Chinese	F	Breast CA 60y
CGS5591-01-001.CYF	1195	Chinese	F	Breast CA 33y
CGS5604-01-001.KLT	1196	Chinese	F	Thyroid CA 54y
CGS5514-01-001.TPH	1197	Chinese	F	Breast CA 47y
CGS5617-01-001.TBA	1198	Indian	F	Colorectal CA 56y
CGS5644-01-001.LKY	1199	Chinese	F	Breast CA 35y
CGS5678-01-001.TKE	1200	Chinese	F	Ovarian CA 55y
CGS5709-01-001.HL	1201	Chinese	F	Ovarian CA 54y
CGS5719-01-001.TLE	1202	Chinese	F	Breast CA 43y<br>Breast CA 50y
CGS5601-01-001.SKL	1203	Chinese	M	Colorectal CA 41y
CGS5746-01-001.TLL	1204	Chinese	F	Ovarian CA 57y
CGS5745-01-001.SRB	1205	Malay	F	Breast CA 34y
CGS5820-01-001.GLW	1206	Chinese	M	Pancreatic CA 52y
CGS5518-01-001.TLK	1207	Chinese	F	Ovarian CA 65y
CGS5992-01-001.HBH	1208	Malay	F	Ovarian CA 55y
CGS6006-01-001.VT	1209	Chinese	F	Ovarian CA 48y
CGS6039-01-001.IL	1210	Chinese	F	Ovarian CA 61y
CGS6042-01-001.QBL	1211	Chinese	F	Breast CA 58y
CGS6022-01-001.RBR	1212	Malay	F	Breast CA 57y
CGS6073-01-001.NBB	1213	Chinese	F	Breast CA
CGS6072-01-001.WSC	1214	Chinese	F	Breast CA
CGS5531-01-001.LKK	1215	Chinese	M	Colorectal CA 64y<br>Gastric CA 64y<br>Bladder CA 77y
CGS6077-01-001.SML	1216	Chinese	F	Breast CA 50y
CGS6079-01-001.KJC	1217	Chinese	F	Breast CA 42y
CGS6082-01-001.LZE	1218	Chinese	F	Ovarian CA 31y
CGS6092-01-001.LFH	1219	Chinese	F	Breast CA 55y<br>Leukemia / Lymphoma (CLL) 43y
CGS6095-01-001.SYC	1220	Chinese	M	Prostate CA 52y
CGS6101-01-001.TLE	1221	Chinese	F	Breast CA 60y<br>Breast CA 62y
CGS6099-01-001.LMH	1222	Chinese	F	Breast CA 38y
CGS6105-01-001.CCL	1223	Chinese	F	Breast CA 58y
CGS5731-01-001.CEC	1224	Chinese	F	Breast CA 44y
CGS6142-01-001.LGC	1225	Chinese	F	Breast CA 45y
CGS6115-01-001.TSH	1226	Chinese	F	Breast CA 44y<br>Breast CA 63y
CGS6127-01-001.CBC	1227	Chinese	M	Colorectal CA 49y

CGS6120-01-001.OH	1228	Chinese	M	Prostate CA 66y
CGS6124-01-001.DLZ	1229	Chinese	F	Breast CA 43y
CGS6132-01-001.SBM	1230	Malay	F	Breast CA 59y
CGS6133-01-001.BBM	1231	Indian	F	Breast CA 31y
CGS6145-01-001.NBA	1232	Malay	F	Breast CA 58y
CGS6152-01-001.YPH	1233	Chinese	F	Breast CA 62y
CGS5808-01-001.YYW	1234	Chinese	F	Breast CA 42y
CGS6180-01-001.SSK	1235	Chinese	F	Endometrial CA 57y
CGS6181-01-001.FEH	1236	Chinese	F	Breast CA 45y<br>Breast CA 55y & 56y
CGS6183-01-001.AGH	1237	Chinese	F	Breast CA 57y
CGS6012-01-001.GEY	1238	Chinese	F	Breast CA 46y
CGS6310-01-001.CCH	1239	Chinese	F	Ovarian CA 51y
CGS4544-01-001.YKC	1240	Chinese	F	Breast CA 55y<br>Ovarian CA 73y
CGS5037-01-001.TKK	1241	Chinese	M	Pancreatic CA 66y
CGS2043-01-001.NSC	1242	Chinese	M	Kidney CA 58y<br>PGL/PCC 51y<br>Lung CA 58y
CGS1421-01-001.SL	1243	Others	F	Ovarian CA 73y
CGS5065-01-001.CHY	1244	Chinese	F	CA of unknown primary 66y
CGS4993-01-001.YPS	1245	Chinese	F	Pancreatic CA 70y
CGS6017-01-001.FSM	1246	Chinese	F	Ovarian CA 72y
CGS4559-01-001.EGY	1247	Chinese	F	Ovarian CA 74y
CGS5524-01-001.TJC	1248	Chinese	M	Prostate CA 72y
CGS3498-01-001.LTL	1249	Chinese	M	Prostate CA 69y
CGS5529-01-001.CTM	1250	Chinese	F	Breast CA 73y
CGS5025-01-001.CSH	1251	Chinese	F	Gastric CA 75y
CGS5333-01-001.TCW	1252	Chinese	M	Prostate CA 72y
CGS5035-01-001.SYH	1253	Chinese	F	Ovarian CA 70y
CGS1682-01-001.BBS	1254	Malay	M	Colorectal CA 86y
CGS5813-01-001.HCF	1255	Chinese	F	Breast CA 75y
CGS1700-01-002.LHT	1256	Chinese	F	Breast CA 57y
CGS4702-01-001.CJK	1257	Chinese	F	Ovarian CA 65y
CGS5614-01-001.LAC	1258	Chinese	F	Breast CA 51y
CGS4127-01-001.TBE	1259	Chinese	F	Ovarian CA 63y
CGS5834-01-001.TLP	1260	Chinese	F	Ovarian CA 62y<br>Breast CA 63y
CGS5079-01-001.HBA	1261	Malay	F	Pancreatic CA 63y
CGS4839-01-001.VDS	1262	Indian	F	Ovarian CA 55y
CGS5663-01-001.OSH	1263	Chinese	F	Ovarian CA 56y
CGS5585-01-001.SSN	1264	Chinese	F	Breast CA 56y
CGS4976-01-001.CSF	1265	Chinese	F	Breast CA 60y
CGS5679-01-001.MTT	1266	Chinese	F	Breast CA 49y & 56y
CGS4997-01-001.DJM	1267	Others	M	Prostate CA 59y
CGS4067-01-001.CSL	1268	Chinese	F	Breast CA 52y
CGS4372-01-001.TSH	1269	Chinese	F	Breast CA 48y
CGS5345-01-001.NSE	1270	Chinese	F	Endometrial CA 50y
CGS6134-01-001.CSM	1271	Chinese	F	Breast CA 47y
CGS4699-01-001.HSL	1272	Chinese	F	Breast CA 40y

CGS5441-01-001.TET	1273	Chinese	F	Breast CA 41y
CGS4535-01-001.LYH	1274	Chinese	F	Breast CA 32y & 36y
CGS5418-01-001.SHB	1275	Malay	F	Extramedullary Multiple Myeloma (MM) 30y<br>Lobular capillary haemangioma 29y
CGS4652-01-001.NAB	1276	Malay	F	Breast CA 27y
CGS4422-01-001.FL	1277	Chinese	F	SCC 23y
CGS5180-01-001.TS	1278	Chinese	M	Prostate CA 69y
CGS4827-01-001.KBA	1279	Malay	F	Breast CA 63y
CGS5245-01-001.NCL	1280	Chinese	F	Breast CA 77y
CGS0590-01-001.CGM	1281	Chinese	F	Ovarian CA 60y
CGS6122-01-001.TJK	1282	Chinese	F	Breast CA 57y<br>Pancreatic CA 76y
CGS5750-01-001.PKH	1283	Chinese	F	Breast CA 73y
CGS4662-01-001.KSC	1284	Chinese	F	Ovarian CA 73y
CGS5078-01-001.HGC	1285	Chinese	F	Ovarian CA 48 & 68y<br>Endometrial CA 48y
CGS3589-01-001.TNT	1286	Chinese	M	Prostate CA 63y
CGS5386-01-001.WCH	1287	Chinese	M	Colorectal CA 50y<br>Prostate CA 60y<br>Lung CA 60y
CGS5085-01-001.LWK	1288	Chinese	M	Colorectal CA 62y
CGS4528-01-001.LGT	1289	Chinese	M	Pancreatic CA 62y
CGS5951-01-001.WYF	1290	Chinese	F	Ovarian CA 62y<br>Endometrial CA 62y
CGS4876-01-001.LTS	1291	Chinese	M	Colorectal CA 59y
CGS4739-01-001.BMD	1292	Indian	F	Ovarian CA 57y
CGS4893-01-001.LHC	1293	Chinese	M	Colorectal CA 55y<br>Gastric CA 58y
CGS4505-01-001.OCH	1294	Chinese	M	Pancreatic CA 58y
CGS3855-01-001.PSC	1295	Chinese	F	Breast CA 40y
CGS2171-01-001.YPL	1296	Chinese	F	Breast CA 54y
CGS4730-01-001.KKD	1297	Others	F	Breast CA 55y
CGS6025-01-001.LSK	1298	Chinese	F	Breast CA 65y
CGS1904-01-001.TTH	1299	Chinese	F	Breast CA 42y & 46y
CGS4885-01-001.TFL	1300	Chinese	F	Breast CA 45y
CGS4696-01-001.TCY	1301	Chinese	F	Endometrial CA 43y
CGS2412-01-001.RRD	1302	Indian	F	Thyroid CA 32y<br>Leukemia / Lymphoma (BCL) 38y
CGS2030-01-001.SSS	1303	Chinese	F	Breast CA 30y
CGS4602-01-001.CKY	1304	Chinese	F	Breast CA 29y
CGS2034-01-001.HKP	1305	Chinese	F	Ovarian CA 67y
CGS3059-01-001.TSH	1306	Chinese	F	Breast CA 64y<br>Ovarian CA64y
CGS5469-01-001.LCT	1307	Chinese	M	Breast CA 69y
CGS5454-01-001.TWK	1308	Chinese	F	Ovarian CA 72y
CGS5435-01-001.CAG	1309	Chinese	F	Ovarian CA 72y
CGS4901-01-001.MKS	1310	Chinese	M	Thyroid CA 58y<br>Pancreatic CA 65y
CGS6087-01-001.CLP	1311	Chinese	F	Breast CA 59y

CGS5373-01-001.YLH	1312	Chinese	F	Breast CA 61y<br>Gall Bladder CA 64y
CGS5788-01-001.TC	1313	Chinese	F	Breast CA 63y
CGS5845-01-001.KTH	1314	Chinese	M	Prostate CA 61y
CGS5946-01-001.SLG	1315	Chinese	F	Ovarian CA 58y
CGS5797-01-001.KSM	1316	Chinese	F	Breast CA 57y
CGS4656-01-001.FSL	1317	Chinese	F	Breast CA 51y
CGS4892-01-001.NNM	1318	Others	F	Breast CA 54y
CGS5434-01-001.GH	1319	Chinese	F	Thyroid CA 53y<br>Pancreatic CA 53y
CGS5490-01-001.LSC	1320	Chinese	F	Breast CA 53y
CGS1311-01-001.LHT	1321	Chinese	F	Breast CA 46y
CGS4716-01-001.AW	1322	Chinese	F	Breast CA 47y
CGS4784-01-001.CHS	1323	Chinese	F	Breast CA 44y
CGS3794-01-001.WPY	1324	Chinese	F	Desmoid Tumor 22y
CGS4417-01-001.ABA	1325	Malay	F	Breast CA 39y
CGS4890-01-001.LCL	1326	Chinese	F	Ovarian CA 41y
CGS6153-01-001.THY	1327	Chinese	F	Breast CA 37y
CGS4682-01-001.QHC	1328	Chinese	F	Pancreatic CA 69y
CGS3668-01-001.LSH	1329	Chinese	M	Pancreatic CA 66y
CGS3934-01-001.WHL	1330	Chinese	M	Prostate CA 72y
CGS4473-01-001.HKG	1331	Chinese	F	Pancreatic CA 75y
CGS4754-01-001.BEW	1332	Chinese	M	Colorectal CA 71y<br>Colorectal CA 72y<br>Prostate CA 75y<br>Lung CA 77y
CGS5636-01-001.CKS	1333	Chinese	M	Prostate CA 73y
CGS5556-01-001.WET	1334	Chinese	F	Pancreatic CA 70y
CGS5045-01-001.TNE	1335	Chinese	F	Pancreatic CA 70y
CGS6010-01-001.KDG	1336	Indian	F	Breast CA 69y<br>Ovarian CA 76y
CGS4435-01-001.SLC	1337	Chinese	F	Breast CA 66y<br>Colorectal CA 65y<br>Lung CA 65y
CGS4869-01-001.EGE	1338	Chinese	F	Colorectal CA 63y
CGS5044-01-001.TGH	1339	Chinese	F	Breast CA 39y
CGS5662-01-001.WWJ	1340	Chinese	F	Ovarian CA 61y
CGS4854-01-001.KCC	1341	Chinese	F	Breast CA 58y
CGS4502-01-001.LKK	1342	Chinese	F	Colorectal CA 58y
CGS4661-01-001.NBJ	1343	Malay	F	Breast CA 58y
CGS5343-01-001.KKY	1344	Chinese	F	Ovarian CA 56y<br>Endometrial CA 56y
CGS5542-01-001.ABM	1345	Malay	F	Breast CA 55y
CGS1813-01-001.CS	1346	Indian	F	Breast CA 51y
CGS3247-01-001.NG	1347	Indian	F	Breast CA 53y
CGS5292-01-001.TBL	1348	Chinese	F	Breast CA 62y & 66y
CGS2160-01-001.YSP	1349	Chinese	M	PGL/PCC 61y
CGS5405-01-001.STH	1350	Chinese	M	Pancreatic CA 59y
CGS4474-01-001.LHC	1351	Chinese	F	Breast CA 48y
CGS5732-01-001.HHB	1352	Indian	F	Breast CA 47y

CGS6045-01-001.KSY	1353	Chinese	F	Breast CA 45y
CGS4903-01-001.RRK	1354	Others	F	Breast CA 32y
CGS4991-01-001.TSG	1355	Chinese	F	Breast CA 69y
CGS3684-01-001.LLT	1356	Chinese	M	Prostate CA 64y
CGS3657-01-001.TAH	1357	Chinese	F	Breast CA 65y
CGS4562-01-001.CMK	1358	Chinese	F	Ovarian CA 63y
CGS5230-01-001.QMG	1359	Chinese	F	Ovarian CA 63y
CGS5102-01-001.CHK	1360	Chinese	F	Ovarian CA 60y
CGS5275-01-001.NCH	1361	Chinese	F	Ovarian CA 56y
CGS4588-01-001.LPC	1362	Chinese	F	Ovarian CA 71y
CGS5523-01-001.LYL	1363	Chinese	F	Ovarian CA 59y
CGS5666-01-001.GSY	1364	Chinese	F	Breast CA 49y
CGS5950-01-001.TNM	1365	Chinese	F	Breast CA 47y
CGS4934-01-001.CFE	1366	Chinese	F	Pancreatic CA 45y
CGS4266-01-001.I	1367	Malay	F	Breast CA 44y
CGS4797-01-001.CMF	1368	Chinese	F	Breast CA 40y
CGS5048-01-001.TJY	1369	Chinese	F	Breast CA 37y
CGS5816-01-001.KS	1370	Chinese	F	Breast CA 34y
CGS4836-01-001.FSL	1371	Chinese	M	Pancreatic CA 69y
CGS6143-01-001.LSH	1372	Chinese	M	Pancreatic CA 71y
CGS4415-01-001.LYT	1373	Chinese	M	Pancreatic CA 67y
CGS5674-01-001.YTK	1374	Chinese	M	Prostate CA 68y
CGS3723-01-001.CSL	1375	Chinese	M	Prostate CA 74y
CGS5279-01-001.GBC	1376	Chinese	F	Breast CA 63y<br>Endometrial CA 68y
CGS4823-01-001.YSP	1377	Chinese	F	Ovarian CA 66y
CGS5071-01-001.CAK	1378	Chinese	F	Breast CA 63y
CGS4471-01-001.RHB	1379	Malay	F	Breast CA 62y
CGS5160-01-001.TES	1380	Chinese	F	Ovarian CA 59y
CGS4837-01-001.LMN	1381	Others	F	Primary Peritoneal CA 83y
CGS4928-01-001.KBS	1382	Malay	F	Breast CA 57y
CGS3875-01-001.GSS	1383	Others	M	Pancreatic CA 55y
CGS5423-01-001.CAM	1384	Chinese	F	Breast CA 53y
CGS5291-01-001.LJC	1385	Chinese	F	Breast CA 54y<br>Leukemia / Lymphoma 25y
CGS4908-01-001.PJF	1386	Chinese	M	Prostate CA 74y
CGS4124-01-001.LYK	1387	Chinese	F	Ovarian CA 70y
CGS5789-01-001.THP	1388	Chinese	F	Ovarian CA 52y
CGS4499-01-001.WYS	1389	Chinese	F	Breast CA 49y
CGS5120-01-001.MOT	1390	Chinese	F	Breast CA 47y
CGS4731-01-001.CHH	1391	Chinese	F	Breast CA 43y
CGS4941-01-001.ZQ	1392	Chinese	F	Breast CA 42y
CGS5566-01-001.BBM	1393	Malay	F	Breast CA 65y
CGS4412-01-001.ASL	1394	Chinese	F	Colorectal CA 49y<br>Cervical CA 69y
CGS5651-01-001.NSM	1395	Chinese	F	Pancreatic CA 84y
CGS5868-01-001.CPH	1396	Chinese	F	Breast CA 65y
CGS3777-01-001.KWS	1397	Chinese	M	Colorectal CA 62y
CGS5752-01-001.SCF	1398	Chinese	M	Prostate CA 60y
CGS4974-01-001.NLH	1399	Chinese	F	Breast CA 61y

CGS4738-01-001.NSP	1400	Chinese	F	Breast CA 55y
CGS5077-01-001.YLS	1401	Chinese	M	Prostate CA 76y
CGS5247-01-001.LKL	1402	Chinese	F	Ovarian CA 60y
CGS4363-01-001.GYN	1403	Chinese	F	Breast CA 48y
CGS5687-01-001.CYP	1404	Chinese	F	Breast CA 47y
CGS5067-01-001.ATM	1405	Chinese	F	Ovarian CA 46y
CGS5645-01-001.GYS	1406	Chinese	F	Ovarian CA 45y
CGS5347-01-001.NSL	1407	Chinese	F	Breast CA 44y
CGS4758-01-001.CYF	1408	Chinese	F	Breast CA 43y
CGS4179-01-001.SGW	1409	Chinese	F	Breast CA 36y
CGS6118-01-001.SL	1410	Chinese	F	Breast CA 37y
CGS4895-01-001.FKL	1411	Chinese	F	Breast CA 67y<br>Endometrial CA 67y
CGS5125-01-001.LPN	1412	Chinese	F	Breast CA 75y
CGS5583-01-001.TBC	1413	Chinese	F	Breast CA 80y
CGS4132-01-001.TKN	1414	Chinese	M	Prostate CA79y
CGS4687-01-001.LLC	1415	Chinese	F	Breast CA 64y
CGS4736-01-001.NHK	1416	Chinese	F	Breast CA 44y
CGS0734-01-001.CKK	1417	Chinese	F	Ovarian CA 59y
CGS5252-01-001.QMH	1418	Chinese	M	Thyroid CA 65y
CGS3946-01-001.WYK	1419	Chinese	F	CA of unknown primary 61y
CGS3642-01-001.TCK	1420	Chinese	F	Breast CA 54y<br>Breast CA 61y
CGS5414-01-001.CLM	1421	Chinese	M	Prostate CA 60y
CGS4495-01-001.LHC	1422	Chinese	F	Breast CA 59y<br>Endometrial CA 59y
CGS5154-01-001.NSH	1423	Chinese	M	Pancreatic CA 59y
CGS5122-01-001.TCH	1424	Chinese	F	Ovarian CA 56y
CGS6005-01-001.HGN	1425	Chinese	F	Breast CA 56y
CGS0514-01-001.HLM	1426	Chinese	F	Breast CA 49y
CGS2169-01-001.DYP	1427	Chinese	F	Breast CA 39y & 48y
CGS6024-01-001.THL	1428	Chinese	F	Breast CA 53y
CGS4880-01-001.HL	1429	Chinese	F	Breast CA 42y
CGS4800-01-001.LHF	1430	Chinese	F	Breast CA 50y
CGS3599-03-001.LKL	1431	Chinese	F	Breast CA 43y
CGS4541-01-001.LSC	1432	Chinese	F	Breast CA 48y
CGS5717-01-001.TJ	1433	Chinese	F	Breast CA 46y
CGS4851-01-001.PM	1434	Chinese	F	Ovarian CA 40y
CGS4561-01-001.THE	1435	Chinese	F	Breast CA 69y
CGS5446-01-001.CSL	1436	Chinese	F	Breast CA 70y
CGS5153-01-001.LHE	1437	Chinese	F	Colorectal CA 70y<br>Lung CA 70y
CGS5533-01-001.LHS	1438	Chinese	M	Colorectal CA 77y<br>Gastric CA 77y
CGS4645-01-001.CLS	1439	Chinese	F	Breast CA 76y
CGS5452-01-001.LSC	1440	Chinese	F	Ovarian CA 75y
CGS5736-01-001.LGE	1441	Chinese	F	Breast CA 62y<br>Breast CA 79y
CGS5541-01-001.LWH	1442	Chinese	F	Pancreatic CA 67y
CGS5477-01-001.RKY	1443	Chinese	F	Breast CA 65y

CGS5567-01-001.TBM	1444	Malay	M	Prostate CA 59y
CGS5586-01-001.WKY	1445	Chinese	F	Breast CA 67y<br>Lung CA 67y
CGS5199-01-001.KB	1446	Chinese	F	Ovarian CA 64y
CGS5372-01-001.HML	1447	Chinese	F	Ovarian CA 60y
CGS6096-01-001.QSC	1448	Chinese	F	Ovarian CA 63y<br>Uterine CA 63y
CGS5691-01-001.KBA	1449	Malay	F	Breast CA 61y
CGS5818-01-001.GKH	1450	Chinese	F	Breast CA 60y
CGS5261-01-001.SSH	1451	Chinese	F	Breast CA 58y
CGS4932-01-001.CAS	1452	Chinese	F	Breast CA 57y
CGS0474-01-001.MMN	1453	Indian	F	Breast CA 39y
CGS4480-01-001.CMC	1454	Chinese	F	Breast CA 54y
CGS4902-01-001.MZB	1455	Indian	M	Colorectal CA 70y
CGS3851-01-001.LBH	1456	Chinese	F	Breast CA 60y<br>Breast CA 65y & 67y
CGS6094-01-001.JH	1457	Chinese	F	Colorectal CA 43y
CGS5049-01-001.NLH	1458	Chinese	F	Breast CA 42y
CGS5735-01-001.DWX	1459	Chinese	F	Breast CA 43y
CGS5534-01-001.LY	1460	Chinese	F	Breast CA 36y
CGS5209-01-001.TPN	1461	Chinese	F	Ovarian CA 67y
CGS4623-01-001.DSJ	1462	Others	F	Ovarian CA 71y
CGS5766-01-001.LJC	1463	Chinese	F	Colorectal CA 88y
CGS5019-01-001.CKN	1464	Chinese	F	Pancreatic CA 76y<br>Leukemia / Lymphoma 73y
CGS5324-01-001.FTY	1465	Chinese	F	Breast CA 72y
CGS4888-01-001.CAC	1466	Chinese	F	Breast CA 75y
CGS4617-01-001.ZBA	1467	Malay	F	Breast CA 66y
CGS5539-01-001.LC	1468	Chinese	F	Ovarian CA 65y
CGS5374-01-001.PSC	1469	Chinese	F	Breast CA 62y
CGS5569-01-001.OSJ	1470	Chinese	F	Breast CA 60y<br>Endometrial CA 54y
CGS5289-01-001.LHK	1471	Chinese	F	Breast CA 55y
CGS3833-01-001.NBL	1472	Chinese	F	Breast CA 52y
CGS4568-01-001.LAC	1473	Chinese	F	Breast CA 60y
CGS2074-01-001.FME	1474	Chinese	F	Ovarian CA 70y
CGS5147-01-001.CBN	1475	Chinese	M	Prostate CA 57y<br>Pancreatic CA 73y
CGS4703-01-001.SBH	1476	Chinese	F	Breast CA 32y
CGS5747-01-001.KPF	1477	Chinese	F	Breast CA 53y
CGS4204-01-001.CPH	1478	Chinese	F	Breast CA 37y
CGS4985-01-001.NLY	1479	Chinese	F	Breast CA 47y
CGS4724-01-001.RBS	1480	Others	F	Breast CA 46y
CGS0958-01-001.TBL	1481	Chinese	F	Breast CA 38y
CGS5366-01-001.MMB	1482	Indian	F	Breast CA 46y
CGS4629-01-001.SMF	1483	Chinese	F	Breast CA 38y
CGS5472-01-001.CMG	1484	Chinese	F	Breast CA 38y
CGS4846-01-001.LJS	1485	Chinese	F	Breast CA 34y
CGS3533-01-001.YKM	1486	Chinese	M	Prostate CA 73y
CGS4489-01-001.WJE	1487	Chinese	F	Ovarian CA 75y

CGS4994-01-001.JK	1488	Others	F	Breast CA 66y
CGS4615-01-001.TTH	1489	Chinese	M	Pancreatic CA 73y
CGS5763-01-001.YNL	1490	Chinese	M	Prostate CA 75y
CGS6036-01-001.CCL	1491	Chinese	F	Pancreatic CA 82y
CGS6223-01-001.SBA	1492	Malay	F	Breast CA 59y<br>Endometrial CA 73y
CGS5773-01-001.TTK	1493	Chinese	F	Colorectal CA 74y<br>Endometrial CA 75y
CGS4409-01-001.CGH	1494	Chinese	F	Breast CA 67y
CGS5769-01-001.CYP	1495	Chinese	F	Breast CA 48y
CGS4857-01-001.LKL	1496	Chinese	F	Breast CA 63y
CGS5391-01-001.LSG	1497	Chinese	F	Breast CA 64y
CGS5913-01-001.KGL	1498	Chinese	F	Breast CA 62y
CGS5012-01-001.QLF	1499	Chinese	F	Breast CA 47y
CGS4514-01-001.KSH	1500	Chinese	F	Breast CA 46y<br>Breast CA 56y
CGS4403-01-001.GHF	1501	Chinese	F	Breast CA 45y<br>Breast CA 54y
CGS5172-01-001.LIL	1502	Chinese	F	Breast CA 77y
CGS5612-01-001.ABD	1503	Indian	F	Ovarian CA 57y
CGS5794-01-001.LMC	1504	Chinese	F	Breast CA 46y
CGS4964-01-001.VOL	1505	Chinese	F	Breast CA 42y
CGS3708-01-001.RRP	1506	Indian	F	Breast CA 34y
CGS4887-01-001.KYH	1507	Chinese	M	Thyroid CA 5y
CGS5692-01-001.LZX	1508	Chinese	F	Breast CA 20y
CGS0853-01-001.ESK	1509	Others	F	Ovarian CA 44y
CGS5825-01-001.OKA	1510	Chinese	M	Prostate CA 77y
CGS5425-01-001.LKT	1511	Chinese	F	Ovarian CA 66y
CGS5350-01-001.KW	1512	outh Korea	F	Breast lumps
CGS4577-01-001.CTL	1513	Caucasian	F	Breast lumps
CGS5887-01-001.BGS	1514	Caucasian	M	Jewish ancestry; Prostate lumps
CGS5399-01-001.MFA	1515	Caucasian	F	Breast lumps
CGS4644-01-001.NJ	1516	Chinese	F	nic divert - and found to have extensive pancreati
CGS4655-01-001.HWL	1517	Chinese	F	fibroblastic lesion, in keeping with Gardner type f
CGS4981-01-001.TYS	1518	Chinese	M	Clinical dx Neurofibromatosis type 1
CGS5056-01-001.OKP	1519	Chinese	M	Paraganglioma of Glomus Jugulare
CGS5250-01-001.LKW	1520	Chinese	M	Koos 3 vestibular schwannoma + right jugular foramen lesions<br>Monocular diplopia (double vision) in the left lateral gaze
CGS5212-01-001.TYS	1521	Chinese	F	Right upper eyelid neurofibroma<br>Clinical depression
CGS5479-01-001.TCI	1522	Chinese	F	Fibroids
CGS5560-01-001.TN	1523	Indian	F	Fibroid
CGS5313-01-001.CJN	1524	Chinese	F	Breast calcifications
CGS5610-01-001.CCY	1525	Chinese	F	Endometriosis
CGS5749-01-001.NPX	1526	Chinese	F	Desmoid tumour
CGS5756-01-001.AL	1527	Chinese	F	Parkinson's disease<br>Breast lumps
CGS6020-01-001.DHG	1528	Vietnamese	F	Recurrent right sided spontaneous pneumothorax

CGS6043-01-001.SWY	1529	Chinese	F	Multiple thyroid nodules
CGS6221-01-001.SBH	1530	Chinese	M	Atrial fibrillation<br>Right sided unilateral gynaecomastia
CGS6296-01-001.CWS	1531	Chinese	F	Bilateral probably benign breast lesions
CGS6348-01-001.LBT	1532	Filipino	M	Iron overload<br>Fatty liver
CGS4875-01-001.NBS	1533	Malay	F	Benign breast tissue with fibrocystic change
CGS5092-01-001.ASN	1534	Chinese	F	Chronic Kidney Disease Stage 2
CGS4777-01-001.LGK	1535	Chinese	F	Colonic polyps<br>Allergic rhinitis
CGS5476-01-001.TA	1536	Indian	M	Allergic Rhinitis
CGS4749-01-001.TSC	1537	Chinese	M	Skin tags
CGS4769-01-001.ACM	1538	Chinese	F	Benign breast lumps<br>Prolapsed intervertebral disc
CGS4532-01-001.PLN	1539	Chinese	F	Uterine fibroids<br>Adenomyomatosis
CGS4603-01-001.CYY	1540	Chinese	F	Colonic polyps<br>Allergic rhinitis
CGS4987-01-001.SJQ	1541	Chinese	M	Hypoplastic anaemia<br>Low vitamin B12
CGS5798-01-001.CWY	1542	Chinese	M	[illegible]rain tumour, excision: Meningioma, WHO Grade [illegible]
CGS5893-01-001.LHW	1543	Chinese	F	Cervix, mild dysplasia
CGS5624-01-001.KC	1544	Chinese	M	MDS
CGS6130-01-001.MT	1545	Indian	F	Juvenile Granulosa Cell tumour of left ovary<br>Autism spectrum disorder
CGS5811-01-001.TJW	1546	Chinese	F	Neurofibromatosis Type 1<br>Atopic dermatitis
CGS4671-01-001.SW	1547	Indonesian	F	bilateral breast cyst (benign)
