# Supplementary material for: Multi-locus inherited neoplasia alleles syndromes in cancer: implications for clinical practice
Source: Eur J Hum Genet. 2025 Jan 23;33(3):289–96. doi: 10.1038/s41431-025-01785-1 (PMC11894078; doi:10.1038/s41431-025-01785-1)
Supplement: Supplementary file 4 — Supplementary Table 4: MINAS [file 41431_2025_1785_MOESM4_ESM.pdf]

Supplementary Table 4: MINAS

Case	Reference	Ethnicity	Family ID	Sex	PV1 HGVS	PV2 HGVS	PV3 HGVS	Clinical Fx with age dx (if known)
1	Kilmartin et al. 1996	Unknown (paper from Ireland)	1	M	NM_000038.6(APC):c.3340C>T	NM_000551.3(VHL):c.(?_?)del		Retinal haemangioma x2 21y‡; Cerebellar haemangioblastoma 41y‡; Rectal carcinoma and multiple colonic polyps 41y†
2	Ramus et al. 1997	Hungarian	1	F	NM_007294.4(BRCA1):c.66_67delAG	NM_000059.3(BRCA2):c.5946delT		Breast cancer 48y*; Ovarian cancer 50y*
3	Friedman et al. 1998	Ashkenazi Jewish	1	F	NM_007294.4(BRCA1):c.66_67delAG	NM_000059.3(BRCA2):c.5946delT		Breast cancer 38y*
4	Friedman et al. 1998	Ashkenazi Jewish	2	F	NM_007294.4(BRCA1):c.66_67delAG	NM_000059.3(BRCA2):c.5946delT		Ovarian cancer 57y*
5	Friedman et al. 1998	Ashkenazi Jewish	4	F	NM_007294.4(BRCA1):c.66_67delAG	NM_000059.3(BRCA2):c.5946delT		Breast cancer 45y*
6	Randall et al. 1998	Ashkenazi Jewish	1	F	NM_007294.4(BRCA1):c.68_69delAG	NM_000059.3(BRCA2):c.5946delT		Breast cancer, multifocal lobular carcinoma 30y (LOH BRCA1)*; Ovarian cancer 41y (LOH BRCA1 and BRCA2)*
7	Liede et al. 1998	Scottish	1	F	NM_007294.4(BRCA1):c.2389G>T	NM_000059.3(BRCA2):c.3067_306		Breast adenocarcinoma 35y*
8	Tesoriero et al. 1999	Unknown (paper from Australia)	1	F	NM_007294.4(BRCA1):c.3769_3770delGA	NM_000059.3(BRCA2):c.5946delT		Breast cancer <40y (LOH BRCA2)*
9	Borg et al. 2000	Swedish	1	F	NM_007294.4(BRCA1):c.3047_3048in	NM_000249.3(MLH1):c.131C>T		Breast invasive ductal carcinoma 35y (MSI low. ERPR -ve)†
10	Bell et al. 2002	Ashkenazi Jewish	1	F	NM_007294.4(BRCA1):c.5266dupC	NM_000059.3(BRCA2):c.5946delT		Breast cancer 33y (LOH BRCA2. No LOH BRCA1)‡; Breast cancer 44y (LOH BRCA2. No LOH BRCA1)‡; Breast cancer 47y (LOH BRCA1. No LOH BRCA2)†

11	de la Hoya et al., 2002	Spanish	1	M	NM_007294.4(BRCA1):c.5123C>A	NM_000059.3(BRCA2):c.6275_627		Prostate cancer 66y*
12	de la Hoya et al., 2002	Spanish	1	F	NM_007294.4(BRCA1):c.5123C>A	NM_000059.3(BRCA2):c.6275_627		Breast cancer 70y*
13	de la Hoya et al., 2002	Spanish	1	F	NM_007294.4(BRCA1):c.5123C>A	NM_000059.3(BRCA2):c.6275_627		Breast cancer 66y*
14	de la Hoya et al., 2002	Spanish	1	F	NM_007294.4(BRCA1):c.5123C>A	NM_000059.3(BRCA2):c.6275_627		Breast cancer 28y (No LOH BRCA1 or BRCA2)*
15	Leegte et al. 2005 Frank et al. 2002	Unknown (Paper from the Netherlands)	Unknown	F	NM_007294.4(BRCA1):c.66_67delAG	NM_000059.3(BRCA2):c.5946delT		Breast cancer 39y*
16	Leegte et al. 2005 Frank et al. 2002	Unknown (Paper from the Netherlands)	Unknown	F	NM_007294.4(BRCA1):c.66_67delAG	NM_000059.3(BRCA2):c.5946delT		Breast cancer 41y*
17	Leegte et al. 2005 Frank et al. 2002	Unknown (Paper from the Netherlands)	Unknown	F	NM_007294.4(BRCA1):c.66_67delAG	NM_000059.3(BRCA2):c.5946delT		Breast cancer. Bilateral 34y*
18	Leegte et al. 2005 Frank et al. 2002	Unknown (Paper from the Netherlands)	Unknown	F	NM_007294.4(BRCA1):c.66_67delAG	NM_000059.3(BRCA2):c.5946delT		Breast cancer 55y*;<br>Breast cancer (contralateral) 56y*
19	Leegte et al. 2005 Frank et al. 2002	Unknown (Paper from the Netherlands)	Unknown	F	NM_007294.4(BRCA1):c.66_67delAG	NM_000059.3(BRCA2):c.5946delT		Breast cancer 40y*

20	Leegte et al. 2005<br>Frank et al. 2002	Unknown (Paper from the Netherlands)	Unknown	F	NM_007294.4(BRCA1):c.66_67delAG	NM_000059.3(BRCA2):c.5946delT		Breast cancer 33y*;<br>Breast cancer (contralateral) 49y*
21	Scheenstra et al. 2003	Dutch	1	M	NM_000038.6(APC):c.3927_3931del	NM_000249.3(MLH1):c.677G>A		Multiple colon polyps (100's) 10†;<br>Tubular adenomas with dysplasia 10y (Loss of MLH1 on IHC)*
22	Choi et al. 2004	Korean	1	F	NM_007294.4(BRCA1):c.4981G>T	NM_000059.3(BRCA2):c.5946_594		Breast cancer 33y*
23	Choi et al. 2004	Korean	2	F	NM_007294.4(BRCA1):c.1516_1520d	NM_000059.3(BRCA2):c.2798_279		Breast cancer 26y*
24	Choi et al. 2004	Korean	3	F	NM_007294.4(BRCA1):c.1656_1656d	NM_000059.3(BRCA2):c.4599A>C		Breast cancer 37y*
25	Thiffault et al. 2004	Unknown (Paper from Canada)	1	F	NM_007294.4(BRCA1):c.314T>G	NM_000251.2(MSH2):c.1277_1386 del		Lobular and ductal carcinoma in situ 32y (ERPR +ve)†;<br>Endometroid adenocarcinoma 40y (IHC MMR normal. MSI low)Δ;<br>Colon villotubular adenoma. 40 (Loss of MSH2 on IHC. MSI high)‡
26	Valle et al. 2004	Spanish	1	F	NM_000038.6(APC):c.540dup	NM_000314.4(PTEN):c.634+5G>A		Multiple colonic polyps 10y‡; Subcutaneous nodules‡;<br>Multinodular goitre 26y†; Papillary thyroid cancer, multiple nodular hyperplasia and follicular adenomas 26y†; Diffuse lymphocytic chronic thyroiditis†; Ovarian Morgani hidatide 15yΔ; Cerebellar dysplastic gangliocytoma 26y†; Palmar keratosis 26y†; Head fibroma 26y†; Lipomas 26y†; Melanocytic neavi x2 28y†; Facial papules 28y†; Oral papillomatosis 28y†

27	Soravia et al. 2005	Italian	1	M	NM_000038.6(APC):c.3471-3474delGAGA	NM_000251.2(MSH2):c.1192dupG		Colon polyps x5. 4 adenomas 24y (1 dysplastic MSI high. Loss of MSH2 and MSH6 on IHC)†; Colon adenocarcinoma. Right colon 25y*; Gastric/duodenal adenoma x30 25y*; Desmoid tumour. Mesenteric 26y†
28	Leegte et al. 2005	Dutch	1	F	NM_007294.4(BRCA1):c.2685_2686delAA	NM_000059.3(BRCA2):c.3487delG		Ovarian papillary serous cystadenocarcinoma 40y (LOH BRCA2)*; Breast infiltrative ductal carcinoma 45y (LOH BRCA1)*
29	Leegte et al. 2005	Dutch	2	F	NM_007294.4(BRCA1):c.2685_2686d	NM_000059.3(BRCA2):c.4449delA		Breast cancer. Ductal 28y*
30	Leegte et al. 2005	Ashkenazi Jewish	4	F	NM_007294.4(BRCA1):c.5263_5264in	NM_000059.3(BRCA2):c.5946delT		Breast invasive lobular carcinoma 51y*
31	Musolino et al. 2005	Italian	1	F	NM_007294.4(BRCA1):c.4285_4286in	NM_000059.3(BRCA2):c.7738C>T		Breast infiltrating duct carcinoma 37y (Triple negative histology)*
32	Choi et al. 2006	Korean	1	F	NM_007294.4(BRCA1):c.1504_1508d	NM_000059.3(BRCA2):c.2798_279		Breast infiltrating duct carcinoma 26y*
33	Choi et al. 2006	Korean	2	F	NM_007294.4(BRCA1):c.4981G>T	NM_000059.3(BRCA2):c.5946delT		Breast infiltrating duct carcinoma 33y*
34	Manoukian et al. 2007	Italian	1	F	NM_000059.3(BRCA2):c.7180A>T	NM_000546.6(TP53):c.847C>T		Breast cancer 31y*; Breast cancer 66y*; Leiomyosarcoma of chest wall 71y (breast radiotherapy field)‡
35	Monnerat et al. 2007	Italian	1	F	NM_000059.3(BRCA2):c.4889C>G	NM_000546.6(TP53):c.329G>T		Cutaneous malignant melanoma 65y‡; Breast cancer 69y*; Ovarian cancer 69y*; Colon cancer 74y‡
36	Ghataorhe et al. 2007	Unknown (paper from the United Kingdom)	1	F	NM_000059.3(BRCA2):c.2808_2811delACAA	NM_130799.2(MEN1):c.1064+1delGT		Abnormal secretory parathyroid gland 34y‡; Pancreatic mass. Unknown histology. Non functional 35y*

37	Ghataorhe et al. 2007	Unknown (paper from the United Kingdom)	1	F	NM_000059.3(BRCA2):c.2808_2811delACAA	NM_130799.2(MEN1):c.1064+1delGT		Cushing syndrome (implied pituitary origin) 10y‡; Hypercalcaemia (implied hyperparathyroidism) 31y‡
38	Ghataorhe et al. 2007	Unknown (paper from the United Kingdom)	1	M	NM_000059.3(BRCA2):c.2808_2811delACAA	NM_130799.2(MEN1):c.1064+1delGT		Parathyroid hyperplasia 56y‡; Breast cancer 60y†
39	Zbuk et al. 2007	Unknown (paper from USA)	1	F	NM_000314.4(PTEN):c.47dup	NM_003001.3(SDHC):c.397C>T		Macrocepahly†; Papillomatous papules†; Paraganglioma. Left common carotid 18y‡; Fibrocystic breast disease 20s†; Papillary thyroidcancer 37y†; Paraganglioma. Right carotid body 39y‡; Uterine leiomyomas 30s†
40	van Puijenbroek et al. 2007	Unknown (Paper from the Netherlands)	1	F	NM_000179.3(MSH6):c.1784delT	NM_001128425.1(MUTYH):c.536A>G	NM_001128425.1(MUTYH):c.1187G>A	Colon adenomas x5 48y (All MSI stable. Retained MSH6 expression)‡
41	Plon et al. 2008	Unknown (paper from USA)	1	F	NM_000314.4(PTEN):c.334C>G	NM_000546.6(TP53):c.844C>T		Neuroblastoma 0y‡; Lipoma. Abdominal wall 0y†; Haemangiomas 1y†; Macrocephaly†; Ovarian granulosa cell tumour 1y (No somatic PTEN or TP53 mutations. LOH PTEN . No LOH TP53)Δ; Xanthoastrocytoma. Temporal lobe 3y (No somatic PTEN or TP53 mutations. No LOH PTEN or TP53)Δ; Pelvic liposarcoma 4y (No somatic PTEN or TP53 mutations. LOH PTEN . No LOH TP53)Δ

42	Uhrhammer & Bignon. 2008	French	1	M	NM_000038.6(APC):c.3183_3187delA	NM_000251.2(MSH2):c.255_256del		Colon cancer 16y*
43	Smith et al. 2008	Unknown (paper from Australia)	1	F	NM_007294.4(BRCA1):c.3331_3334delCAAG	NM_000059.3(BRCA2):c.631+2T>G		Breast cancer 34y*; Colorectal carcinoma. Transverse. (Normal MMR IHC. MSI-L) 35yΔ; Breast cancer 53y*
44	Foppiani et al. 2008	Unknown (paper from Italy)	1	M	NM_000077.4(CDKN2A):c.142C>A	NM_020975.4(RET):c.2410G>A		Cutaneous malignant melanoma <55y‡; Parathyroid chief cell adenoma 55y†; Thyroid sclerotic papillary carcinoma 55y†; Thyroid cell hyperplasia 55y†
45	Pilato et al. 2010	Italian	1	F	NM_007294.4(BRCA1):c.5263_5264dup	NM_000059.3(BRCA2):c.5796_5797delTA		Breast intraductal carcinoma 38y (Triple negative histology)*; Ovarian papillary adenocarcinoma. Bilateral 42y*
46	Zuradelli et al. 2010	Italian	1	F	NM_007294.4(BRCA1):c.835delC	NM_000059.3(BRCA2):c.8195T>G		Breast carcinoma. Metaplastic 43y (Triple negative histology)*
47	Zuradelli et al. 2010	Italian	2	F	NM_007294.4(BRCA1):c.3916_3917delTT	NM_000059.3(BRCA2):c.5380delG		Breast ductal cancer. Medullary type 30y (ERPRve)*; Ovarian serous papillary carcinoma 36y*
48	Zuradelli et al. 2010	Italian	3	F	NM_007294.4(BRCA1):c.1687C>T	NM_000059.3(BRCA2):c.6469C>T		Breast infiltrating duct carcinoma 2x foci 46y (1 lymph node ERPR -ve. 1 lymph node ERPR+ve)*
49	Zuradelli et al. 2010	Italian	4	F	NM_007294.4(BRCA1):c.2405_2406delTG	NM_000059.3(BRCA2):c.4285C>T		Breast ductal carcinoma 52y (Triple negative histology)*; Ovarian serous adenocarcinoma.Bilateral 52y*
50	Steffensen et al. 2010	Danish	1	F	NM_007294.4(BRCA1):c.5096G>A	NM_000059.3(BRCA2):c.631+4A>G		Breast cancer 53y*; Ovarian cancer 59y*
51	Steffensen et al. 2010	Danish	1	M	NM_007294.4(BRCA1):c.5096G>A	NM_000059.3(BRCA2):c.631+4A>G		Breast cancer* 76y

52	Mastroianno et al. 2011	Italian	1	M	NM_130799.2(MEN1):c.669+1G>T	NM_020975.4(RET):c.1997A>T		Pituitary tumour 38y‡; Primary hyperparathyroidism 45y*; Papillary thyroid cancer 46yΔ; Medullary thyroid cancer 46y†; Gastric carcinoid tumour 47y‡; Gastrinoma‡
53	Mastroianno et al. 2011	Italian	1	F	NM_130799.2(MEN1):c.669+1G>T	NM_020975.4(RET):c.1997A>T		Primary hyperparathyroidism 13y*; Pituitary tumour 15y*
54	Augustyn et al. 2011	German	1	F	NM_007294.4(BRCA1):c.1961delA	NM_000059.3(BRCA2):c.1672delC		Ovarian serous carcinoma with papillary features. Bilateral 50y*
55	Augustyn et al. 2011	Ashkenazi Jewish	2	F	NM_007294.4(BRCA1):c.5266dupC	NM_000059.3(BRCA2):c.4829_483		Breast cancer 40y (Triple negative histology)*
56	Noh et al. 2011	Korean	1	F	NM_007294.4(BRCA1):c.3746_3747in	NM_000059.3(BRCA2):c.6952_695		Breast infiltrating duct carcinoma 26y*
57	Noh et al. 2011	Korean	2	F	NM_007294.4(BRCA1):c.390C>A	NM_000059.3(BRCA2):c.3018delA		Breast infiltrating duct carcinoma 45y*
58	Noh et al. 2011	Korean	3	F	NM_007294.4(BRCA1):c.5030_5033d	NM_000059.3(BRCA2):c.1399A>T		Breast infiltrating duct carcinoma 35y*
59	Kashiwada et al. 2012	Japanese	1	F	NM_000038.6(APC):c.637C>T	NM_144997.5(FLCN):c.1285dup		Facial papules <28y‡; Colon carcinoma and multiple colon polyps 28y†; Recurrent pneumothoraces x4. Pulmonary cysts 28y (first one)‡

60	Lindor et al. 2012	Mixed ancestry (from Kashmir, Egypt and Saudi Arabia)	1	M	NM_000038.6(APC):c.694C>T	NM_000249.3(MLH1):c.1732_2271del		Rectal carcinoma and multiple colon polyps 14y*;<br>Jejunal adenocarcinoma x6 28y x3, 34y, 44y, 52y (Loss of MLH1 and PMS2 on IHC)*;<br>Duodenal adenocarcinoma 54y*;<br>Congenital hypertrophy of retinal pigment epithelium 54y†;<br>Squamous cell carcinoma. Multiple facialΔ;<br>Pilomatricoma. Scalp 54y†;<br>Sebaceous adenoma 54y (Loss of MLH1 and PMS2 on IHC)‡
61	Pern et al. 2012	German	1	F	NM_007294.4(BRCA1):c.927delA	NM_024675.3(PALB2):c.756dup		Uterine myomas <65yΔ; Meningioma<65yΔ;<br>Breast invasive ductal carcinoma. Multifocal 65y (Triple negative histology)*
62	Heidemann et al. 2012	German	1	F	NM_007294.4(BRCA1):c.5266dup	NM_000059.3(BRCA2):c.5645C>G		Breast cancer 37y*;<br>Breast cancer 39y*;<br>Ovarian cancer 63y*
63	Heidemann et al. 2012	German	2	F	NM_007294.4(BRCA1):c.68_69delAG	NM_000059.3(BRCA2):c.5718_571		Breast cancer 32y*
64	Heidemann et al. 2012	German	3	F	NM_007294.4(BRCA1):c.962G>A	NM_000059.3(BRCA2):c.2231C>G		Breast cancer 31y*;<br>Breast cancer (contralateral) 35y*
65	Heidemann et al. 2012	German	4	F	NM_007294.4(BRCA1):c.3910delG	NM_000059.3(BRCA2):c.2830A>T		Breast cancer 39y*
66	Heidemann et al. 2012	German	5	F	NM_007294.4(BRCA1):c.5277+1delG	NM_000059.3(BRCA2):c.658_659d		Colorectal cancer. Caecal 58yΔ;<br>Ovarian cancer 61y*
67	Heidemann et al. 2012	German	6	F	NM_007294.4(BRCA1):c.3700_3704d	NM_000059.3(BRCA2):c.1813_181		Cervical cancer 26yΔ;<br>Breast cancer 40y*
68	Loubser et al. 2012	Afrikaner	1	F	NM_007294.4(BRCA1):c.2641G>T	NM_000059.3(BRCA2):c.7934delG		Breast ductal carcinoma 42y*

69	Gong et al. 2012	Unknown (paper from USA)	1	F	NM_000059.3(BRCA2):c.5946delT	NM_000249.3(MLH1):c.677G>T	NM_000179.3(MSH6):c.3959_396	Endometrial cancer 41yo
70	Kast et al. 2012	German	1	F	NM_007294.4(BRCA1):c.213-12A>G	NM_000179.3(MSH6):c.515dup		Endometrial endometroid adenocarcinoma 46y (Loss of MSH6 on IHC)‡
71	Pedroni et al. 2013	Italian	1	F	NM_007294.4(BRCA1):c.300T>G	NM_000249.3(MLH1):c.1489dupC		Breast cancer 35y (Loss of MLH1 on IHC . LOH MLH1 & BRCA1)†;<br>Endometrial carcinoma (Loss of MLH1 on IHC. LOH MLH1)‡; Ovarian carcinoma 39y (Loss of MLH1 on IHC. LOH MLH1)*;<br>Renal clear cell carcinoma 39yΔ;<br>Breast cancer (contralateral) 46y (Loss of MLH1 on IHC. LOH and BRCA1)‡
72	Campos et al. 2013	Spanish	1	F	NM_007294.4(BRCA1):c.4107_4110dup	NM_000267.3(NF1):c.4120C>T		Café au lait patches; multiple cutaneous; neurofibromas;‡<br>Axillary/inguinal freckling in childhood‡;<br>Breast infiltrating duct carcinoma 35y*
73	Sokolenko et al. 2014	Russian	BC297	F	NM_007294.4(BRCA1):c.5266dup	NM_007194.3(CHEK2):c.5395del		Breast cancer 52yo‡ T1N1M0 G1 ER+PR+Her2- LOH at CHEK2; no LOH at BRCA1
74	Sokolenko et al. 2014	Russian	BC1408	F	NM_007294.4(BRCA1):c.3247del5	NM_007194.3(CHEK2):c.5395del		Breast cancer 42yo‡
75	Sokolenko et al. 2014	Russian	BC960	F	NM_000057.4(BLM):c.1642C>T	NM_007294.4(BRCA1):c.5266dup		Ovarian cancer 50yo†;<br>Breast cancer 68 yo† T2N0M0 G2 ER-PR-HER2- No LOH at BRCA1 and BLM
76	Sokolenko et al. 2014	Russian	BC1444	F	NM_000057.4(BLM):c.1642C>T	NM_007294.4(BRCA1):c.5266dup		Waldenstrom disease (lymphoplasmacytic lymphoma) 58yoΔ<br>Breast cancer 67yo† T2N0M0 G3 ER-PR-HER2- No LOH at BRCA1 and BLM
77	Sokolenko et al. 2014	Russian	BC828	F	NM_000057.4(BLM):c.1642C>T	NM_007194.3(CHEK2):c.1100delC		Breast cancer 51yo† T2N0M0 G2 ER+PR+HER2- No LOH at CHEK2 and BLM

78	Sokolenko et al. 2014	Russian	BC1672	F	NM_000057.4(BLM):c.1642C>T	NM_002485.4(NBN):c.657_661del5		Breast cancer 48yo† T2N0Mx G2 ER-PR+HER2+ No LOH at at NBS1 and BLM
79	Sokolenko et al. 2014	Byelorussian	N324	F	NM_000057.4(BLM):c.1642C>T	NM_007294.4(BRCA1):c.5266dup		Breast cancer 44yo† T3N3M1 Gx ER+
80	Sokolenko et al. 2014	Byelorussian	N757	F	NM_000051.4(ATM):c.5932G>T	NM_007294.4(BRCA1):c.181T>G		Breast cancer 40yo* T2N1M0 Gx ER-
81	Sokolenko et al. 2014	Byelorussian	N996	F	NM_000051.4(ATM):c.5932G>T	NM_007294.4(BRCA1):c.181T>G		Breast cancer 42yo* T2N1M0 G2 ER-
82	Sokolenko et al. 2014	Byelorussian	N1312	F	NM_000057.4(BLM):c.1642C>T	NM_007294.4(BRCA1):c.5266dup		Breast cancer 45yo† T2aNxMx G1 ER-
83	Sokolenko et al. 2014	Byelorussian	N2018	F	NM_000051.4(ATM):c.5932G>T	NM_007194.3(CHEK2):c.5395del		Breast cancer 67yo*
84	Sokolenko et al. 2014	Byelorussian	N2041	F	NM_000057.4(BLM):c.1642C>T	NM_007194.3(CHEK2):c.5395del		Breast cancer 44yo†
85	Sokolenko et al. 2014	Byelorussian	N2365	F	NM_007294.4(BRCA1):c.181T>G	NM_007194.3(CHEK2):c.1100delC		Breast cancer 58yo* G1 ER+
86	Sokolenko et al. 2014	Byelorussian	N2412	F	NM_007194.3(CHEK2):c.444+1G>A	NM_002485.4(NBN):c.657del5		Breast cancer 53yo*
87	Sokolenko et al. 2014	Polish	52181	F	NM_007194.3(CHEK2):c.444+1G>A	NM_002485.4(NBN):c.657del5		Bilateral breast cancer 59/60yo* ER+PR+HER2+
88	Sokolenko et al. 2014	Polish	25456	F	NM_007194.3(CHEK2):c.444+1G>A	NM_002485.4(NBN):c.657del5		Breast cancer 53yo* ER+PR
89	Sokolenko et al. 2014	Polish	57561	F	NM_007294.4(BRCA1):c.5266dup	NM_007194.3(CHEK2):c.444+1G>A		Breast cancer 54yo* ER-PR-HER2-
90	Ercolino et al. 2014	Caucasian	1	F	NM_000267.3(NF1):c.1185+1G>A	NM_020975.4(RET):c.2410G>A		Macrocephaly, café au lait patches and axillary freckling 57y†;<br>Kyphoscoliosis 57y†; Multiple cutaneous neurofibromas 57y†; Thyroid Cell hyperplasia 57y‡;<br>Parathyroid hyperplasia 57y‡

91	Bell et al. 2014	Unknown (paper from Canada)	1	F	NM_007294.4(BRCA1):c.81-?_134+?del	NM_000546.6(TP53):c.375+2T>C		Metaplastic breast carcinoma 20yo‡; Breast IDC 20yo‡; both were triple negative
92	Yang et al. 2015	Chinese	7	F	NM_000059.3(BRCA2):c.5699C>G	NM_033084.3(FANCD2):c.4234_4239delAGTGAG		Breast cancer 30y†
93	Yang et al. 2015	Chinese	22	F	NM_007294.4(BRCA1):c.2110_2111delAA	NM_001128425.1(MUTYH):c.850-2A>G	NM_002439.3(MSH3):c.162_179d	Breast ca 37yo†
94	Yang et al. 2015	Chinese	24	F	NM_000059.3(BRCA2):c.7142delC	NM_000553.4(WRN):c.4245dupT		Breast ca 26yo; 36yo†
95	Yang et al. 2015	Chinese	29	F	NM_007294.4(BRCA1):c.5503C>T	NM_012452.3(TNFRSF13B):c.102d		Breast ca 39yo‡
96	Yang et al. 2015	Chinese	75	F	NM_002439.3(MSH3):c.2305delG	NM_001137669.2(RGSL1):c.1357C		Breast ca 51yoΔ
97	Yang et al. 2015	Chinese	77	F	NM_001195132.2(CDKN2A):c.480G>A	NM_005732.3(RAD50):c.1291_1295delGAGAT		Breast ca 34yo†
98	Yang et al. 2015	Chinese	86	F	NM_058216.2(RAD51C):c.343dupG	NM_032444.2(SLX4):c.3583_3585d		Breast ca 52yo†
99	Nomizu et al. 2015	Japanese	1	F	NM_007294.4(BRCA1):c.188T>A	NM_000059.3(BRCA2):c.5576_557		Breast cancer 55y*
100	Whitworth et al. 2016	Unknown (paper from the United Kingdom)	1	M	NM_144997.5(FLCN):c.1062+2T>G	NM_000267.3(NF1):c.1381C>T		Testicular seminoma 39yΔ;<br>Renal cell carcinoma. Chromophobe 55y†;<br>Recurrent pneumothoraces.†<br>Phaeochromocyto ma 43y‡;<br>Gastrointestinal stromal tumour x2 55y‡;<br>Malignant peripheral nerve sheath tumour 56y‡;<br>Multiple cutaneous neurofibromas‡;<br>Cafe au lait patches‡;

101	Whitworth et al. 2016	Unknown (paper from the United Kingdom)	2	M	NM_144997.5(FLCN):c.715C>T	NM_000546.6(TP53):c.526T>C		Rectal carcinoma 27yΔ;<br>Gastroesophageal adenocarcinoma 32yΔ;<br>Renal cell carcinoma. Chromophobe 32y†;<br>Facial fibrofolliculomas†
102	Whitworth et al. 2016	Unknown (paper from the United Kingdom)	3	F	NM_144997.5(FLCN):c.1285delC	NM_000251.2(MSH2):c.892C>T		Pneumothorax 37y†;<br>Endometrial cancer 52y‡
103	Whitworth et al. 2016	Indian	4	M	NM_000249.3(MLH1):c.306G>T	NM_000380.4(XPA):c.555+8A>G		Caecal cancer. Mucinous 65y†;<br>Sigmoid cancer 67y†;<br>Previous skin tumours including squamous carcinoma in an actinic keratosis, multiple seborrhoeic keratoses, keratoacanthomata/ squamous carcinomas x2, junctional naevi, squamous carcinoma and lentigo malignae x2‡
104	Whitworth et al. 2016	Unknown (paper from the United Kingdom)	5	F	NM_000059.3(BRCA2):c.5213_5216del	NM_000267.3(NF1):c.6792C>G		Breast ductal carcinoma 48y*;<br>Cutaneous melanoma 57y†;<br>Multiple cutaneous neurofibromas‡;<br>Malignant peripheral nerve sheath tumour‡;<br>Café au lait patch‡;<br>Possible L ischnodules‡
105	Meynard et al. 2017	French	1	F	NM_007294.4(BRCA1):c.1016dupA	NM_000059.3(BRCA2):c.6814del		Bilateral breast cancer 46y*
106	Spannuth et al. 2007	Ashkenazi Jewish	1	F	NM_007294.4(BRCA1):c.5266dup	NM_000059.3(BRCA2):c.5946delT		Right breast cancer IDC 46y*;<br>Ovarian cancer Stage IV serous 57y*
107	Njoroge et al. 2017	European (Scottish, Norwegian, German)	1	F	NM_004360.3(CDH1):c.2287G>T	NM_000535.4(PMS2):c.2445+1G>T		Breast invasive lobular carcinoma 51y*(ER+ PR+ Her2-)†;<br>Thyroid follicular adenoma 52yΔ;<br>Thyroid papillary microcarcinoma 52yΔ;<br>Colonic polyp 65‡

108	Silva-Smith et al. 2017	Hispanic	1	M	NM_004329.2(BMPR1A):c.25A>T	NM_000535.4(PMS2):c.1882C>T		Bladder cancer. High grade transitional cell carcinoma 39y‡<br>Colon cancer. Rectosigmoid colon adenocarcinoma (IHC loss of PMS2) 39y*
109	Stradella et al. 2019	Unknown (Paper from Spain)	1	F	NM_000038.6(APC):c.423-3T>A	NM_007294.4(BRCA1):c.1961delA		Colonic polyps 76y†
110	Stradella et al. 2019	Unknown (Paper from Spain)	2	F	NM_130799.2(MEN1):c.784-9G>A	NM_000249.3(MLH1):c.244A>G		Neuroendocrine tumour 41y†;<br>Pituitary tumour 36y†;<br>Parathyroid 39y†;<br>Parathyroid Hyperplasia 29y†;<br>Hepatic haemangiomasΔ
111	Stradella et al. 2019	Unknown (Paper from Spain)	2	F	NM_130799.2(MEN1):c.784-9G>A	NM_000249.3(MLH1):c.244A>G		Endometrial carcinoma 41y‡;<br>Colorectal cancer 48y‡;<br>Haemangiomas 45yΔ;<br>Hyperparathyroidism 45y†
112	Stradella et al. 2019	Unknown (Paper from Spain)	3	F	NM_007294.4(BRCA1):c.3607C>T	NM_000546.6(TP53):c.659A>G		Ovarian cancer 45y†
113	Stradella et al. 2019	Unknown (Paper from Spain)	4	F	NM_002878.3(RAD51D):c.694C>T	NM_000548.4(TSC2):c.5227C>T		Subcutaneous benign tumours 6y†;<br>Epilepsy†; Intellectual disability†
114	Stradella et al. 2019	Unknown (Paper from Spain)	5	F	NM_000465.3(BARD1):c.157delT	NM_000143.3(FH):c.905-2A>G		Cutaneous leiomyomas 40y‡
115	Stradella et al. 2019	Unknown (Paper from Spain)	6	M	NM_000038.6(APC):c.5826_5829del	NM_130398.4(EXO1):c.1900C>T		Colonic polyps 52y†;<br>Colorectal cancer 53y‡
116	Stradella et al. 2019	Unknown (Paper from Spain)	7	F	NM_007294.4(BRCA1):c.2309C>A	NM_000380.4(XPA):c.553C>T		Ovarian cancer 51y†

117	Stradella et al. 2019	Unknown (Paper from Spain)	7	F	NM_007294.4(BRCA1):c.2309C>A	NM_000380.4(XPA):c.553C>T		Ovarian cancer 37y†
118	Stradella et al. 2019	Unknown (Paper from Spain)	8	F	NM_000122.2(ERCC3):c.325C>T	NM_144997.5(FLCN):c.346C>T		Ovarian cancer 66y†; recurrent pneumothorax (x5) 33y‡
119	Stradella et al. 2019	Unknown (Paper from Spain)	9	F	NM_000051.4(ATM):c.3802delG	NM_024675.3(PALB2):c.3256C>T		Breast cancer 54y*; Pancreatic cancer 59y*
120	Stradella et al. 2019	Unknown (Paper from Spain)	10	F	NM_007194.3(CHEK2):c.433C>T	NM_007194.3(CHEK2):c.470T>C		Bilateral breast cancer 35y*
121	Stradella et al. 2019	Unknown (Paper from Spain)	11	F	NC_000022.11.(?_?)del	NM_007194.3(CHEK2):c.499G>A		Breast cancer 42y*
122	Stradella et al. 2019	Unknown (Paper from Spain)	12	F	.(?_?)del	NM_000135.2(FANCA):c.2602-1G>C		Breast cancer 35y†
123	Stradella et al. 2019	Unknown (Paper from Spain)	13	F	NM_000135.2(FANCA):c.3558dupG	NM_003000.2(SDHB):c.505C>T		Ovarian cancer 49y
124	Tang et al. 2018	Unknown (Paper from USA)	1	M	NM_001308093.3(GATA4):c.778C>T	NM_000314.4(PTEN):c.517C>T		Left ventricular noncompaction cardiomyopathy 48y†<br>Macrocephaly‡<br>6mm colonic adenoma‡
125	Tang et al. 2018	Unknown (Paper from USA)	1	M	NM_001308093.3(GATA4):c.778C>T	NM_000314.4(PTEN):c.517C>T		LVNC 19y†<br>Macrocephaly‡
126	Palmirotta et al. 2018	Italian	1	F	NM_007294.4(BRCA1):c.1687C>T	NM_000059.3(BRCA2):c.9976A>T		Right breast cancer 40y and 54y*; Left breast cancer 47y*; Melanoma 54y‡

127	Le Page et al. 2018	French Canadian	1	F	NM_007294.4(BRCA1):c.4327C>T	NM_000059.3(BRCA2):c.3170del		Ovarian cancer 40y*
128	Le Page et al. 2018	French Canadian	2	F	NM_007294.4(BRCA1):c.4327C>T	NM_000059.3(BRCA2):c.2816insA		Breast cancer 52y*; Ovarian cancer 63y*
129	Agiannitopoulos et al. 2019	Greek	1	F	NM_000251.2(MSH2):c.1661+1_1662-1-(*1_?)	NM_024675.3(PALB2):c.757_758delCT		Endometrial cancer 42y† (IHC expression MSH2: 5%; MLH1: 80%; MSH6: 30%; PMS2: 60%)
130	Andres et al. 2019	Spanish	1	F	NM_000051.4(ATM):c.2413C>T	NM_007294.4(BRCA1):c.5123C>A		Breast cancer, triple negative 55y*; Ampulloma pT2N0M0 62y‡; Endometrial cancer, clear cell, HG Stage IIIC 71yΔ
131	Sorscher et al. 2019	Unknown (paper from USA)	1	F	NM_000059.3(BRCA2):c.1310_1313del	NM_000249.3(MLH1):c.1381A>T		Acute myelogenous leukemia 26yΔ Squamous cell CA of vulva 51y‡ Breast Intraductal CA 52y†
132	Vietri et al. 2020	Italian	1	F	NM_007294.4(BRCA1):c.547+2T>A	NM_000059.3(BRCA2):c.2830A>T		Bilateral breast cancer 32y*
133	Vietri et al. 2020	Italian	1	F	NM_007294.4(BRCA1):c.547+2T>A	NM_000059.3(BRCA2):c.2830A>T		Breast cancer 39y*; Ovarian cancer 46y*
134	Vietri et al. 2020	Italian	2	F	NM_007294.4(BRCA1):c.3752_3755G	NM_000059.3(BRCA2):c.425+2T>C		Ovarian cancer 36y*
135	Vietri et al. 2020	Italian	2	M	NM_007294.4(BRCA1):c.3752_3755G	NM_000059.3(BRCA2):c.425+2T>C		Colorectal cancer 40yΔ
136	Hur et al. 2020	Korean	1	F	NM_007294.4(BRCA1):c.390C>A	NM_000059.3(BRCA2):c.5576_557		Breast cancer IDC triple negative 43y*
137	Hur et al. 2020	Korean	2	F	NM_007294.4(BRCA1):c.922_924deli	NM_000059.3(BRCA2):c.3599_360		Breast cancer IDC T2N0M0 triple negative 36y*
138	Hur et al. 2020	Korean	3	F	NM_007294.4(BRCA1):c.5496_5506d	NM_000059.3(BRCA2):c.7480C>T		Bilateral breast cancer 31y* R IDC T2N0M0; L DCIS TisN0 both ER/PR+ Her2-
139	Hur et al. 2020	Korean	4	F	NM_007294.4(BRCA1):c.5030_5033del	NM_000059.3(BRCA2):c.1399A>T		L breast cancer triple negative pT1N0M0 35y*; R breast cancer triple negative pT1N0M0 41y*;

140	Teker et al. 2020	Unknown (paper from Turkey)	1	F	NM_000051.4(ATM):c.5065C>T	NM_000059.3(BRCA2):c.537dup		Breast cancer 34yo* IDC+ILC ER+PR+<br>Pancreatic cancer 48yo*
141	Teresa Vietri et al. 2020	Italian	1	F	NM_000038.6(APC):c.3927_3931delA	NM_007294.4(BRCA1):c.3756_375		Profuse FAP 18y†; Desmoid tumor 22y†;<br>Ovarian cancer HGS 45y‡
142	Laish et al. 2021	Ashkenazi Jewish	1	M	NM_007294.4(BRCA1):c.185delAG	NM_000251.2(MSH2):c.1906G>C		Bladder CA 42y‡<br>TA-LGD < 10 mm × 5 (62–74)‡
143	Laish et al. 2021	Ashkenazi Jewish	2	M	NM_000059.3(BRCA2):c.6174del	NM_000179.3(MSH6):c.3984_3987dupGTCA		Ascending Colon adenocarcinoma 76y‡<br>TA-LGD < 10 mm × 3 70y‡<br>Glioblastoma multiforme 91y†
144	Laish et al. 2021	Ashkenazi Jewish	2	F	NM_000059.3(BRCA2):c.6174del	NM_000179.3(MSH6):c.3984_3987dupGTCA		Breast IDC 49y; 59y†<br>Non-Hodgkin's lymphoma 60yΔ<br>T-cell Cutaneous SCC 63y‡<br>Melanoma 66y†
145	Laish et al. 2021	Ashkenazi Jewish	2	F	NM_000059.3(BRCA2):c.6174del	NM_000179.3(MSH6):c.3984_3987		Endometrial Endometrioid adeno-CA 53y‡
146	Laish et al. 2021	Ashkenazi Jewish	3	M	NM_000059.3(BRCA2):c.6174del	NM_000179.3(MSH6):c.3956_3957		GIST Stromal tumor 62yΔ<br>TA-LGD > 10 mm × 1 65y
147	Laish et al. 2021	Ashkenazi Jewish	3	F	NM_000059.3(BRCA2):c.6174del	NM_000179.3(MSH6):c.3956_3957		Breast Ductal carcinoma in situ 40y†
148	Laish et al. 2021	Ashkenazi Jewish	4	M	NM_000059.3(BRCA2):c.6174del	NM_000251.2(MSH2):c.1906G>C		Melanoma cancer 65y†<br>Prostate cancer 72y†<br>Bladder Urothelial cancer 72y‡
149	Laish et al. 2021	Ashkenazi Jewish	4	F	NM_000059.3(BRCA2):c.6174del	NM_000251.2(MSH2):c.1906G>C		Breast Intraductal adeno-CA 36y†<br>Breast Intraductal adeno-CA 42y†

150	Sukumar et al. 2021	Caucasian	1	F	NM_007294.4(BRCA1):c.181T>G	NM_000059.3(BRCA2):c.4398_4402delACATT	NM_007194.3(CHEK2):c.1100delC	chronic hepatitis C infection, cirrhosis, coronary artery<br>disease, hyperthyroidism<br>L breast cancer ER-PR+HEr2- 1A (pT1cN0) dx 55yo;<br>R IDC incidentally found ER/PR- Her2-
151	Ferrer-Avargues et al. 2021	Unknown (paper from Spain)	1	M	NM_000059.3(BRCA2):c.3492dupT	NM_000249.3(MLH1):c.1717_1718delGT		Colon CA 49y‡
152	Ferrer-Avargues et al. 2021	Unknown (paper from Spain)	1	F	NM_000059.3(BRCA2):c.3492dupT	NM_000249.3(MLH1):c.1717_1718delGT		Colon CA 43y‡<br>Ovarian CA 53†<br>Also a third PV in NBN gene
153	Ferrer-Avargues et al. 2021	Unknown (paper from Spain)	2	F	NM_007294.4(BRCA1):c.5152+5G>A	NM_000249.3(MLH1):c.701delA		Colon CA 59y‡<br>Endometrial CA 60y‡
154	Ferrer-Avargues et al. 2021	Unknown (paper from Spain)	3	F	NM_000051.4(ATM):c.6711_6715delGGAAA	NM_000251.2(MSH2):c.2633_2634delAG		Colorectal cancer 39y‡<br>Endometrial cancer 52y‡
155	Ferrer-Avargues et al. 2021	Unknown (paper from Spain)	4	M	NM_000179.3(MSH6):c.762dupT	NM_000267.3(NF1):c.5129_5141del		Colorectal cancer 37y†
156	Ferrer-Avargues et al. 2021	Unknown (paper from Spain)	5	F	NM_000135.2(FANCA):c.1115_1118delTTGG	NM_000249.3(MLH1):c.1731G>A		Colorectal cancer 40y‡
157	Belanger et al. 2015	French Canadian	-	F	NM_007294.4(BRCA1):c.3770_3771d	NM_000059.3(BRCA2):c.5946del		Ovarian cancer 41y*
158	Koren-Michowitz et al. 2005	Ashkenazi Jewish	2	F	NM_007294.4(BRCA1):c.5382insC	NM_000059.3(BRCA2):c.6174delT	NM_000057.4(BLM):c.2207_2212	Breast cancer* 45y

159	Koren-Michowitz et al. 2005	Ashkenazi Jewish	1	F	NM_007294.4(BRCA1):c.5382insC	NM_000059.3(BRCA2):c.6174delT		Breast cancer* 32y
160	Na et al. 2017	European American	23	M	NM_007294.4(BRCA1):c.5266dup	NM_000059.3(BRCA2):c.5946del		Prostate cancer (localised) 57y* Gleason score 6
161	Vietri et al. 2013	Italian	1	F	NM_007294.4(BRCA1):c.547+2T>A	NM_000059.3(BRCA2):c.2830A>T	NM_000059.3(BRCA2)	Bilateral breast cancer* 32y ER/PR+ Her2-
162	Vietri et al. 2014	Italian	1	F	NM_007294.4(BRCA1):c.547+2T>A	NM_000059.3(BRCA2):c.2830A>T	NM_000059.3(BRCA2)	Breast cancer* 39y
163	Claus et al. 2004	Mixed European	2	F	NM_007294.4(BRCA1):c.962G>A	NM_000059.3(BRCA2):c.3170_317		Breast cancer* 37y
164	Nomizu et al. 2015	Japanese	1	F	NM_007294.4(BRCA1):c.188T>A	NM_000059.3(BRCA2):c.5576_557		Breast cancer* 41y Endometrial cancerΔ 46y
165	Tedaldi et al. 2017	Paper from Italy		F	NM_007294.4(BRCA1):c.2049_2050d	NM_000059.3(BRCA2):c.5266dupC		Breast cancer 53y* IDC
166	Tedaldi et al. 2017	Paper from Italy		F	NM_000059.3(BRCA2):c.6998dupT	NM_000135.2(FANCA):c.987_990d		Breast cancer 30y†
167	Tedaldi et al. 2017	Paper from Italy		F	NM_000059.3(BRCA2):c.658_659del	NM_032043.2(BRIP1):c.2992_2995d		Breast cancer x2 IDC 37y†
168	Tedaldi et al. 2017	Paper from Italy		F	NM_000051.4(ATM):c.3275C>A	NM_000059.3(BRCA2):c.8487+1G>		Breast DCIS 38y* Breast IDC 45y*
169	Tedaldi et al. 2017	Paper from Italy		F	NM_007294.4(BRCA1):c.5266dupC	NM_000122.2(ERCC3):c.1757delA		Breast IDC 39y*
170	Nurmi et al. 2019	Finnish		F	NM_020937.4(FANCM):c.5101C>T	NM_024675.3(PALB2):c.1592delT		Breast cancer triple pos 33y*
171	Nurmi et al. 2019	Finnish		F	NM_020937.4(FANCM):c.5101C>T	NM_024675.3(PALB2):c.1592delT		Breast cancer 55y TNBC*
172	Nurmi et al. 2019	Finnish		F	NM_007194.3(CHEK2):c.1100delC	NM_007194.3(CHEK2):c.319+2T>A		Breast cancer 53y*
173	Nurmi et al. 2019	Finnish		F	NM_007194.3(CHEK2):c.1100delC	NM_007194.3(CHEK2):c.444+1G>A		Bilateral breast cancer 38y; 68y*

174	Nurmi et al. 2019	Finnish		F	NM_000051.4(ATM):c.6908dupA	NM_007194.3(CHEK2):c.1100delC		Breast cancer 53y*<br>Bladder cancer 58yΔ
175	Nurmi et al. 2019	Finnish		F	NM_000051.4(ATM):c.7570G>C	NM_007194.3(CHEK2):c.1100delC		Breast cancer 60y*
176	Nurmi et al. 2019	Finnish		F	NM_007194.3(CHEK2):c.1100delC	NM_020937.4(FANCM):c.5101C>T		Breast cancer 46y†
177	Nurmi et al. 2019	Finnish		F	NM_007194.3(CHEK2):c.1100delC	NM_020937.4(FANCM):c.5101C>T		Breast cancer 87y†
178	Nurmi et al. 2019	Finnish		F	NM_000051.4(ATM):c.6908dupA	NM_020937.4(FANCM):c.5101C>T		Ovarian cancer 48y†
179	Nurmi et al. 2019	Finnish		F	NM_000051.4(ATM):c.7570G>C	NM_020937.4(FANCM):c.5791C>T		Bilateral breast cancer 28y;30y†
180	Nurmi et al. 2019	Finnish		F	NM_020937.4(FANCM):c.5101C>T	NM_020937.4(FANCM):c.5791C>T		Breast cancer 43y*
181	Nurmi et al. 2019	Finnish		F	NM_000051.4(ATM):c.6908dupA	NM_007294.4(BRCA1):c.3485delA	NM_058216.2(RAD51	Ovarian cancer 60y*
182	Nurmi et al. 2019	Finnish		F	NM_000051.4(ATM):c.6908dupA	NM_007294.4(BRCA1):c.3485delA		Ovarian cancer 53y*
183	Nurmi et al. 2019	Finnish		F	NM_007294.4(BRCA1):c.3485delA	NM_020937.4(FANCM):c.5101C>T		Ovarian cancer 42y†
184	Nurmi et al. 2019	Finnish		F	NM_007294.4(BRCA1):c.4097-2A>G	NM_020937.4(FANCM):c.5101C>T		Breast cancer 60y†
185	Nurmi et al. 2019	Finnish		F	NM_007294.4(BRCA1):c.4327C>T	NM_007194.3(CHEK2):c.1100delC		Bilateral breast cancer 49y; 55y*
186	Nurmi et al. 2019	Finnish		F	NM_007294.4(BRCA1):c.4327C>T	NM_020937.4(FANCM):c.5101C>T		Breast cancer 37y†<br>Ovarian cancer 40y†
187	Nurmi et al. 2019	Finnish		F	NM_007294.4(BRCA1):c.5266dupC	NM_020937.4(FANCM):c.5791C>T		Breast cancer 48y†
188	Nurmi et al. 2019	Finnish		F	NM_000051.4(ATM):c.7570G>C	NM_000059.3(BRCA2):		Breast cancer 76y*

189	Nurmi et al. 2019	Finnish		F	NM_000059.3(BRCA2):c.7480C>T	NM_024675.3(PALB2):c.1592delT		Bilateral breast cancer 38y; 52y*
190	Nurmi et al. 2019	Finnish		F	NM_000059.3(BRCA2):c.7480C>T	NM_007194.3(CHEK2):c.1100delC	NM_020937.4(FANCM	Breast cancer 55y*
191	Nurmi et al. 2019	Finnish		F	NM_000059.3(BRCA2):c.7480C>T	NM_007194.3(CHEK2):c.1100delC		Breast cancer 61y*
192	Nurmi et al. 2019	Finnish		F	NM_000059.3(BRCA2):c.7480C>T	NM_020937.4(FANCM):c.5791C>T		Breast cancer 41y† Ovarian cancer 55y†
193	Nurmi et al. 2020	Finnish	a	F	NM_000059.3(BRCA2):c.8327T>G	NM_020937.4(FANCM):c.5101C>T		Breast cancer 36y†
194	Nurmi et al. 2021	Finnish	a	F	NM_000059.3(BRCA2):c.8327T>G	NM_020937.4(FANCM):c.5101C>T		Bilateral breast cancer 53y; 77y† Ovarian cancer 82y†
195	Nurmi et al. 2022	Finnish		F	NM_000059.3(BRCA2):c.8947dupG	NM_024675.3(PALB2):c.1592delT		Breast cancer 46y*
196	Nurmi et al. 2023	Finnish		F	NM_000059.3(BRCA2):c.9118-2A>G	NM_020937.4(FANCM):c.5101C>T		Bilateral breast cancer 53y; 59y†
197	Ouyang et al. 2019	Chinese	1	F	NM_007294.4(BRCA1):c.5030_5033d	NM_000059.3(BRCA2):c.631+1G>A		TNBC 35y*
198	Ouyang et al. 2019	Chinese	2	F	NM_007294.4(BRCA1):c.1387A>T	NM_024675.3(PALB2):c.751C>T		TNBC 35y*
199	Ouyang et al. 2019	Chinese	3	F	NM_007294.4(BRCA1):c.5095C>T	NM_024675.3(PALB2):c.1059del		TNBC 42y*
200	Ouyang et al. 2019	Chinese	4	F	NM_000059.3(BRCA2):c.8474_8487d	NM_024675.3(PALB2):c.472del		Pancreatic cancer 48y*
201	Ouyang et al. 2019	Chinese	5	F	NM_000051.4(ATM):c.-30-1G>A	NM_007294.4(BRCA1):c.2269delG		TNBC 47y*
202	Ouyang et al. 2019	Chinese	6	F	NM_007294.4(BRCA1):c.1016del	NM_000179.3(MSH6):c.1483C>T		Ovarian cancer 43y*
203	Ouyang et al. 2019	Chinese	7	F	NM_000059.3(BRCA2):c.3577delC	NM_000535.4(PMS2):c.2140C>T		TNBC 64y*

204	Ouyang et al. 2019	Chinese	8	F	NM_000051.4(ATM):c.1507G>T	NM_000535.4(PMS2):c.1A>G		Colorectal ca 67y†
205	Cote et al. 2012	French Canadian		F	NM_000059.3(BRCA2):c.9004G>A	NM_024675.3(PALB2):c.2323C>T		Breast cancer 45y*
206	Momozaw et al. 2018	Japanese		F	NM_000051.4(ATM):c.72+1G>A	NM_000059.3(BRCA2):c.2813delC		Breast IDC 45y* ER/PR+ Her2-
207	Momozaw et al. 2018	Japanese		F	NM_000059.3(BRCA2):c.9382C>T	NM_007194.3(CHEK2):c.573+1delG		Breast IDC 56y* ER/PR+ Her2?
208	Momozaw et al. 2018	Japanese		F	NM_000059.3(BRCA2):c.7586del	NM_007194.3(CHEK2):c.1567G>A		Breast IDC 44y* ER/PR+ Her2?
209	Sarkadi et al. 2019	Paper from Hungary	1	F	NM_007294.4(BRCA1):c.269_281delTTTGTGCTTTTCA	NM_020975.4(RET):c.1902C>G		Medullary Thyroid Carcinoma 16y‡
210	Fostira et al. 2019	Greek		F	NM_007294.4(BRCA1):c.5497G>A	NM_024675.3(PALB2):c.2257C>T		Breast cancer 44y*
211	Fostira et al. 2019	Greek		F	NM_000059.3(BRCA2):c.2490_2491in	NM_058216.2(RAD51C):c.904+5G>		Breast cancer 46y*; 56y*
212	Fostira et al. 2019	Greek		F	NM_000059.3(BRCA2):c.7879A>T	NM_003001.3(SDHC):c.397C>T		Bilateral breast cancer 31y†
213	Fostira et al. 2019	Greek		F	NM_007294.4(BRCA1):c.5406 + 664_*8273del	NM_000059.3(BRCA2):c.9748dupT		TNBC 31y*; breast cancer 49y*
214	Fostira et al. 2019	Greek		F	NM_000059.3(BRCA2):c.1813dupA	NM_004260.4(RECQL4):c.1879-		Breast cancer 46y†<br>Ovarian cancer 63y†
215	Fostira et al. 2019	Greek		F	NM_000051.4(ATM):c.3576G>A	NM_000059.3(BRCA2):c.(80+1_81-1)_(593+1_594-		Breast cancer 40y*
216	Fostira et al. 2019	Greek		F	NM_000122.2(ERCC3):c.576_583delCGTGATCC	NM_000136.2(FANCC):c.346-1G>A		TNBC 34y†

217	Fostira et al. 2019	Greek		F	NM_024675.3(PALB2):c.2747_2748+4delAGGTAA	NM_032444.2(SLX4):c.4089_4090delAG		Breast cancer 44y†<br>Ovarian cancer 48y†
218	Fostira et al. 2019	Greek		F	NM_000051.4(ATM):c.1562_1563delA	NM_024675.3(PALB2):c.2257C>T		Breast cancer 33y*
219	Njoroge et al. 2017	Paper from USA		F	NM_004360.3(CDH1):c.2287G>T	NM_000535.4(PMS2):c.2445+1G>T		LBC 51y†<br>Follicular adenoma(thyroid)Δ<br>Micropapillary thyroid carcinoma 52yΔ
220	Vahteristo et al. 2001	Finnish		F	NM_007194.3(CHEK2):c.1100del	NM_000179.3(MSH6):c.2983G>T		Breast cancer 34y*<br>Colorectal cancer 34y†
221	Brown et al. 2020	Mexican/Spanish		M	NM_130799.2(MEN1):c.525_526insT	NM_020975.4(RET):c.1889G>A		Medullary Thyroid Carcinoma 27y‡
222	Pearlman et al. 2017	Paper from USA		F	NM_000251.2(MSH2):c.2388delT	NM_001128425.1(MUTYH):c.1187G		Colorectal cancer 30y†; MMR deficient
223	Pearlman et al. 2017	Paper from USA		F	NM_000051.4(ATM):c.7271T>G	NM_007194.3(CHEK2):c.1100del		Colorectal cancer 40y‡; MMR proficient
224	Pearlman et al. 2017	Paper from USA		F	NM_000059.3(BRCA2):c.1755_1759d	NM_007194.3(CHEK2):c.751A>T		Colorectal cancer 47y‡; MMR proficient
225	Pearlman et al. 2017	Paper from USA		M	NM_000038.6(APC):c.2377C>T	NM_000535.4(PMS2):c.1281delT		Polyposis 29y†
226	Pearlman et al. 2017	Paper from USA		F	NM_000249.3(MLH1):c.2252_2253d	NM_000251.2(MSH2):c.80C>T		Colorectal cancer 28y; 46y; MMR deficient*
227	Yamamoto et al. 2006	Japanese	1	M	Data unavailable	NM_002834.3(PTPN11):c.182A>G		ALL 5y*; survival > 154 months
228	Ng et al. 2016	Asian	1	F	NM_000465.3(BARD1):c.1487C>G	NM_000059.3(BRCA2):c.5164_546		Breast cancer 35y* ER/PR+Her2-
229	Papi et al. 2009	Paper from Italy	1	F	NM_007294.4(BRCA1):c.3228_3229delAG	NM_130799.2(MEN1):c.908delGCT		2x insulinoma 33y‡<br>Mediastinal lipoma 35yΔ<br>hyperparathyroidism 32y‡<br>Hyperprolactinemia 33y‡

230	Sekido et al. 2017	Japanese	1	F	Data unavailable	NM_000222.2(KIT):c.1670G>T	NM_000222.2(KIT):c.1	BC† + GIST‡ 65y; IHC = Trip Neg
231	Balta et al. 2019	Paper from Turkey	1	M	NM_000051.4(ATM):c.8977C>T	NM_000135.2(FANCA):c.1374delC	NM_000135.2(FANCA):c.1374delC	Child of 2 individuals above<br>Fanconi anaemia phenotype*<br>Pancytopenia and macrocytosis 9y*
232	Balta et al. 2019	Paper from Turkey	1	F	NM_000051.4(ATM):c.8977C>T	NM_000135.2(FANCA):c.1374delC	NM_000135.2(FANCA):c.1374delC	Child of 2 individuals above<br>Fanconi anaemia phenotype*<br>Pancytopenia and macrocytosis 8y*
233	Balta et al. 2019	Paper from Turkey	1	F	NM_000051.4(ATM):c.8977C>T	NM_000051.4(ATM):c.8977C>T	NM_000135.2(FANCA):c.1374delC	Child of 2 individuals above<br>Ataxia telangiectasia phenotype 12*<br>No FA
234	Stajkovska et al. 2019	North Macedonian	1	M	NM_000249.3(MLH1):c.333_334delT	NM_000546.6(TP53):c.847C>T		Glioblastoma 13y‡
235	Genomics England	Unknown		F	NM_000059.3(BRCA2):c.6276_6278d	NM_007194.3(CHEK2):c.470T>A		Breast ILC & IDC 25-30y*
236	Genomics England	Unknown		F	NM_000059.3(BRCA2):c.6276_6278d	NM_007194.3(CHEK2):c.470T>A		Breast adenocarcinoma 45-50y*
237	Genomics England	Unknown		F	NM_000051.4(ATM):c.8988_8989deli	NM_000057.4(BLM):c.2207C>T		Breast tubular adenocarcinoma 45-50y†
238	Genomics England	Unknown		F	NM_000051.4(ATM):c.8988_8989deli	NM_000057.4(BLM):c.2207C>T		Breast adenocarcinoma 45-50y*
239	Genomics England	Unknown		M	NM_000059.3(BRCA2):c.6276_6278d	NM_002691.3(POLD1):c.952G>C		Paraganglioma 40-45yΔ
240	Genomics England	Unknown		M	NM_000123.4(ERCC5):c.2186_2191d	NM_000380.4(XPA):c.649_650delin		Pitutary adenoma 40-45y
241	Carbajal-Mamani et al. 2020	white Caucasian	1	F	NM_000051.4(ATM):(?_?)del (exon 62_63del)	NM_024675.3(PALB2):c.2840T>C		46y benign fibroids<br>Breast DCIS 49y*; IHC = ER+, PR+<br>OC 50y* (clear cell)

242	Ataei-Kachouei et al. 2015	Arab	1	F	NM_000059.3(BRCA2):c.2808_2811d	NM_000455.4(STK11):c.1264A>G		Met Breast IDC 38y ; IHC = ER+, PR+, HER2?*
243	Occhi et al. 2010	Italian		F	NM_003977.3(AIP):c.911G>A	NM_130799.2(MEN1):c.133G>C		Acromegaly 30yΔ
244	Yilmaz et al. 2020	Paper from Turkey	1	M	NM_000179.3(MSH6):c.3261delC	NM_000535.4(PMS2):c.187G>A		Colon cancer 63y*
245	Nakahara et al. 1997	Japanese		M	NM_000249.3(MLH1):c.397G>T	NM_000251.2(MSH2):c.1916A>G		Colon cancer 41y*<br>Stomach cancer 41y*
246	Nakahara et al. 1997	Japanese		F	NM_000249.3(MLH1):c.397G>T	NM_000251.2(MSH2):c.1916A>G		multiple CRCs + uterine cancer 57y*
247	Nakahara et al. 1997	Japanese		F	NM_000249.3(MLH1):c.397G>T	NM_000251.2(MSH2):c.1916A>G		multiple CRCs + uterine cancer 55y*
248	Taeubner et al. 2017	Paper from Germany	1	M	NM_000264.3(PTCH1):c.113G>A	NM_003738.3(PTCH2):c.1864C>T		Congenital embryonal rhabdomyosarcomaΔ<br>No tumour loss of heterozygosity (LOH) in PTCH1 and PTCH2
249	Kamory et al. 2006	Paper from Hungary	1	M	NM_000249.3(MLH1):c.2146G>A	NM_000251.2(MSH2):c.2210+1G>C		Multiple synchronous colorectal carcinoma 25y*<br>IHC = loss of MLH1 and MSH2
250	Frank-Raue et al. 2005	Paper from Germany	1	M	NM_130799.2(MEN1):c.825-1G>A	NM_020975.4(RET):c.2372A>T		Primary hyperparathyroidism*<br>Pancreatic tumor 35y*<br>Fibroma 35yΔ
251	Frank-Raue et al. 2005	Paper from Germany	1	M	NM_130799.2(MEN1):c.825-1G>A	NM_020975.4(RET):c.2372A>T		Primary hyperparathyroidism*<br>Pitutary tumour*<br>Pancreatic tumour*<br>FibromaΔ<br>brother of above
252	Lorca et al. 2019	Paper from Spain	1	F	NM_002528.2(NTHL1):c.527T	NM_006231.3(POLE):c.3857G>A		Adenomatous polyposis 70y*<br>Endometrial hyperplasia 70y*<br>Hypothyroidism 70yΔ
253	Wen et al. 2019	Chinese	1	M	NM_007294.4(BRCA1):c.5521del	NM_000059.3(BRCA2):c.5656C>T		Gastric cancer 50yΔ

254	Stolavora et al. 2020	Czech		F	NM_007194.3(CHEK2):c.909-2028_1095+330del5395	NM_015450.2(POT1):c.347C>T		Melanoma 41y; 42;y 44y† Breast cancer 47y‡
255	Stolavora et al. 2020	Czech		M	NM_001252024.2(TRPM1):(?_?)del (exons 2_7del)	NM_000550.3(TYRP1):c.1054_1057del4		Melanoma 36y*
256	Stolavora et al. 2020	Czech		M	NM_016180.5(SLC45A2):(?_?)del (exons 1_2del)	NM_000849.5(GSTM3):c.393C>A		Melanoma 25y*
257	Stolavora et al. 2020	Czech		F	NM_000136.2(FANCC):c.455dupA	NM_000550.3(TYRP1):c.1037-7T>A		Breast cancer 52y† Melanoma 66y* Colorectal cancer 66yΔ
258	Stolavora et al. 2020	Czech		F	NM_002485.4(NBN):c.1723G>T	NM_004556.3(NFKBIE):c.165_169d		Melanoma 9y*
259	Stolavora et al. 2020	Czech		M	NM_000059.3(BRCA2):c.7007G>A	NM_022168.4(IFIH1):c.2464C>T		Melanoma 22y*
260	Stolavora et al. 2020	Czech		M	NM_000059.3(BRCA2):c.8168_8172in	NM_000550.3(TYRP1):c.1254C>A		Melanoma 36y* Non-Hodgkin Lymphoma 38yΔ
261	Stolavora et al. 2020	Czech		F	NM_000051.4(ATM):c.7630-2A>C	NM_007294.4(BRCA1):c.4214delT	NM_001128425.1(MUTYH):c.118	Melanoma 46yΔ Ovarian cancer 46y* Breast cancer 49y*
262	Stolavora et al. 2020	Czech		F	NM_000051.4(ATM):c.381delA	NM_000553.4(WRN):c.1105C>T		Melanoma 41yΔ Melanoma 50yΔ
263	Stolavora et al. 2020	Czech		F	NM_007194.3(CHEK2):c.917G>C	NM_002878.3(RAD51D):c.405+2T>		Melanoma 26yΔ
264	Zheng et al. 2020	Paper from China		F	NM_007294.4(BRCA1):c.3348_3351delAGTT	NM_000251.2(MSH2):c.(?_?)del (exons 4_16del)		Endometrial carcinoma 52y‡

265	Pazderová et al. 2020	Paper from Slovak Republic		M	NM_000059.3(BRCA2):c.8487G>T	NM_007194.3(CHEK2):c.(?_?)del (exons 9_10del)		Pancreatic Cancer 50y*
266	Sukumar et al. 2021	Caucasian	2	F	NM_007294.4(BRCA1):c.181T>G	NM_007194.3(CHEK2):c.1100delC		Breast cancer 34y* ER+ PR- Her2-
267	Le Duc et al. 2020	Paper from Germany	1	M	NM_058216.2(RAD51C):(?_?)del (exons 5_9del)	NM_000546.6(TP53):c.394A>G		Basal Cell Carcinoma 38y† Sebaceous Gland Carcinoma 41y†
268	Del Valle et al. 2020	Paper from Spain		F	Unknown	NM_000135.2(FANCA):c.2602-		Breast cancer 35y*
269	Del Valle et al. 2020	Paper from Spain		F	NM_000135.2(FANCA):c.3588dup	NM_003000.2(SDHB):c.(?_?)		Ovarian cancer 49yΔ
270	Del Valle et al. 2020	Paper from Spain		M	NM_018062.3(FANCL):c.1111_1114d	NM_000249.3(MLH1):c.(?_?)		Colorectal cancer 29‡
271	Le Page et al. 2020	?	3	F	NM_007294.4(BRCA1):c.3916-	NM_000059.3(BRCA2):c.5380delG		Breast cancer 30y*; Ovarian cancer 36y*
272	Le Page et al. 2020	Slovenian	4	F	NM_007294.4(BRCA1):c.1687C>T	NM_000059.3(BRCA2):c.6469C>T		Breast cancer 46y*; Ovarian cancer 58y*
273	Le Page et al. 2020	?	5	F	NM_007294.4(BRCA1):c.2405_2406d	NM_000059.3(BRCA2):c.4285C>T		Breast cancer 52y*; Ovarian cancer 52y*
274	Le Page et al. 2020	Ashkenazi	9	F	NM_007294.4(BRCA1):c.68_69delAG	NM_000059.3(BRCA2):c.5946delT		Ovarian cancer 57y*
275	Le Page et al. 2020	Ashkenazi	10	F	NM_007294.4(BRCA1):c.68_69delAG	NM_000059.3(BRCA2):c.5946delT		Ovarian cancer 50y*
276	Le Page et al. 2020	Ashkenazi	24	F	NM_007294.4(BRCA1):c.68_69delAG	NM_000059.3(BRCA2):c.5946delT		Breast cancer 48y*; Ovarian cancer 50y*
277	Suspitsin et al. 2013	Paper from Russia		F	NM_000057.4(BLM):c.1642C>T	NM_007294.4(BRCA1):c.5266dup		TNBC 68y*
278	Suspitsin et al. 2013	Paper from Russia		F	NM_000057.4(BLM):c.1642C>T	NM_007294.4(BRCA1):c.5266dup		TNBC 68y*

279	Suspitsin et al. 2013	Paper from Russia		F	NM_000057.4(BLM):c.1642C>T	NM_007194.3(CHEK2):c.1100delC		Breast cancer 51y *IHC = ER+,PR+,HER2-
280	Suspitsin et al. 2013	Paper from Russia		F	NM_007294.4(BRCA1):c.5266dup	NM_007194.3(CHEK2):c.5395del		Breast cancer 52y* IHC = ER+,PR+,HER2-
281	Suspitsin et al. 2013	Paper from Russia		F	NM_000057.4(BLM):c.1642C>T	NM_002485.4(NBN):c.657_661del		Breast cancer 48y* IHC = ER-,PR+,HER2+
282	Penkert et al. 2018	German		F	NM_000051.4(ATM):c.8793T>A	NM_007194.3(CHEK2):c.(?_?)del (exons 9_10del)		Breast cancer 39y*
283	Penkert et al. 2018	German		F	NM_058195.3(CDKN2A):c.292C>T	NM_004260.4(RECQL4):c.1390+1G		Breast cancer 32yΔ IHC = HER2+
284	Penkert et al. 2018	German		F	NM_001113378.1(FANCI):c.3853C>T	NM_000535.4(PMS2):c.(?_?)del (exons 3_8del)		Breast cancer 30yΔ IHC = HER2+
285	Ohmoto et al. 2018	Japanese		M	NM_007194.3(CHEK2):c.1111C>T	NM_000535.4(PMS2):c.631 C> T p.Arg211Ter		Colorectal cancer 58y*
286	Infante et al. 2022	Spanish	678	F	NM_007294.4(BRCA1):c.34C>T	NM_000059.3(BRCA2):c.1587delTi		Breast cancer 48y* IHC = ER-,PR-
287	Infante et al. 2022	Spanish	776	F	NM_000059.3(BRCA2):c.5146_5149d	NM_000535.4(PMS2):c.903G>T		Breast cancer 40y† IHC = ER/Pr+, Her2-<br>Colorectal cancer 54y‡
288	Infante et al. 2022	Spanish	3699	F	NM_007294.4(BRCA1):c.-	NM_000059.3(BRCA2):c.5796_579		Breast cancer 45y* IHC = ER-,PR-
289	Infante et al. 2022	Spanish	C1423	F	NM_007294.4(BRCA1):c.4165_4166d	NM_058216.2(RAD51C):c.709C>T		HGS Ovarian cancer 81y*
290	Caldes et al. 2002	Spanish	1	F	NM_007294.4(BRCA1):c.5123C>A	NM_000059.3(BRCA2):c.6275_627		Breast cancer 28y*
291	Caldes et al. 2002	Spanish	1	M	NM_007294.4(BRCA1):c.5123C>A	NM_000059.3(BRCA2):c.6275_627		Prostate cancer 66y*
292	Caldes et al. 2002	Spanish	1	F	NM_007294.4(BRCA1):c.5123C>A	NM_000059.3(BRCA2):c.6275_627		Breast cancer 70y*

293	Caldes et al. 2002	Spanish	1	F	NM_007294.4(BRCA1):c.5123C>A	NM_000059.3(BRCA2):c.6275_627		Breast cancer 66y*
294	Mastroianno et al. 2011	Italian	1	M	NM_130799.2(MEN1):c.669+1G>T	NM_020975.4(RET):c.1997A>T		Primary hyperparathyroidism 40y*<br>Cushing syndrome 40y<br>Carcinoid 40y*<br>Lipoma 40y*<br>Angiofibroma 40y*<br>Papillary thyroid cancer 40yΔ<br>Medullary thyroid cancer 40y†<br>Gastrinoma 41y‡
295	This article	Chinese	1	F	NM_007194.3(CHEK2):c.1459C>T	NM_000249.3(MLH1):c.793C>T		Breast cancer 47y† R IDC ER/PR+ Her2-
296	This article	Chinese	2	F	NM_000492.3(CFTR):c.1210-34TG[12]T[5]	NM_002769.4(PRSS1):c.86A>T		Pancreatic cancer 52y mucinous adenocarcinoma*;<br>Chronic pancreatitis 20-30y*
297	This article	Malay	3	F	NM_007294.4(BRCA1):c.2726dup	NM_001128425.1(MUTYH):c.325C>		Ovarian cancer 40y HGS†
298	This article	Malay	4	F	NM_007294.4(BRCA1):c.2726dup	NM_001128425.1(MUTYH):c.1518+2del (splice site)		Tonsil SCC 49yΔ
299	This article	Chinese	5	F	NM_000465.3(BARD1):c.69_70delins25	NM_000535.4(PMS2):c.(?_?)del (exons 1_6del)		Breast cancer 35y† L IDC ER/PR+ Her2-
300	This article	Chinese	6	F	NM_000059.3(BRCA2):c.7480C>T	NM_018062.3(FANCL):c.933T>A		Breast cancer 33y† R IDC ER/PR+ Her2+<br>Acute myleoid leukemia 13y‡<br>Polycystic ovarian syndromeΔ<br>L parasagittal meningomaΔ
301	This article	Malay	7	F	NM_000267.3(NF1):c.4537C>T	NM_000546.6(TP53):c.(?_?)del (promoter region)		Clinical dx NF1†<br>Temporal diffuse astrocytoma 3y*<br>Optic nerve glioma†<br>MPNST 8y†

302	This article	Malay	8	F	NC_000002.12.(?_?)del	NM_000251.2(MSH2):c.(?_?)del (exons 1_6del)		Likely a single deletion event spanning *EPCAM* and *MSH6*<br>Urothelial ca 47*<br>Endometroid endometrial ca 54* loss of MSH2/MSH6 proteins
303	This article	Chinese	9	F	NM_002354.2(EPCAM):(?_?)del (exon 4del)	NM_000251.2(MSH2):c.(?_?)del (exon 1del)		Endometrial ca 52y*<br>Ovarian ca 52y*<br>loss of MSH2/MSH6 proteins
304	This article	Malay	10	M	):c.3927_3931delAAAGA	1(MUTYH):c.1435G>T		Multiple colonic polyps (TA) 18y*;<br>Multiple fundic gland polyps 18y*
305	Colombo et al. 2023	Paper from Italy	1	F	NM_000051.4(ATM):c.1672G>T	RCA1):c.5406+6T>C		Triple negative breast ca 36y†
306	Tsaousis et al. 2022	Paper from Greece		F	NM_007294.4(BRCA1):c.181T>G	NM_007194.3(CHEK2):c.470T>C		Breast cancer 43y*
307	Mampel et al. 2021	Argentinean	1	F	NM_007294.4(BRCA1):c.4201C>T	RCA2):c.5146_5149del		Breast IDC 45y*<br>Breast IDC 65y*
308	Vogelaar et al. 2021	Paper from USA	1	F	NM_000251.2(MSH2):c.2006G>T	NM_000179.3(MSH6):c.3936_4001+8dup		multiple colorectal polyps 50y<br>multiple gastric polyps 55y<br>duodenal advanced adenoma 55y*
309	Schamschula et al. 2022	Paper from Germany	1	M	NM_000535.4(PMS2):c.2007-786_2174+493del1447	NM_002691.3(POLD1):c.946G>A		Rectal carcinoma 17y*<br>Multiple adenomas 17y*<br>Colon cancer 27y*<br>urothelial carcinoma 34y*<br>Bladder adenoma 36y*
310	Schamschula et al. 2023	Paper from Germany	1	F	NM_000535.4(PMS2):c.2007-786_2174+493del1448	NM_002691.3(POLD1):c.946G>A		Cecal & colon ascendens carcinoma 19y*

311	Michaeli et al. 2021	Paper from Israel	1	M	NM_000535.4(PMS2):c.2148dupC	NM_006231.3(POLE):c.830A>G		Cafe au lait macule 4.5yΔ<br>Desmoplastic medulloblastoma 4.5yΔ
312	Agaoglu et al. 2021	Paper from Turkey	P1	F	A1):c.5102_5103delTG	1(MUTYH):c.884C>T		Bilateral breast cancer 33y; 38y†
313	Agaoglu et al. 2021	Paper from Turkey	P2	F	NM_000179.3(MSH6):c.3226C>T	NM_002485.4(NBN):c.966C>G		Breast cancer 42y; 45y*
314	Agaoglu et al. 2021	Paper from Turkey	P3	F	A1):c.3700_3704del	AD50):c.326_329del		HGS Ovarian cancer 60y*
315	Agaoglu et al. 2021	Paper from Turkey	P4	M	NM_000059.3(BRCA2):c.1189C>T	1(MUTYH):c.884C>T		Breast cancer 44y†<br>Colon cancer 53y‡
316	Agaoglu et al. 2021	Paper from Turkey	P5	F	NM_007194.3(CHEK2):c.1427C>T	1(MUTYH):c.884C>T		Colon cancer 55y*
317	Agaoglu et al. 2021	Paper from Turkey	P6	F	NM_000051.4(ATM):c.8582T>C	NM_007294.4(BRCA1):c.788dupG		Bilateral breast cancer 45y*
318	Agaoglu et al. 2021	Paper from Turkey	P7	F	NM_000051.4(ATM):c.3802del	NM_000059.3(BRCA2):c.3751dupA		Lobular breast cancer 33y*
319	Agaoglu et al. 2021	Paper from Turkey	P8	F	NM_004360.3(CDH1):c.187C>T	NM_007194.3(CHEK2):c.1427C>T	NM_001128425.1(MUTYH):c.117	Gastric cancer 51y†<br>Lobular breast cancer 56y*<br>Ovarian cysts 56yΔ
320	Agaoglu et al. 2021	Paper from Turkey	P9	F	NM_000059.3(BRCA2):c.8754+1G>T	NM_007194.3(CHEK2):c.1100del		Breast cancer 29y*
321	Agaoglu et al. 2021	Paper from Turkey	P10	F	NM_000059.3(BRCA2):c.3199del	NM_002485.4(NBN):c.657_661del		Breast cancer 60y*<br>HGS endomterial 66yΔ
322	Agaoglu et al. 2021	Paper from Turkey	P11	F	NM_000051.4(ATM):c.8395_8404del	NM_001128425.1(MUTYH):c.1187G		Breast cancer 30y†
323	Torrezan et al. 2023 (LOVD database)	Brazil	MINAS_01	M	NM_000248.3(MITF):c.952G>A	NM_024675.3(PALB2):c.355del		Liposarcoma 29yΔ
324	Torrezan et al. 2023 (LOVD database)	Brazil	MINAS_02	F	NM_014967.2(FAN1):c.1571del	NM_000249.3(MLH1):c.1896+280_		Colon cancer 47y‡

325	Torrezan et al. 2023 (LOVD database)	Brazil	MINAS_03	F	NM_000051.4(ATM):c.5644C>T	NM_000321.3(RB 1):c.1421G>T		Retinoblastoma 11months‡; Meningioma 11monthsΔ; Leiomyosarcoma 11months‡
326	Torrezan et al. 2023 (LOVD database)	Brazil	MINAS_04	F	NM_000122.2(ERC C3):c.1588C>T	NM_001128425.1 (MUTYH):c.504+1 9_504+31del		Neuroendocine tumour of appendix 28yΔ
327	Torrezan et al. 2023 (LOVD database)	Brazil	MINAS_05	F	NM_130799.2(ME N1):c.1664G>A	NM_004260.4(RE CQL4):c.2547_25		Pancreatic neuroendocrine tumour 40y† Papillary thyroid carcinoma 40y†
328	Torrezan et al. 2023 (LOVD database)	Brazil	MINAS_06	F	NM_000059.3(BRC A2):c.5617_5621d	NM_007194.3(CH EK2):c.470T>C		Breast cancer 49y*
329	Torrezan et al. 2023 (LOVD database)	Brazil	MINAS_08	F	NM_000051.4(ATM):c.6529C>T	NM_000059.3(BR CA2):c.156_157in		Breast cancer 41y*
330	Torrezan et al. 2023 (LOVD database)	Brazil	MINAS_10	F	NM_007194.3(CH EK2):c.2711G>A	NM_024675.3(PA LB2):c.319+2T>A		Breast cancer 43y*
331	Torrezan et al. 2023 (LOVD database)	Brazil	MINAS_11	F	NM_000059.3(BRC A2):c.9117G>A	NM_007194.3(CH EK2):c.847-1G>T		Bilateral breast cancer 43y*
332	Torrezan et al. 2023 (LOVD database)	Brazil	MINAS_12	F	NM_000059.3(BRC A2):c.5681dup	NM_000546.6(TP 53):c.1010G>A		Breast cancer 36y*
333	Torrezan et al. 2023 (LOVD database)	Brazil	MINAS_13	F	NM_007294.4(BRC A1):c.8351G>A	NM_000059.3(BR CA2):c.5266dup		Breast cancer 35y*
334	Torrezan et al. 2023 (LOVD database)	Brazil	MINAS_14	F	NM_032043.2(BRI P1):c.1201_1204d	NM_001128425.1 (MUTYH):c.1187G		Beast cancer 38y†
335	Torrezan et al. 2023 (LOVD database)	Brazil	MINAS_15	F	NM_007194.3(CH EK2):c.1427C>T	NM_024675.3(PA LB2):c.43G>T		Breast cancer 33y*
336	Torrezan et al. 2023 (LOVD database)	Brazil	MINAS_17	F	NM_001128425.1(MUTYH):c.617A>G	NM_004168.3(SD HA):c.554dup		GIST 31y‡
337	Torrezan et al. 2023 (LOVD database)	Brazil	MINAS_18	F	NM_000059.3(BRC A2):c.6381_6382in	NM_002875.4(RA D51):c.449G>A		Breast cancer 32y*
338	Torrezan et al. 2023 (LOVD database)	Brazil	MINAS_19	M	NM_000059.3(BRC A2):c.7976G>A	NM_007194.3(CH EK2):c.793-1G>A		Prostate adenocarcinoma 61y†

339	Torrezan et al. 2023 (LOVD database)	Brazil	MINAS_21	F	NM_007294.4(BRCA1):c.4712del	NM_001128425.1(MUTYH):c.536A>		Adenocarcinoma of left ovary and carcinosarcoma of the right ovary 51y†
340	Torrezan et al. 2023 (LOVD database)	Brazil	MINAS_22	F	NM_000059.3(BRCA2):c.2808_2811d	NM_006502.3(POLH):c.1222_1225		Melanoma 31y†<br>Desmoid tumour 31yΔ
341	Torrezan et al. 2023 (LOVD database)	Brazil	MINAS_23	F	NM_000051.4(ATM):c.(?_3994-2)_(4236+7_?)del	NM_007294.4(BRCA1):c.1687C>T		Breast cancer 39y*
342	Torrezan et al. 2023 (LOVD database)	Brazil	MINAS_24	F	NM_001128425.1(MUTYH):c.736G>T	NM_006502.3(POLH):c.490G>T		Breast cancer 44yΔ
343	Torrezan et al. 2023 (LOVD database)	Brazil	MINAS_25	M	NM_000059.3(BRCA2):c.9382C>T	NM_001040108.1(MLH3):c.82G>T		Malignant meningeal melanoma of the equine tail 56y†
344	Torrezan et al. 2023 (LOVD database)	Brazil	MINAS_26	F	NM_000465.3(BARD1):c.1970dup	NM_000249.3(MLH1):c.1667+1G>A		Colon cancer (65y)‡<br>Breast cancer (69 yo)†
345	Torrezan et al. 2023 (LOVD database)	Brazil	MINAS_27	F	NM_007194.3(CHEK2):c.470T>C	NM_001113378.1(FANCI):c.3853C>		Breast cancer 59y†
346	Torrezan et al. 2023 (LOVD database)	Brazil	MINAS_28	M	NM_000179.3(MSH6):c.2059dup	NM_000546.6(TP53):c.1010G>A		Glioma 46y‡
347	Torrezan et al. 2023 (LOVD database)	Brazil	MINAS_29	F	NM_000135.2(FANCA):c.3788_3790d	NM_000546.6(TP53):c.1010G>A		Breast cancer 48y‡
348	Torrezan et al. 2023 (LOVD database)	Brazil	MINAS_30	F	NM_000059.3(BRCA2):c.2808_2811d	NM_007194.3(CHEK2):c.1111_1127		Breast cancer 37y*
349	Torrezan et al. 2023 (LOVD database)	Brazil	MINAS_31	F	NM_007294.4(BRCA1):c.5074+2T>C	NM_000535.4(PMS2):c.1119_1122d		Breast cancer 29y*
350	Torrezan et al. 2023 (LOVD database)	Brazil	MINAS_32	F	NM_007294.4(BRCA1):c.1687C>T	NM_001128425.1(MUTYH):c.536A>		Breast cancer 40y†
351	Torrezan et al. 2023 (LOVD database)	Brazil	MINAS_34	F	NM_001128425.1(MUTYH):c.1187G>	NM_003401.5(XRCC4):c.25del		Breast cancer 40y†
352	Torrezan et al. 2023 (LOVD database)	Brazil	MINAS_35	F	NM_001128425.1(MUTYH):c.1187G>A	NM_003401.5(XRCC4):c.25del		Kidney cancer 44yΔ<br>Colon adenocarcinoma 44y†<br>Thyroid cancer 44yΔ

353	Megid et al. 2022	Paper from Brazil	1:F02114	F	NM_007294.4(BRCA1):c.5266dupC	NM_024675.3(PALB2):c.3271C>T	NM_000546.6(TP53):c	Breast cancer 32y*
354	Megid et al. 2022	Paper from Brazil	2:F02114	F	NM_007294.4(BRCA1):c.5266dupC	NM_024675.3(PALB2):c.3271C>T	NM_000546.6(TP53):c	Breast cancer 40y*
355	Megid et al. 2022	Paper from Brazil	3:F00620	F	NM_007294.4(BRCA1):c.4165_4166d	NM_024675.3(PALB2):c.1240C>T	NM_000546.6(TP53):c	Breast cancer 32y; 37y*
356	Megid et al. 2022	Paper from Brazil	4:F00006)	F	NM_007294.4(BRCA1):c.5266dupC	NM_000546.6(TP53):c.1010G>A		Breast cancer 36y*<br>Pancreas cancer 40y*<br>Lung cancer 53y‡
357	Megid et al. 2022	Paper from Brazil	5:F00952	F	NM_000051.4(ATM):c.5266dupC	NM_007294.4(BRCA1):c.6729_673		TNBC 33y*
358	Megid et al. 2022	Paper from Brazil	6:F01844	F	NM_007294.4(BRCA1):c.1687C>T	NM_002485.4(NBN):c.1142delC		Breast cancer 38y; 58y*
359	Megid et al. 2022	Paper from Brazil	7:FY0240	F	NM_000059.3(BRCA2):c.6024dup	NM_007194.3(CHEK2):c.478A>G		Breast cancer 40y; 57y*
360	Megid et al. 2022	Paper from Brazil	8:F02417	F	NM_000059.3(BRCA2):c.2516dupA	NM_000535.4(PMS2):c.2182_2184d		Breast cancer 49y†
361	Megid et al. 2022	Paper from Brazil	9:FY0073	F	NM_000051.4(ATM):c.8960del	NM_000059.3(BRCA2):c.901+1G>A		Breast cancer 32y*
362	Megid et al. 2022	Paper from Brazil	10:FY0073	F	NM_000051.4(ATM):c.8960del	NM_000059.3(BRCA2):c.901+1G>A		Breast cancer 42y; 52y*
363	Megid et al. 2022	Paper from Brazil	11:FY0751	F	NM_024675.3(PALB2):c.2711G>A	NM_000546.6(TP53):c.1010G>A		Breast cancer 47y*
364	Megid et al. 2022	Paper from Brazil	12:F01266	F	NM_000051.4(ATM):c.4906C>T	NM_000546.6(TP53):c.1010G>A		Breast cancer 42y*
365	Megid et al. 2022	Paper from Brazil	13:F01776	F	NM_007194.3(CHEK2):c.319+2T>A	NM_024675.3(PALB2):c.2711G>A		Breast cancer 42y*
366	Megid et al. 2022	Paper from Brazil	14:F02082	F	NM_000051.4(ATM):c.2140del	NM_024675.3(PALB2):c.3350+5G>		Breast cancer 42y*
367	Tung et al. 2022	Paper from USA	1	F	NM_003002.3(SDHD):c.1054C>T	NM_000551.3(VHL):c.500G>A		Well-differentiated neuroendocrine tumor at pancreatic tail 23y*

368	Vibert et al. 2023	Paper from France		M	NM_000249.3(MLH1):c.199G>T	NM_002354.2(EPCAM):c.(?_?)del		Colon cancer 21y*
369	Vibert et al. 2023	Paper from France		M	NM_000535.4(PMS2):gene	NM_001128425.1(MUTYH):c.1145G		Colon cancer 28y*
370	Vibert et al. 2023	Paper from France		M	NM_000535.4(PMS2):(?_?)del (exons 9_10del)	NM_001128425.1(MUTYH):c.1145G>A		Colon cancer 26y*
371	Vibert et al. 2023	Paper from France		M	NM_000249.3(MLH1):c.678-2A>T	NM_000051.4(ATM):c.6100C>T		Colon cancer 28y
372	D'Elia G et al. 2023	Paper from Italy		F	NM_032043.2(BRIP1):c.55dup	NM_001128425.1(MUTYH):c.1187G		Breast cancer 57y†
373	This article	Chinese	11	M	NM_000314.4(PTEN):c.802-2A>G	NM_000553.4(WRN):c.502_503delAA		Multiple adenomatous & hyperplastic polyps 43y†;<br>Tongue papilloma 53y†;<br>Thyroid nodule 41y†;<br>Back lipoma 41y†;<br>Macocephaly†; keratosis†; trichilemmomas†; short stature†, flat nasal bridge†, severe teeth cavies (he lost all his teeth in his 30-40s)†; small chin/jaw (Micrognathia)†
374	This article	Chinese	11	F	NM_000314.4(PTEN):c.802-2A>G	NM_000553.4(WRN):c.502_503delA		Synchronous TNBC and HER2+ breast cancer primaries†
375	This article	Emirati (from UAE)	12	F	NM_024675.3(PALB2):c.2257C>T	NM_000535.4(PMS2):c.730C>T		Breast cancer 68y R IDC triple negative†;<br>Benign ovarian serous cyst adenoma 68yΔ;<br>Chronic salpingitis 68yΔ
376	This article	Indian	13	F	NM_000059.3(BRCA2):c.5583dupA	NM_002439.3(MSH3):c.802C>T		Breast cancer 39y L IDC triple negative†;
377	This article	Chinese	14	F	NM_007294.4(BRCA1):(?_?)del (exons 1_15del)	NM_005236.2(ERCC4):c.2169C>A		Breast cancer 35y R IDC†;<br>Bilateral breast cancer 55y L IDC triple negative†

378	This article	Chinese	15	F	NM_007294.4(BRCA1):c.3214delC	NM_002485.4(NBN):c.1882_1885d		Ovarian cancer 63y HGS†
379	This article	Malay	16	F	NM_000051.4(ATM):c.2377-2A>G	NM_000179.3(MSH6):c.2603del		Endometrial cancer 60y‡ endometroid IHC normal
380	This article	Emirati (from UAE)	17	F	NM_000038.6(APC):c.3920T>A	NM_000059.3(BRCA2):c.5645C>A		Breast cancer 38y‡ L IDC ER/PR+ Her2- FISH-
381	This article	Chinese	18	F	NM_000051.4(ATM):c.1702A>T	NM_000059.3(BRCA2):c.1763_176		Breast cancer 45y* R IDC ER/PR+ Her2-
382	This article	Chinese	19	M	NM_000249.3(MLH1):c.790+1G>A	NM_002485.4(NBN):c.1496C>A		Colorectal cancer 47† transverse colon adenocarcinoma
383	This article	Chinese	20	F	NM_007294.4(BRCA1):c.3661G>T	NM_003000.2(SDHB):c.(?_?)del (exon 1del)		Ovarian carcinoma 73y<br>Breast cancer 60y and 72y<br>Papillary urothelial carcinoma, age unknown
384	This article	Chinese	21	F	NM_032043.2(BRIP1):c.1343G>A	NM_002878.3(RAD51D):c.270_271		Triple negative breast ca 45y
385	This article	Chinese	22	F	NM_000465.3(BARD1):c.69_70delins	NM_058216.2(RAD51C):c.394dup		Breast cancer 49y† R IDC ER/PR- Her2equviocal
386	This article	Chinese	23	F	NM_007294.4(BRCA1):(?_?)del (exons	NM_000249.3(MLH1):c.2081-1G>A		Breast cancer 36y†
387	This article	Chinese	24	F	NM_000059.3(BRCA2):c.5164_5165d	NM_001128425.1 (MUTYH):c.467G>		Breast IDC ER/PR+ Her2-†
388	This article	Chinese	25	F	NM_000135.2(FANCA):c.1777-1G>C	NM_006231.3(POLE):c.1360-		Papillary thyroid ca 52yΔ<br>Triple negative breast ca 39†
389	This article	Chinese	26	F	NM_007294.4(BRCA1):c.1098T>C	NM_002354.2(EPCAM):c.(?_?)del		Papillary ovarian ca 52y*<br>Breast IDC ER/PR+ Her2- 52y‡
390	This article	Chinese	27	F	NM_000179.3(MSH6):c.1190_1191d	NM_000135.2(FANCA):c.2639G>A		Fallopian tube cancer dx 39y†
391	This article	Chinese	28	M	NM_000059.3(BRCA2):c.3716_3717d	NM_000267.3(NF1):c.5944-5A>G		Clinical dx NF1‡

392	This article	Chinese	29	F	NM_000465.3(BARD1):c.623dup	NM_032043.2(BRIP1):c.(?_?)del (exons 5_6del)		Pancreas cancer 58yΔ<br>Benign breast cysts
393	This article	Chinese	30	F	NM_007294.4(BRCA1):(?_?)del (exon	NM_000249.3(MLH1):c.(?_?)del		Endometrial cancer 31y‡<br>Ovarian cancer 31y†
394	This article	Chinese	31	F	NM_000059.3(BRCA2):c.5073dup	NM_000135.2(FANCA):c.3918dup		Triple negative breast ca 44y†
395	Schamschula et al. 2022	Paper from Germany	1	M	NM_000535.4(PMS2):c.2007-786_2174+493del1447	NM_002691.3(POLD1):c.946G>A		Rectal carcinoma 17y* - MSI-H<br>Multiple adenomas 17y*<br>Colon ascendens right flexure and cecum bifocal carcinoma 27y* - PMS2 loss<br>Distal right ureter urothelial carcinoma 34y†<br>Nephrogenic adenoma of the urinary bladderΔ
396	Schamschula et al. 2022	Paper from Germany	1	F	NM_000535.4(PMS2):c.2007-786_2174+493del1447	NM_002691.3(POLD1):c.946G>A		Synchronous cecal and colon ascendens carcinoma 19y* - PMS2 loss
397	Mur et al. 2020	Spanish	1	F	NM_000051.4(ATM):c.7220C>A	NM_002691.3(POLD1):c.1573C>T		Breast CA 35y*<br>Breast CA 49y*<br>Breast CA 53y*<br>CRC 69y*
395	Schamschula et al. 2022	Paper from Germany	1	M	NM_000535.4(PMS2):c.2007-786_2174+493del1447	NM_002691.3(POLD1):c.946G>A		Rectal carcinoma 17y* - MSI-H<br>Multiple adenomas 17y*<br>Colon ascendens right flexure and cecum bifocal carcinoma 27y* - PMS2 loss<br>Distal right ureter urothelial carcinoma 34y†<br>Nephrogenic adenoma of the urinary bladderΔ
396	Schamschula et al. 2022	Paper from Germany	1	F	NM_000535.4(PMS2):c.2007-786_2174+493del1447	NM_002691.3(POLD1):c.946G>A		Synchronous cecal and colon ascendens carcinoma 19y* - PMS2 loss

397	Mur et al. 2020	Spanish	1	F	NM_000051.4(ATM):c.7220C>A	NM_002691.3(POLD1):c.1573C>T		Breast CA 35y*<br>Breast CA 49y*<br>Breast CA 53y*<br>CRC 69y*
398	Madar et al. 2023	Hungarian	1	F	NM_007294.4(BRCA1):c.5095C>T	NM_000059.3(BRCA2):c.658_659d		TNBC 36y* IDC pT2pN0, histological grade 3
399	Harada et al. 2023	Japanese	1	F	NM_000059.3(BRCA2):c.8016delA	NM_000251.2(MSH2):c.1168_1170		Ovarian cancer 28y*
400	Harada et al. 2024	Japanese	1	M	NM_000059.3(BRCA2):c.8016delA	NM_000251.2(MSH2):c.1168_1170 dup		Rectal cancer 43yΔ<br>Colon cancer 51y‡<br>Bladder cancer 54y‡<br>Renal pelvis cancer 64y‡
401	Harada et al. 2025	Japanese	1	F	NM_000059.3(BRCA2):c.8016delA	NM_000251.2(MSH2):c.1168_1170 dup		Renal cancer 55yΔ<br>Colon cancer 70y‡<br>Rectal carcinoid 70yΔ<br>Gastric cancer 72y‡
402	Sanchez-Castro et al. 2022	Peruvian	1	F	NM_000051.4(ATM):c.8156G>A	NM_005732.3(RAD50):c.3715C>T		Breast Ca 50y*<br>Endometrial hyperplasia 53yΔ
403	Huang et al.	Chinese	1	M	NM_000038.6(APC):(?_?)del (exon	NM_007294.4(BRCA1):c.5469delA		Colorectal CA 20y*
404	Raygada et al. 2021	Paper from USA	1	F	NM_000251.2(MSH2):c.211+1G>T	NM_020975.4(RET):c.2410G>A		ACC 40yΔ
405	Pinto et al. 2016	Portuguese	1	M	NM_000251.2(MSH2):c.2785C>T	NM_000179.3(MSH6):c.1030C>T		Colon adenoma 26y*
406	Pinto et al. 2016	Portuguese	2	F	NM_000251.2(MSH2):c.2785C>T	NM_000179.3(MSH6):c.1030C>T		Colorectal CA 38y*
407	Pinto et al. 2016	Portuguese	3	F	NM_000251.2(MSH2):c.2785C>T	NM_000179.3(MSH6):c.1030C>T		Colon adenoma 31y*
408	Pinto et al. 2016	Portuguese	4	M	NM_000251.2(MSH2):c.2785C>T	NM_000179.3(MSH6):c.1030C>T		Colorectal CA 48y*

409	Pinto et al. 2016	Portuguese	5	F	NM_000251.2(MSH2):c.2785C>T	NM_000179.3(MSH6):c.1030C>T		Colorectal CA 40y*
410	Pinto et al. 2016	Portuguese	6	F	NM_000251.2(MSH2):c.2785C>T	NM_000179.3(MSH6):c.1030C>T		Colorectal CA 47y*
411	Pinto et al. 2016	Portuguese	7	F	NM_000251.2(MSH2):c.2785C>T	NM_000179.3(MSH6):c.1030C>T		Ovarian CA 46y*
412	Pinto et al. 2016	Portuguese	8	F	NM_000251.2(MSH2):c.2785C>T	NM_000179.3(MSH6):c.1030C>T		Endometrial CA 47y*
413	Goehringer et al. 2016	Paper from Germany	1	M	NM_000038.6(APC):c.3103dupC	NM_000059.3(BRCA2):c.516_516?1delGGinsT		Colon adenomas 35y†<br>Right thoracic wall, right groin, upper jejunum desmoid tumors†<br>Intraductal papillary mucinous neoplasm (IPMN)‡
