# Supplementary material for: Multi-locus inherited neoplasia alleles syndromes in cancer: implications for clinical practice
Source: Eur J Hum Genet. 2025 Jan 23;33(3):289–96. doi: 10.1038/s41431-025-01785-1 (PMC11894078; doi:10.1038/s41431-025-01785-1)
Supplement: Supplementary file 5 — Supplementary Table 5: Omitted Cases [file 41431_2025_1785_MOESM5_ESM.pdf]

Supplementary Table 5: Omitted Cases

Reference	Ethnicity	Family ID in report	Sex	PV 1	PV 1 details	PV 2	PV 2 details	PV 3	PV 3 details	Clinical Hx	Reason for removal
Friedman et al. 1998	Ashkenazi J	3	F	BRCA1	c.66_67delAG	BRCA2	c.5946delTp.Ser1982Argfs*22			No features 50y	No cancer
Loader et al. 1998	German	1	M	BRCA1	c.5080G>Tp.G	BRCA2	c.6405_6409delCTTAAp.Asn2135fs			No features 36y	No cancer
Loader et al. 1998	German	1	F	BRCA1	c.5080G>Tp.G	BRCA2	c.6405_6409delCTTAAp.Asn2135fs			No features 34y	No cancer
Loader et al. 1998	German	1	M	BRCA1	c.5080G>Tp.G	BRCA2	c.6405_6409delCTTAAp.Asn2135fs			No features 30y	No cancer
Moslehi et al. 2000	Ashkenazi J	1	F	BRCA1	c.3769_3770d	BRCA2	c.5946delT p.Ser1982Argfs*22			No features 36y	No cancer
de la Hoya et al., 2002	Spanish	1	F	BRCA1	c.5123C>Ap.A	BRCA2	c.6275_6276delTTp.Leu2092Profs*			No features 77y	No cancer
de la Hoya et al., 2002	Spanish	1	F	BRCA1	c.5123C>Ap.A	BRCA2	c.6275_6276delTTp.Leu2092Profs*			No features 47y	No cancer
de la Hoya et al., 2002	Spanish	1	F	BRCA1	c.5123C>Ap.A	BRCA2	c.6275_6276delTTp.Leu2092Profs*			No features 41y	No cancer
de la Hoya et al., 2002	Spanish	1	F	BRCA1	c.5123C>Ap.A	BRCA2	c.6275_6276delTTp.Leu2092Profs*			No features 40y	No cancer
Leegte et al. 2005<br>Frank et al. 2002	Unknown (	Unknown	F	BRCA1	c.66_67delAG	BRCA2	c.5946delTp.Ser1982Argfs*22			No features 66y	No cancer
Leegte et al. 2005<br>Frank et al. 2002	Unknown (	Unknown	F	BRCA1	c.66_67delAG	BRCA2	c.5946delTp.Ser1982Argfs*22			No features 61y	No cancer
Leegte et al. 2005	Ashkenazi J	3	F	BRCA1	c.66_67delAG	BRCA2	c.5946delTp.Ser1982Argfs*22			No features 40y	No cancer
Smith et al. 2008	Unknown (	1	F	BRCA1	c.3331_3334d	BRCA2	c.631+2T>G			No features 65y	No cancer
Steffensen et al. 2010	Danish	1	F	BRCA1	c.5096G>Ap.A	BRCA2	c.631+4A>G			No features	No cancer
Steffensen et al. 2010	Danish	1	M	BRCA1	c.5096G>Ap.A	BRCA2	c.631+4A>G			No features	No cancer
Mastroianno et al. 2011	Italian	1	M	RET	c.1997A>Tp.Ly	MEN1	c.669+1G>T			No features 6y	No cancer
Heidemann et al. 2012	German	2	M	BRCA1	c.68_69delAG	BRCA2	c.5718_5719delp.Leu1908Argfs*2			No features 53y	No cancer
Heidemann et al. 2012	German	5	M	BRCA1	c.5277+1delG	BRCA2	c.658_659delGTp.Val220Ilefs*4			No features (no ag	No cancer
Loubser et al. 2012	Afrikaner	1	M	BRCA1	c.2641G>Tp.G	BRCA2	c.7934delGp.Arg2645Asnfs*3			No features 49y	No cancer
Ahlborn et al. 2014	Pakistani	1	F	BRCA2	c.9648G>Ap.L	RAD51C	c.773G>Ap.Arg258His			No features 38yo	No cancer
Palmirotta et al. 2018	Italian	1	F	BRCA1	c.1687C>Tp.G	BRCA2	c.9976A>Tp.Lys3326*			No features 36y	No cancer
Vietri et al. 2020	Italian	2	F	BRCA1	c.3752_3755G	BRCA2	c.425+2T>C(IVS4+2T>C)			No features 70y	No cancer
Laish et al. 2021	Ashkenazi J	2	M	BRCA2	c.6174delp.Ph	MSH6	c.3984_3987dupGTCAp.Leu1330fs			No features 37y	No cancer
Laish et al. 2021	Ashkenazi J	3	F	BRCA2	c.6174delp.Ph	MSH6	c.3956_3957dupp.Ala1320fs			No features 31y	No cancer
Laish et al. 2021	Ashkenazi J	4	M	BRCA2	c.6174delp.Ph	MSH2	c.1906G>Cp.Ala636Pro			No features 50y	No cancer
Ferrer-Avargues et al. 2021	Unknown (	1	M	BRCA2	c.3492dupTp.	MLH1	c. 1717_1718delGTp.Val573fs*11			No features	No cancer
Ferrer-Avargues et al. 2021	Unknown (	2	M	BRCA1	c.5152+5G>A	MLH1	c.701delA p.Glu234Glyfs*5			No features	No cancer
Ferrer-Avargues et al. 2021	Unknown (	2	M	BRCA1	c.5152+5G>A	MLH1	c.701delA p.Glu234Glyfs*5			No features	No cancer

Koren-Michowitz et al. 200	Ashkenazi	1	F	BRCA1	c.5382insC p.(	BRCA2	c.6174delT	FANCC	c.456+4A>T	No features 33y	No cancer
Vietri et al. 2015	Italian	1	M	BRCA1	c.547+2T>Ap.(	BRCA2	c.2830A>T	BRCA2	c.426-57A>	No features 71y	No cancer
Balta et al. 2019	Paper from	1	M	FANCA	c.1374delC	ATM	c.8977C>T	FANCA	c.1361-137	No features 36y	No cancer
Balta et al. 2019	Paper from	1	F	FANCA	c.1374delC	ATM	c.8977C>T	FANCA	c.1361-137	No features 36y	No cancer
Frank-Raue et al. 2005	Paper from	1	F	MEN1	IVS5 + 1G>A	RET	Y791F			No features	No cancer
This article	Caucasian	27	M	BRCA1	c.1417C>T p.G	BLM	c.1479_1480delp.Thr494Profs*9			No features 33y	No cancer
Rebbeck et al. 2016	Jewish	-	F	BRCA1	c.68_69delAG	BRCA2	c.5946del p.Ser1982fs			No cancer	No cancer
Rebbeck et al. 2016	Jewish	-	F	BRCA1	c.68_69delAG	BRCA2	c.5946del p.Ser1982fs			No cancer	No cancer
Rebbeck et al. 2016	Jewish	-	F	BRCA1	c.68_69delAG	BRCA2	c.5946del p.Ser1982fs			No cancer	No cancer
Rebbeck et al. 2016	Jewish	-	F	BRCA1	c.68_69delAG	BRCA2	c.5946del p.Ser1982fs			No cancer	No cancer
Rebbeck et al. 2016	Jewish	-	F	BRCA1	c.68_69delAG	BRCA2	c.5946del p.Ser1982fs			No cancer	No cancer
Rebbeck et al. 2016	Jewish	-	F	BRCA1	c.68_69delAG	BRCA2	c.5946del p.Ser1982fs			No cancer	No cancer
Rebbeck et al. 2016	Jewish	-	F	BRCA1	c.68_69delAG	BRCA2	c.5946del p.Ser1982fs			No cancer	No cancer
Rebbeck et al. 2016	Jewish	-	F	BRCA1	c.68_69delAG	BRCA2	c.5946del p.Ser1982fs			No cancer	No cancer
Rebbeck et al. 2016	Jewish	-	F	BRCA1	c.68_69delAG	BRCA2	c.5946del p.Ser1982fs			No cancer	No cancer
Rebbeck et al. 2016	Jewish	-	F	BRCA1	c.68_69delAG	BRCA2	c.5946del p.Ser1982fs			No cancer	No cancer
Rebbeck et al. 2016	Jewish	-	F	BRCA1	c.68_69delAG	BRCA2	c.5946del p.Ser1982fs			No cancer	No cancer
Rebbeck et al. 2016	Jewish	-	F	BRCA1	c.68_69delAG	BRCA2	c.5946del p.Ser1982fs			No cancer	No cancer
Rebbeck et al. 2016	Jewish	-	F	BRCA1	c.68_69delAG	BRCA2	c.5946del p.Ser1982fs			No cancer	No cancer
Rebbeck et al. 2016	Jewish	-	F	BRCA1	c.68_69delAG	BRCA2	c.5946del p.Ser1982fs			No cancer	No cancer
Rebbeck et al. 2016	Austrian		F	BRCA1	c.181T>Gp.Glu	BRCA2	c.1318_1319dupCTp.Thr441fs			No cancer	No cancer
Rebbeck et al. 2016	Austrian		F	BRCA1	c.181T>Gp.Glu	BRCA2	c.1318_1319dupCTp.Thr441fs			No cancer	No cancer
Rebbeck et al. 2016	Caucasian (UK)		F	BRCA1	c.246delTp.Va	BRCA2	c.517-2A > G			No cancer	No cancer
Rebbeck et al. 2016	Swedish		F	BRCA1	c.3048_3052d	BRCA2	c.2830A>Tp.Lys944Ter			No cancer	No cancer
Rebbeck et al. 2016	Caucasian (Australia)		F	BRCA1	c.3155delAp.A	BRCA2	c.3160_ 3163delGATAp.Asp1054fs			No cancer	No cancer
Rebbeck et al. 2016	Caucasian (UK)		F	BRCA1	c.3400G>T	BRCA2	c.2808_2811delACAA			No cancer	No cancer
Rebbeck et al. 2016	Caucasian (Australia)		F	BRCA1	c.4035delA	BRCA2	c.658_659delGT			No cancer	No cancer
Rebbeck et al. 2016	Caucasian (UK)		F	BRCA1	c.4186-?_435	BRCA2	c.2636_2637delCT			No cancer	No cancer
Rebbeck et al. 2016	Jewish		F	BRCA1	c.5266dupC	BRCA2	c.5946delT			No cancer	No cancer
Stolavora et al. 2020	Czech		F	OCA2	c.1211C>T (p.	KAT6A	c.1138G>T (p.E380*)			No cancer 29y	No cancer
Sukumar et al. 2021	Caucasian	2	F	BRCA1	c.181T>Gp.Cy	CHEK2	c.1100delC			No cancer 29y	No cancer
Tsongalis et al. 1998	Unknown (	1	F	BRCA1	c.68_69delAG	BRCA2	c.5946delTp.Ser1982Argfs*22			Unknown	Insufficient c
Ouyang et al. 2019	Chinese	9	F	BRCA1	p.V1120Dfs*1	BRCA2	p.D687*fs*1			Unknown primary	Insufficient c

Tesoriero et al. 1999	Unknown (	1	F	BRCA1	c.3770_3771d	BRCA2	c.5946delTp.Ser1982Argfs*22		Breast cancer* LOH	Insufficient c
Whitworth et al. 2016	Unknown (	3	F	FLCN	c.1285delCp.H	MSH2	c.892C>Tp.Gln298*		Renal cell carcinom	Insufficient c
Whitworth et al. 2016	Unknown (	3	M	FLCN	c.1285delCp.H	MSH2	c.892C>Tp.Gln298*		Facial fibrofolliculo	Insufficient c
Li et al. 2019	Chinese	1	F	BRCA1	c.1214C>Gp.S	BRCA2	c.3883C>Tp.Gln1295*		Ovarian cancer HG	Insufficient c
Chatzikyriakou et al. 2021	Unknown (	1	M	NF1	c.5107delA p.S	SDHD	c.3G>Ap.Met1Ile		Multiple head and neck paragangliomas (HNPGL)‡	Insufficient c
Shani et al. 2021	Ashkenazi J	1	F	BRCA1	c.5266dupp.G	TP53	c.733C>Ap.Gly245Ser		primary infertility (	No cancer
Rebbeck et al. 2016	Jewish	-	F	BRCA1	c.-19-?_80 + ?	BRCA2	c.8633-?_8754 + ?amp*		Breast cancer*	Insufficient c
Rebbeck et al. 2016	Jewish		F	BRCA1	c.68_69delAG	BRCA2	c.5946del p.Ser1982fs		Breast cancer*	Insufficient c
Rebbeck et al. 2016	Jewish	-	F	BRCA1	c.68_69delAG	BRCA2	c.5946del p.Ser1982fs		Breast cancer*	Insufficient c
Rebbeck et al. 2016	Jewish	-	F	BRCA1	c.68_69delAG	BRCA2	c.5946del p.Ser1982fs		Breast cancer*	Insufficient c
Rebbeck et al. 2016	Jewish	-	F	BRCA1	c.68_69delAG	BRCA2	c.5946del p.Ser1982fs		Breast cancer*	Insufficient c
Rebbeck et al. 2016	Jewish	-	F	BRCA1	c.68_69delAG	BRCA2	c.5946del p.Ser1982fs		Breast cancer*	Insufficient c
Rebbeck et al. 2016	Jewish	-	F	BRCA1	c.68_69delAG	BRCA2	c.5946del p.Ser1982fs		Breast cancer*	Insufficient c
Rebbeck et al. 2016	Jewish	-	F	BRCA1	c.68_69delAG	BRCA2	c.5946del p.Ser1982fs		Breast cancer*	Insufficient c
Rebbeck et al. 2016	Jewish	-	F	BRCA1	c.68_69delAG	BRCA2	c.5946del p.Ser1982fs		Breast cancer*	Insufficient c
Rebbeck et al. 2016	Jewish	-	F	BRCA1	c.68_69delAG	BRCA2	c.5946del p.Ser1982fs		Breast cancer*	Insufficient c
Rebbeck et al. 2016	Jewish	-	F	BRCA1	c.68_69delAG	BRCA2	c.5946del p.Ser1982fs		Breast cancer*	Insufficient c
Rebbeck et al. 2016	Jewish	-	F	BRCA1	c.68_69delAG	BRCA2	c.5946del p.Ser1982fs		Breast cancer*	Insufficient c
Rebbeck et al. 2016	Jewish	-	F	BRCA1	c.68_69delAG	BRCA2	c.5946del p.Ser1982fs		Breast cancer*	Insufficient c
Rebbeck et al. 2016	Jewish	-	F	BRCA1	c.68_69delAG	BRCA2	c.5946del p.Ser1982fs		Breast cancer*	Insufficient c
Rebbeck et al. 2016	Jewish	-	F	BRCA1	c.68_69delAG	BRCA2	c.5946del p.Ser1982fs		Ovarian cancer*	Insufficient c
Rebbeck et al. 2016	Jewish	-	F	BRCA1	c.68_69delAG	BRCA2	c.5946del p.Ser1982fs		Breast cancer*Ova	Insufficient c
Rebbeck et al. 2016	Jewish	-	F	BRCA1	c.68_69delAG	BRCA2	c.5946del p.Ser1982fs		Breast cancer*Ova	Insufficient c
Rebbeck et al. 2016	Jewish	-	F	BRCA1	c.68_69delAG	BRCA2	c.5946del p.Ser1982fs		Breast cancer*Ova	Insufficient c
Rebbeck et al. 2016	Caucasian (USA)		F	BRCA1	c.1793T>?p.Le	BRCA2	c.8537_8538delAGp.Glu2846fs		Breast cancer*Ova	Insufficient c
Rebbeck et al. 2016	Austrian		F	BRCA1	c.181T>Gp.Glu	BRCA2	c.1318_1319dupCTp.Thr441fs		Breast cancer*	Insufficient c
Rebbeck et al. 2016	Caucasian (UK)		F	BRCA1	c.211A>G	BRCA2	c.4380_4381delTTp.Ser1461fs		Breast cancer*	Insufficient c
Rebbeck et al. 2016	Spanish		F	BRCA1	c.212 + 1G>A	BRCA2	c.739_740delATp.Ile247fs		Breast cancer*	Insufficient c
Rebbeck et al. 2016	Italian		F	BRCA1	c.213-12A>G	BRCA2	c.7180A>Tp.Arg2394Ter		Breast cancer*	Insufficient c
Rebbeck et al. 2016	Caucasian (Canada)		F	BRCA1	c.2389G>Tp.G	BRCA2	c.3068dupAp.Asn1023fs		Breast cancer*	Insufficient c
Rebbeck et al. 2016	Italian		F	BRCA1	c.2405_2406d	BRCA2	c.4284dupTp.Gln1429fs		Breast cancer*Ova	Insufficient c
Rebbeck et al. 2016	Caucasian (UK)		F	BRCA1	c.246delTp.Va	BRCA2	c.517-2A > G		Breast cancer*	Insufficient c
Rebbeck et al. 2016	Caucasian (USA)		F	BRCA1	c.301+1G>A	BRCA2	c.5682C>Gp.Tyr1894Ter		Breast cancer*	Insufficient c

Rebbeck et al. 2016	Swedish		F	BRCA1	c.3048_3052d	BRCA2	c.2830A>Tp.Lys944Ter		Breast cancer*	Insufficient c
Rebbeck et al. 2016	Caucasian (Australia)		F	BRCA1	c.3155delAp.A	BRCA2	c.3160_ 3163delGATAp.Asp1054fs		Breast cancer*	Insufficient c
Rebbeck et al. 2016	German		F	BRCA1	c.3196G>Tp.G	BRCA2	c.658_659delGTp.Val220fs		Breast cancer*	Insufficient c
Rebbeck et al. 2016	Caucasian (UK)		F	BRCA1	c.3228_3229d	BRCA2	c.3689delC		Breast cancer*Ova	Insufficient c
Rebbeck et al. 2016	Italian		F	BRCA1	c.3228_3229d	BRCA2	c.9253dupA		Breast cancer*	Insufficient c
Rebbeck et al. 2016	Caucasian (UK)		F	BRCA1	c.3400G>T	BRCA2	c.2808_2811delACAA		Breast cancer*	Insufficient c
Rebbeck et al. 2016	Italian		F	BRCA1	c.3477_3480 d	BRCA2	c.9401delG		Ovarian cancer*	Insufficient c
Rebbeck et al. 2016	Korean		F	BRCA1	c.3627dupA	BRCA2	c.6724_6725delGA		Breast cancer*	Insufficient c
Rebbeck et al. 2016	Caucasian (Australia)		F	BRCA1	c.3700_3704d	BRCA2	c.681 + 1G > A		Breast cancer*	Insufficient c
Rebbeck et al. 2016	German		F	BRCA1	c.3700_3704d	BRCA2	c.1815dupA		Breast cancer*	Insufficient c
Rebbeck et al. 2016	Caucasian (USA)		F	BRCA1	c.3756_3759d	BRCA2	c.7757G>A		Breast cancer*	Insufficient c
Rebbeck et al. 2016	Hispanic		F	BRCA1	c.3759_3760d	BRCA2	c.9699_9702 delTATG		Breast cancer*Ova	Insufficient c
Rebbeck et al. 2016	Jewish		F	BRCA1	c.3770_3771d	BRCA2	c.5946delT		Breast cancer*	Insufficient c
Rebbeck et al. 2016	Jewish		F	BRCA1	c.3770_3771d	BRCA2	c.5946delT		Breast cancer*Ova	Insufficient c
Rebbeck et al. 2016	French		F	BRCA1	c.3839_3843 d	BRCA2	c.1636delT		Ovarian cancer*	Insufficient c
Rebbeck et al. 2016	French		F	BRCA1	c.3839_3843 d	BRCA2	c.1636delT		Breast cancer*Ova	Insufficient c
Rebbeck et al. 2016	Korean		F	BRCA1	c.390C>A	BRCA2	c.3018delA		Breast cancer*	Insufficient c
Rebbeck et al. 2016	German		F	BRCA1	3910delG	BRCA2	c.2830A>T		Breast cancer*	Insufficient c
Rebbeck et al. 2016	Italian		F	BRCA1	c.3916_3917d	BRCA2	c.5380delG		Breast cancer*Ova	Insufficient c
Rebbeck et al. 2016	Caucasian (USA)		F	BRCA1	c.4065_4068d	BRCA2	c.5350_5351delAA		Breast cancer*	Insufficient c
Rebbeck et al. 2016	Caucasian (UK)		F	BRCA1	c.4186-?_435	BRCA2	c.2636_2637delCT		Breast cancer*	Insufficient c
Rebbeck et al. 2016	Danish		F	BRCA1	c.427G > T	BRCA2	c.8730delT		Breast cancer*	Insufficient c
Rebbeck et al. 2016	Korean		F	BRCA1	c.5030_5033 d	BRCA2	c.1399A > T		Breast cancer*	Insufficient c
Rebbeck et al. 2016	German		F	BRCA1	c.5123C > A	BRCA2	c.6275_6276delTT		Breast cancer*	Insufficient c
Rebbeck et al. 2016	Asian (USA)		F	BRCA1	c.5136G > A	BRCA2	c.4965delC		Breast cancer*	Insufficient c
Rebbeck et al. 2016	German		F	BRCA1	c.5193 + 1del	BRCA2	c.658_659delGT		Ovarian cancer*	Insufficient c
Rebbeck et al. 2016	Austrian		F	BRCA1	c.5251C>T	BRCA2	c.6753_6754delTT		Breast cancer*	Insufficient c
Rebbeck et al. 2016	Austrian		F	BRCA1	c.5266dupC	BRCA2	c.8364G>A		Breast cancer*	Insufficient c
Rebbeck et al. 2016	Jewish		F	BRCA1	c.5266dupC	BRCA2	c.5946delT		Breast cancer*	Insufficient c
Rebbeck et al. 2016	Jewish		F	BRCA1	c.5266dupC	BRCA2	c.5946delT		Breast cancer*	Insufficient c
Rebbeck et al. 2016	Jewish		F	BRCA1	c.5266dupC	BRCA2	c.5946delT		Breast cancer*	Insufficient c
Rebbeck et al. 2016	Jewish		F	BRCA1	c.5266dupC	BRCA2	c.5946delT		Breast cancer*Ova	Insufficient c
Rebbeck et al. 2016	German		F	BRCA1	c.5266dupC	BRCA2	c.4478_4481delAAAG		Breast cancer*	Insufficient c

Rebbeck et al. 2016	German		F	BRCA1	c.5266dupC	BRCA2	c.5645C > A			Breast cancer*Ova	Insufficient c
Rebbeck et al. 2016	Greek		F	BRCA1	c.5406 + 664_	BRCA2	c.9748dupT			Breast cancer*	Insufficient c
Rebbeck et al. 2016	German		F	BRCA1	c.548-?_4185	BRCA2	c.2269A > T			Breast cancer*	Insufficient c
Rebbeck et al. 2016	German		F	BRCA1	c.962G > A	BRCA2	c.2231C > G			Breast cancer*	Insufficient c
Rebbeck et al. 2016	German	-	F	BRCA1	c.68_69delAG	BRCA2	c.5722_5723delCT p.Leu1908Argfs			Breast cancer*	Insufficient c
Rebbeck et al. 2016	Asian (USA)		F	BRCA1	c.1016delA p.	BRCA2	c.7379_7382delACAAp.Asn2460fs			Breast cancer*	Insufficient c
Rebbeck et al. 2016	Hispanic		F	BRCA1	c.1390delAp.T	BRCA2	c.658_659delGTp.Val220fs			Breast cancer*	Insufficient c
Rebbeck et al. 2016	Asian (Korea)		F	BRCA1	c.1504_1508d	BRCA2	c.2798_2799delCAp.Thr933fs			Breast cancer*	Insufficient c
Rebbeck et al. 2016	German		F	BRCA1	c.1504_1508d	BRCA2	c.462_463delAAp.Arg155_Asp156i			Breast cancer*Ova	Insufficient c
Rebbeck et al. 2016	Italian		F	BRCA1	c.1687C>Tp.G	BRCA2	c.6469C>Tp.Gln2157Ter			Breast cancer*Ova	Insufficient c
Lavie et al. 2011	Jewish		F	BRCA1	185delAG	BRCA2	6174delT				Insufficient c
Lavie et al. 2011	Jewish		F	BRCA1	185delAG	BRCA2	6174delT				Insufficient c
Lavie et al. 2011	Jewish		F	BRCA1	185delAG	BRCA2	6174delT				Insufficient c
Lavie et al. 2011	Jewish		F	BRCA1	185delAG	BRCA2	6174delT				Insufficient c
Lavie et al. 2011	Jewish		F	BRCA1	185delAG	BRCA2	6174delT				Insufficient c
Lavie et al. 2011	Jewish		F	BRCA1	185delAG	BRCA2	6174delT				Insufficient c
Lavie et al. 2011	Jewish		F	BRCA1	185delAG	BRCA2	6174delT				Insufficient c
Lavie et al. 2011	Jewish		F	BRCA1	185delAG	BRCA2	6174delT				Insufficient c
Lavie et al. 2011	Jewish		F	BRCA1	185delAG	BRCA2	6174delT				Insufficient c
Lavie et al. 2011	Jewish		F	BRCA1	185delAG	BRCA2	6174delT				Insufficient c
Lavie et al. 2011	Jewish		F	BRCA1	185delAG	BRCA2	6174delT				Insufficient c
Lavie et al. 2011	Jewish		F	BRCA1	185delAG	BRCA2	6174delT				Insufficient c
Lavie et al. 2011	Jewish		F	BRCA1	185delAG	BRCA2	6174delT				Insufficient c
Lavie et al. 2011	Jewish		F	BRCA1	185delAG	BRCA2	6174delT				Insufficient c
Lavie et al. 2011	Jewish		M	BRCA1	185delAG	BRCA2	6174delT				Insufficient c
Lavie et al. 2011	Jewish		M	BRCA1	185delAG	BRCA2	6174delT				Insufficient c
Lavie et al. 2011	Jewish		M	BRCA1	185delAG	BRCA2	6174delT				Insufficient c
Lavie et al. 2011	Jewish		M	BRCA1	185delAG	BRCA2	6174delT				Insufficient c
Lavie et al. 2011	Jewish		F	BRCA1	5382insC	BRCA2	6174delT				Insufficient c
Lavie et al. 2011	Jewish		F	BRCA1	5382insC	BRCA2	6174delT				Insufficient c
Lavie et al. 2011	Jewish		M	BRCA1	5382insC	BRCA2	6174delT				Insufficient c
Lavie et al. 2011	Jewish		M	BRCA1	5382insC	BRCA2	6174delT				Insufficient c

linical details
linical details

linical details
linical details
linical details
linical details
linical details

linical details
linical details
linical details
linical details
linical details
linical details
linical details
linical details
linical details
linical details
linical details
linical details
linical details
linical details
linical details
linical details
linical details
linical details
linical details
linical details
linical details
linical details
linical details
linical details
linical details
linical details
linical details

linical details
linical details
linical details
linical details
linical details
linical details
linical details
linical details
linical details
linical details
linical details
linical details
linical details
linical details
linical details
linical details
linical details
linical details
linical details
linical details
linical details
linical details
linical details
linical details
linical details
linical details
linical details
linical details
linical details
linical details
linical details
linical details
linical details

linical details
linical details
linical details
linical details
linical details
linical details
linical details
linical details
linical details
linical details
linical details
linical details
linical details
linical details
linical details
linical details
linical details
linical details
linical details
linical details
linical details
linical details
linical details
linical details
linical details
linical details
linical details
linical details
linical details
linical details
linical details
linical details
