# Supplementary material for: Multi-locus inherited neoplasia alleles syndromes in cancer: implications for clinical practice
Source: Eur J Hum Genet. 2025 Jan 23;33(3):289–96. doi: 10.1038/s41431-025-01785-1 (PMC11894078; doi:10.1038/s41431-025-01785-1)
Supplement: Supplementary file 6 — Supplementary Materials 6: References for the MINAS cohort [file 41431_2025_1785_MOESM6_ESM.pdf]

Supplementary Materials 6: References for the MINAS cohort

1. Agaoglu NB, Doganay L. Concurrent pathogenic variations in patients with hereditary cancer syndromes. Eur J Med Genet. 2021;64(12):104366.
2. Agiannitopoulos K, Papadopoulou E, Tsaousis GN, Pepe G, Kampouri S, Patsea E, et al. Report of a germline double heterozygote in MSH2 and PALB2. Mol Genet Genomic Med. 2020;8(10):e1242.
3. Ahlborn LB, Steffensen AY, Jønson L, Djursby M, Nielsen FC, Gerdes AM, et al. Identification of a breast cancer family double heterozygote for RAD51C and BRCA2 gene mutations. Fam Cancer. 2015;14(1):129-33.
4. Ancot F, Arcand SL, Mes-Masson AM, Provencher DM, Tonin PN. Double PALB2 and BRCA1/BRCA2 mutation carriers are rare in breast cancer and breast-ovarian cancer syndrome families from the French Canadian founder population. Oncol Lett. 2015;9(6):2787-90.
5. Andrés R, Menao S, Arruebo M, Quílez E, Cardiel MJ. Double heterozygous mutation in the BRCA1 and ATM genes involved in development of primary metachronous tumours: a case report. Breast Cancer Res Treat. 2019;177(3):767-70.
6. Ataei-Kachouei M, Nadaf J, Akbari MT, Atri M, Majewski J, Riazalhosseini Y, et al. Double Heterozygosity of BRCA2 and STK11 in Familial Breast Cancer Detected by Exome Sequencing. Iran J Public Health. 2015;44(10):1348-52.
7. Augustyn AM, Agostino NM, Namey TL, Nair S, Martino MA. Two patients with germline mutations in both BRCA1 and BRCA2 discovered unintentionally: a case series and discussion of BRCA testing modalities. Breast Cancer Res Treat. 2011;129(2):629-34.
8. Balta G, Patiroglu T, Gumruk F. Fanconi Anemia and Ataxia Telangiectasia in Siblings who Inherited Unique Combinations of Novel FANCA and ATM Null Mutations. J Pediatr Hematol Oncol. 2019;41(3):243-6.
9. Bang YJ, Kwon WK, Nam SJ, Kim SW, Chae BJ, Lee SK, et al. Clinicopathological Characterization of Double Heterozygosity for BRCA1 and BRCA2 Variants in Korean Breast Cancer Patients. Cancer Res Treat. 2022;54(3):827-33.
10. Belanger MH, Dolman L, Arcand SL, Shen Z, Chong G, Mes-Masson AM, et al. A targeted analysis identifies a high frequency of BRCA1 and BRCA2 mutation carriers in women with ovarian cancer from a founder population. J Ovarian Res. 2015;8:1.
11. Bell K, Hodgson N, Levine M, Sadikovic B, Zbuk K. Double heterozygosity for germline mutations in BRCA1 and p53 in a woman with early onset breast cancer. Breast Cancer Res Treat. 2014;146(2):447-50.
12. Borg A, Isola J, Chen J, Rubio C, Johansson U, Werelius B, et al. Germline BRCA1 and HMLH1 mutations in a family with male and female breast carcinoma. Int J Cancer. 2000;85(6):796-800.
13. Brown SJ, Riconda DL, Zheng F, Jackson GL, Suo L, Robbins RJ. Features of Multiple Endocrine Neoplasia Type 1 and 2A in a Patient with Both RET and MEN1 Germline Mutations. J Endocr Soc. 2020;4(4):bvaa020.
14. Caldes T, de la Hoya M, Tosar A, Sulleiro S, Godino J, Ibañez D, et al. A breast cancer family from Spain with germline mutations in both the BRCA1 and BRCA2 genes. J Med Genet. 2002;39(8):e44.
15. Campos B, Balmaña J, Gardenyes J, Valenzuela I, Abad O, Fàbregas P, et al. Germline mutations in NF1 and BRCA1 in a family with neurofibromatosis type 1 and early-onset breast cancer. Breast Cancer Res Treat. 2013;139(2):597-602.
16. Carbajal-Mamani SL, Markham MJ, Santolaya-Forgas J, Castagno JC, Cardenas-Goicoechea J. Germline PALB2, ATM variants in a patient with breast and ovarian cancer at risk for familial cancer syndrome: Is there a role for risk-reducing salpingo-oophorectomy? Obstet Gynecol Sci. 2020;63(2):205-8.

17. Chatzikyriakou P, Touska P, Moonim MT, Obholzer R, Afridi S, Sandison A, et al. Case report of a man with multiple paragangliomas and pathogenic germline variants in both NF1 and SDHD. Cancer Genet. 2021;256-257:110-4.
18. Choi DH, Lee MH, Bale AE, Carter D, Haffty BG. Incidence of BRCA1 and BRCA2 mutations in young Korean breast cancer patients. J Clin Oncol. 2004;22(9):1638-45.
19. Claus EB, Petruzella S, Matloff E, Carter D. Prevalence of BRCA1 and BRCA2 mutations in women diagnosed with ductal carcinoma in situ. Jama. 2005;293(8):964-9.
20. Colombo M, Mondini P, Minenza E, Foglia C, Mosconi A, Molica C, et al. A novel BRCA1 splicing variant detected in an early onset triple-negative breast cancer patient additionally carrying a pathogenic variant in ATM: A case report. Front Oncol. 2023;13:1102184.
21. Cote S, Arcand SL, Royer R, Nolet S, Mes-Masson AM, Ghadirian P, et al. The BRCA2 c.9004G>A (E2002K) [corrected] variant is likely pathogenic and recurs in breast and/or ovarian cancer families of French Canadian descent. Breast Cancer Res Treat. 2012;131(1):333-40.
22. D'Elia G, Caliendo G, Passariello L, Albanese L, Makker J, Molinari AM, et al. Hereditary Cancer Syndrome in a Family with Double Mutation in BRIP1 and MUTYH Genes. Genes (Basel). 2023;14(2).
23. de la Hoya M, Osorio A, Godino J, Sulleiro S, Tosar A, Perez-Segura P, et al. Association between BRCA1 and BRCA2 mutations and cancer phenotype in Spanish breast/ovarian cancer families: implications for genetic testing. Int J Cancer. 2002;97(4):466-71.
24. Del Valle J, Rofes P, Moreno-Cabrera JM, López-Dóriga A, Belhadj S, Vargas-Parra G, et al. Exploring the Role of Mutations in Fanconi Anemia Genes in Hereditary Cancer Patients. Cancers (Basel). 2020;12(4).
25. Duzkale Teker N, Eyerci N. Double Heterozygous Mutations in the BRCA2 and ATM Genes: A Case Report and Review of the Literature. Breast Care (Basel). 2021;16(4):412-7.
26. Ercolino T, Lai R, Giachè V, Melchionda S, Carella M, Delitala A, et al. Patient affected by neurofibromatosis type 1 and thyroid C-cell hyperplasia harboring pathogenic germ-line mutations in both NF1 and RET genes. Gene. 2014;536(2):332-5.
27. Ferrer-Avargues R, Castillejo MI, Dámaso E, Díez-Obrero V, Garrigos N, Molina T, et al. Co-occurrence of germline pathogenic variants for different hereditary cancer syndromes in patients with Lynch syndrome. Cancer Commun (Lond). 2021;41(3):218-28.
28. Foppiani L, Forzano F, Ceccherini I, Bruno W, Ghiorzo P, Caroli F, et al. Uncommon association of germline mutations of RET proto-oncogene and CDKN2A gene. Eur J Endocrinol. 2008;158(3):417-22.
29. Fostira F, Kostantopoulou I, Apostolou P, Papamentzelopoulou MS, Papadimitriou C, Faliakou E, et al. One in three highly selected Greek patients with breast cancer carries a loss-of-function variant in a cancer susceptibility gene. J Med Genet. 2020;57(1):53-61.
30. Frank-Raue K, Rondot S, Hoeppner W, Goretzki P, Raue F, Meng W. Coincidence of multiple endocrine neoplasia types 1 and 2: mutations in the RET protooncogene and MEN1 tumor suppressor gene in a family presenting with recurrent primary hyperparathyroidism. J Clin Endocrinol Metab. 2005;90(7):4063-7.
31. Friedman E, Bar-Sade Bruchim R, Kruglikova A, Risel S, Levy-Lahad E, Halle D, et al. Double heterozygotes for the Ashkenazi founder mutations in BRCA1 and BRCA2 genes. Am J Hum Genet. 1998;63(4):1224-7.
32. Ghataorhe P, Kurian AW, Pickart A, Trapane P, Norton JA, Kingham K, et al. A carrier of both MEN1 and BRCA2 mutations: case report and review of the literature. Cancer Genet Cytogenet. 2007;179(2):89-92.
33. Goehringer C, Sutter C, Kloor M, Gebert J, Slater EP, Keller M, et al. Double germline mutations in APC and BRCA2 in an individual with a pancreatic tumor. Fam Cancer. 2017;16(2):303-9.

34. Gong P, Charles S, Rosenblum N, Wang Z, Witkiewicz AK. A case of endometrial cancer in the context of a BRCA2 mutation and double heterozygosity for Lynch syndrome. Gynecol Oncol Case Rep. 2012;2(3):69-72.
35. Harada R, Matsubayashi H, Kiyozumi Y, Kobayashi H, Mitsuya K, Imai K, et al. A Japanese case of ovarian mucinous adenocarcinoma with germline double variants of MSH2 and BRCA2. J Hum Genet. 2023;68(11):783-7.
36. Heidemann S, Fischer C, Engel C, Fischer B, Harder L, Schlegelberger B, et al. Double heterozygosity for mutations in BRCA1 and BRCA2 in German breast cancer patients: implications on test strategies and clinical management. Breast Cancer Res Treat. 2012;134(3):1229-39.
37. Huang W, Bian J, Qian X, Shao L, Li H, Zhang L, et al. Case Report: Coinheritance of Germline Mutations in APC and BRCA1 in Colorectal Cancer. Front Oncol. 2021;11:658389.
38. Hur JY, Kim JY, Ahn JS, Im YH, Lee J, Kwon M, et al. Clinical Characteristics of Korean Breast Cancer Patients Who Carry Pathogenic Germline Mutations in Both BRCA1 and BRCA2: A Single-Center Experience. Cancers (Basel). 2020;12(5).
39. Infante M, Arranz-Ledo M, Lastra E, Abella LE, Ferreira R, Orozco M, et al. Increased Co-Occurrence of Pathogenic Variants in Hereditary Breast and Ovarian Cancer and Lynch Syndromes: A Consequence of Multigene Panel Genetic Testing? Int J Mol Sci. 2022;23(19).
40. Kámory E, Tanyi M, Kolacsek O, Olasz J, Tóth L, Damjanovich L, et al. Two germline alterations in mismatch repair genes found in a HNPCC patient with poor family history. Pathol Oncol Res. 2006;12(4):228-33.
41. Kashiwada T, Shimizu H, Tamura K, Seyama K, Horie Y, Mizoo A. Birt-Hogg-Dubé syndrome and familial adenomatous polyposis: an association or a coincidence? Intern Med. 2012;51(13):1789-92.
42. Kast K, Neuhann TM, Görgens H, Becker K, Keller K, Klink B, et al. Germline truncating-mutations in BRCA1 and MSH6 in a patient with early onset endometrial cancer. BMC Cancer. 2012;12:531.
43. Kilmartin DJ, Mooney DJ, Acheson RW, Payne SJ, Maher ER, Eustace P. von Hippel-Lindau disease and familial polyposis coli in the same family. Arch Ophthalmol. 1996;114(10):1294.
44. Kobayashi H, Ohno S, Sasaki Y, Matsuura M. Hereditary breast and ovarian cancer susceptibility genes (review). Oncol Rep. 2013;30(3):1019-29.
45. Koren-Michowitz M, Friedman E, Gershoni-Baruch R, Brok-Simoni F, Patael Y, Rechavi G, et al. Coinheritance of BRCA1 and BRCA2 mutations with Fanconi anemia and Bloom syndrome mutations in Ashkenazi Jewish population: possible role in risk modification for cancer development. Am J Hematol. 2005;78(3):203-6.
46. Laish I, Friedman E, Levi-Reznick G, Kedar I, Katz L, Levi Z, et al. Double heterozygotes of BRCA1/BRCA2 and mismatch repair gene pathogenic variants: case series and clinical implications. Breast Cancer Res Treat. 2021;188(3):685-94.
47. Le Duc D, Hentschel J, Neuser S, Stiller M, Meier C, Jäger E, et al. In cis TP53 and RAD51C pathogenic variants may predispose to sebaceous gland carcinomas. Eur J Hum Genet. 2021;29(3):489-94.
48. Le Page C, Rahimi K, Rodrigues M, Heinzelmann-Schwarz V, Recio N, Tommasi S, et al. Clinicopathological features of women with epithelial ovarian cancer and double heterozygosity for BRCA1 and BRCA2: A systematic review and case report analysis. Gynecol Oncol. 2020;156(2):377-86.
49. Leegte B, van der Hout AH, Deffenbaugh AM, Bakker MK, Mulder IM, ten Berge A, et al. Phenotypic expression of double heterozygosity for BRCA1 and BRCA2 germline mutations. Journal of Medical Genetics. 2005;42(3):e20.
50. Liede A, Rehal P, Vesprini D, Jack E, Abrahamson J, Narod SA. A breast cancer patient of Scottish descent with germ-line mutations in BRCA1 and BRCA2. Am J Hum Genet. 1998;62(6):1543-4.

51. Lindor NM, Smyrk TC, Buehler S, Gunawardena SR, Thomas BC, Limburg P, et al. Multiple jejunal cancers resulting from combination of germline APC and MLH1 mutations. Fam Cancer. 2012;11(4):667-9.
52. Lorca V, Rueda D, Martín-Morales L, Fernández-Aceñero MJ, Grolleman J, Poves C, et al. Contribution of New Adenomatous Polyposis Predisposition Genes in an Unexplained Attenuated Spanish Cohort by Multigene Panel Testing. Sci Rep. 2019;9(1):9814.
53. Loubser F, de Villiers JN, van der Merwe NC. Two double heterozygotes in a South African Afrikaner family: implications for BRCA1 and BRCA2 predictive testing. Clin Genet. 2012;82(6):599-600.
54. Madar L, Majoros V, Szűcs Z, Nagy O, Babicz T, Butz H, et al. Double Heterozygosity for Rare Deleterious Variants in the BRCA1 and BRCA2 Genes in a Hungarian Patient with Breast Cancer. Int J Mol Sci. 2023;24(20).
55. Mai PL, Best AF, Peters JA, DeCastro RM, Khincha PP, Loud JT, et al. Risks of first and subsequent cancers among TP53 mutation carriers in the National Cancer Institute Li-Fraumeni syndrome cohort. Cancer. 2016;122(23):3673-81.
56. Mampel A, Sottile ML, Denita-Juárez SP, Vargas AL, Vargas-Roig LM. Double heterozygous pathogenic variants in the BRCA1 and BRCA2 genes in a patient with bilateral metachronous breast cancer. Cancer Genet. 2022;260-261:14-7.
57. Manoukian S, Peissel B, Pensotti V, Barile M, Cortesi L, Stacchiotti S, et al. Germline mutations of TP53 and BRCA2 genes in breast cancer/sarcoma families. Eur J Cancer. 2007;43(3):601-6.
58. Mastroianno S, Torlontano M, Scillitani A, D'Aloiso L, Verrienti A, Bonfitto N, et al. Coexistence of multiple endocrine neoplasia type 1 and type 2 in a large Italian family. Endocrine. 2011;40(3):481-5.
59. Megid TBC, Barros-Filho MC, Pisani JP, Achatz MI. Double heterozygous pathogenic variants prevalence in a cohort of patients with hereditary breast cancer. Front Oncol. 2022;12:873395.
60. Meynard G, Mansi L, Lebahar P, Villanueva C, Klajer E, Calcagno F, et al. First description of a double heterozygosity for BRCA1 and BRCA2 pathogenic variants in a French metastatic breast cancer patient: A case report. Oncol Rep. 2017;37(3):1573-8.
61. Michaeli O, Ladany H, Erez A, Ben Shachar S, Izraeli S, Lidzbarsky G, et al. Di-genic inheritance of germline POLE and PMS2 pathogenic variants causes a unique condition associated with pediatric cancer predisposition. Clin Genet. 2022;101(4):442-7.
62. Momozawa Y, Iwasaki Y, Parsons MT, Kamatani Y, Takahashi A, Tamura C, et al. Germline pathogenic variants of 11 breast cancer genes in 7,051 Japanese patients and 11,241 controls. Nat Commun. 2018;9(1):4083.
63. Monnerat C, Chompret A, Kannengiesser C, Avril MF, Janin N, Spatz A, et al. BRCA1, BRCA2, TP53, and CDKN2A germline mutations in patients with breast cancer and cutaneous melanoma. Fam Cancer. 2007;6(4):453-61.
64. Morak M, Massdorf T, Sykora H, Kerscher M, Holinski-Feder E. First evidence for digenic inheritance in hereditary colorectal cancer by mutations in the base excision repair genes. Eur J Cancer. 2011;47(7):1046-55.
65. Mur P, García-Mulero S, del Valle J, Magraner-Pardo L, Vidal A, Pineda M, et al. Role of POLE and POLD1 in familial cancer. Genetics in Medicine. 2020;22(12):2089-100.
66. Musolino A, Naldi N, Michiara M, Bella MA, Zanelli P, Bortesi B, et al. A breast cancer patient from Italy with germline mutations in both the BRCA1 and BRCA2 genes. Breast Cancer Res Treat. 2005;91(2):203-5.
67. Na R, Zheng SL, Han M, Yu H, Jiang D, Shah S, et al. Germline Mutations in ATM and BRCA1/2 Distinguish Risk for Lethal and Indolent Prostate Cancer and are Associated with Early Age at Death. Eur Urol. 2017;71(5):740-7.

68. Nakahara M, Yokozaki H, Yasui W, Dohi K, Tahara E. Identification of concurrent germ-line mutations in hMSH2 and/or hMLH1 in Japanese hereditary nonpolyposis colorectal cancer kindreds. Cancer Epidemiol Biomarkers Prev. 1997;6(12):1057-64.
69. Ng PS, Wen WX, Fadlullah MZ, Yoon SY, Lee SY, Thong MK, et al. Identification of germline alterations in breast cancer predisposition genes among Malaysian breast cancer patients using panel testing. Clin Genet. 2016;90(4):315-23.
70. Njoroge SW, Burgess KR, Cobleigh MA, Alnajar HH, Gattuso P, Usha L. Hereditary diffuse gastric cancer and lynch syndromes in a BRCA1/2 negative breast cancer patient. Breast Cancer Res Treat. 2017;166(1):315-9.
71. Noh JM, Choi DH, Nam SJ, Lee JE, Kim JW, Kim SW, et al. Characteristics of double heterozygosity for BRCA1 and BRCA2 germline mutations in Korean breast cancer patients. Breast Cancer Res Treat. 2012;131(1):217-22.
72. Nomizu T, Matsuzaki M, Katagata N, Kobayashi Y, Sakuma T, Monma T, et al. A case of familial breast cancer with double heterozygosity for BRCA1 and BRCA2 genes. Breast Cancer. 2015;22(5):557-61.
73. Nurmi AK, Suvanto M, Dennis J, Aittomäki K, Blomqvist C, Nevanlinna H. Pathogenic Variant Spectrum in Breast Cancer Risk Genes in Finnish Patients. Cancers (Basel). 2022;14(24).
74. Occhi G, Trivellin G, Ceccato F, De Lazzari P, Giorgi G, Demattè S, et al. Prevalence of AIP mutations in a large series of sporadic Italian acromegalic patients and evaluation of CDKN1B status in acromegalic patients with multiple endocrine neoplasia. Eur J Endocrinol. 2010;163(3):369-76.
75. Ohmoto A, Morizane C, Kubo E, Takai E, Hosoi H, Sakamoto Y, et al. Germline variants in pancreatic cancer patients with a personal or family history of cancer fulfilling the revised Bethesda guidelines. J Gastroenterol. 2018;53(10):1159-67.
76. Palmirotta R, Lovero D, Stucci LS, Silvestris E, Quaresmini D, Cardascia A, et al. Double Heterozygosity for BRCA1 Pathogenic Variant and BRCA2 Polymorphic Stop Codon K3326X: A Case Report in a Southern Italian Family. Int J Mol Sci. 2018;19(1).
77. Papi L, Palli D, Masi L, Putignano AL, Congregati C, Zanna I, et al. Germline mutations in MEN1 and BRCA1 genes in a woman with familial multiple endocrine neoplasia type 1 and inherited breast-ovarian cancer syndromes: a case report. Cancer Genet Cytogenet. 2009;195(1):75-9.
78. Pearlman R, Frankel WL, Swanson B, Zhao W, Yilmaz A, Miller K, et al. Prevalence and Spectrum of Germline Cancer Susceptibility Gene Mutations Among Patients With Early-Onset Colorectal Cancer. JAMA Oncol. 2017;3(4):464-71.
79. Pedroni M, Di Gregorio C, Cortesi L, Reggiani Bonetti L, Magnani G, Simone ML, et al. Double heterozygosity for BRCA1 and hMLH1 gene mutations in a 46-year-old woman with five primary tumors. Tech Coloproctol. 2014;18(3):285-9.
80. Penkert J, Schmidt G, Hofmann W, Schubert S, Schieck M, Auber B, et al. Breast cancer patients suggestive of Li-Fraumeni syndrome: mutational spectrum, candidate genes, and unexplained heredity. Breast Cancer Res. 2018;20(1):87.
81. Pern F, Bogdanova N, Schürmann P, Lin M, Ay A, Länger F, et al. Mutation analysis of BRCA1, BRCA2, PALB2 and BRD7 in a hospital-based series of German patients with triple-negative breast cancer. PLoS One. 2012;7(10):e47993.
82. Pinto C, Pinheiro M, Peixoto A, Santos C, Veiga I, Rocha P, et al. Co-occurrence of nonsense mutations in MSH6 and MSH2 in Lynch syndrome families evidencing that not all truncating mutations are equal. J Hum Genet. 2016;61(2):151-6.
83. Plon SE, Pirics ML, Nuchtern J, Hicks J, Russell H, Agrawal S, et al. Multiple tumors in a child with germ-line mutations in TP53 and PTEN. N Engl J Med. 2008;359(5):537-9.
84. Ramus SJ, Friedman LS, Gayther SA, Ponder BA, Bobrow L, van der Looji M, et al. A breast/ovarian cancer patient with germline mutations in both BRCA1 and BRCA2. Nat Genet. 1997;15(1):14-5.
85. Randall TC, Bell KA, Rebane BA, Rubin SC, Boyd J. Germline mutations of the BRCA1 and BRCA2 genes in a breast and ovarian cancer patient. Gynecol Oncol. 1998;70(3):432-4.

86. Raygada M, Raffeld M, Bernstein A, Miettinen M, Glod J, Hughes MS, et al. Case report of adrenocortical carcinoma associated with double germline mutations in MSH2 and RET. Am J Med Genet A. 2021;185(4):1282-7.
87. Rebbeck TR, Friebel TM, Mitra N, Wan F, Chen S, Andrulis IL, et al. Inheritance of deleterious mutations at both BRCA1 and BRCA2 in an international sample of 32,295 women. Breast Cancer Res. 2016;18(1):112.
88. Sánchez Castro EE, Ziegler-Rodriguez G, Castro Mujica MDC. Double heterozygous mutation in RAD50 and ATM genes in a Peruvian family with five cancer types: a case report. Rev Fac Cien Med Univ Nac Cordoba. 2022;79(1):53-6.
89. Sarkadi B, Baghy K, Sápi Z, Nyirő G, Likó I, Patócs A. Germline BRCA1 Mutation Detected in a Multiple Endocrine Neoplasia Type 2 Case With RET Codon 634 Mutation. Front Genet. 2019;10:544.
90. Schamschula E, Kinzel M, Wernstedt A, Oberhuber K, Gottschling H, Schnaiter S, et al. Teenage-Onset Colorectal Cancers in a Digenic Cancer Predisposition Syndrome Provide Clues for the Interaction between Mismatch Repair and Polymerase δ Proofreading Deficiency in Tumorigenesis. Biomolecules. 2022;12(10).
91. Scheenstra R, Rijcken FE, Koornstra JJ, Hollema H, Fodde R, Menko FH, et al. Rapidly progressive adenomatous polyposis in a patient with germline mutations in both the APC and MLH1 genes: the worst of two worlds. Gut. 2003;52(6):898-9.
92. Schubert SA, Ruano D, Tiersma Y, Drost M, de Wind N, Nielsen M, et al. Digenic inheritance of MSH6 and MUTYH variants in familial colorectal cancer. Genes Chromosomes Cancer. 2020;59(12):697-701.
93. Sekido Y, Ohigashi S, Takahashi T, Hayashi N, Suzuki K, Hirota S. Familial Gastrointestinal Stromal Tumor with Germline KIT Mutations Accompanying Hereditary Breast and Ovarian Cancer Syndrome. Anticancer Res. 2017;37(3):1425-31.
94. Shani H, Bernstein-Molho R, Laitman Y, Netzer I, Friedman E. Double heterozygosity for TP53 and BRCA1 mutations: clinical implications in populations with founder mutations. Breast Cancer Res Treat. 2021;186(1):259-63.
95. Silva-Smith R, Sussman DA. Co-occurrence of Lynch syndrome and juvenile polyposis syndrome confirmed by multigene panel testing. Fam Cancer. 2018;17(1):87-90.
96. Smith M, Fawcett S, Sigalas E, Bell R, Devery S, Andrieska N, et al. Familial breast cancer: double heterozygosity for BRCA1 and BRCA2 mutations with differing phenotypes. Fam Cancer. 2008;7(2):119-24.
97. Sokolenko AP, Bogdanova N, Kluzniak W, Preobrazhenskaya EV, Kuligina ES, Iyevleva AG, et al. Double heterozygotes among breast cancer patients analyzed for BRCA1, CHEK2, ATM, NBN/NBS1, and BLM germ-line mutations. Breast Cancer Res Treat. 2014;145(2):553-62.
98. Soravia C, DeLozier CD, Dobbie Z, Berthod CR, Arrigoni E, Bründler MA, et al. Double frameshift mutations in APC and MSH2 in the same individual. Int J Colorectal Dis. 2006;21(1):79-83.
99. Sorscher S, Ansley K, Delaney SD, Ramkissoon S. The implications of BRCA loss of heterozygosity (LOH) and deficient mismatch repair gene (dMMR) expression in the breast cancer of a patient with both inherited breast and ovarian cancer syndrome (BRCA2) and Lynch syndrome (MLH1). Breast Cancer Res Treat. 2020;180(2):511-4.
100. Spannuth WA, Thaker PH, Sood AK. Concomitant BRCA1 and BRCA2 gene mutations in an Ashkenazi Jewish woman with primary breast and ovarian cancer. Am J Obstet Gynecol. 2007;196(4):e6-9.
101. Steffensen AY, Jønson L, Ejlertsen B, Gerdes AM, Nielsen FC, Hansen TV. Identification of a Danish breast/ovarian cancer family double heterozygote for BRCA1 and BRCA2 mutations. Fam Cancer. 2010;9(3):283-7.
102. Stradella A, del Valle J, Rofes P, Feliubadaló L, Grau Garces È, Velasco À, et al. Does multilocus inherited neoplasia alleles syndrome have severe clinical expression? Journal of Medical Genetics. 2019;56(8):521.

103. Sukumar J, Kassem M, Agnese D, Pilarski R, Ramaswamy B, Sweet K, et al. Concurrent germline BRCA1, BRCA2, and CHEK2 pathogenic variants in hereditary breast cancer: a case series. Breast Cancer Res Treat. 2021;186(2):569-75.
104. Suspitsin EN, Yanus GA, Sokolenko AP, Yatsuk OS, Zaitseva OA, Bessonov AA, et al. Development of breast tumors in CHEK2, NBN/NBS1 and BLM mutation carriers does not commonly involve somatic inactivation of the wild-type allele. Med Oncol. 2014;31(2):828.
105. Taeubner J, Brozou T, Qin N, Bartl J, Ginzel S, Schaper J, et al. Congenital embryonal rhabdomyosarcoma caused by heterozygous concomitant PTCH1 and PTCH2 germline mutations. Eur J Hum Genet. 2018;26(1):137-42.
106. Tang VT, Arscott P, Helms AS, Day SM. Whole-Exome Sequencing Reveals GATA4 and PTEN Mutations as a Potential Digenic Cause of Left Ventricular Noncompaction. Circ Genom Precis Med. 2018;11(1):e001966.
107. Tedaldi G, Tebaldi M, Zampiga V, Danesi R, Arcangeli V, Ravegnani M, et al. Multiple-gene panel analysis in a case series of 255 women with hereditary breast and ovarian cancer. Oncotarget. 2017;8(29):47064-75.
108. Tesoriero A, Andersen C, Southey M, Somers G, McKay M, Armes J, et al. De novo BRCA1 mutation in a patient with breast cancer and an inherited BRCA2 mutation. Am J Hum Genet. 1999;65(2):567-9.
109. Thiffault I, Hamel N, Pal T, McVety S, Marcus VA, Farber D, et al. Germline truncating mutations in both MSH2 and BRCA2 in a single kindred. Br J Cancer. 2004;90(2):483-91.
110. Tsaousis GN, Papadopoulou E, Agiannitopoulos K, Pepe G, Tsoulos N, Boukovinas I, et al. Revisiting the Implications of Positive Germline Testing Results Using Multi-gene Panels in Breast Cancer Patients. Cancer Genomics Proteomics. 2022;19(1):60-78.
111. Tung ML, Chandra B, Dillahunt K, Gosse MD, Sato TS, Sidhu A. Co-occurrence of VHL and SDHA Pathogenic Variants: A Case Report. Front Oncol. 2022;12:925582.
112. Uhrhammer N, Bignon YJ. Report of a family segregating mutations in both the APC and MSH2 genes: juvenile onset of colorectal cancer in a double heterozygote. Int J Colorectal Dis. 2008;23(11):1131-5.
113. Vahteristo P, Tamminen A, Karvinen P, Eerola H, Eklund C, Aaltonen LA, et al. p53, CHK2, and CHK1 genes in Finnish families with Li-Fraumeni syndrome: further evidence of CHK2 in inherited cancer predisposition. Cancer Res. 2001;61(15):5718-22.
114. Valle L, Rodríguez-López R, Robledo M, Benítez J, Urioste M. Concurrence of germline mutations in the APC and PTEN genes in a colonic polyposis family member. J Clin Oncol. 2004;22(11):2252-3.
115. Vibert R, Hasnaoui J, Perrier A, Lefebvre A, Colas C, Dhooge M, et al. Lynch syndrome: influence of additional susceptibility variants on cancer risk. Eur J Hum Genet. 2023;31(9):1078-82.
116. Vietri MT, Caliendo G, D'Elia G, Resse M, Casamassimi A, Minucci PB, et al. Five Italian Families with Two Mutations in BRCA Genes. Genes (Basel). 2020;11(12).
117. Vietri MT, D'Elia G, Caliendo G, Casamassimi A, Resse M, Passariello L, et al. Double mutation of APC and BRCA1 in an Italian family. Cancer Genet. 2020;244:32-5.
118. Vietri MT, Molinari AM, Caliendo G, De Paola ML, Giovanna D, Gambardella AL, et al. Double heterozygosity in the BRCA1 and BRCA2 genes in Italian family. Clin Chem Lab Med. 2013;51(12):2319-24.
119. Whitworth J, Skytte AB, Sunde L, Lim DH, Arends MJ, Happerfield L, et al. Multilocus Inherited Neoplasia Alleles Syndrome: A Case Series and Review. JAMA Oncol. 2016;2(3):373-9.
120. Yamamoto T, Isomura M, Xu Y, Liang J, Yagasaki H, Kamachi Y, et al. PTPN11, RAS and FLT3 mutations in childhood acute lymphoblastic leukemia. Leuk Res. 2006;30(9):1085-9.
121. Yılmaz A, Mirili C, Bilici M, Tekin SB. Colorectal cancer in Lynch syndrome associated with PMS2 and MSH6 mutations. Int J Colorectal Dis. 2020;35(2):351-3.
122. Zajac V, Tomka M, Ilenciková D, Májek P, Stevurková V, Kirchhoff T. A double germline mutations in the APC and p53 genes. Neoplasma. 2000;47(6):335-41.

123. Zbuk KM, Patocs A, Shealy A, Sylvester H, Miesfeldt S, Eng C. Germline mutations in PTEN and SDHC in a woman with epithelial thyroid cancer and carotid paraganglioma. Nat Clin Pract Oncol. 2007;4(10):608-12.
124. Zheng H, Yuan M, Wu H, Chen R, Gao Y. Case Report: Double Germline Mutations in BRCA1 and MSH2 in a Patient With Mixed Serous-Endometrioid Endometrial Carcinoma. Front Med (Lausanne). 2020;7:581982.
125. Zuradelli M, Peissel B, Manoukian S, Zaffaroni D, Barile M, Pensotti V, et al. Four new cases of double heterozygosity for BRCA1 and BRCA2 gene mutations: clinical, pathological, and family characteristics. Breast Cancer Res Treat. 2010;124(1):251-8.
